# Matching Monomers to Metals: Quantified Linear Free Energy Relationships in Metal-Catalyzed Ring-Opening Polymerizations

**DOI:** 10.1021/jacs.6c06048

**Published:** 2026-08-14

**Authors:** Rosie Thorogood, Thomas M. McGuire, Charlotte K. Williams

**Affiliations:** Department of Chemistry, Chemistry Research Laboratory, 150602 University of Oxford, 12 Mansfield Road, Oxford OX1 3TA, United Kingdom

## Abstract

Despite the importance of metal catalysts for cyclic ester and -carbonate ring-opening polymerizations, quantified and generally applicable catalyst structure-performance relationships remain elusive. Here, a generally applicable linear correlation between the metal active site Lewis acidity, as described by the metal cation p*K*_h_ value, and the ring-opening polymerization rate is presented. The linear relationship applies to five different metal catalysts, each used to polymerize five different commercial monomers, including ε-caprolactone, δ-valerolactone, γ-methyl ε-caprolactone, trimethylene carbonate, and 2,2-dimethyltrimethylene carbonate. Using microscale calorimetry, the polymerization rate coefficients and transition state barriers are determined for polymerizations using commercial metal­(2-ethylhexanoate)/benzyl alcohol catalyst systems, where the metal = Sn­(II), Zn­(II), Mg­(II), Bi­(III), Zr­(IV). All polymerizations are quantified under industrially relevant conditions, i.e., at high temperatures (140–200 °C), low catalyst loadings (1:1000 metal:monomer), and either high concentrations ([monomer]_0_ = 6.25 M) or using neat monomer melts. The generally applicable linear free energy relationships, consistent with the coordination–insertion polymerization mechanism and supported by monomer spectroscopy characterization, reveal that Sn­(II) is the best for cyclic ester polymerizations, while Zn­(II) is the best for cyclic carbonate polymerizations. The linear free energy relationship predicts that l-lactide will be polymerized fastest using Sn­(II) catalysts, a finding which is verified experimentally. It also enables improvements to the activity of Mg­(II) catalysts, through ligand modifications, increasing polymerization rates by 3-fold. The generally applicable linear free energy relationships are important to identify the most effective monomer-metal catalyst partnerships, to select optimum polymerization processes, and to streamline future catalyst design targeting next-generation sustainable polymers.

## Graphical abstract

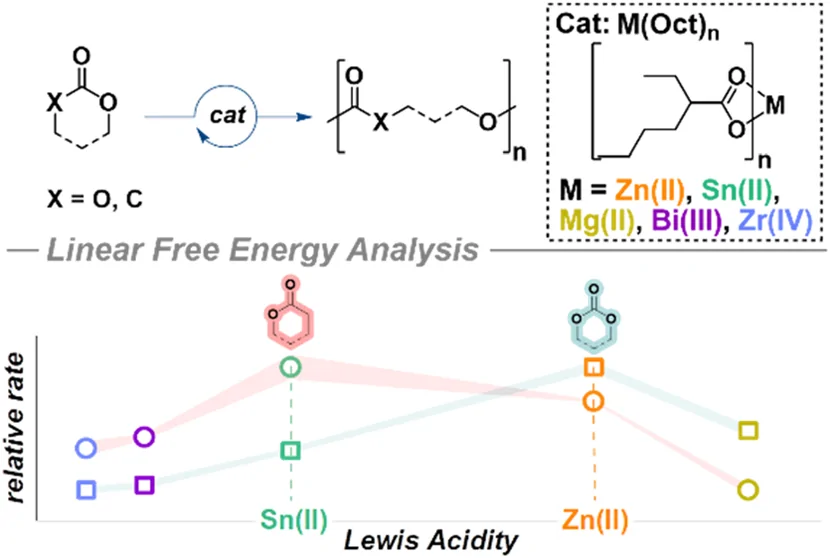

## Introduction

Plastics are essential for future technologies; however, their integration with fossil fuel extraction, high material stability, and durability contributes to pollution both through growing carbon dioxide emissions and microplastics.^1,2^ In the future, we need to move to a more circular plastics economy, replacing single-use, fossil-sourced plastics with materials derived from renewable resources and enabling low-energy, efficient polymer recycling.^3–5^ Polyesters and polycarbonates are important sustainable polymers since carbon dioxide emissions are reduced by monomers sourced from biomass or even directly from carbon dioxide and, after use, they undergo low-energy and high efficiency (re)-processing, chemical recycling to monomer or biodegradation to metabolites.^3–5^ These polymers are efficiently produced using cyclic ester or -carbonate ring-opening polymerizations (ROP); these methods are used commercially to make poly­(l-lactide) (PLLA), poly­(caprolactone) (PCL), and poly­(trimethylene carbonate) (PTMC).^6–8^ Increasing the uptake of these sustainable polymers requires fast and selective polymerization catalysts able to operate under industry manufacturing conditions, including at high temperatures, in neat monomer melts, and using low catalyst loadings.^8–10^

Many metals are known to catalyze cyclic ester or -carbonate ring-opening polymerizations, but Sn­(II), Zn­(II), and Mg­(II) complexes are among those showing very high rates and operating at low loadings.^9,11–34^ Tin­(II)­bis­(ethylhexanoate) or tin octoate, Sn­(Oct)_2_, used with alcohols (ROH), is very widely applied due to its impressive activity, ability to operate at very low catalyst loadings, and stability to high temperature melt phase polymerizations (conducted in neat monomer).^15^ Pioneering research from Penczek and co-workers addressed the kinetics and mechanisms for Sn­(Oct)_2_/ROH catalyst systems for both ε-caprolactone (CL) and l-lactide (l-LA) polymerizations. These studies support the now widely accepted coordination–insertion mechanism for the ring-opening polymerization of cyclic esters and carbonates (Figure 1).^13,15–18,20^ They discovered that cyclic ester ring-opening polymerizations were most effective when conducted with at least an equivalent (vs Sn­(Oct)_2_) of alcohol (ROH); the active catalyst is proposed to be a [(Oct)­Sn­(OR)] complex (Figure 1).^13,15–18,20^ Initiation occurs by cyclic ester ring-opening by the active catalyst Sn-OR (OR comes from added alcohol) to form a propagating Sn-alkoxide intermediate (where the alkoxide forms by ring-opening the first cyclic ester monomer). The polymerization rate law is first-order in both catalyst and monomer concentrations, with the rate-determining step being proposed to comprise both monomer coordination and activation at the Sn­(II) center and Sn-alkoxide nucleophilic attack at the monomer ester group (Figure 1). Monomer acyl bond cleavage and ring-opening (re)­forms the propagating Sn-alkoxide species. Computational studies provided further insight into the propagating mechanism during catalysis (Figure 1).^23,35–37^

**Figure 1:**
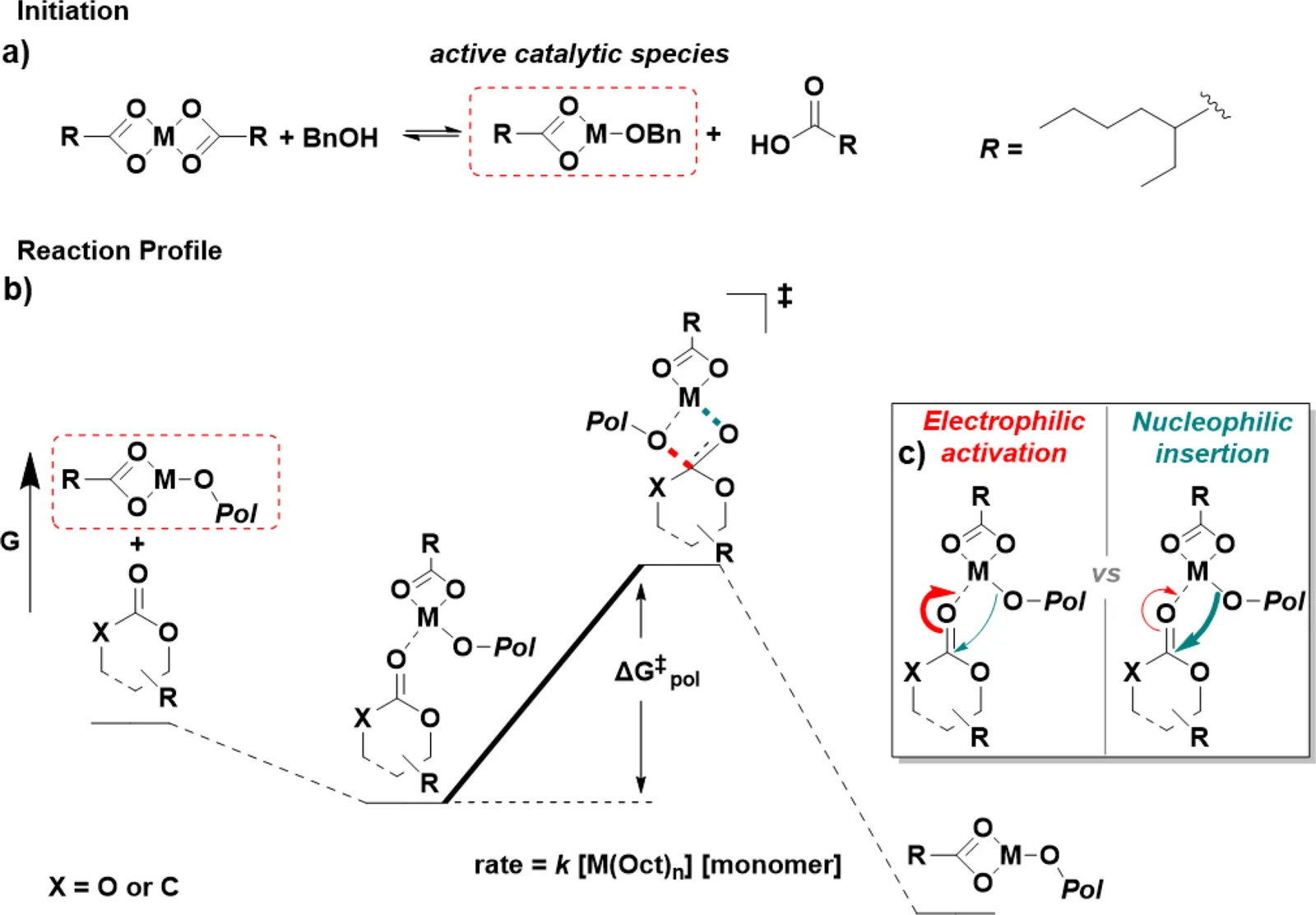
Coordination–insertion mechanism for the ring-opening polymerization of lactones (X = C) and cyclic carbonates (X = O) using M­(Oct)_n_/ROH catalyst systems. (a) The initiation step proposed to form the active catalytic species. (b) Polymerization catalysis energy profile, indicating the likely rate-determining step and the associated transition state energy barrier (Δ*G*_pol_^‡^). (c) Key features for the metal catalyst in the rate-determining step.

In another important study, Penczek and co-workers established that the related zinc catalyst system, i.e., Zn­(Oct)_2_/ROH, followed the same rate law and was proposed to form a related active site catalyst, [(Oct)­Zn­(OR)].^20,38^ They showed that, under equivalent conditions, caprolactone (CL) polymerization occurred faster using (Oct)­Zn­(OR) than with (Oct)­Sn­(OR) ([CL] = 2 M, THF, 80 °C).^39^ Confusingly, other researchers reported the opposite rate trends, finding that CL polymerization was faster for Sn­(II) than Zn­(II) catalysts (neat CL, 150 °C).^40^ Using ligated but homoleptic catalysts, the groups of Herres-Pawlis and Jones independently reported that Zn­(II) catalysts showed very high rates in melt l-lactide (l-LA) polymerizations;^41,42^ these rates exceeded those using (Oct)­Sn­(OR), although the two metals are not compared under equivalent coordination environments.^32^ The successes using Zn­(II) catalysts motivated exploration of Mg­(II) catalysts as lightweight and earth-abundant metal alternatives.^11,19,25–29,31,42–45^ There are no direct comparisons between Zn­(Oct)_2_/ROH and Mg­(Oct)_2_/ROH catalyst systems, but comparisons have been made between various heteroleptic (ligated) Zn­(II) and Mg­(II) catalysts. For example, Darensbourg and co-workers showed that trimethylene carbonate (TMC) was polymerized faster using a Mg­(II)­(Schiff base) catalyst than the analogous Zn­(II) complex.^43^ In contrast, Tolman and Hillmyer investigated both lactide (LA) and CL polymerization, using Mg­(II) and Zn­(II) complexes coordinated by another type of Schiff base ligand, a diphenolate-tetra-amine ligand.^44^ For LA ROP, the catalysis was faster using Zn­(II) than Mg­(II), but the opposite applied to CL ROP, where the Mg­(II) catalyst was faster than the Zn­(II) complex. Later, Jones and co-workers compared Zn­(II) and Mg­(II) complexes, coordinated by a very similar Schiff base ligand, showing that in melt l-lactide polymerizations the Zn­(II) catalyst was faster than the Mg­(II) analogue.^42^ Very recently, Lamberti and Mazzeo compared (N-heterocyclic carbene)­Zn­(II) or Mg­(II) amido complexes, each used with alcohol, for trimethylene carbonate ROP, showing that Mg­(II) > Zn­(II), but when using 2,2-dimethyltrimethylene carbonate (DTC) or l-LA, the polymerization rates were reversed, with Zn­(II) > Mg­(II).^45^ These prior results are quite hard to interpret, showing contradictory results in terms of rate trends using similar ligands with the same metals and monomers. It is important to recognize that while catalytic performance is strongly impacted by metal choice, ligand design is also fundamental to achieving a high-performance catalyst, and there are many prior studies that investigate in depth the effects of ligand variations for a given metal, commonly to achieve stereoselectivity in polymerizations.^19,29^ These studies can require complex multistep ancillary ligand syntheses, and the synthetic accessibility of ligated metal complexes may be metal dependent (notably Mg­(II) and Zn­(II) complexes are more widely explored with ligand variations than Sn­(II)).^22,28,41^ Therefore, this work seeks to measure rates using simple metal carboxylate catalysts studied under the same conditions and to use the resulting kinetic data to quantify the trends in catalyst structure-activity relationships.

Linear free energy relationships correlate the free energy and activation energy for reactions. They are widely used in catalysis to both quantify and understand the key parameters influencing rates.^46^ We propose that they may be valuable in heterocycle ring-opening polymerization catalysis to quantify and correlate trends in rates (or activation energies) against catalyst ground state descriptors, such as metal ionic radius or metal Lewis acidity.^46,47^ In 2023, we established linear free energy relationships for bimetallic catalysts used in the ring-opening copolymerization of epoxides with carbon dioxide or with anhydrides.^48,49^ These heterometallic catalysts show exponential correlations between polymerization rates and the s-block metal Lewis acidity, as measured by the p*K*_a_ of the metal aqua complex cation, i.e., they show true linear free energy relationships. Our work was inspired by pioneering research that established linear free energy relationships between electrochemical redox potentials and s- and f-block metal Lewis acidity using related bimetallic complexes.^50,51^ Last year, we established another linear free energy relationship correlating the rates of oxygenated polymer recycling reactions with metal catalyst Lewis acidity, again as measured by p*K*_h_ values, and using simple metal carboxylate catalysts.^52^ The structure-performance relationship in recycling catalysis was shown to apply to different polyesters and polycarbonates, with the best catalyst always being Zn­(Oct)_2_.^47,52^ In the recycling catalysis, the polymer chain is proposed to react with the catalyst via its alkoxide chain end-group, undergoing intramolecular attack at a carbonyl group in the chain, followed by ring-closing to extrude the cyclic monomer.^47^ Viewed simply, the depolymerization is the reverse of polymerization, indeed many of these heterocycle ring-opening polymerizations are textbook chemical equilibria.^53,54^ It is less obvious, however, that the same rate-determining transition states and intermediates apply to both polymerization and depolymerization catalysis. Thus, this work seeks to establish whether quantified catalyst structure-performance relationships and linear free energy relationships apply to simple metal carboxylate catalysts that are widely used in common cyclic monomer ring-opening polymerizations. To ensure that catalysts are compared under the most broadly useful conditions, polymerization rates and transition state parameters, from Eyring analyses, are determined at high temperatures, low catalyst concentrations, and high monomer concentrations, i.e., conditions relevant to large-scale polymer manufacturing. If linear free energy relationships are uncovered, their generality should be proven and utility should be tested in two ways: (1) prediction of the most active catalyst for a new (to the study) monomer used in ring-opening polymerization and (2) logical improvement in catalyst activity for a known monomer-metal pairing in the series by tuning active site descriptors.

## Results and Discussion

### Catalyst and Monomer Selection

We selected a series of commercial and widely applied metal catalysts that are all metal­(2-ethylhexanoate) complexes, M­(Oct)_n_, where M = Zn­(II), Sn­(II), Mg­(II), Bi­(III), Zr­(IV). The metals were selected for their precedence in ring-opening polymerization catalysis and because they cover a range of Lewis acidity values (as assessed by p*K*_h_ values).^9,55–59^ The use of aqueous metal hydrolysis constants as a descriptor for metal catalyst Lewis acidity was investigated by ^31^P­{^1^H} NMR spectroscopy utilizing a trioctylphosphine oxide (TOPO) probe which provided experimental support for the trends in Lewis acidity values (Figures S1 and S2).^60,61^ The metals have different oxidation states, which means a variable number of carboxylate ligands and potentially different numbers of initiating sites. To compensate for this oxidation state variability, an excess of benzyl alcohol (10 equiv vs catalyst) was applied in all polymerizations. The excess alcohol was used to ensure that all polymers have a consistent molar mass and to drive the catalyst equilibria toward the active alkoxide initiator, thereby helping standardize rate comparisons (Figure 1).^13,39^

Five common cyclic monomers were selected for investigation: ε-caprolactone (CL), δ-valerolactone (VL), γ-methyl ε-caprolactone (MeCL), trimethylene carbonate (TMC), and 2,2-dimethyltrimethylene carbonate (DTC).^47,63^ All monomers propagate via a primary alkoxide group, and hence should both show fast rates and allow for understanding of the influences of the different catalysts and monomer pairings. Some of these monomers are already used to make commercial polymers by ring-opening polymerization, for example, VL, CL, and TMC.^7,64^ Others are selected to investigate substituent influences, because they form useful amorphous polymers, or because the monomers can be sourced from biobased feedstocks; it has already been established that all the resulting polymers are fully chemically recyclable to monomer (Figure 2).^7,64–66^

**Figure 2:**
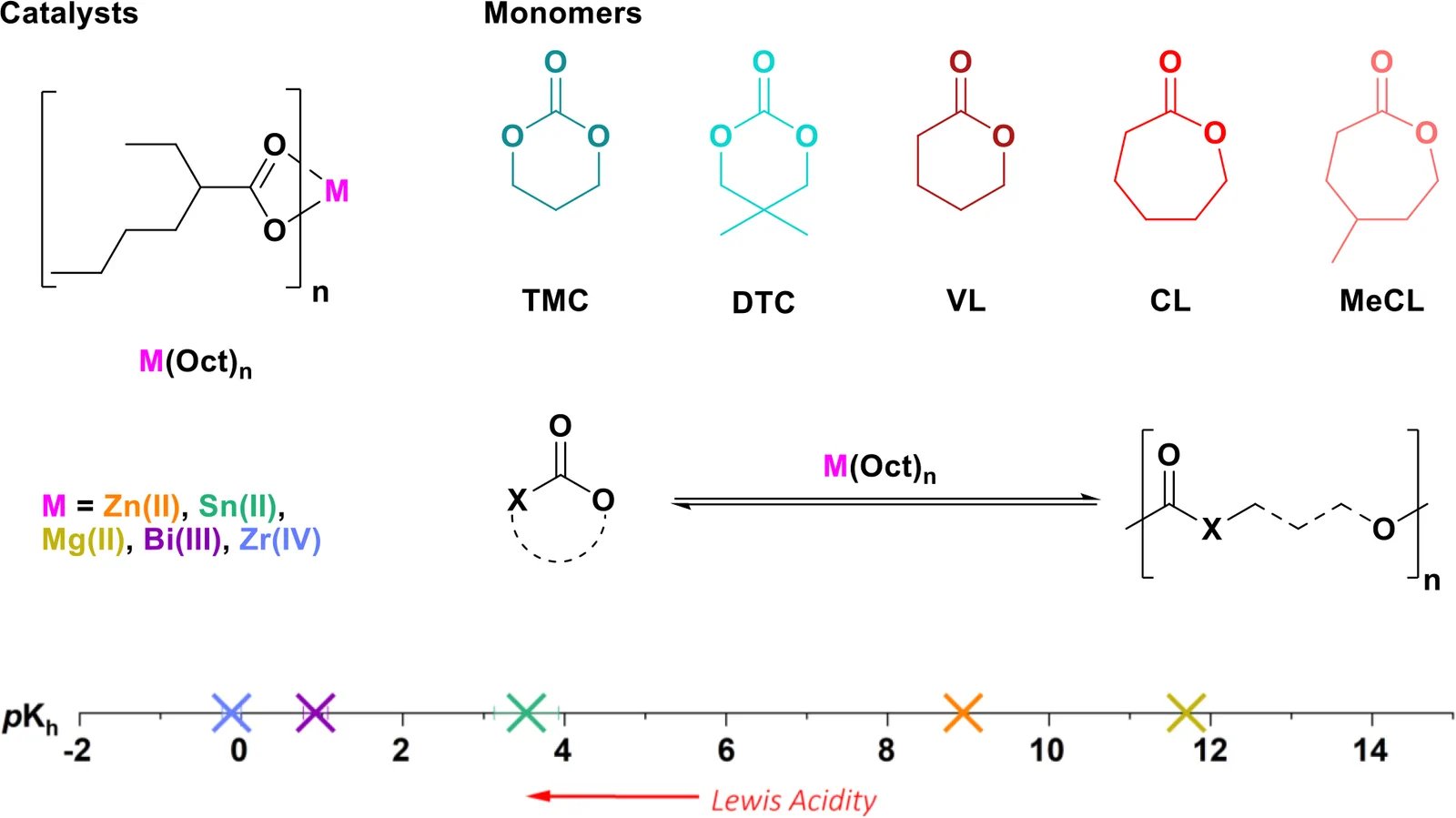
Structures of the metal carboxylate catalysts and the monomers investigated in this work, alongside the metal p*K*_h_ values, which are inversely related to Lewis acidity.^62^

### Polymerization Kinetics and Rate Law

For each monomer, the polymerization kinetics were measured using a recently reported microscale methodology, applying differential scanning calorimetry (DSC), which allows for precise measurement of the reaction heat change under isothermal conditions.^67^ Using this method enables fast and reliable quantification of the polymerization rate constant, *k*_obs_, for each monomer and catalyst combination over a range of temperatures. It is important to note that prior work establishes that the rates obtained using the methodology (DSC instrument) are both comparable to, and in some cases more accurate than, the rates measured in lab-scale experiments, either using *in situ* Fourier-transform infrared (FTIR) spectroscopy or by regular aliquot analysis to determine monomer conversion.^67^ In each experiment, demanding conditions are used, including catalyst loadings of M­(Oct)_n_:BnOH:monomer 1:10:1000 (molar ratios), fixed temperatures over the range 140–200 °C, and high monomer concentrations of [monomer]_0_ = 6.25 M in 1,2-dimethoxybenzene, with the solvent selected for its high specific heat capacity, high boiling point, and as a recommended green solvent.^67^ The M­(Oct)_n_:BnOH ratio of 1:10 was selected to drive the catalyst equilibria toward the active alkoxide initiator (Figure 1).^13,39^

To illustrate how the data are interpreted, the procedure to analyze the ring-opening polymerization of valerolactone (VL) using the Zn­(Oct)_2_ catalyst is described here, with the same methods used for all other combinations of monomers and catalysts (Figure 3 and Supporting Information). The catalyst, alcohol, and monomer mixture was loaded into the DSC pan, which was sealed, and the sample was loaded into the instrument and rapidly heated to 160 °C. At 160 °C, the polymerization heat flow vs time data were measured, with reaction completion indicated by constant heat flow values (Figure 3a). The exponential change in heat flow, during the polymerization, is exactly what would be expected in a reaction showing a first-order dependence on monomer concentration and is fully consistent with the coordination–insertion mechanism (Figure 3a).^67–69^ Once the reaction was complete, the sample was rapidly cooled, weighed to confirm mass balance, and both polymer and monomer conversions were externally calibrated by ^1^H NMR spectroscopy. To provide results in a format more familiar to those working in polymerization catalysis, heat flow vs time data are integrated and normalized for polymer conversion (using the NMR calibration), which produces plots of monomer or polymer concentration (conversion) vs time (Figure 3b–d). The resulting plots of [PVL] vs time clearly fit an exponential function (*R* ^2^ = 0.998), consistent with a first-order rate dependence on monomer concentration (Figure 3d). The pseudo-first-order polymerization rate constant, *k*_obs_, is obtained as the gradient of either the exponential or semilogarithmic fits (Figure 3d). For all polymerizations, the reported rates are the average of three independent polymerization experiments and reported errors are the standard error of the mean of the three repeats (Figure S3 and Table S1). For PVL catalysis using Zn­(Oct)_2_/BnOH, the polymerization rate coefficient, *k*_obs_, was 1.09 ± 0.01 × 10^–3^ s^–1^. In this case, and all other polymerization catalysis reactions, errors are low and rates are highly reproducible.

**Figure 3:**
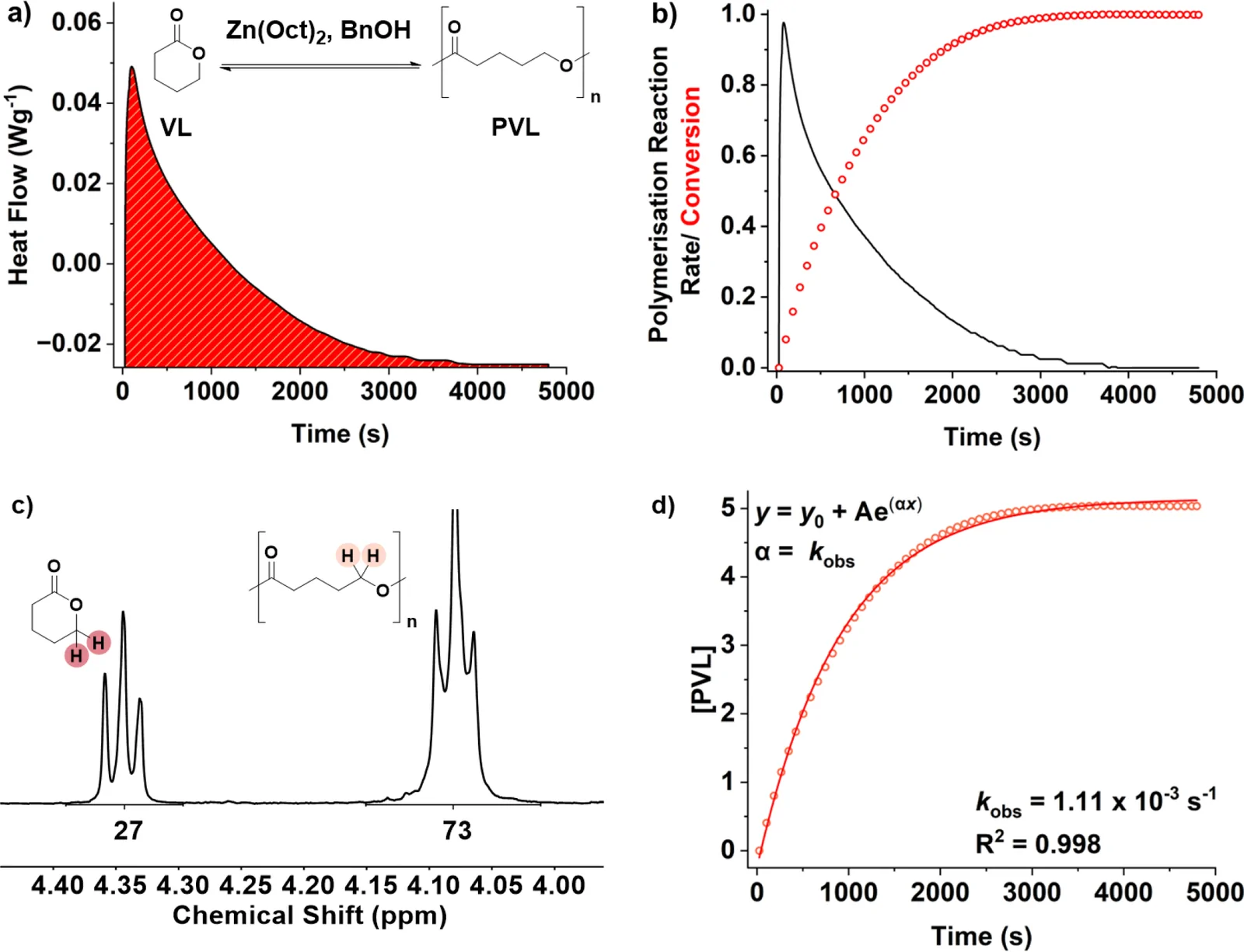
Kinetic analysis of cyclic ester and -carbonate ring-opening polymerization (ROP) catalysis. (a) Plot showing the polymerization heat flow vs time for the ROP of VL to PVL, with a Zn­(Oct)_2_ catalyst at 160 °C. (b) Plot of polymerization rate (black line) or PVL conversion (red open circles) vs time, with the PVL conversion data obtained by normalization and integration of the rate data (for conversion between 0 and 1). (c) ^1^H NMR spectrum of the crude reaction mixture, i.e., the aliquot used to determine PVL conversion by integration of peaks at 4.35 ppm (VL) and 4.08 ppm (PVL). (d) Plot of [PVL] vs time, obtained by calibrating plot (b) with respect to the conversion determined in plot (c). For clarity, every 800th data point from the data set is shown. The polymerization rate constant (*k*_obs_) is then determined from exponential fitting. Polymerization conditions: 1:10:1000 Zn­(Oct)_2_:BnOH:VL, [VL] = 6.25 M in 1,2-dimethoxybenzene.

Next, the Zn­(Oct)_2_/BnOH-catalyzed polymerization rate coefficient, at 160 °C and 6.25 M monomer concentration, was determined using the other monomers. These monomers include two further lactones, CL and MeCL, and two cyclic carbonates, TMC and DTC (Figure 2, Figures S3–S21, and Tables S1–S5). The cyclic carbonates all show significantly faster rates of polymerization than the lactones (Figure 4). The highest performing monomer was TMC, achieving a *k*_obs_ = 8.94 ± 0.80 × 10^–3^ s^–1^, while MeCL was the slowest to polymerize and exhibited a *k*_obs_ over an order of magnitude lower. All polymers had similar molar masses, consistent with the excess alcohol used in the reactions (Tables S1–S5 and Figure 1).^13,39^

**Figure 4:**
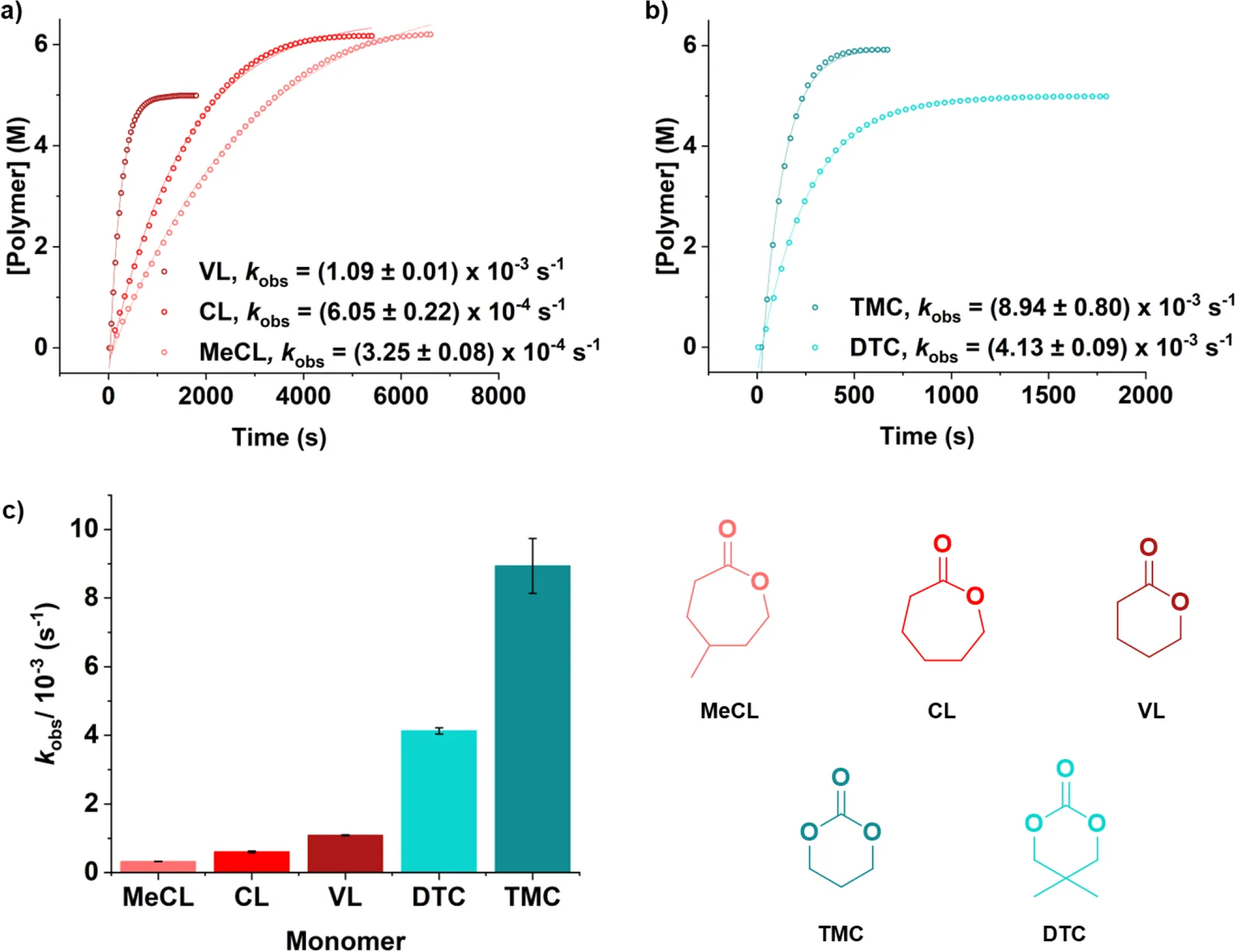
Ring-opening polymerization catalysis using Zn­(Oct)_2_/BnOH and a range of different cyclic monomers. (a) Plot of [Polymer] vs time used to determine the *k*_obs_ for each lactone monomer, i.e., VL, CL, and MeCL. (b) Plot of [Polymer] vs time used to determine the *k*_obs_ for each cyclic carbonate monomer, i.e., TMC and DTC. For clarity, every 800th data point from the data sets is shown. (c) Bar chart showing the polymerization rate constants, *k*_obs_, for each monomer and the structures of those monomers. Polymerization conditions: 1:10:1000 Zn­(Oct)_2_:BnOH:monomer, [monomer] = 6.25 M in 1,2-dimethoxybenzene, 160 °C.

The influence of the catalyst concentration on the polymerization rates was investigated using the fastest monomer, TMC. As expected, decreasing the catalyst concentration, [Zn­(Oct)_2_], led to a decrease in the polymerization rate. Plots of *k*_obs_ vs [Zn­(Oct)_2_] and ln­(*k*_obs_) vs ln­([Zn­(Oct)_2_]) are both linear, with the double logarithmic plot having a gradient of 1; both data sets indicate a first-order rate dependence on catalyst concentration (Figures S22–S24 and Table S6). The rate law is consistent with the coordination–insertion mechanism (Figure 1):^38^

$$\mathrm{rate}=k\left[\mathrm{Zn}{\left(\mathrm{Oct}\right)}_{2}\right]\left[\mathrm{monomer}\right]$$

### Eyring Analyses

Eyring analysis was used to determine the transition state enthalpy, Δ*H*_pol_^‡^, entropy, Δ*S*_pol_^‡^, and free energy barrier, Δ*G*_pol_^‡^. For example, five VL polymerizations were conducted, using Zn­(Oct)_2_/BnOH as the catalyst, over a range of different temperatures, and the rates were measured (Figure 5). Plots of ln­(*k*_p_/*T*) vs 1/*T* enabled determination of the transition state energies (Figure 5, Figure S8, and Table S1). The polymerization transition state entropy (Δ*S*_pol_^‡^) is negative, consistent with the associative rate-determining step involving monomer coordination at the metal active site (Figure 1). The polymerization transition state enthalpy (Δ*H*_pol_^‡^) is positive, likely due to bond breaking occurring during metal-alkoxide insertion and cyclic monomer ring-opening in the rate-determining step (Figure 1).

**Figure 5:**
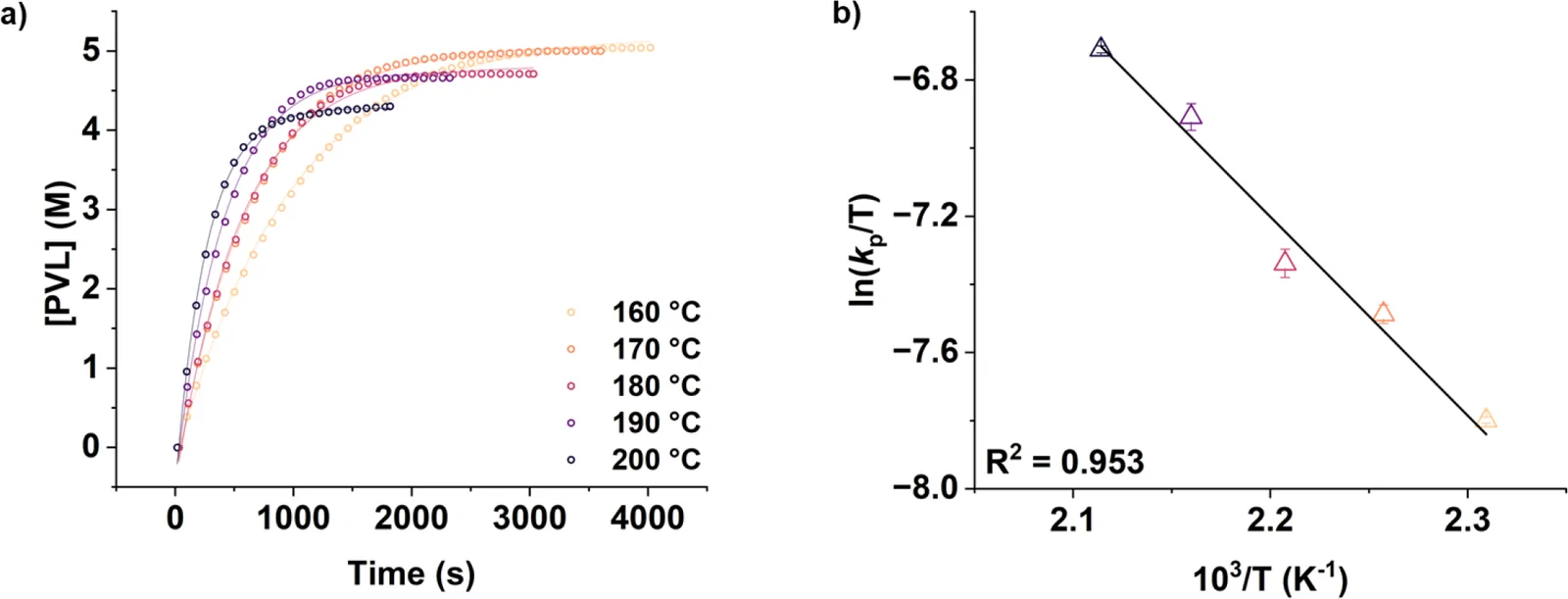
Influence of polymerization temperature on the transition state enthalpy, entropy, and free energy values for VL ROP using Zn­(Oct)_2_/BnOH as the catalyst. (a) Plots of [PVL] vs time, with associated exponential fits, for polymerizations conducted at systematically varied temperatures from 160 to 200 °C. For clarity, every 600th data point from the data sets is shown. The *k*_p_ values are determined as (*k*_obs_ average at each temperature)/[Zn­(Oct)_2_]_0_, where [Zn­(Oct)_2_]_0_ = 0.00625 M. Determined rate constants, *k*_p_, were *k*_p_ (160 °C) = 0.18 ± 0.002 M^–1^ s^–1^, *k*_p_ (170 °C) = 0.25 ± 0.007 M^–1^ s^–1^, *k*_p_ (180 °C) = 0.29 ± 0.012 M^–1^ s^–1^, *k*_p_ (190 °C) = 0.46 ± 0.018 M^–1^ s^–1^, and *k*_p_ (200 °C) = 0.58 ± 0.006 M^–1^ s^–1^. (b) Plot of ln­(*k*_p_/*T*) vs 1/*T*, where *T* = temperature in Kelvin (the Eyring plot) used to determine the transition state enthalpy and entropy values: Δ*H*_pol_^‡^ = 48.5 ± 6.2 kJ mol^–1^, Δ*S*_pol_^‡^ = −150.8 ± 13.8 J mol^–1^ K^–1^, and Δ*G*_pol_^‡^ (433 K) = 113.7 ± 8.8 kJ mol^–1^. Polymerization conditions: 1:10:1000 Zn­(Oct)_2_:BnOH:VL, [VL] = 6.25 M in 1,2-dimethoxybenzene.

### Comparing Metal Catalysts

The polymerization rates for each catalyst, i.e., M­(Oct)_n_, where M = Zn­(II), Sn­(II), Mg­(II), Bi­(III), and Zr­(IV), were measured for all of the different monomers, under equivalent conditions, to enable comparisons (Figure 6). Overall, the fastest catalysts for all of the monomers are Sn­(Oct)_2_ and Zn­(Oct)_2_. Interestingly, there is a distinct monomer-metal relationship: all the lactones (MeCL, CL, and VL) are polymerized fastest using the Sn­(Oct)_2_ catalyst, while the cyclic carbonates all achieve the highest rates of polymerization with Zn­(Oct)_2_. For example, in the polymerization of TMC, Zn­(Oct)_2_ shows 4 times higher rates than Sn­(Oct)_2_ (*k*_obs_ = 8.94 × 10^–3^ s^–1^ and 2.04 × 10^–3^ s^–1^, respectively), whereas for CL polymerization, Sn­(Oct)_2_ catalyst is 6 times faster than Zn­(Oct)_2_ (*k*_obs_ = 3.65 × 10^–3^ s^–1^ and 6.05 × 10^–4^ s^–1^, respectively).

**Figure 6:**
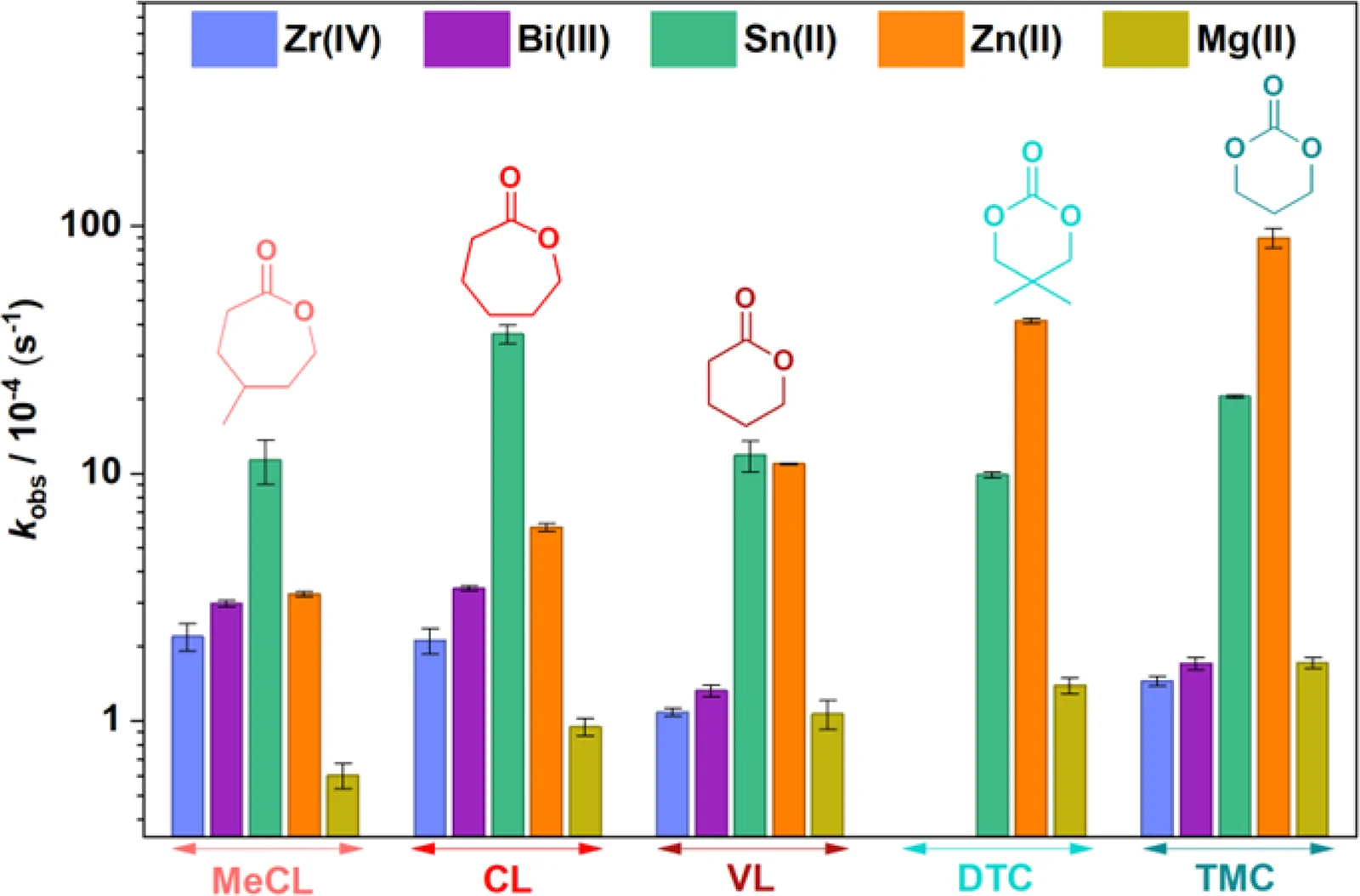
Bar chart of polymerization rate constants, *k*_obs_, for each monomer tested with each M­(Oct)_n_ catalyst. Polymerization conditions: 1:10:1000 M­(Oct)_n_:BnOH:monomer, [monomer] = 6.25 M in 1,2-dimethoxybenzene, 160 °C. Catalysts Bi­(Oct)_3_ and Zr­(Oct)_4_ showed negligible activity for DTC polymerization under these conditions. Note that a plot of rate constant normalized for the number of octoate chains per metal center shows the same relationship and the LFER is upheld under conditions of 1 equiv BnOH (Table S7 and Figures S25–S27). To reassure that possible effects of aggregation were not contributing to the lower observed performance for the Mg­(Oct)_2_ species, the order in polymerization rate with respect to [Mg­(Oct)_2_] was studied and shown to be first-order with DOSY NMR spectroscopy also confirming the catalyst speciation (Table S8 and Figures S28–S31).

### Ring-Opening Polymerization Catalysis: Linear Free Energy Relationships

To understand whether there are any relationships between polymerization rates and catalyst descriptors, rate correlations were explored with respect to metal oxophilicity and, then, against metal ionic radius. There were no significant correlations between rates and either of these metal properties (Figures S32 and S33). Next, the relationship between the polymerization rate and the metal Lewis acidity, as assessed by the metal p*K*_h_ value, was investigated (Figure 7). In this case, there is a clear linear free energy relationship linking the polymerization rate coefficient (*k*_obs_) to the metal catalyst (Lewis) acidity value (Figure 7). Indeed, for each of the monomers, the double-log plots (log­(*k*_obs_) vs metal p*K*_h_) show a characteristic “volcano” shape. Such plots indicate that the best catalysts have intermediate Lewis acidity values (Figure 7). Interestingly, the lactones and cyclic carbonates peak at different metals: the cyclic carbonates achieve the highest rates with Zn­(Oct)_2_, while the lactones show the greatest rates with Sn­(Oct)_2_ (Figure 7).

**Figure 7:**
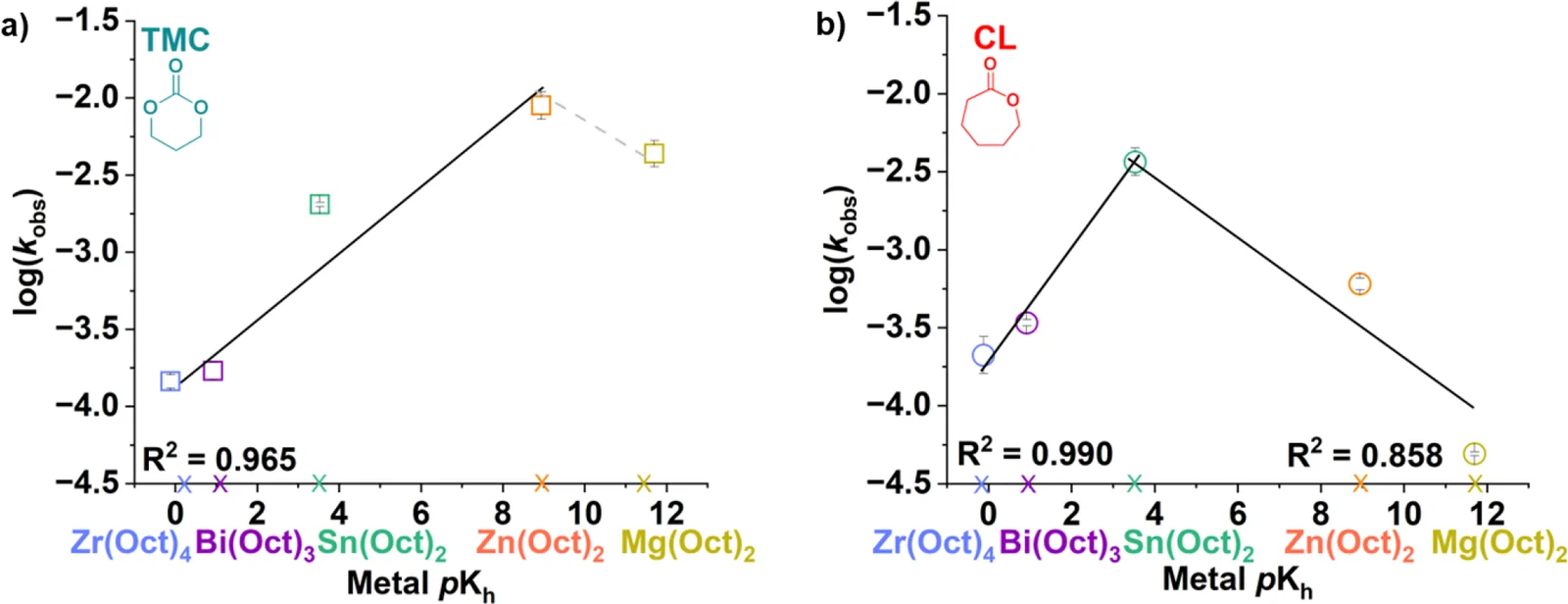
Linear free energy relationships in cyclic ester and -carbonate polymerization catalysis. (a) Plot of log­(*k*_obs_) vs metal p*K*_h_ for trimethylene carbonate (TMC) polymerization. (b) Plot of log­(*k*_obs_) vs metal p*K*_h_ for caprolactone (CL) polymerization. Polymerization conditions: 1:10:1000 M­(Oct)_n_:BnOH:monomer, [monomer] = 6.25 M in 1,2-dimethoxybenzene, 160 °C.

Each of the five monomers conforms to the same linear free energy relationship linking polymerization rate coefficient, *k*_obs_, to metal acidity (Figure 8). Plotting the normalized relative rates for all monomers, with the width of the line representing the distribution of different rates within each monomer subclass, clearly illustrates the generality of the linear free energy relationship (Figure 8).

**Figure 8:**
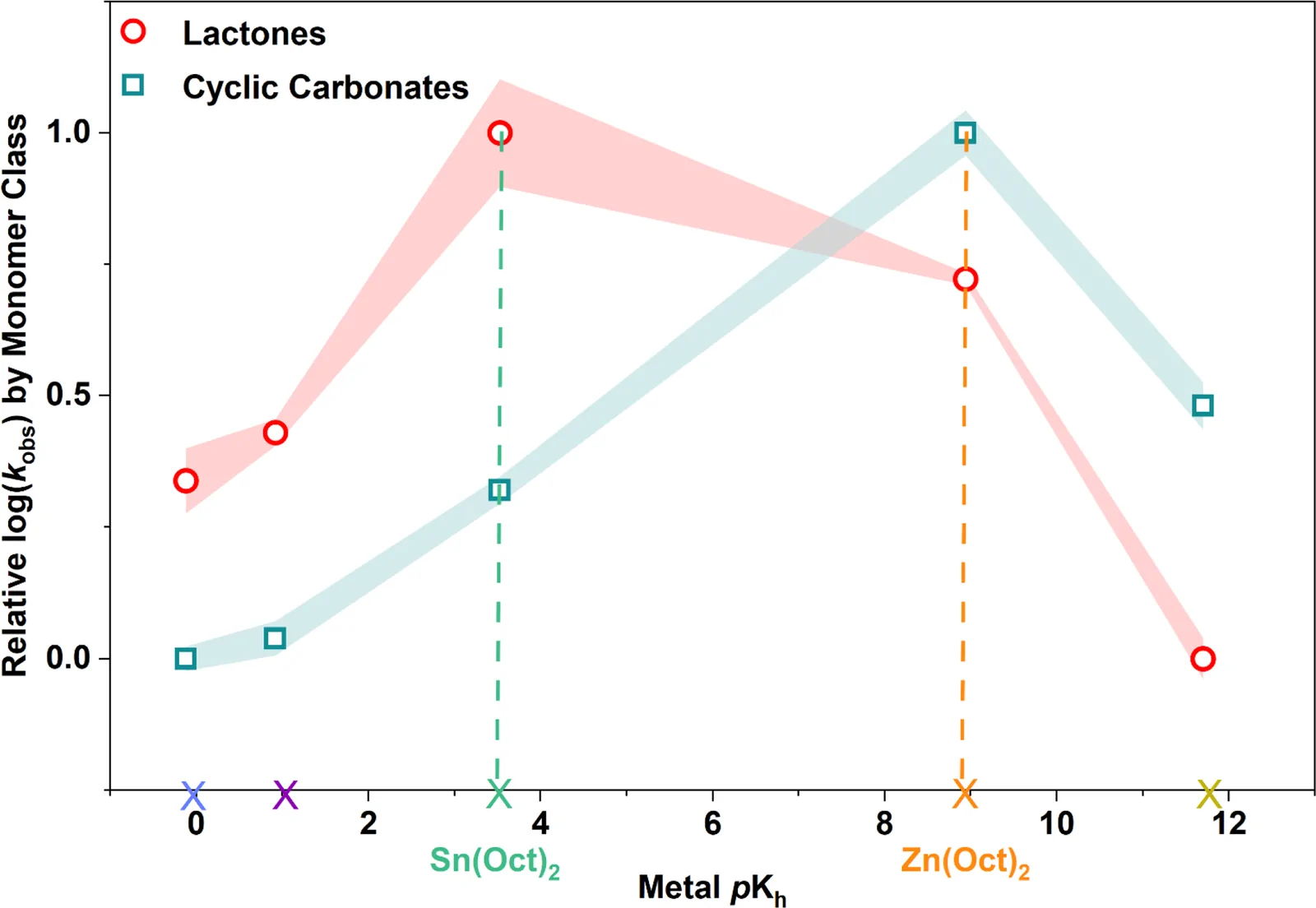
Plot of relative log­(*k*_obs_) vs metal p*K*_h_, highlighting the difference in optimum active site Lewis acidity for the two different monomer subclasses (lactone and cyclic carbonate). The log­(*k*_obs_) values for each monomer subclass are calculated as the average of the normalized log­(*k*_obs_) values for each monomer and M­(Oct)_n_ catalyst. The shaded areas indicate the error range for the normalized values. It is noted that the Bi­(Oct)_3_ catalyst is included in the correlation, sitting between the Zr­(Oct)_4_ and Sn­(Oct)_2_ catalysts, though MALDI analyses of end groups suggest a more complex mechanism for this catalyst (Figures S34–S59) with some extent of catalyst aggregation indicated (Table S9 and Figures S60–S63). It is hypothesized that aggregation of this species causes it to sit below the straight line fit that would describe the left side of the “volcano”, i.e., a linear fit from the peak at Zn­(Oct)_2_ (for cyclic carbonates) or Sn­(Oct)_2_ (for lactones) to the lowest performing Zr­(Oct)_4_ catalyst on the left side of the plot.

The linear free energy relationship linking polymerization rate coefficient, *k*_obs_, to active site acidity is fully consistent with the rate law, the coordination–insertion polymerization mechanism, and its proposed rate-determining step. In the catalysis, the metal center fulfills two different roles as it must both activate the metal-coordinated carbonyl group and provide the metal-alkoxide nucleophile to attack the carbonyl functionality (Figure 1c). The linear free energy relationship is consistent with low activity values for metals at either end of the Lewis acidity scale (Figure 8). For metals with high Lewis acidity (low p*K*_h_), the metal-alkoxide chain end nucleophile reactivity is reduced, e.g., rationalizing the low activity shown by the Bi­(III) and Zr­(IV) catalysts. At the other extreme, the Lewis acidity is sufficiently low (high p*K*_h_) that the monomer activation is reduced and rates are lower, e.g., the lower activity shown by the Mg­(II) catalyst. In the middle of the Lewis acidity series, Zn­(II) and Sn­(II) catalysts exhibit the most effective balance of monomer activation and metal-alkoxide lability, with the highest rates also depending on the nature of the monomer, i.e., cyclic carbonate or lactone.

Next, it is important to understand the difference in peak reactivity between the lactones and the cyclic carbonates. The trend is particularly interesting, as it points to an important relationship between the reactivity of the monomer carbonyl group and the metal active site Lewis acidity. We proposed that the cyclic carbonates, which have an additional oxygen atom, show reduced carbonyl electrophilicity compared to the lactones and thus may be better matched to metal catalysts with a slightly lower relative Lewis acidity (i.e., Zn­(II) in this series).

To investigate the electrophilicity of the monomer carbonyl group, the monomers were characterized using various spectroscopy techniques to establish any trends in their resonance frequencies. Accordingly, IR spectroscopy was used to compare the cyclic ester and -carbonate C=O stretching frequencies, and ^13^C NMR spectroscopy was used to compare the chemical shifts of the carbonyl C atoms (Figures S64–S77). In the IR spectra, comparing the ester and carbonate monomers reveals distinct differences, with valerolactone (VL) showing a C=O resonance at 1734 cm^–1^, and trimethylene carbonate (TMC) showing its C=O resonance at a lower energy value of 1716 cm^–1^ (Figure 9a). In the ^13^C­{^1^H} NMR spectra, the quaternary carbon (C=O) chemical shift indicates its relative electrophilicity, with higher chemical shifts indicating a more electrophilic monomer (Figures S65–S77). Once again, comparing the two 6-membered ring monomers shows that TMC has δ = 148.6 ppm while VL shows its carbonyl signal at δ = 171.4 ppm (Figure 9b, Figures S66 and S68). Both the IR and NMR spectroscopy data indicate that the lactone is more electrophilic than the cyclic carbonate.

**Figure 9:**
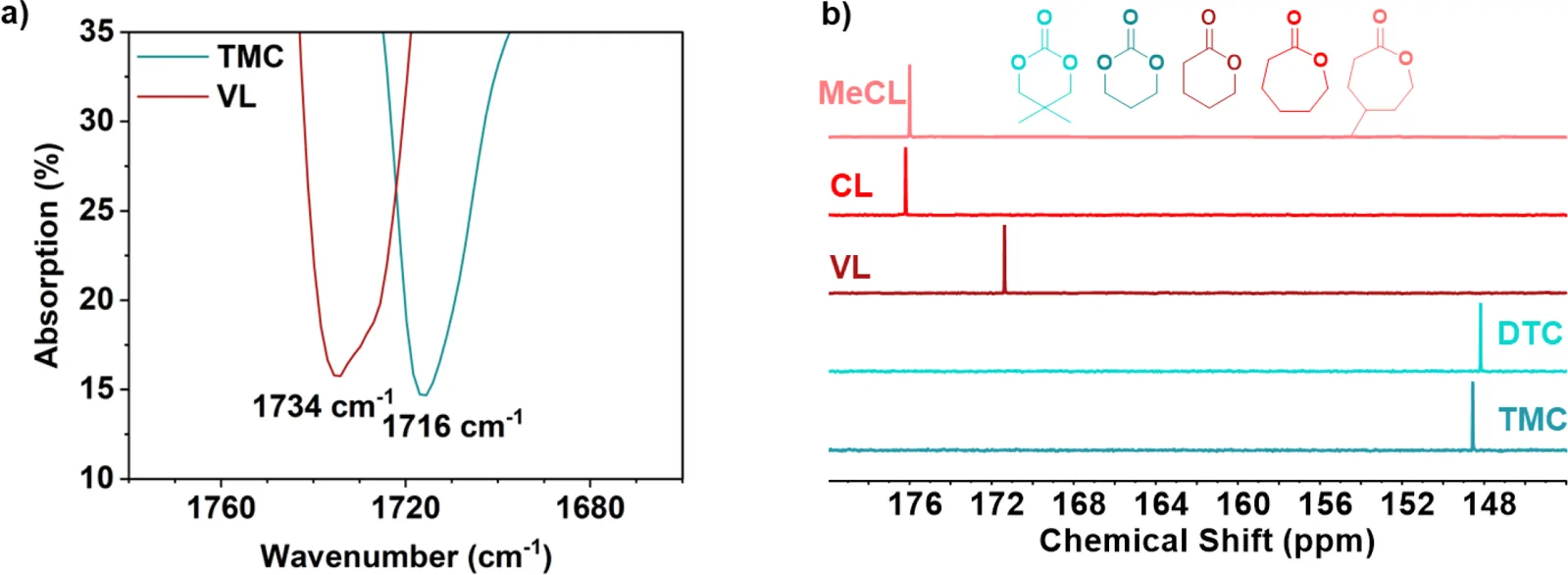
Spectroscopy to inform upon monomer electrophilicity. (a) A selected region of the IR spectrum, showing the differences in C=O resonances for cyclic carbonate (TMC) and lactone (VL), where a lower frequency vibration corresponds to a less electrophilic carbon (see Figure S64 for the full spectra). (b) Selected regions of the ^13^C­{^1^H} NMR spectra for the five monomers, showing the carbonyl resonance, with peaks at higher chemical shift corresponding to more electrophilic carbons (Figures S65–S77). For clarity, a zoomed-in spectrum on the carbonyl shifts for MeCL and CL can be found in Figure S77.

The monomer characterization data can be related to the relative polymerization rates and linear free energy relationships. The cyclic carbonates have lower electrophilicity, and thus, for these monomers, the rate-determining step appears to be more influenced by the relative catalyst metal-alkoxide nucleophilicity. Hence, the weaker Lewis acid Zn­(II) with a nucleophilic alkoxide is favored for these polymerizations. In contrast, the lactones are more electrophilic and polymerization rates are slightly faster using a more Lewis acidic Sn­(II) active site, perhaps because monomer coordination exerts a more significant influence on the rate-determining step.

Examining the relative rates for the 7-membered lactone (CL) shows a bigger separation in the rates for the Sn­(II) and Zn­(II) catalysts, with the stronger Lewis acid (lower p*K*_h_) being more clearly favored, compared with the 6-membered lactone (VL) (Figure 6). Consistent with the rate observation, the ^13^C­{^1^H} NMR resonance for the carbonyl carbon in CL is 176.2 ppm, which is higher than the corresponding signal for VL (δ = 171.4 ppm, Figure S68). These findings suggest that CL has a more electrophilic carbonyl group than VL (Figure 9b and Figures S68 and S70). Consequently, CL may be expected to require a stronger Lewis acid to activate it to ring-opening, rationalizing the larger relative difference in rates between Sn­(Oct)_2_ and Zn­(Oct)_2_, compared with VL, where the rates are closer (Figures 6 and 8).

The transition state barriers for the catalysts were examined using Eyring analyses conducted using VL ROP (Figure S78). In all cases, polymerizations involve a negative transition state entropy change, Δ*S*_pol_^‡^, which is interpreted by an associative rate-determining step involving the monomer coordination at the catalyst, which is expected to be entropically disfavored. In all polymerizations, the transition state enthalpy change, Δ*H*_pol_^‡^, is positive, consistent with energy being required to break the metal-alkoxide bond during monomer ring-opening. Plotting the transition state barriers for VL ROP vs the different catalysts’ metal Lewis acidities (p*K*_h_ values) shows that the enthalpy decreases with increasing metal p*K*_h_ but the entropy increases with increasing metal p*K*_h_ (Figure 10). The most Lewis acidic metals, e.g., Zr­(IV) and Bi­(III), are expected to show stronger coordination to the carbonyl group in the monomer, increasing the transition state preorganization and reducing the entropy barrier. On the other hand, the least Lewis acidic metals, e.g., Mg­(II) and Zn­(II), should show more labile alkoxide nucleophiles and hence show the lowest transition state enthalpy. Hence, the fastest catalysts are those that most effectively minimize both the enthalpy and entropy for polymerization. Interestingly, the crossover between the linear fits to enthalpy and entropy (vs Lewis acidity) sits between Sn­(II) and Zn­(II), suggesting that the optimum catalyst would have properties that make it slightly more acidic than Zn­(Oct)_2_ and less acidic than Sn­(Oct)_2_. This finding provides a clear direction for future catalyst optimization and suggests that opposite ligand design strategies should be applied to the two metals.

**Figure 10:**
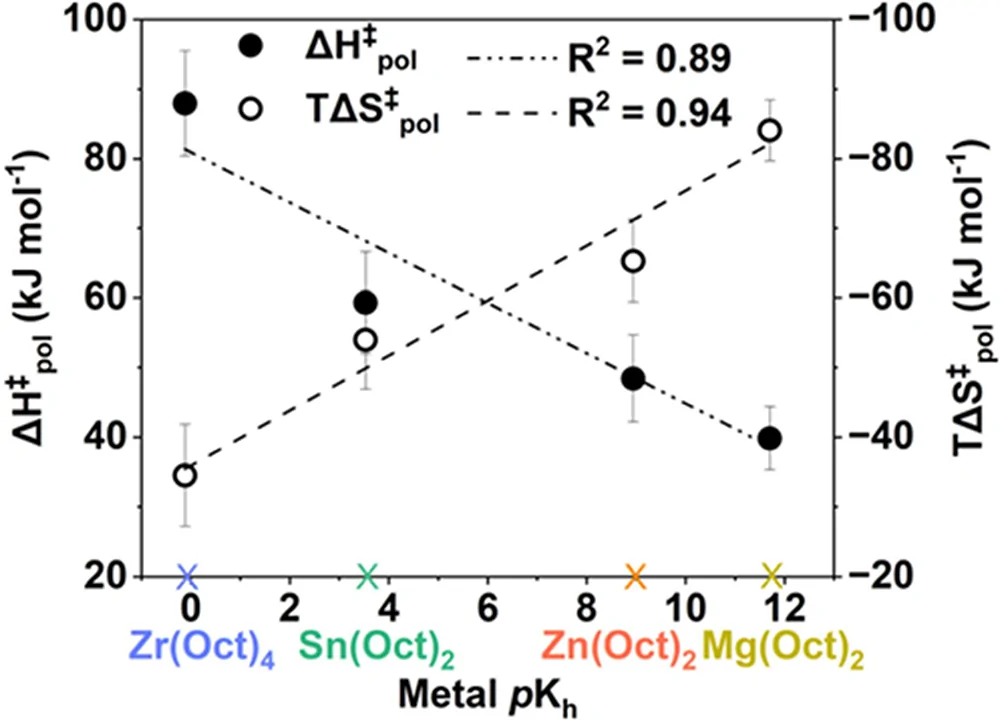
Plot of transition state enthalpies and entropies vs metal p*K*_h_ for polymerization of VL to PVL, with each M­(Oct)_n_ catalyst. Bi­(Oct)_3_ has been omitted from the plot, as its kinetic parameters are outliers (Figures S60–S63 and Table S9), and MALDI analyses of end groups suggest a different mechanism for this catalyst (Figures S47–S59).

Additionally, Eyring analyses were conducted for the two fastest M­(Oct)_n_ catalysts for CL ROP: Sn­(Oct)_2_ and Zn­(Oct)_2_ (Figure 6 and Table S2). These were investigated following the observation of a greater difference in polymerization rate using Sn­(Oct)_2_ in comparison to Zn­(Oct)_2_ for CL than VL, i.e., CL has an even greater preference for the Sn­(II) catalyst than VL (Figure 6), which should indicate a greater difference in polymerization transition state barriers. The Eyring analyses indicate Δ*H*_pol_^‡^ = 95.7 ± 7.0 kJ mol^–1^ for Sn­(Oct)_2_ catalyzed CL ROP, but a lower Δ*H*_pol_^‡^ = 66.2 ± 5.0 kJ mol^–1^ for the equivalent Zn­(Oct)_2_ catalysis, consistent with its less Lewis acidic and more labile metal-alkoxide nucleophile (Figure S79 and Table S2). Further, the determined Δ*S*_pol_^‡^ = −30.3 ± 16.2 J mol^–1^K^–1^ for Sn­(Oct)_2_-catalyzed CL ROP has a lower entropy barrier than the Δ*S*_pol_^‡^ = −114.9 ± 11.0 J mol^–1^K^–1^ for the equivalent Zn­(Oct)_2_ catalysis (Figure S79 and Table S2). This result is in line with the hypothesized greater transition state preorganization possible for the more Lewis acidic metals. At the reaction temperatures, the difference in entropy penalty (*T*Δ*S*_pol_^‡^), between Zn­(Oct)_2_ and Sn­(Oct)_2_ catalysts, is greater than the difference in their enthalpy penalty (*T*Δ*S*_pol_^‡^ – Δ*H*_pol_^‡^). This indicates that the improved performance of the Sn­(Oct)_2_ catalyst, in comparison to the Zn­(Oct)_2_ catalyst, for CL ROP, is primarily driven by a reduced entropy penalty.

### Utilizing Linear Free Energy Relationships to Improve the Catalysis

Having established a series of generally applicable linear free energy relationships correlating rates and catalyst metal Lewis acidity, we were interested to understand its predictive power for selecting the most appropriate metal carboxylate catalyst for another monomer in ring-opening polymerization. The catalysis for l-lactide (l-LA) polymerization is a priority since it is the commercial route to make bioderived poly­(l-lactide);^9,10^ as outlined in the introduction, there is still contradictory literature regarding the most active metals for its catalysis.^39–42^ To evaluate the monomer properties and, in particular, its carbonyl electrophilicity, l-LA was characterized using ^13^C­{^1^H} NMR and IR spectroscopy. It has carbonyl resonances at 1743 cm^–1^ (IR, Figure S64) and δ = 167.7 ppm (^13^C NMR, Figure S76). As such, its carbonyl group electrophilicity, and expected reactivity, is closer to the values determined for VL than for TMC. Therefore, it is predicted to show the highest polymerization rates using the slightly more Lewis acidic Sn­(Oct)_2_ compared with Zn­(Oct)_2_. It is worth noting that, in contrast to the monomers previously studied in this work, the l-LA propagates via a secondary alkoxide, and hence, rates of polymerization should be somewhat slower than monomers enchained via primary alkoxides. The l-LA ROP rate coefficients were measured, under identical conditions, for both catalysts: for Zn­(Oct)_2_, *k*_obs_ = 0.98 ± 0.07 × 10^–3^ s^–1^, while for Sn­(Oct)_2_, *k*_obs_ = 11.5 ± 0.12 × 10^–3^ s^–1^ (note that due to lower monomer solubility, conditions for these polymerizations are 1:10:250 M­(Oct)_2_:BnOH:l-LA and [l-LA] = 1.56 M in 1,2-dimethoxybenzene, Table S10, Figures S80 and S81). This result underscores the utility of the linear free energy relationship, combined with the monomer spectroscopy characterizations, in predicting the most effective monomer-metal pairings. Importantly, it allows for quantification of metal Lewis acidity influences in the coordination–insertion mechanism.

As proof of concept in using these relationships to increase catalytic activity, the performance of the Mg­(Oct)_2_ catalyst was targeted since it is earth-abundant, light, and inexpensive. In all the ring-opening polymerizations, Mg­(Oct)_2_ showed rather low rates and was not competitive with the best catalysts. To improve its performance, the nature of the active site needs to be considered. Previously, Penczek and co-workers proposed that Sn­(Oct)_2_/ROH catalysts operate by (Oct)­Sn­(OR) active species.^17^ To understand its relevance to other M­(II) catalysts, MALDI-TOF analyses were conducted using a reaction aliquot removed from a polymerization of VL conducted with Zn­(Oct)_2_/BnOH. The data reveal a series of chains consistent with the formula units H­[O­(O)­C­(CH_2_)_4_]_n_OZn­(Oct), i.e., chains with (Oct)­Zn­(OR) where R is the PVL chain (Figures S44–S46). These data strongly support the same catalyst active site speciation proposed by Penczek and co-workers, indicating that one of the original carboxylate (Oct) ligands remains coordinated at the metal throughout the polymerization. One consequence of such an active site chemistry is that modifying the carboxylate ligand might serve to tune the relative metal Lewis acidity, and, hence, rates. We targeted a magnesium benzoate propagating intermediate since we hypothesized that a more electron-withdrawing aromatic carboxylate ligand (p*K*_a_ benzoic acid = 4.20 vs 2-ethylhexanoic acid = 4.70) might increase the relative Lewis acidity at the Mg­(II) center and, hence, increase polymerization rates. To test the hypothesis, VL polymerization was conducted using Mg­(Bu)_2_:BzOH:BnOH:VL 1:2:10:1000, where BzOH is benzoic acid (with [VL] = 6.25 M in 1,2-dimethoxybenzene). The conditions are selected to form the precatalyst, Mg­(OBz)_2_ *in situ* by reaction of the organomagnesium reagent and benzoic acid (Table S11 and Figures S82–S84). To make the precatalyst, Mg­(Bu)_2_ and BzOH were stirred together for 2 h to preform the Mg­(OBz)_2_ catalytic species (Figures S82 and S83). During this period, gas release was observed, then BnOH, VL, and 1,2-dimethoxybenzene solvent were added to give the typical conditions for catalysis. To ensure polymerizations were conducted under identical conditions, the Mg­(Oct)_2_ precatalyst was also generated *in situ* by reaction of Mg­(Bu)_2_ and 2-ethylhexanoic acid (OctH) (Table S11 and Figure S84). Comparing the relative rates for VL ROP between the catalysts shows that Mg­(OBz)_2_ has a significantly higher polymerization rate coefficient, *k*_obs_ = 2.02 ± 0.19 × 10^–4^ s^–1^, compared with Mg­(Oct)_2_, where *k*_obs_ = 0.6 ± 0.07 × 10^–4^ s^–1^ (Figure 11). Furthermore, as a control for the effects of steric differences in the ligands on polymerization rate, 4-trifluoromethyl substituted benzoate was tested as a ligand for the Mg­(II). This shows a similar steric profile to the unsubstituted benzoate, but is an even more electron-withdrawing ligand (p*K*_a_ trifluoromethyl benzoic acid = 3.70 vs p*K*_a_ benzoic acid = 4.20). Indeed, this revealed a further increased rate constant for polymerization, supporting the hypothesis that the carboxylate ligands are modulating the active site Lewis acidity so as to improve its performance as a catalyst (Figures S82–S84 and Table S11).

**Figure 11:**
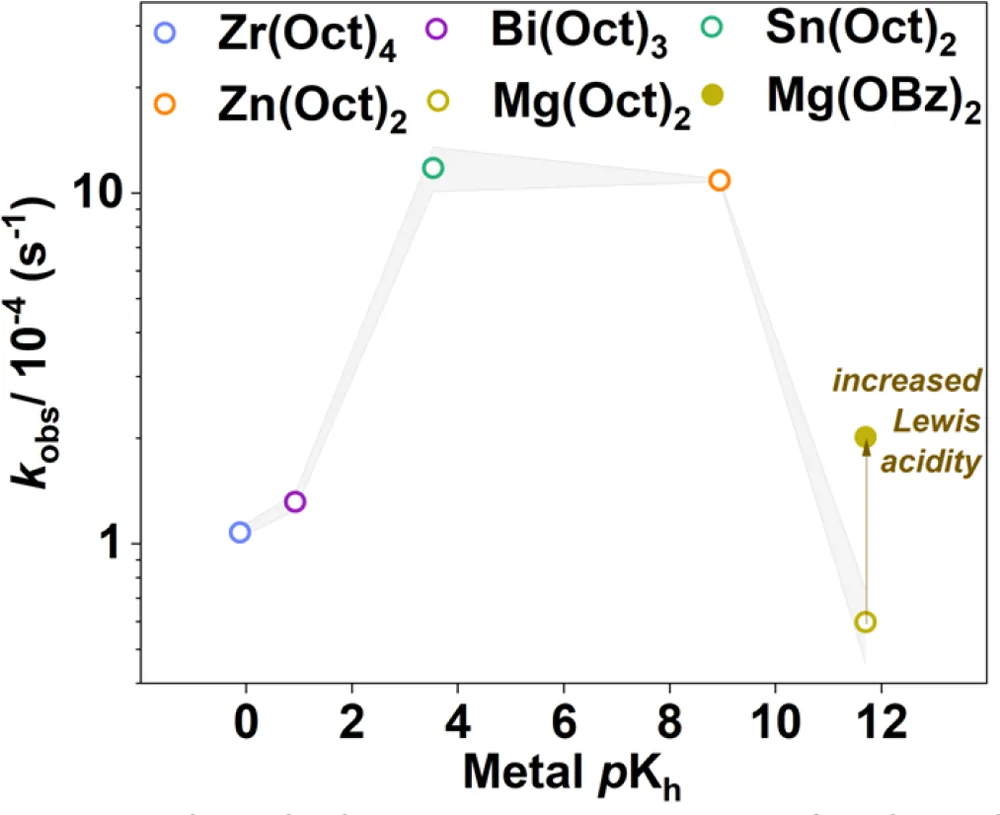
Plots of polymerization rate constants, *k*_obs_, for each catalyst tested for VL polymerization, highlighting the Mg­(II) benzoate species as improved in comparison to the Mg­(II) 2-ethylhexanoate on the original LFER. Polymerization conditions: 1:10:1000 M­(OR)_n_:BnOH:monomer, [monomer] = 6.25 M in 1,2-dimethoxybenzene, 160 °C.

These findings highlight the potential to use the linear free energy relationships to target higher activity catalysts, with appropriate consideration of the ancillary ligand electronic influences at the metal active site.^46^ The relationship also identifies that the strategies for these ligand modifications should be metal-dependent (with the peak activity in the volcano plot being very important): for metals showing Lewis acidity values below the value for Zn­(II), ligands that are more electron-withdrawing than octoate (Oct) should be prioritized. On the other hand, for metals showing Lewis acidity values greater than the value for Sn­(II), ligands that are more electron-donating than octoate (Oct) should be prioritized. This difference in ancillary ligand electronic features is important since it may help to explain some of the apparent ligand-catalyst activity contradictions in the literature. It also clearly rationalizes why different ligand features are desirable for particular metals and provides a rationale for the ligand design. It is important to note, however, that ligand sterics may also influence catalytic performance,^70^ so ligand modifications will always require a careful balance of optimal acidity and structure for efficient monomer activation and ring opening.

Finally, it is important to understand whether the quantified structure–activity relationships determined in concentrated monomer solutions (in 1,2-dimethoxybenzene) also apply to polymerizations conducted in neat monomer melts. To explore further, VL ROP was investigated using all five M­(Oct)_n_ catalysts under high-temperature, neat conditions. As expected, all of the polymerizations showed slightly higher rates with neat monomer (Figure 12, Figure S85, and Table S12). Importantly, though, the same structure–activity relationship and linear free energy relationship are observed, with the peak activity being, once again, observed for Sn­(Oct)_2_. This last finding is potentially important since it signals that small-scale, automated rate measurements, using the calorimetry methodology presented in this work, may serve as predictors for catalyst performances under industrially relevant conditions.

**Figure 12:**
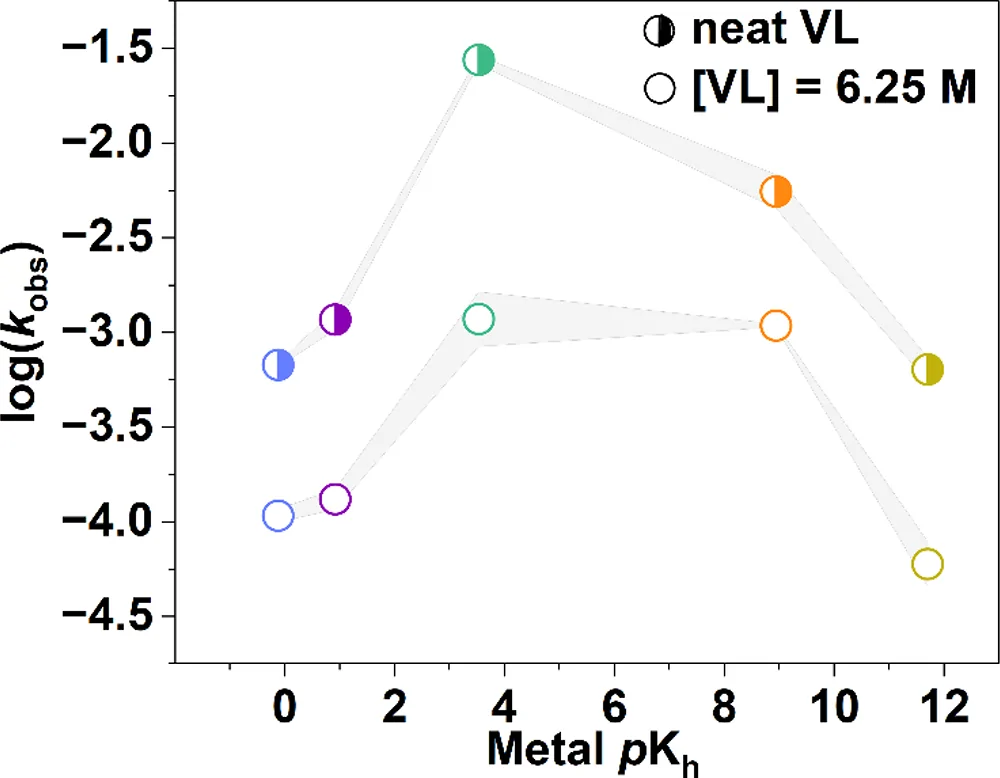
Plot of log­(*k*_obs_) vs metal p*K*_h_ for the polymerizations conducted in neat VL (half-filled circles), with the previous LFER for [VL] = 6.25 M in 1,2-dimethoxybenzene indicated for comparison (open circles), showing that the LFER applies to polymerizations conducted in both neat monomer and concentrated solutions.

## Conclusions

A series of metal octoate catalysts were compared, under low catalyst loadings, high monomer concentrations, and high temperatures, for the ring-opening polymerization of a series of cyclic esters and carbonates. A new and generally applicable quantified linear free energy relationship (LFER) exists between catalytic activity and metal active site Lewis acidity. For the cyclic esters, Sn­(Oct)_2_ is the best precatalyst, whereas for the cyclic carbonates, Zn­(Oct)_2_ is the fastest. The relative monomer carbonyl group electrophilicity was measured using IR and ^13^C­{^1^H} NMR spectroscopies, and results were used to rationalize the most effective monomer-metal partnerships. The quantified structure-performance relationship was tested using a new monomer, l-lactide, to correctly predict that Sn­(II) would be the faster catalyst; the prediction was experimentally validated. The relationships were also used to rationally improve the performance of a Mg­(II) carboxylate precatalyst, substituting the octoate for the more electron-withdrawing benzoate, so as to increase rates. Finally, the linear free energy relationship was shown to apply to high-temperature, neat monomer conditions that are relevant to industrial polymerizations. These small-scale, fast, reliable, and quantified structure–activity relationships should accelerate the discovery of the most efficient catalysts and conditions for the production of sustainable polymers.

## Supplementary Material



## Data Availability

Data for the information contained in the paper and supporting information are open access and available via the Oxford University Research Archive, ORA, at https://ora.ox.ac.uk/objects/uuid:27753da3-e5eb-4e0c-9ae9-71b3550a8787.

## Notes

Materials, instrumentation, methods, NMR spectroscopy data, IR spectroscopy data, polymerization rate data, DSC data, and MALDI-TOF mass spectrometry data (PDF)
