# Supplementary material for: Matching Monomers to Metals: Quantified Linear Free Energy Relationships in Metal-Catalyzed Ring-Opening Polymerizations
Source: J Am Chem Soc. 2026 Aug 14;148(33):35484–97. doi: 10.1021/jacs.6c06048 (PMC13523706; doi:10.1021/jacs.6c06048)
Supplement: Supplementary file 1 [file ja6c06048_si_001.pdf]

Supporting Information

Matching Monomers to Metals: Quantified Linear Free Energy Relationships in Metal-Catalyzed Ring-Opening Polymerizations

Rosie Thorogood, Thomas M. McGuire, Charlotte K. Williams*

Department of Chemistry, Chemistry Research Laboratory, 12 Mansfield Road, University of Oxford, Oxford, OX1 3TA, United Kingdom

*charlotte.williams@chem.ox.ac.uk

Table of Contents

Supporting Information 1

1. *Materials* 7
2. *Methods*.. 8
   Procedure for DSC Kinetic Experiments[3]..8
   Procedure for $^{31}P\{^1H\}$ NMR Studies to Probe Metal Catalyst Lewis Acidity[4] 8
3. *Additional Information* 9
   Fig. S1 Stacked $^{31}P\{^1H\}$ NMR spectra (400 MHz, $CDCl_3$, 298 K) for the Lewis acidity ordering of the $M(Oct)_n$ species coordinated to a trioctylphosphine oxide probe, relative to a phosphoric acid standard (contained in a capillary) set to 0 ppm....9
   Fig. S2 Plot of $\Delta^{31}P$ NMR δ for a trioctylphosphine oxide coordinated $M(Oct)_n$ species relative to the trioctylphosphine oxide alone *vs*. the *p*K_h values for each metal. A lower than expected shift for $Bi(Oct)_3$ is proposed to occur due to aggregation (Table S9, Fig. S60-S63) which reduces propensity for the catalyst to coordinate. 10
   Fig. S3 Isothermal polymerization kinetic experiments, conducted on a DSC instrument, for each 10 °C temperature increment from 160 °C to 200 °C (conducted in triplicate) for the polymerization of VL with the $Zn(Oct)_2$ catalyst. The stacked plot shows one representative dataset at each temperature. For clarity, every 600th data point from the datasets is shown. Polymerization conditions: 1:10:1000 $Zn(Oct)_2$:BnOH:VL, [VL] = 6.25 M. In each case $[PVL]_t = 1 - [VL]_t$........................................11
   Fig. S4 Isothermal polymerization kinetic experiments, conducted on a DSC instrument, for each 10 °C temperature increment from 160 °C to 200 °C (conducted in triplicate) for the polymerization of CL with the Zn(Oct)2 catalyst. The stacked plot shows one representative dataset at each temperature. For clarity, every 600th data point from the datasets is shown. Polymerization conditions: 1:10:1000 Zn(Oct)2:BnOH:CL, [CL] = 6.25 M. In each case [PCL]t = 1 - [CL]t. 12
   Fig. S5 Isothermal polymerization kinetic experiments, conducted on a DSC instrument, for each 10 °C temperature increment from 160 °C to 200 °C (conducted in triplicate) for the polymerization of MeCL with the $Zn(Oct)_2$ catalyst. The stacked plot shows one representative dataset at each temperature. For clarity, every 600th data point from the datasets is shown. Polymerization conditions: 1:10:1000 $Zn(Oct)_2$:BnOH:MeCL, [MeCL] = 6.25 M. In each case $[PMCL]_t = 1 - [MeCL]_t$................................13
   Fig. S6 Isothermal polymerization kinetic experiments, conducted on a DSC instrument, for each 10 °C temperature increment from 140 °C to 180 °C (conducted in triplicate) for the polymerization of TMC with the $Zn(Oct)_2$ catalyst. The stacked plot shows one representative dataset at each temperature. For

clarity, every 600th data point from the datasets is shown. Polymerization conditions: 1:10:1000 $Zn(Oct)_2$:BnOH:TMC, [TMC] = 6.25 M. In each case $[PTMC]_t = 1 - [TMC]_t$. 14
Fig. S7 Isothermal polymerization kinetic experiments, conducted on a DSC instrument, for each 10 °C temperature increment from 160 °C to 200 °C (conducted in triplicate) for the polymerization of DTC with the $Zn(Oct)_2$ catalyst. The stacked plot shows one representative dataset at each temperature. For clarity, every 600th data point from the datasets is shown. Polymerization conditions: 1:10:1000 $Zn(Oct)_2$:BnOH:DTC, [DTC] = 6.25 M. In each case $[PDTC]_t = 1 - [DTC]_t$. 15
Fig. S8 Isothermal polymerization kinetic experiments, conducted on a DSC instrument, for each 10 °C temperature increment from 160 °C to 200 °C (conducted in triplicate) for the polymerization of VL with the $Sn(Oct)_2$ catalyst. The stacked plot shows one representative dataset at each temperature. For clarity, every 600th data point from the datasets is shown. Polymerization conditions: 1:10:1000 $Sn(Oct)_2$:BnOH:VL, [VL] = 6.25 M. In each case $[PVL]_t = 1 - [VL]_t$. 16
Fig. S9 Isothermal polymerization kinetic experiments, conducted on a DSC instrument, for each 10 °C temperature increment from 160 °C to 200 °C (conducted in triplicate) for the polymerization of VL with the $Bi(Oct)_3$ catalyst. The stacked plot shows one representative dataset at each temperature. For clarity, every 1000th data point from the datasets is shown. Polymerization conditions: 1:10:1000 $Bi(Oct)_3$:BnOH:VL, [VL] = 6.25 M. In each case $[PVL]_t = 1 - [VL]_t$. 17
Fig. S10 Isothermal polymerization kinetic experiments, conducted on a DSC instrument, for each 10 °C temperature increment from 160 °C to 200 °C (conducted in triplicate) for the polymerization of VL with the $Zr(Oct)_4$ catalyst. The stacked plot shows one representative dataset at each temperature. For clarity, every 6000th data point from the datasets is shown. Polymerization conditions: 1:10:1000 $Zr(Oct)_4$:BnOH:VL, [VL] = 6.25 M. In each case $[PVL]_t = 1 - [VL]_t$. 18
Fig. S11 Isothermal polymerization kinetic experiments, conducted on a DSC instrument, for each 10 °C temperature increment from 160 °C to 200 °C (conducted in triplicate) for the polymerization of VL with the $Mg(Oct)_2$ catalyst. The stacked plot shows one representative dataset at each temperature. For clarity, every 8000th data point from the datasets is shown. Polymerization conditions: 1:10:1000 $Mg(Oct)_2$:BnOH:VL, [VL] = 6.25 M. In each case $[PVL]_t = 1 - [VL]_t$. 19
Fig. S12 Isothermal polymerization kinetic experiments, conducted on a DSC instrument, for each 10 °C temperature increment from 140 °C to 180 °C (conducted in triplicate) for the polymerization of CL with the $Sn(Oct)_2$ catalyst. The stacked plot shows one representative dataset at each temperature. For clarity, every nth data point from the datasets is shown (n = 600, 400, 400, 200, and 100 for 140 °C, 150 °C, 160 °C, 170 °C and 180 °C respectively). Polymerization conditions: 1:10:1000 $Sn(Oct)_2$:BnOH:CL, [CL] = 6.25 M. In each case $[PCL]_t = 1 - [CL]_t$. 20
Fig. S13 Isothermal polymerization kinetic experiments, conducted on a DSC instrument, at 160 °C for the polymerization of CL with the $Bi(Oct)_3$ catalyst (plot in purple, top LHS), $Zr(Oct)_4$ catalyst (plot in blue, top RHS), and $Mg(Oct)_2$ catalyst (plot in yellow, bottom RHS). For clarity, every nth data point from the datasets is shown (n= 1000, 6000, 8000 for Bi(III), Zr(IV), Mg(II) respectively). Polymerization conditions: 1:10:1000 $M(Oct)_n$:BnOH:CL, [CL] = 6.25 M. In each case $[PCL]_t = 1 - [CL]_t$. 21
Fig. S14 Isothermal polymerization kinetic experiments, conducted on a DSC instrument, at 160 °C for the polymerization of MeCL with the $Sn(Oct)_2$ catalyst (plot in green, top LHS), $Bi(Oct)_3$ catalyst (plot in purple, top RHS), $Zr(Oct)_4$ catalyst (plot in blue, bottom LHS), and $Mg(Oct)_2$ catalyst (plot in yellow, bottom RHS). For clarity, every nth data point from the datasets is shown (n= 600, 1000, 6000, 8000 for Sn(II), Bi(III), Zr(IV), Mg(II) respectively). Polymerization conditions: 1:10:1000 $M(Oct)_n$:BnOH:MeCL, [MeCL] = 6.25 M. In each case $[PMCL]_t = 1 - [MeCL]_t$. 22
Fig. S15 Isothermal polymerization kinetic experiments, conducted on a DSC instrument, at 160 °C for the polymerization of TMC with the $Sn(Oct)_2$ catalyst (plot in green, top LHS), $Bi(Oct)_3$ catalyst (plot in purple, top RHS), $Zr(Oct)_4$ catalyst (plot in blue, bottom LHS), and $Mg(Oct)_2$ catalyst (plot in yellow, bottom RHS). For clarity, every n^{th} data point from the datasets is shown (n = 600, 1000, 6000, 8000 for Sn(II), Bi(III), Zr(IV), Mg(II) respectively). For the polymerization with $Bi(Oct)_3$ k_{obs} was extracted from a sigmoidal instead of exponential fit, of the form: $[PTMC] = A(1-\exp(-k_{obs}t))^s$, where s is the 'sigmoidal factor' (=3.3-3.5). Polymerization conditions: 1:10:1000 $M(Oct)_n$:BnOH:TMC, [TMC] = 6.25 M. In each case $[PTMC]_t = 1 - [TMC]_t$. 23

Fig. S16 Isothermal polymerization kinetic experiments, conducted on a DSC instrument, at 160 °C for the polymerization of DTC with the $Sn(Oct)_2$ catalyst (plot in green, LHS), and $Mg(Oct)_2$ catalyst (plot in yellow, RHS). For clarity, every nth data point from the datasets is shown (n = 600 and 8000 for Sn(II) and Mg(II) respectively). Polymerization conditions: 1:10:1000 $M(Oct)_n$:BnOH:DTC, [DTC] = 6.25 M. In each case $[PDTC]_t = 1 - [DTC]_t$. 24
Fig. S17 Representative ^1H NMR spectrum (400 MHz, $CDCl_3$, 298 K) for calculation of conversion of VL to PVL. Polymerization conditions: 1:10:1000 $Zn(Oct)_2$:BnOH:VL, [VL] = 6.25 M, at 200 °C. 25
Fig. S18 Representative ^1H NMR spectrum (400 MHz, $CDCl_3$, 298 K) for calculation of conversion of CL to PCL. Polymerization conditions: 1:10:1000 $Zn(Oct)_2$:BnOH:CL, [CL] = 6.25 M, at 200 °C. 25
Fig. S19 Representative ^1H NMR spectrum (400 MHz, $CDCl_3$, 298 K) for calculation of conversion of MeCL to PMCL. Polymerization conditions: 1:10:1000 $Zn(Oct)_2$:BnOH:MeCL, [MeCL] = 6.25 M, at 200 °C. 26
Fig. S20 Representative ^1H NMR spectrum (400 MHz, $CDCl_3$, 298 K) for calculation of conversion of TMC to PTMC. Polymerization conditions: 1:10:1000 $Zn(Oct)_2$:BnOH:TMC, [TMC] = 6.25 M, at 180 °C. 26
Fig. S21 Representative ^1H NMR spectrum (400 MHz, $CDCl_3$, 298 K) for calculation of conversion of DTC to PDTC. Polymerization conditions: 1:10:1000 $Zn(Oct)_2$:BnOH:DTC, [DTC] = 6.25 M, at 200 °C. 27
Table S1 Polymerization data for polymerization of VL. Polymerization conditions: 1:10:1000 $M(Oct)_n$:BnOH:VL, [VL] = 6.25 M. 28
Table S2 Polymerization data for polymerization of CL. Polymerization conditions: 1:10:1000 $M(Oct)_n$:BnOH:CL, [CL] = 6.25 M. 30
Table S3 Polymerization data for polymerization of MeCL. Polymerization conditions: 1:10:1000 $M(Oct)_n$:BnOH:MeCL, [MeCL] = 6.25 M. 31
Table S4 Polymerization data for polymerization of TMC. Polymerization conditions: 1:10:1000 $M(Oct)_n$:BnOH:TMC, [TMC] = 6.25 M. 32
Table S5 Polymerization data for polymerization of DTC. Polymerization conditions: 1:10:1000 $M(Oct)_n$:BnOH:DTC, [DTC] = 6.25 M. 33
Table S6 k_{obs} determined at incrementally increasing catalyst loadings for the polymerization of TMC using the $Zn(Oct)_2$ catalyst and BnOH chain-transfer agent at 100 °C. 34
Fig. S22 Plots of [PTMC] *vs*. time for isotherms conducted at 100 °C. k_{obs} is taken from the exponential fitting of each line ($y = A - Be^{-kobsx}$), with the average k_{obs} taken as the mean of the three repeats and error determined from the standard deviation of the mean of the three repeats. Polymerization conditions: 20:200:1000 $Zn(Oct)_2$:BnOH:TMC. 34
Fig. S23 Plot of k_{obs} *vs*. [$Zn(Oct)_2$] for isotherms conducted at 100 °C with polymerization conditions: x:10x:1000 $Zn(Oct)_2$:BnOH:TMC where x= 1, 10, 20, and 25, [TMC] = 6.25 M. 35
Fig. S24 Plot of ln(k_{obs}) *vs*. ln([$Zn(Oct)_2$]) for isotherms conducted at 100 °C with polymerization conditions: x:10x:1000 $Zn(Oct)_2$:BnOH:TMC where x= 1, 10, 20, and 25, [TMC] = 6.25 M. 35
Fig. S25 Bar chart of polymerization rate constant, k_{obs}, normalized for the number of octoate chains per metal center (n), for each monomer tested with each $M(Oct)_n$ catalyst. Polymerization conditions: 1:10:1000 $M(Oct)_n$:BnOH:monomer, [monomer] = 6.25 M in 1,2-dimethoxybenzene, 160 ^oC. Catalysts $Bi(Oct)_3$ and $Zr(Oct)_4$ showed negligible activity for DTC polymerization under these conditions. 36
Table S7 Polymerization data for polymerization of VL. Polymerization conditions: 1:1:1000 $M(Oct)_n$:BnOH:VL, [VL] = 6.25 M, 160 ^oC. 37
Fig. S26 Isothermal polymerization kinetic experiments, conducted on a DSC instrument, at 160 ^oC (conducted in triplicate) for the polymerization of VL with the $Zn(Oct)_2$ catalyst (top row, LHS), $Sn(Oct)_2$ catalyst (top row, RHS), and $Bi(Oct)_3$ catalyst (bottom row, LHS). For clarity, every nth data point from the datasets is shown, where n = 600, 1000 and 3000 for Zn(II), Sn(II) and Bi(III) respectively. Polymerization conditions: 1:1:1000 $M(Oct)_n$:BnOH:VL, [VL] = 6.25 M. In each case $[PVL]_t = 1 – [VL]_t$. ..38
Fig. S27 Plot of k_{obs} using 10 eq BnOH and TOF using 1 eq BnOH *vs*. metal pK_h, indicating the LFER is upheld under both sets of polymerization conditions. Polymerization conditions: 1:x:1000 $M(Oct)_n$:BnOH:VL, x = 1 or 10, [VL] = 6.25 M. In each case $[PVL]_t = 1 – [VL]_t$. 39
Table S8 k_{obs} determined at systematically varied catalyst loadings for the polymerization of TMC using the $Mg(Oct)_2$ catalyst and BnOH chain-transfer agent at 160 °C. 40

Fig. S28 Plots of [PTMC] *vs*. time for isotherms conducted at 160 °C. k_{obs} is taken from the exponential fitting of each line ($y = A - Be^{-k_{obs}x}$), with the average k_{obs} taken as the mean of the three repeats and error determined from the standard deviation of the mean of the three repeats. Polymerization conditions: 2:20:1000 $Mg(Oct)_2$:BnOH:TMC. 40
Fig. S29 Plot of k_{obs} *vs*. [$Mg(Oct)_2$] for isotherms conducted at 160 °C with polymerization conditions: x:10x:1000 $Mg(Oct)_2$:BnOH:TMC where x= 0.25, 0.5, 1, and 2, [TMC] = 6.25 M. 41
Fig. S30 Plot of ln(k_{obs}) *vs*. ln([$Mg(Oct)_2$]) for isotherms conducted at 160 °C with polymerization conditions: x:10x:1000 $Mg(Oct)_2$:BnOH:TMC where x= 0.25, 0.5, 1, and 2, [TMC] = 6.25 M. 41
Fig. S31. DOSY NMR Spectrum of $Mg(Oct)_2$ catalyst in d-THF at 50 °C. Two diffusion coefficients are observed, for the d-THF solvent (log(D) = -4.65) and for the $Mg(Oct)_2$ catalyst (log(D) = -5.09). 42
Fig. S32. Plot showing k_{obs} *vs*. metal ionic radius (coordination number 6) for the polymerization of each monomer (VL, CL, MeCL, TMC and DTC) catalyzed by $M(Oct)_n$. Polymerization conditions: $M(Oct)_n$:BnOH:monomer, 1:10:1000, 160 °C, [monomer] = 6.25 M in 1,2-dimethoxybenzene. Ionic radii were taken from [7, 8]. 43
Fig. S33. Plot showing k_{obs} *vs*. metal oxophilicity for the polymerization of each monomer (VL, CL, MeCL, TMC and DTC) catalyzed by $M(Oct)_n$. Polymerization conditions: $M(Oct)_n$:BnOH:monomer, 1:10:1000, 160 °C, [monomer] = 6.25 M in 1,2-dimethoxybenzene. Oxophilicity values taken from [9]. 44
Fig. S34 MALDI-TOF Spectrum for PVL produced using $Zn(Oct)_2$ catalyst. The polymer (10 mg mL^{-1} in THF), and dithranol (10 mg mL^{-1} in THF) were premixed in a 1:25 ratio (v/v). Identified end group structures are given and these are analyzed in detail in the following figures (Fig. S35-S46). 45
Fig. S35 m/z *vs.* repeat unit plot for the first series of identified peaks from MALDI-TOF analysis of PVL produced using the $Zn(Oct)_2$ catalyst, where the slope gives the repeat unit and the intercept gives the end group molar mass. 46
Fig. S36 MALDI-TOF Spectrum for PVL produced using $Zn(Oct)_2$ catalyst, focusing on peaks identified as PVL with a benzyl alcohol end group (doped with Na^+). The polymer (10 mg mL^{-1} in THF), and dithranol (10 mg mL^{-1} in THF) were premixed in a 1:25 ratio (v/v). 46
Fig. S37 The experimental spectrum stacked with an isotope simulated spectrum for $C_5H_7O[C_5H_8O_2]_{30}H$, Na^+ m/z = 3134.64. 47
Fig. S38 m/z *vs.* repeat unit plot for the second series of identified peaks from MALDI-TOF analysis of PVL produced using the $Zn(Oct)_2$ catalyst, where the slope gives the repeat unit and the intercept gives the end group molar mass. 48
Fig. S39 MALDI-TOF Spectrum for PVL produced using $Zn(Oct)_2$ catalyst, focusing on peaks identified as PVL with a hydroxyl end group (doped with Na^+). The polymer (10 mg mL^{-1} in THF), and dithranol (10 mg mL^{-1} in THF) were premixed in a 1:25 ratio (v/v). 48
Fig. S40 The experimental spectrum stacked with an isotope simulated spectrum for $HO[C_5H_8O_2]_{18}H$, Na^+ m/z = 1843.11. 49
Fig. S41 m/z *vs.* repeat unit plot for the third series of identified peaks from MALDI-TOF analysis of PVL produced using the $Zn(Oct)_2$ catalyst, where the slope gives the repeat unit and the intercept gives the end group molar mass. 50
Fig. S42 MALDI-TOF Spectrum for PVL produced using $Zn(Oct)_2$ catalyst, focusing on peaks identified as cyclic PVL (doped with Na^+). The polymer (10 mg mL^{-1} in THF), and dithranol (10 mg mL^{-1} in THF) were premixed in a 1:25 ratio (v/v). 50
Fig. S43 The experimental spectrum stacked with an isotope simulated spectrum for $[C_5H_8O_2]_{20}$, Na^+ m/z = 2025.39. 51
Fig. S44 m/z *vs*. repeat unit plot for the fourth series of identified peaks from MALDI-TOF analysis of PVL produced using the $Zn(Oct)_2$ catalyst, where the slope gives the repeat unit and the intercept gives the end group molar mass. 52
Fig. S45 MALDI-TOF Spectrum for PVL produced using $Zn(Oct)_2$ catalyst, focusing on peaks identified as PVL with a Zn(Oct) end group (doped with Na^+). The polymer (10 mg mL^{-1} in THF), and dithranol (10 mg mL^{-1} in THF) were premixed in a 1:25 ratio (v/v). 52
Fig. S46 The experimental spectrum stacked with an isotope simulated spectrum for $HO[C_5H_8O_2]_{40}Zn(CO_2(CH(C_4H_9)(C_2H_5)))$, Na^+ m/z = 4253.26. 53

Fig. S47 MALDI-TOF Spectrum for PVL produced using $Bi(Oct)_3$ catalyst. The polymer (10 mg mL^{-1} in THF), and dithranol (10 mg mL^{-1} in THF) were premixed in a 1:25 ratio (v/v). Identified end group structures are given and these are analyzed in detail in the following figures (Fig. S48-S59). 54
Fig. S48 m/z *vs*. repeat unit plot for the first series of identified peaks from MALDI-TOF analysis of PVL produced using the $Bi(Oct)_3$ catalyst, where the slope gives the repeat unit and the intercept gives the end group molar mass. 54
Fig. S49 MALDI-TOF Spectrum for PVL produced using $Bi(Oct)_3$ catalyst, focusing on peaks identified as PVL with a hydroxyl end group (doped with Na^+). The polymer (10 mg mL^{-1} in THF), and dithranol (10 mg mL^{-1} in THF) were premixed in a 1:25 ratio (v/v). 55
Fig. S50 The experimental spectrum stacked with an isotope simulated spectrum for $HO[C_5H_8O_2]_{10}H$, Na^+ m/z = 1042.17. 56
Fig. S51 m/z *vs*. repeat unit plot for the second series of identified peaks from MALDI-TOF analysis of PVL produced using the $Bi(Oct)_3$ catalyst, where the slope gives the repeat unit and the intercept gives the end group molar mass. 57
Fig. S52 MALDI-TOF Spectrum for PVL produced using $Bi(Oct)_3$ catalyst, focusing on peaks identified as PVL with a hydroxyl end group (doped with K^+). The polymer (10 mg mL^{-1} in THF), and dithranol (10 mg mL^{-1} in THF) were premixed in a 1:25 ratio (v/v). 57
Fig. S53 The experimental spectrum stacked with an isotope simulated spectrum for $HO[C_5H_8O_2]_{10}H$, K^+ m/z = 1058.28..58
Fig. S54 m/z *vs*. repeat unit plot for the third series of identified peaks from MALDI-TOF analysis of PVL produced using the $Bi(Oct)_3$ catalyst, where the slope gives the repeat unit and the intercept gives the end group molar mass. 59
Fig. S55 MALDI-TOF Spectrum for PVL produced using $Bi(Oct)_3$ catalyst, focusing on peaks identified as PVL with a benzyl alcohol end group (doped with Na^+). The polymer (10 mg mL^{-1} in THF), and dithranol (10 mg mL^{-1} in THF) were premixed in a 1:25 ratio (v/v). 59
Fig. S56 The experimental spectrum stacked with an isotope simulated spectrum for $C_7H_8O[C_5H_8O_2]_{10}H$, Na^+ m/z = 1132.30. 60
Fig. S57 m/z *vs*. repeat unit plot for the fourth series of identified peaks from MALDI-TOF analysis of PVL produced using the $Bi(Oct)_3$ catalyst, where the slope gives the repeat unit and the intercept gives the end group molar mass. 61
Fig. S58 MALDI-TOF Spectrum for PVL produced using $Bi(Oct)_3$ catalyst, focusing on peaks identified as PVL with a benzyl alcohol end group (doped with K^+). The polymer (10 mg mL^{-1} in THF), and dithranol (10 mg mL^{-1} in THF) were premixed in a 1:25 ratio (v/v). 61
Fig. S59 The experimental spectrum stacked with an isotope simulated spectrum for $C_7H_8O[C_5H_8O_2]_{10}H$, K^+ m/z = 1148.41. 62
Table S9 k_{obs} determined at incrementally increasing catalyst loadings for the polymerization of CL using the $Bi(Oct)_3$ catalyst and BnOH chain-transfer agent at 200 °C. 63
Fig. S60 Plots of [PCL] *vs*. time for isotherms conducted at 200 °C. k_{obs} is taken from the exponential fitting of each line (y = A - Be^{-kobsx}), with the average k_{obs} taken as the mean of the three repeats and error determined from the standard deviation of the mean of the three repeats. Polymerization conditions: 0.5:5:1000 $Bi(Oct)_3$:BnOH:CL. 63
Fig. S61 Plot of k_{obs} *vs*. $[Bi(Oct)_3]$ for isotherms conducted at 200 °C with polymerization conditions: x:10x:1000 $Bi(Oct)_3$:BnOH:CL where x= 0.5, 1, 5, 10, and 20, [CL] = 6.25 M. 64
Fig. S62 Plot of ln(k_{obs}) *vs*. ln($[Bi(Oct)_3]$) for isotherms conducted at 200 °C with polymerization conditions: x:10x:1000 $Bi(Oct)_3$:BnOH:CL where x= 0.5, 1, 5, 10, and 20, [CL] = 6.25 M..........................64
Fig. S63 Plots of $k_p^{(1-m)}$ *vs*. $[cat]_0 k_p^{-m}$ where m = estimated degree of aggregation = 2, 3, 4, 5, 10 and 15. Polymerization conditions: x:10x:1000 $Bi(Oct)_3$:BnOH:CL where x= 0.5, 1, 5, 10, and 20, [CL] = 6.25 M, 200 °C. 65
Fig. S64 IR Spectra of monomers VL, TMC, CL, MeCL, DTC and *L*-LA, indicating the C=O stretching frequency in each case. 66
Fig. S65 ^1H NMR spectrum (400 MHz, $CDCl_3$, 298 K) of TMC. 67
Fig. S66 $^{13}C\{^1H\}$ NMR spectrum (400 MHz, $CDCl_3$, 298 K) of TMC. 67
Fig. S67 ^1H NMR spectrum (400 MHz, $CDCl_3$, 298 K) of VL. 68

Fig. S68 $^{13}C\{^1H\}$ NMR spectrum (400 MHz, $CDCl_3$, 298 K) of VL. 68
Fig. S69 ^1H NMR spectrum (400 MHz, $CDCl_3$, 298 K) of CL. 69
Fig. S70 $^{13}C\{^1H\}$ NMR spectrum (400 MHz, $CDCl_3$, 298 K) of CL. 69
Fig. S71 ^1H NMR spectrum (400 MHz, $CDCl_3$, 298 K) of MeCL. 70
Fig. S72 $^{13}C\{^1H\}$ NMR spectrum (400 MHz, $CDCl_3$, 298 K) of MeCL. 70
Fig. S73 ^1H NMR spectrum (400 MHz, $CDCl_3$, 298 K) of DTC. 71
Fig. S74 $^{13}C\{^1H\}$ NMR spectrum (400 MHz, $CDCl_3$, 298 K) of DTC. 71
Fig. S75 ^1H NMR spectrum (400 MHz, $CDCl_3$, 298 K) of *L*-LA. 72
Fig. S76 $^{13}C\{^1H\}$ NMR spectrum (400 MHz, $CDCl_3$, 298 K) of *L*-LA. 72
Fig. S77 Zoomed in stacked $^{13}C\{^1H\}$ NMR spectra (400 MHz, $CDCl_3$, 298 K) of MeCL and CL. The full spectrum for CL can be found in Fig. S70 and the full spectrum for MeCL can be found in Fig. S72. 73
Fig. S78 Plots of $\ln(k_p/T)$ *vs*. 1/T as a part of Eyring analyses to determine transition state enthalpy ($\Delta H^‡_{pol}$), entropy ($\Delta S^‡_{pol}$) and Gibbs energy of activation ($\Delta G^‡_{pol}$) for polymerization of VL, using each $M(Oct)_n$ (M= Zn(II), Sn(II), Bi(III), Zr(IV), Mg(II)) catalyst. Polymerization conditions: 1:10:1000 $M(Oct)_n$:BnOH:VL, [VL] = 6.25 M. 74
Fig. S79 Plots of $\ln(k_p/T)$ *vs*. 1/T as a part of Eyring analyses to determine transition state enthalpy ($\Delta H^‡_{pol}$), entropy ($\Delta S^‡_{pol}$) and Gibbs energy of activation ($\Delta G^‡_{pol}$) for polymerization of CL, using the $Zn(Oct)_2$ and $Sn(Oct)_2$ catalysts. Polymerization conditions: 1:10:1000 $M(Oct)_n$:BnOH:CL, [CL] = 6.25 M. 75
Table S10 Polymerization Rate Data Table for the Polymerization of *L*-LA to PLLA using $Zn(Oct)_2$ and $Sn(Oct)_2$ catalysts. Polymerization conditions: 1:10:250 $M(Oct)_2$:BnOH:*L*-LA, [*L*-LA] = 1.56 M. 76
Fig. S80 Representative ^1H NMR spectrum (400 MHz, $CDCl_3$, 298 K) for calculation of conversion of *L*-LA to PLLA. Polymerization conditions: 1:10:250 $M(Oct)_2$:BnOH:*L*-LA, [*L*-LA] = 1.56 M, at 160 °C. 76
Fig. S81 Isothermal polymerization kinetic experiments, conducted on a DSC instrument, for polymerization of *L*-LA (conducted in triplicate). (a) Polymerization conditions: 1:10:250 $Zn(Oct)_2$:BnOH:*L*-LA, [*L*-LA] = 1.56 M, 160 °C. (b) Polymerization conditions: 1:10:250 $Sn(Oct)_2$:BnOH:*L*-LA, [*L*-LA] = 1.56 M, 160 °C. 77
Fig. S82 IR Spectra of the benzoic acid starting material and corresponding bis Mg(II) benzoate species. Disappearance of the C=O stretch and carboxylic acid O-H stretch present in the starting material, is in line with the formation of the Mg(II)(carboxylate)$_2$ species. a) BzOH. b) $Mg(OBz)_2$. c) F_3CBzOH. d) $Mg(OBzCF_3)_2$. 77
Fig. S83 a) ^1H NMR Spectra of the $Mg(OBz)_2$ catalyst. b) Zoomed-in stacked ^1H NMR spectra of the BzOH starting material and $Mg(OBz)_2$ product. c) ^1H NMR spectrum of $Mg(OBzCF_3)_2$ catalyst. d) Zoomed-in stacked ^1H NMR spectra of the F_3CBzOH starting material and $Mg(OBzCF_3)_2$ product. Disappearance of the carboxylic acid O-H proton and shifting of the aromatic signals is observed in line with formation of the magnesium carboxylate species. 79
Table S11 Polymerization Data for the Polymerization of VL using MgR_2 (R = Oct, OBz, $OBzCF_3$) catalysts. Polymerization conditions: 1:10:1000 $Mg(OR)_2$:BnOH:VL, [VL] = 6.25 M. 80
Fig. S84 Isothermal polymerization kinetic experiments, conducted on a DSC instrument, for polymerization of VL (conducted in triplicate). (a) Polymerization conditions: 1:2:10:1000 $Mg(Bu)_2$:BzOH:BnOH:VL, [VL] = 6.25 M, 160 °C. (b) Polymerization conditions: 1:2:10:1000 $Mg(Bu)_2$:F_3CBzOH:BnOH:VL, [VL] = 6.25 M, 160 °C. (c) Polymerization conditions: 1:2:10:1000 $Mg(Bu)_2$:OctOH:BnOH:VL, [VL] = 6.25 M, 160 °C. 81
Fig. S85 Isothermal polymerization kinetic experiments, conducted on a DSC instrument, at 160 °C for the polymerization of VL with the $Zn(Oct)_2$ catalyst (plot in orange, top row, LHS), $Sn(Oct)_2$ catalyst (plot in green, top row, RHS), $Bi(Oct)_3$ catalyst (plot in purple, middle row, LHS), $Zr(Oct)_4$ catalyst (plot in blue, middle row, RHS), and $Mg(Oct)_2$ catalyst (plot in yellow, bottom row), run in neat VL. Polymerization conditions: 1:10:1000 $M(Oct)_n$:BnOH:VL. 82
Table S12 Polymerization data for polymerization of VL in absence of solvent. Polymerization conditions: 1:10:1000 $M(Oct)_n$:BnOH:VL, neat in VL. 83

4. *References* *83*

1. Materials

Anhydrous solvents were obtained from a solvent purification system (SPS), degassed using three freeze-pump-thaw cycles and stored over 3 Å molecular sieves, under a N_2 atmosphere. Mg(II)$(Oct)_2$ was purchased from Strem Chemicals. Zn(II)$(Oct)_2$ was purchased from Fluorochem. Sn(II)$(Oct)_2$ was purchased from Sigma-Aldrich. Bi(III)$(Oct)_3$ was purchased from Thermo Fisher Scientific. Zr(IV)$(Oct)_4$ was purchased from Alfa Aesar. ε-caprolactone (CL) was purchased from Sigma-Aldrich. δ-valerolactone (VL) was purchased from Alfa Aesar. Trimethylene carbonate (TMC) was purchased from Tokyo Chemical Industry. All lactones were stirred over CaH_2 before being distilled under reduced pressure. All metal (2-ethylhexanoate) catalysts were dried under high vacuum, and stored in a glovebox, under a N_2 atmosphere. γ-methyl-ε-caprolactone (MeCL) was synthesised according to literature.[1] 2,2-dimethyltrimethylene carbonate (DTC) was synthesised according to literature.[2] Prior to use, DTC and TMC were twice recrystallised from anhydrous diethyl ether. All lactone and cyclic carbonate monomers were stored in a glovebox, under a N_2 atmosphere.

NMR Spectroscopy

^{1}H and ^{13}C{^{1}H} NMR spectroscopic analysis was performed using a 400 MHz Bruker Avance III HD Nanobay spectrometer equipped with a 9.4 T magnet, at 298 K.

Gel Permeation Chromatography (GPC)

Gel permeation chromatography (GPC) was performed on a Shimadzu LC-20AD instrument with two mixed bed PSS SDV linear S columns in series at 40 °C. THF was used as the eluent with a flow rate of 1 mL min^{-1}. Molar mass calibration was conducted with a narrow molar mass polystyrene standard.

Differential Scanning Calorimetry (DSC)

Differential scanning calorimetry (DSC) was performed using a TA Discovery 25-Auto. A sealed empty pan was used as a reference, and the DSC temperature and enthalpy were calibrated using indium. (See below for a detailed outline on the procedure for determination of kinetic parameters using DSC).

Infrared (IR) Spectroscopy

FT-IR measurements were conducted with a Shimadzu IRSpirit spectrometer (inside a N_2 glove box) using a single reflection ATR accessory.

Matrix-Assisted Laser Desorption/Ionisation Time of Flight (MALDI-TOF) Mass Spectrometry

MALDI-TOF analyses were conducted on a Bruker MALDI-MS Autoflex Speed instrument, set to positive ion reflectron mode. Sample solutions of polymer (10 mg mL^{-1} in THF), and dithranol (10 mg mL^{-1} in THF) were premixed in a 1:25 ratio. Each sample was spotted three times onto a metal plate and allowed to dry completely before analysis.

2. Methods

Procedure for DSC Kinetic Experiments[3]

The same procedure was followed for all DSC experiments for polymerization of the 6- and 7-membered lactones and cyclic carbonates with the $M(Oct)_n$ catalysts. A representative method for VL ROP under conditions of 1:10:1000 $Zn(Oct)_2$:BnOH:VL is detailed here: A solution of VL (48.6 µL, 0.5 mmol), $Zn(Oct)_2$ and BnOH stock solution (5 µL of 0.1M $Zn(Oct)_2$ and 1M BnOH in 1,2-dimethoxybenzene), and 1,2-dimethoxybenzene (26.4 µL, to obtain final $[VL]_0$ = 6.25 M) was prepared under a N_2 atmosphere. 5 µL of this solution was added to a pre-weighed hermetic DSC pan, and the pan pressed out to seal the reaction mixture. The pan was reweighed to calculate the total sample mass in the pan, used for further kinetic analyses. The sample was run using a hermetic reference with the desired reaction parameters inputted on the instrument (load temperature, heating rate, or isotherm). All reactions were run under a N_2 flow of 50 mL min^{-1}. After the reaction, the pan was retrieved and a sample of the final reaction mixture taken for NMR and GPC analysis.

Procedure for $^{31}P\{^1H\}$ NMR Studies to Probe Metal Catalyst Lewis Acidity[4]

In order to understand the relationship between the pK_h values of the metals and the Lewis acidity of the metal catalysts, a series of ^{31}P NMR spectroscopy experiments were conducted. Trioctylphosphine oxide (TOPO) was selected as an appropriate probe.[5] Solutions of 1:20:200 TOPO:$M(Oct)_n$:BnOH were made up in chloroform (i.e. 20 eq of metal relative to phosphine, and a fixed ratio of 1:10 M(Oct)n:BnOH to match conditions used for the polymerizations). A $^{31}P\{^1H\}$ NMR spectrum was obtained, with a capillary tube containing orthophosphoric acid added as a reference (set to 0 ppm). For each $M(Oct)_n$, the $\Delta^{31}P$ NMR δ was determined relative to the chemical shift of only TOPO (no $M(Oct)_n$) (Fig. S1).

3. Additional Information

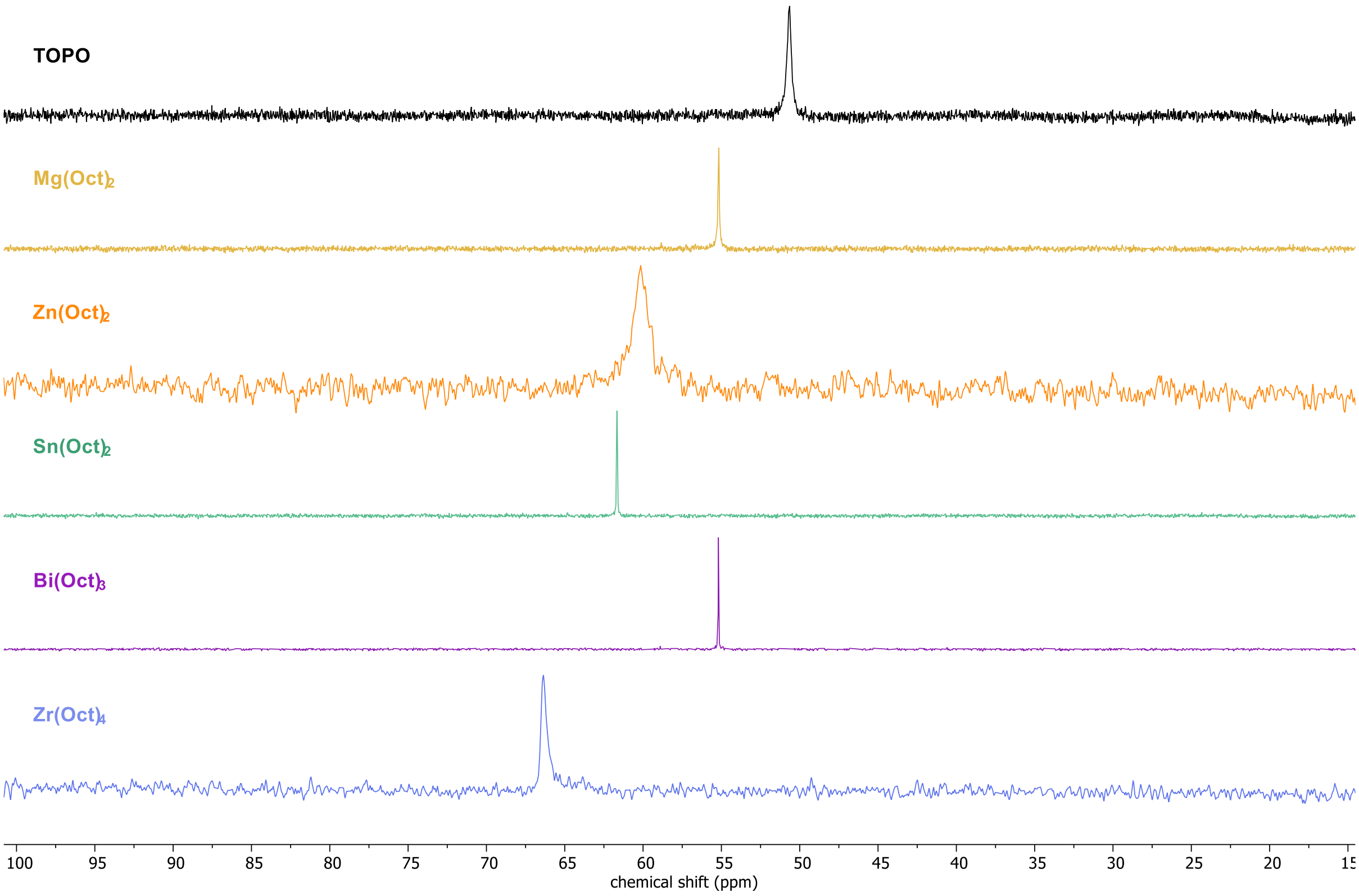

Fig. S1 Stacked $^{31}P\{^{1}H\}$ NMR spectra (400 MHz, $CDCl_3$, 298 K) for the Lewis acidity ordering of the $M(Oct)_n$ species coordinated to a trioctylphosphine oxide probe, relative to a phosphoric acid standard (contained in a capillary) set to 0 ppm.

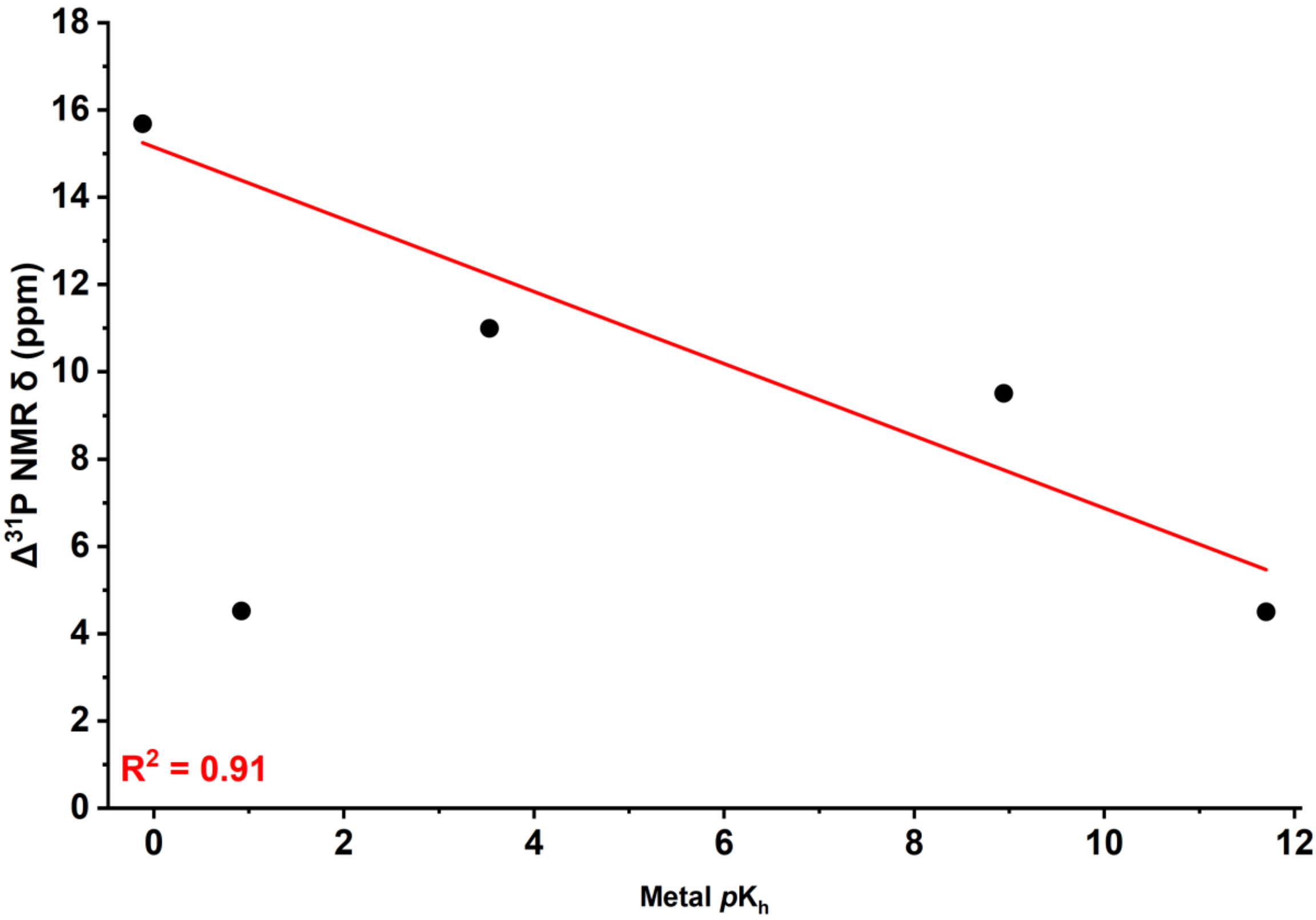

Fig. S2 Plot of Δ^{31}P NMR δ for a trioctylphosphine oxide coordinated M(Oct)$_n$ species relative to the trioctylphosphine oxide alone *vs*. the *p*K$_h$ values for each metal. A lower than expected shift for Bi(Oct)$_3$ is proposed to occur due to aggregation (Table S9, Fig. S60-S63) which reduces propensity for the catalyst to coordinate.

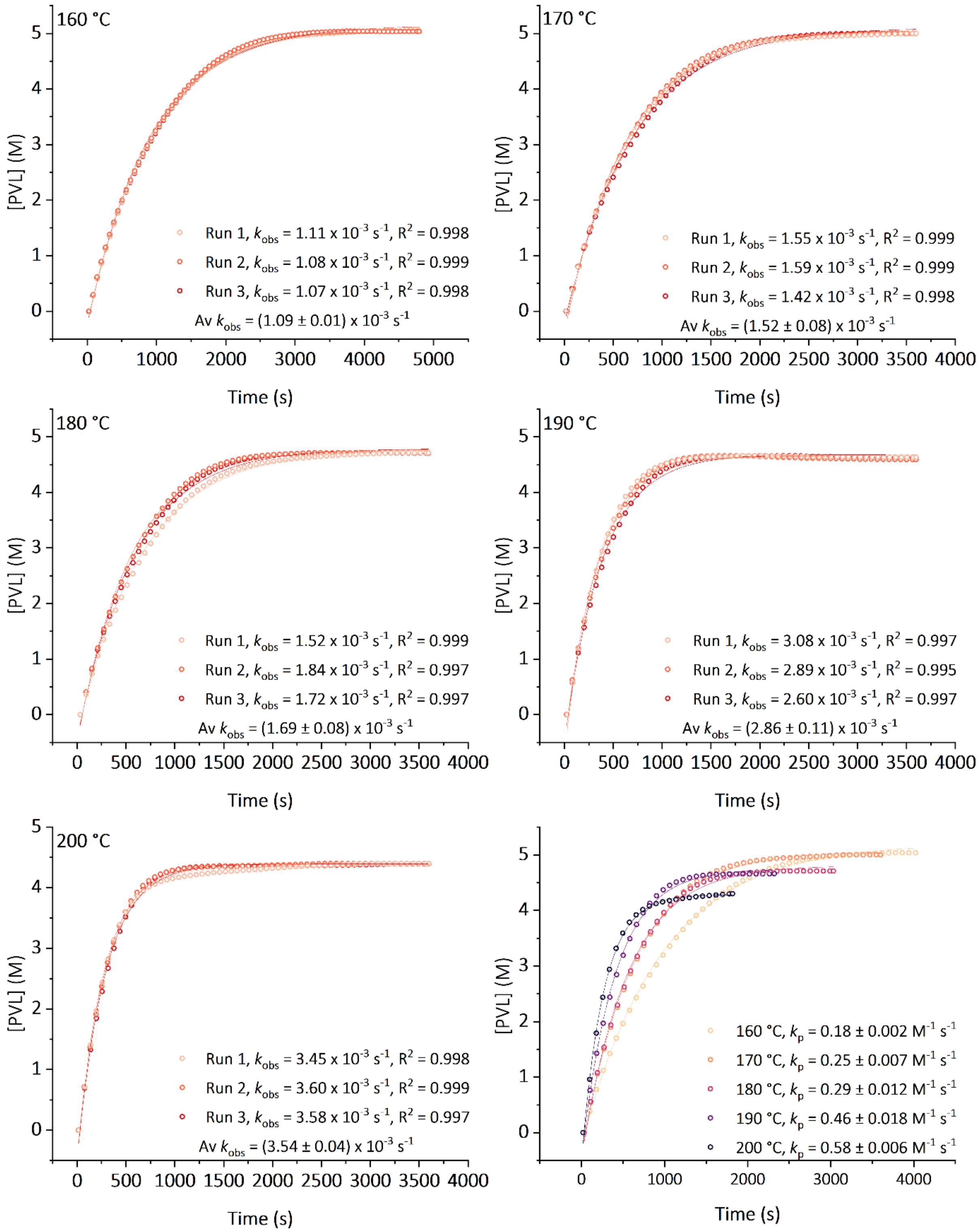

Fig. S3 Isothermal polymerization kinetic experiments, conducted on a DSC instrument, for each 10 °C temperature increment from 160 °C to 200 °C (conducted in triplicate) for the polymerization of VL with the $Zn(Oct)_2$ catalyst. The stacked plot shows one representative dataset at each temperature. For clarity, every 600th data point from the datasets is shown. Polymerization conditions: 1:10:1000 $Zn(Oct)_2$:BnOH:VL, [VL] = 6.25 M. In each case $[PVL]_t = 1 - [VL]_t$.

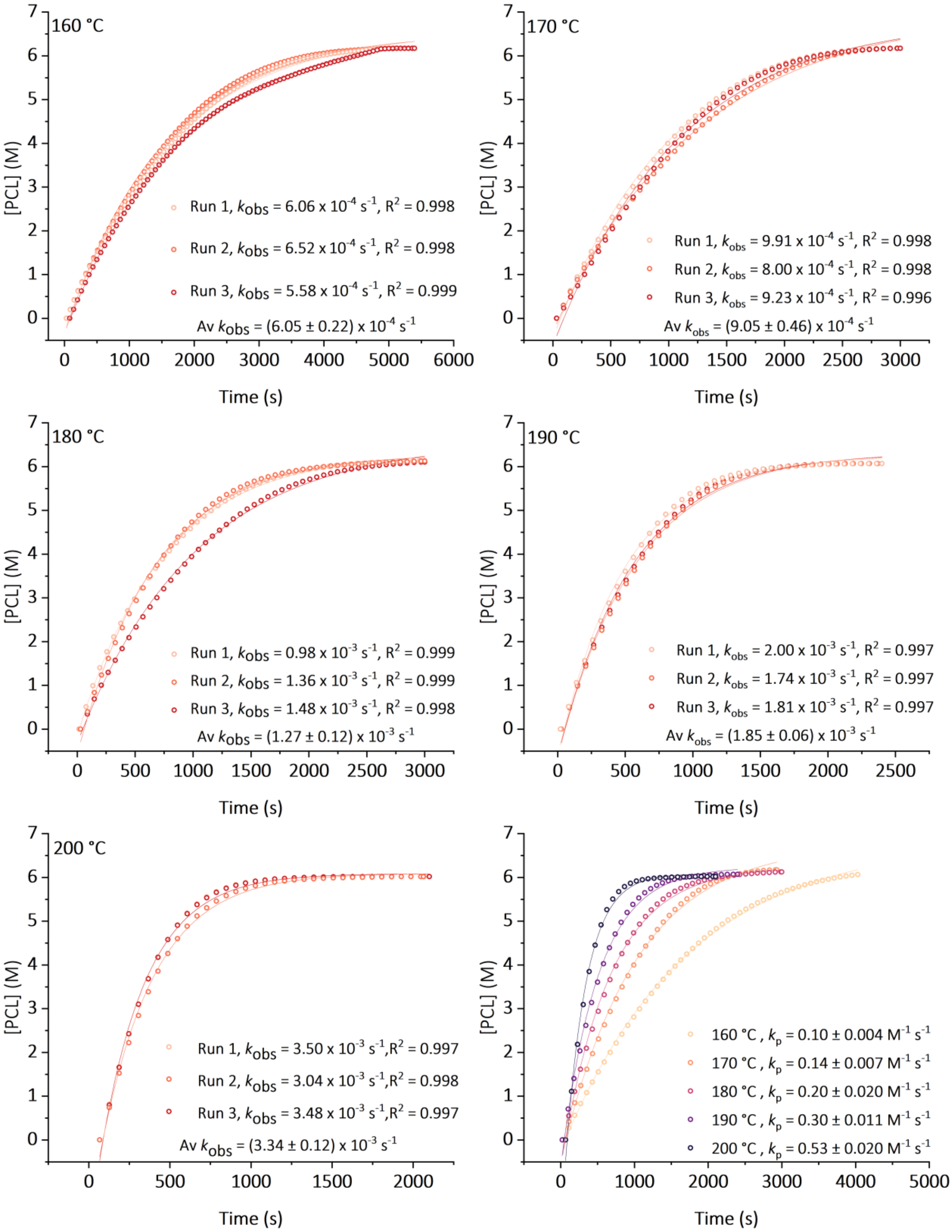

Fig. S4 Isothermal polymerization kinetic experiments, conducted on a DSC instrument, for each 10 °C temperature increment from 160 °C to 200 °C (conducted in triplicate) for the polymerization of CL with the Zn(Oct)2 catalyst. The stacked plot shows one representative dataset at each temperature. For clarity, every 600th data point from the datasets is shown. Polymerization conditions: 1:10:1000 Zn(Oct)2:BnOH:CL, [CL] = 6.25 M. In each case [PCL]t = 1 - [CL]t.

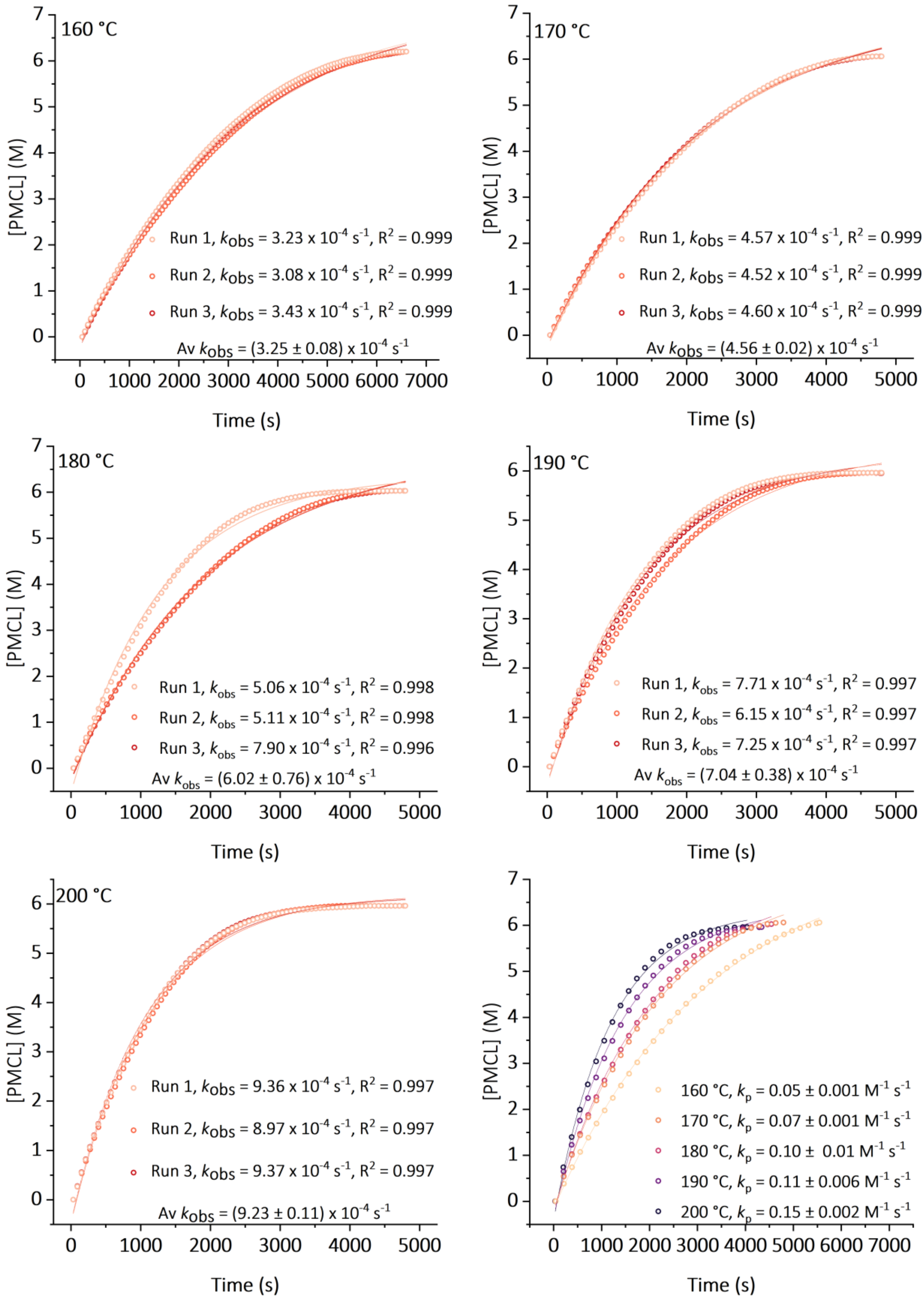

Fig. S5 Isothermal polymerization kinetic experiments, conducted on a DSC instrument, for each 10 °C temperature increment from 160 °C to 200 °C (conducted in triplicate) for the polymerization of MeCL with the $Zn(Oct)_2$ catalyst. The stacked plot shows one representative dataset at each temperature. For clarity, every 600th data point from the datasets is shown. Polymerization conditions: 1:10:1000 $Zn(Oct)_2$:BnOH:MeCL, [MeCL] = 6.25 M. In each case $[PMCL]_t = 1 - [MeCL]_t$.

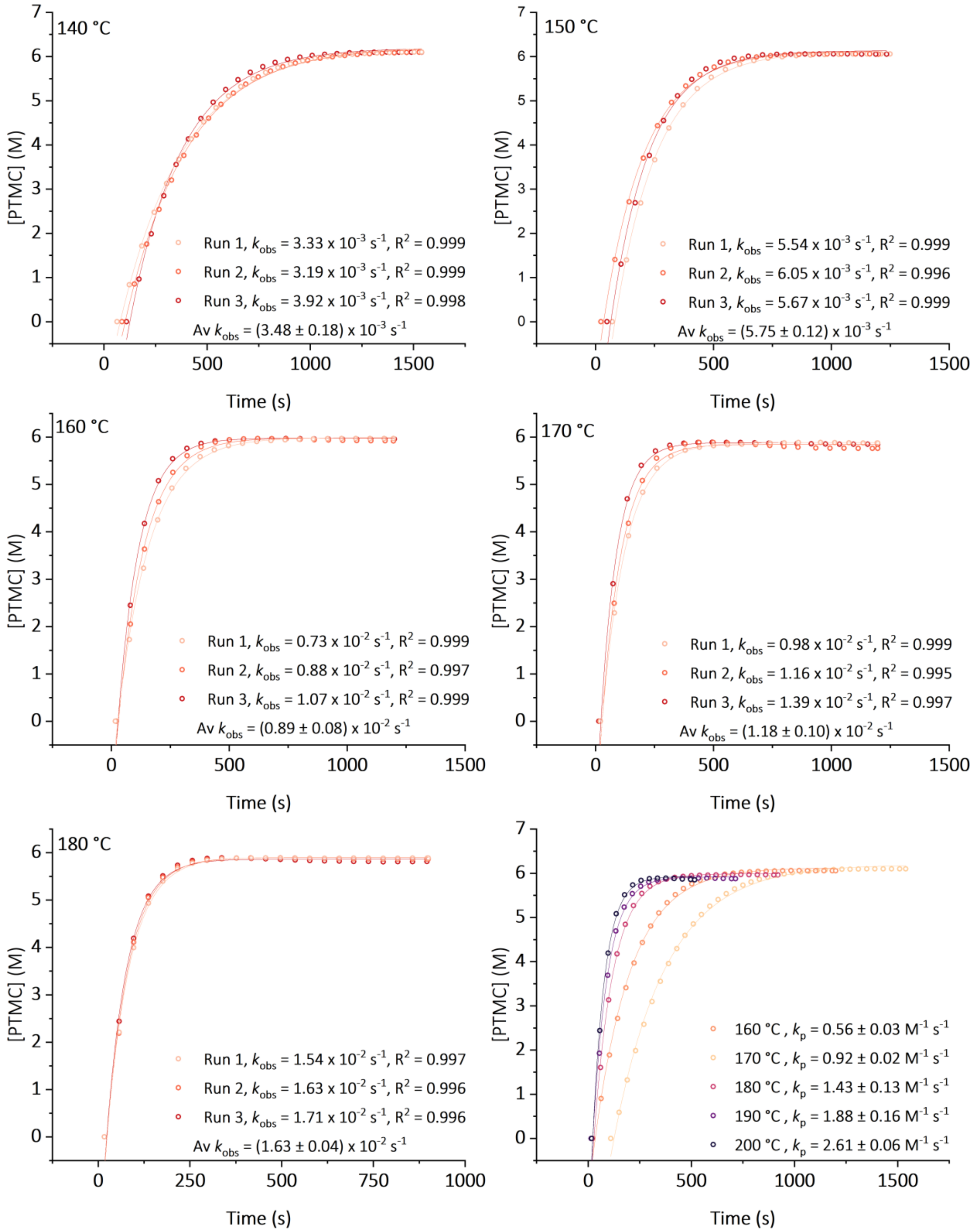

Fig. S6 Isothermal polymerization kinetic experiments, conducted on a DSC instrument, for each 10 °C temperature increment from 140 °C to 180 °C (conducted in triplicate) for the polymerization of TMC with the Zn(Oct)$_2$ catalyst. The stacked plot shows one representative dataset at each temperature. For clarity, every 600th data point from the datasets is shown. Polymerization conditions: 1:10:1000 Zn(Oct)$_2$:BnOH:TMC, [TMC] = 6.25 M. In each case [PTMC]$_t$ = 1 - [TMC]$_t$.

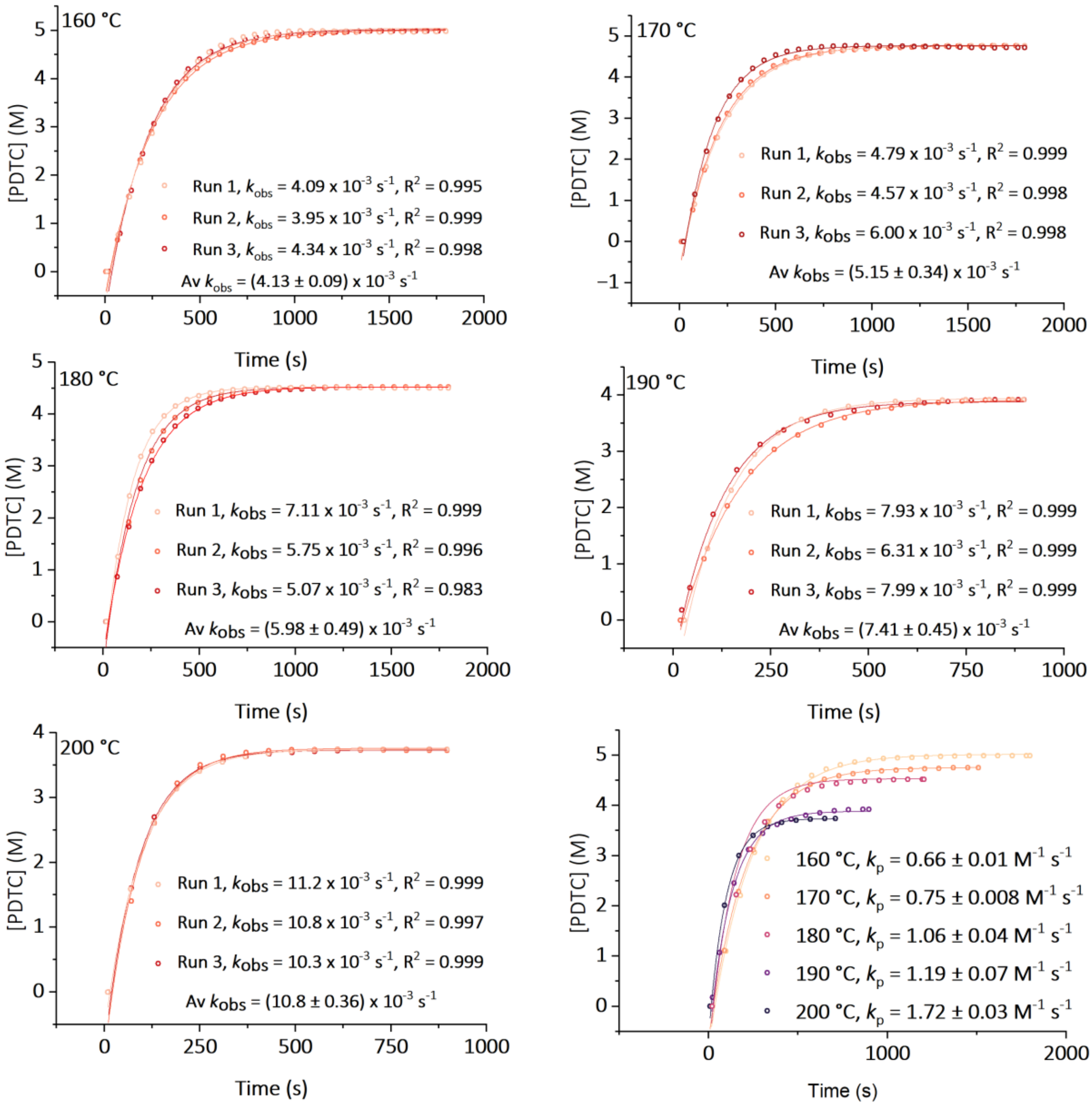

Fig. S7 Isothermal polymerization kinetic experiments, conducted on a DSC instrument, for each 10 °C temperature increment from 160 °C to 200 °C (conducted in triplicate) for the polymerization of DTC with the $Zn(Oct)_2$ catalyst. The stacked plot shows one representative dataset at each temperature. For clarity, every 600th data point from the datasets is shown. Polymerization conditions: 1:10:1000 $Zn(Oct)_2$:BnOH:DTC, [DTC] = 6.25 M. In each case $[PDTC]_t = 1 - [DTC]_t$.

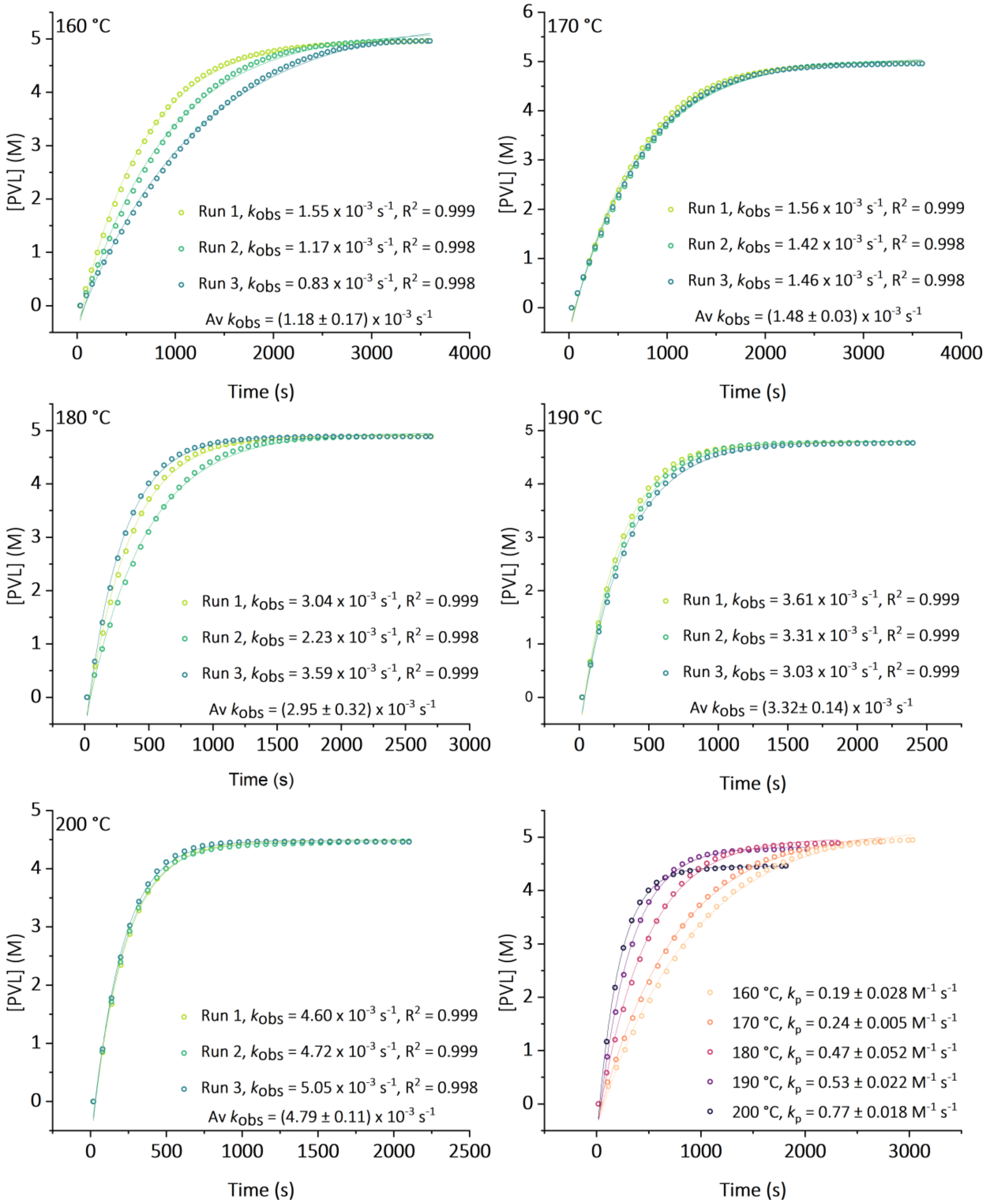

Fig. S8 Isothermal polymerization kinetic experiments, conducted on a DSC instrument, for each 10 °C temperature increment from 160 °C to 200 °C (conducted in triplicate) for the polymerization of VL with the $Sn(Oct)_2$ catalyst. The stacked plot shows one representative dataset at each temperature. For clarity, every 600th data point from the datasets is shown. Polymerization conditions: 1:10:1000 $Sn(Oct)_2$:BnOH:VL, [VL] = 6.25 M. In each case $[PVL]_t = 1 - [VL]_t$.

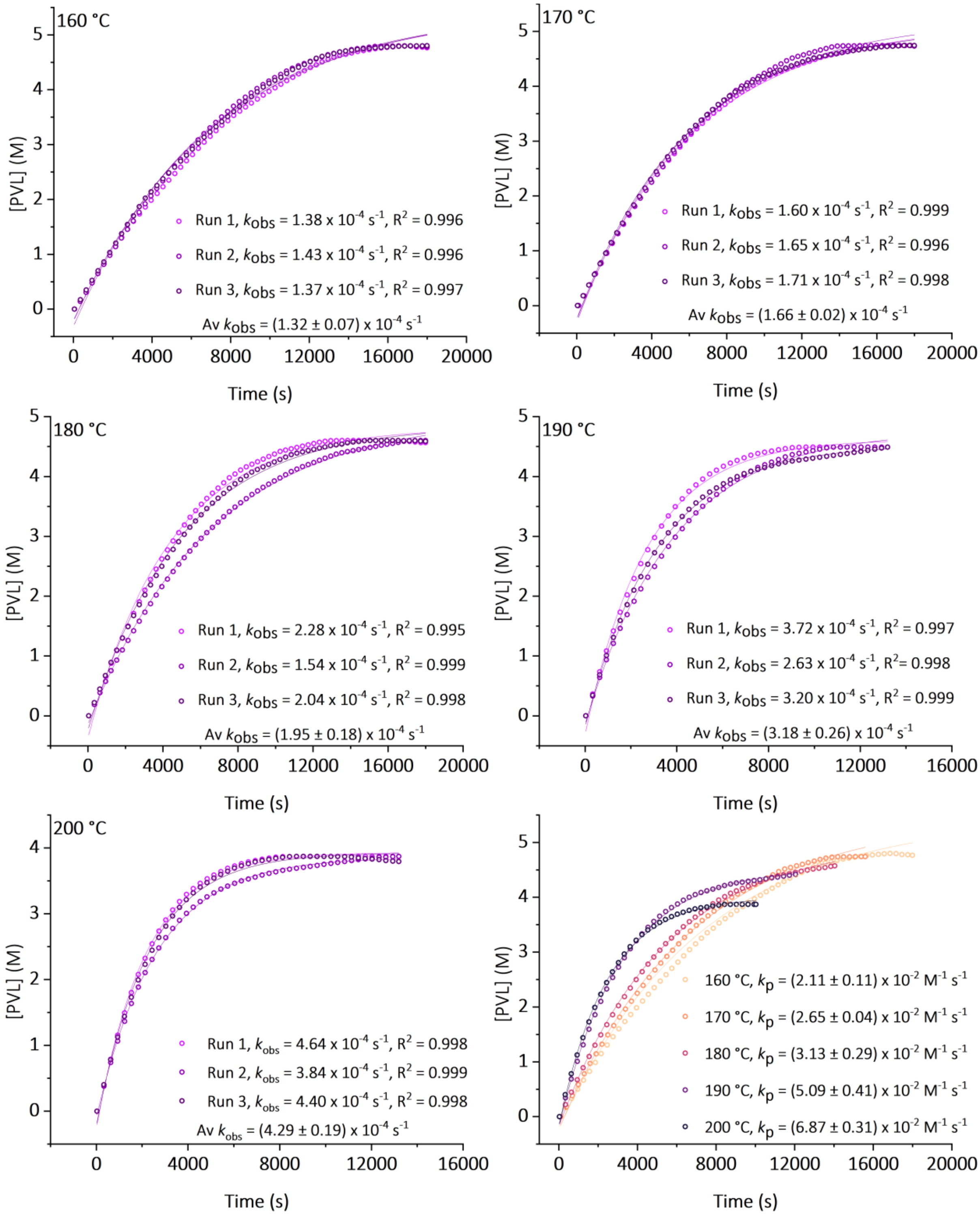

Fig. S9 Isothermal polymerization kinetic experiments, conducted on a DSC instrument, for each 10 °C temperature increment from 160 °C to 200 °C (conducted in triplicate) for the polymerization of VL with the Bi(Oct)$_3$ catalyst. The stacked plot shows one representative dataset at each temperature. For clarity, every 1000th data point from the datasets is shown. Polymerization conditions: 1:10:1000 Bi(Oct)$_3$:BnOH:VL, [VL] = 6.25 M. In each case [PVL]$_t$ = 1 - [VL]$_t$.

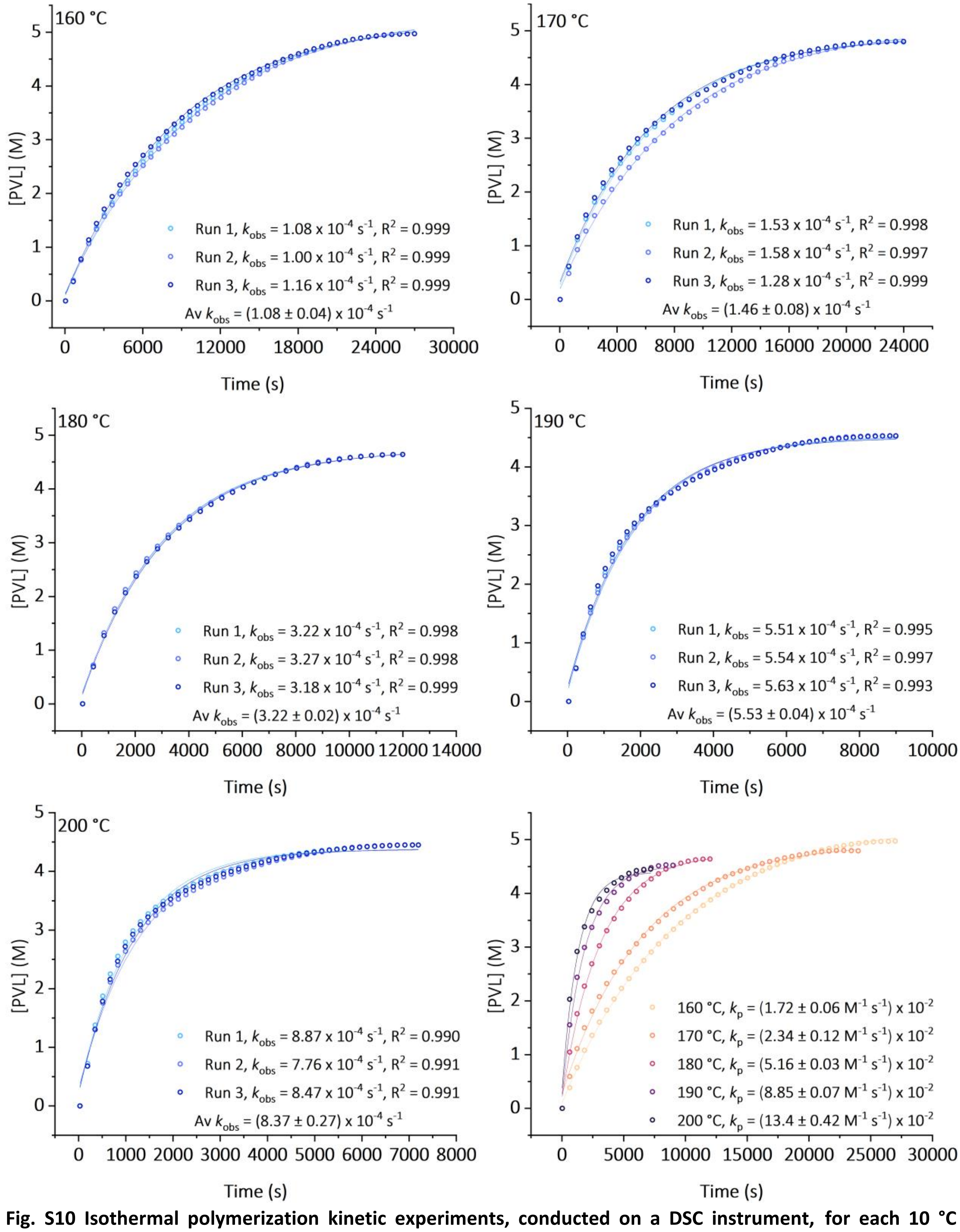

Fig. S10 Isothermal polymerization kinetic experiments, conducted on a DSC instrument, for each 10 °C temperature increment from 160 °C to 200 °C (conducted in triplicate) for the polymerization of VL with the $Zr(Oct)_4$ catalyst. The stacked plot shows one representative dataset at each temperature. For clarity, every 6000th data point from the datasets is shown. Polymerization conditions: 1:10:1000 $Zr(Oct)_4$:BnOH:VL, [VL] = 6.25 M. In each case $[PVL]_t = 1 - [VL]_t$.

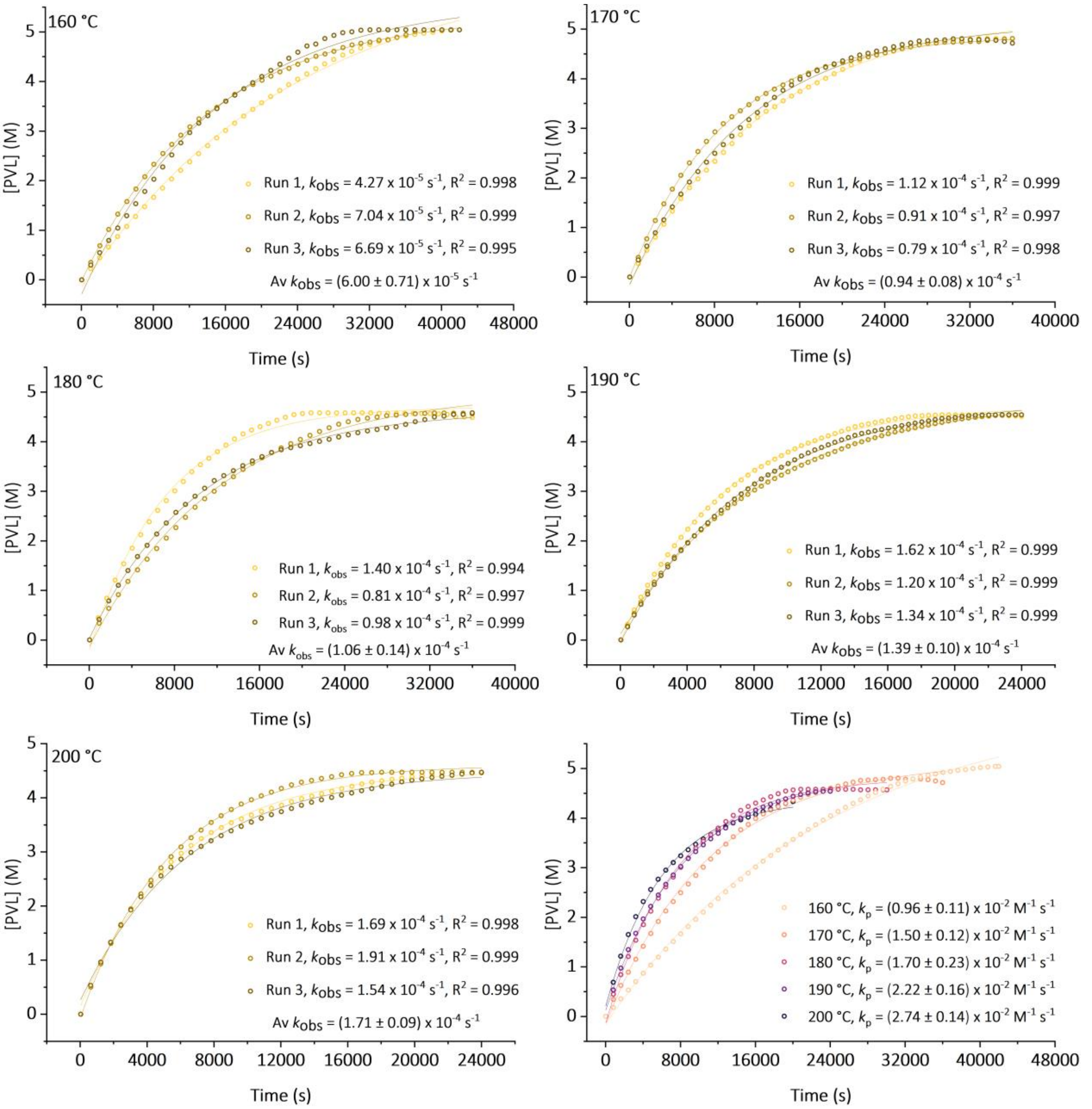

Fig. S11 Isothermal polymerization kinetic experiments, conducted on a DSC instrument, for each 10 °C temperature increment from 160 °C to 200 °C (conducted in triplicate) for the polymerization of VL with the $Mg(Oct)_2$ catalyst. The stacked plot shows one representative dataset at each temperature. For clarity, every 8000th data point from the datasets is shown. Polymerization conditions: 1:10:1000 $Mg(Oct)_2$:BnOH:VL, [VL] = 6.25 M. In each case $[PVL]_t$ = 1 - $[VL]_t$.

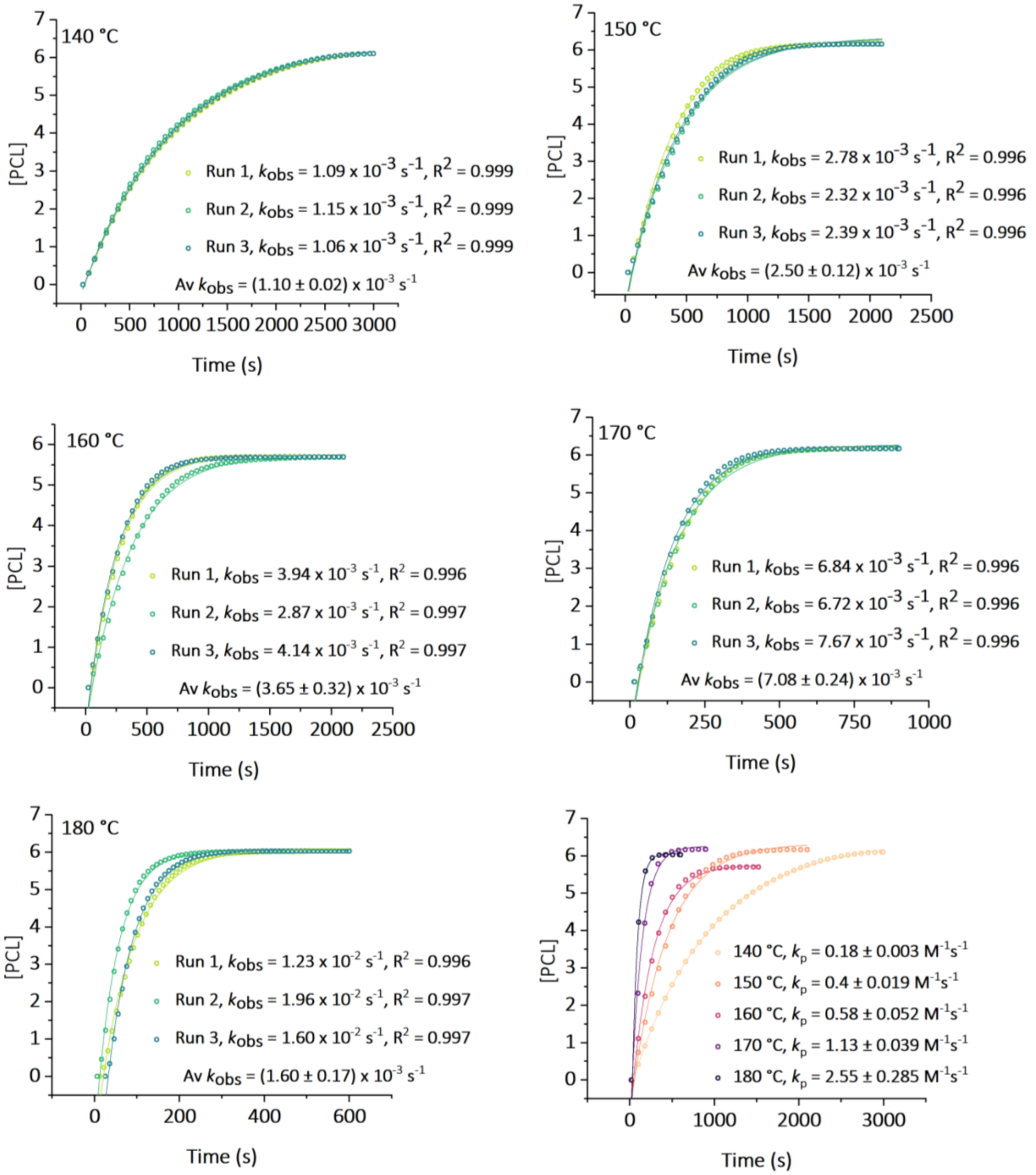

Fig. S12 Isothermal polymerization kinetic experiments, conducted on a DSC instrument, for each 10 °C temperature increment from 140 °C to 180 °C (conducted in triplicate) for the polymerization of CL with the $Sn(Oct)_2$ catalyst. The stacked plot shows one representative dataset at each temperature. For clarity, every nth data point from the datasets is shown (n = 600, 400, 400, 200, and 100 for 140 °C, 150 °C, 160 °C, 170 °C and 180 °C respectively). Polymerization conditions: 1:10:1000 $Sn(Oct)_2$:BnOH:CL, [CL] = 6.25 M. In each case $[PCL]_t = 1 - [CL]_t$.

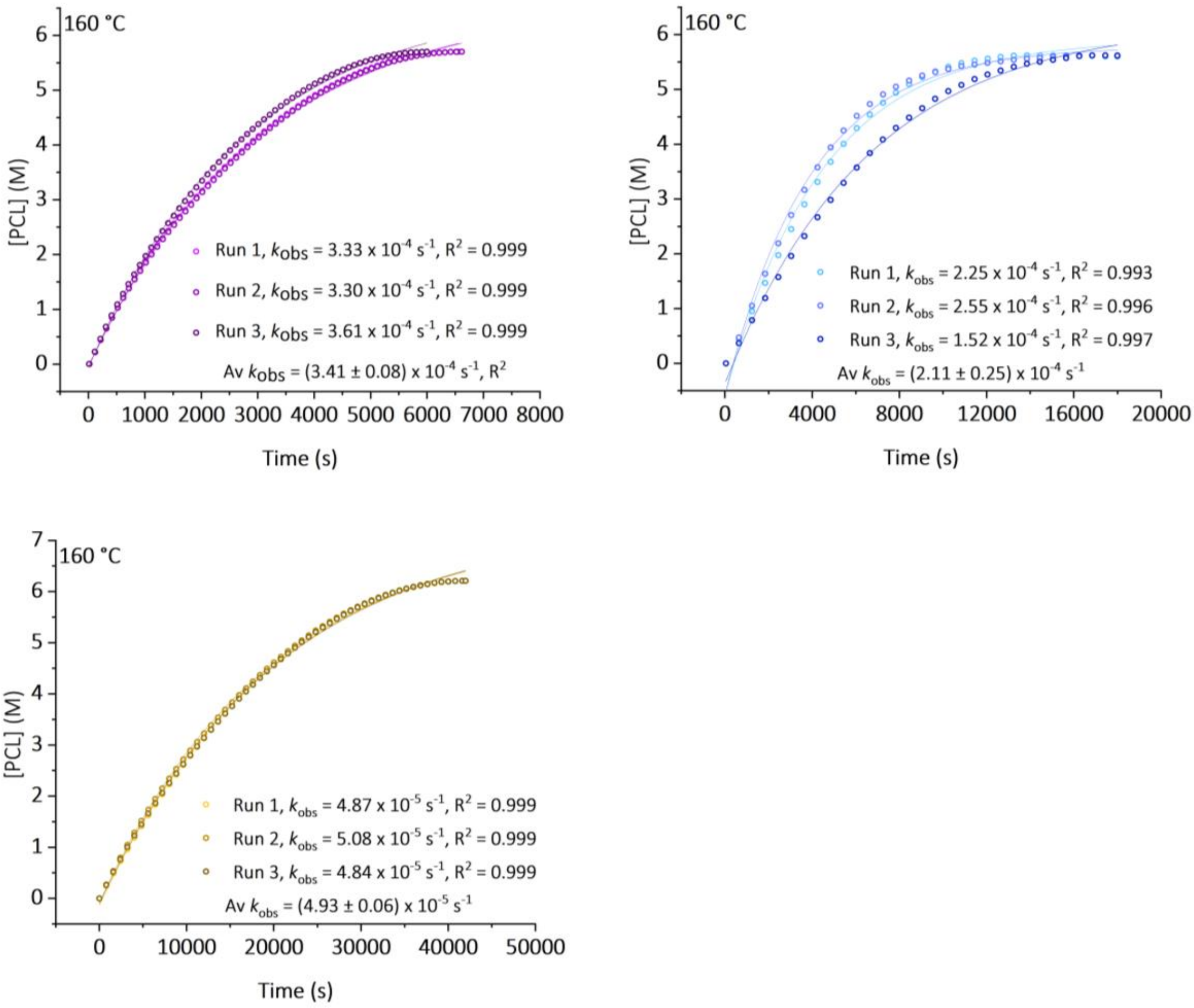

Fig. S13 Isothermal polymerization kinetic experiments, conducted on a DSC instrument, at 160 °C for the polymerization of CL with the $Bi(Oct)_3$ catalyst (plot in purple, top LHS), $Zr(Oct)_4$ catalyst (plot in blue, top RHS), and $Mg(Oct)_2$ catalyst (plot in yellow, bottom RHS). For clarity, every nth data point from the datasets is shown (n= 1000, 6000, 8000 for Bi(III), Zr(IV), Mg(II) respectively). Polymerization conditions: 1:10:1000 $M(Oct)_n$:BnOH:CL, [CL] = 6.25 M. In each case $[PCL]_t = 1 - [CL]_t$.

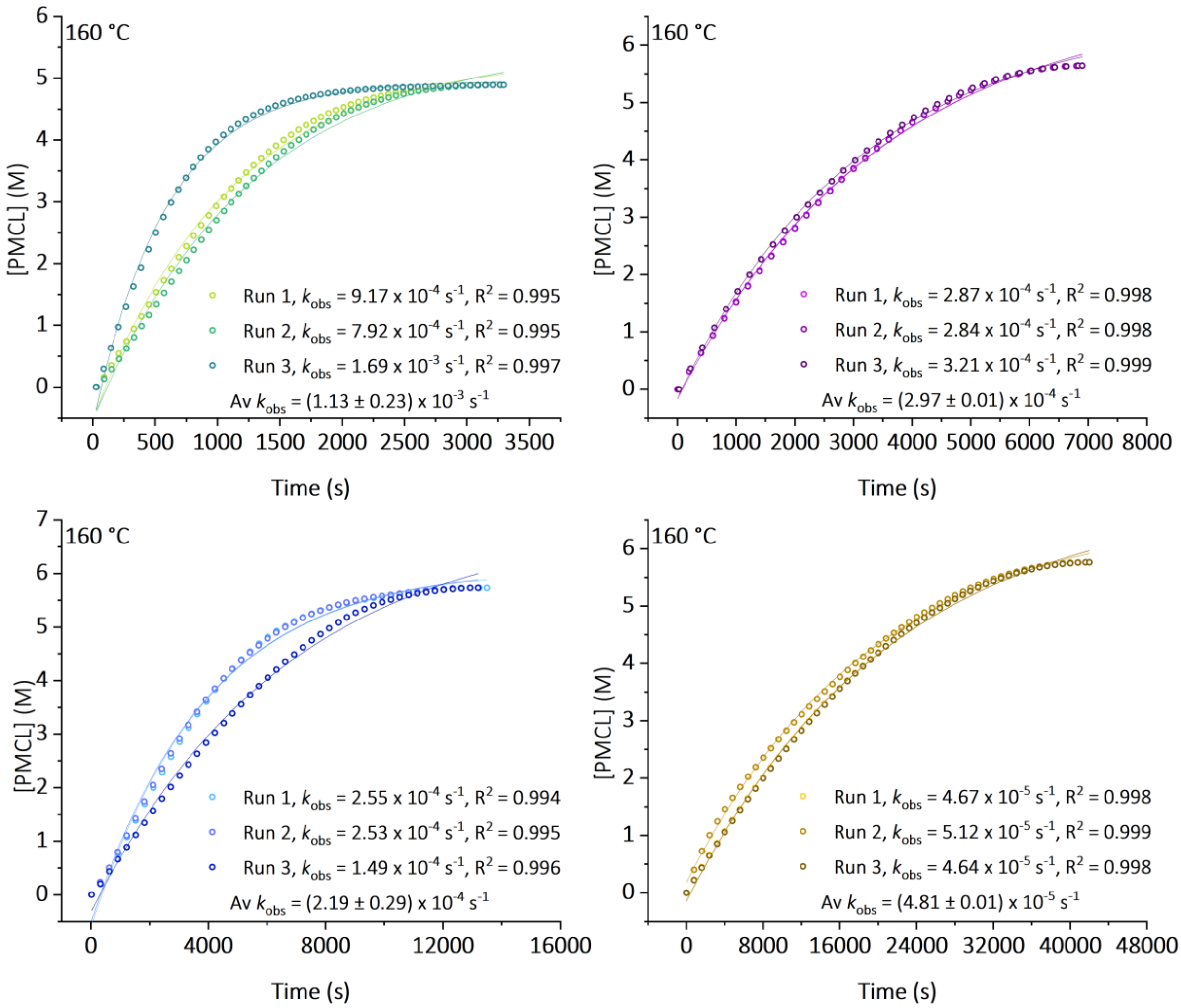

Fig. S14 Isothermal polymerization kinetic experiments, conducted on a DSC instrument, at 160 °C for the polymerization of MeCL with the $Sn(Oct)_2$ catalyst (plot in green, top LHS), $Bi(Oct)_3$ catalyst (plot in purple, top RHS), $Zr(Oct)_4$ catalyst (plot in blue, bottom LHS), and $Mg(Oct)_2$ catalyst (plot in yellow, bottom RHS). For clarity, every nth data point from the datasets is shown (n= 600, 1000, 6000, 8000 for Sn(II), Bi(III), Zr(IV), Mg(II) respectively). Polymerization conditions: 1:10:1000 $M(Oct)_n$:BnOH:MeCL, [MeCL] = 6.25 M. In each case $[PMCL]_t$ = 1 - $[MeCL]_t$.

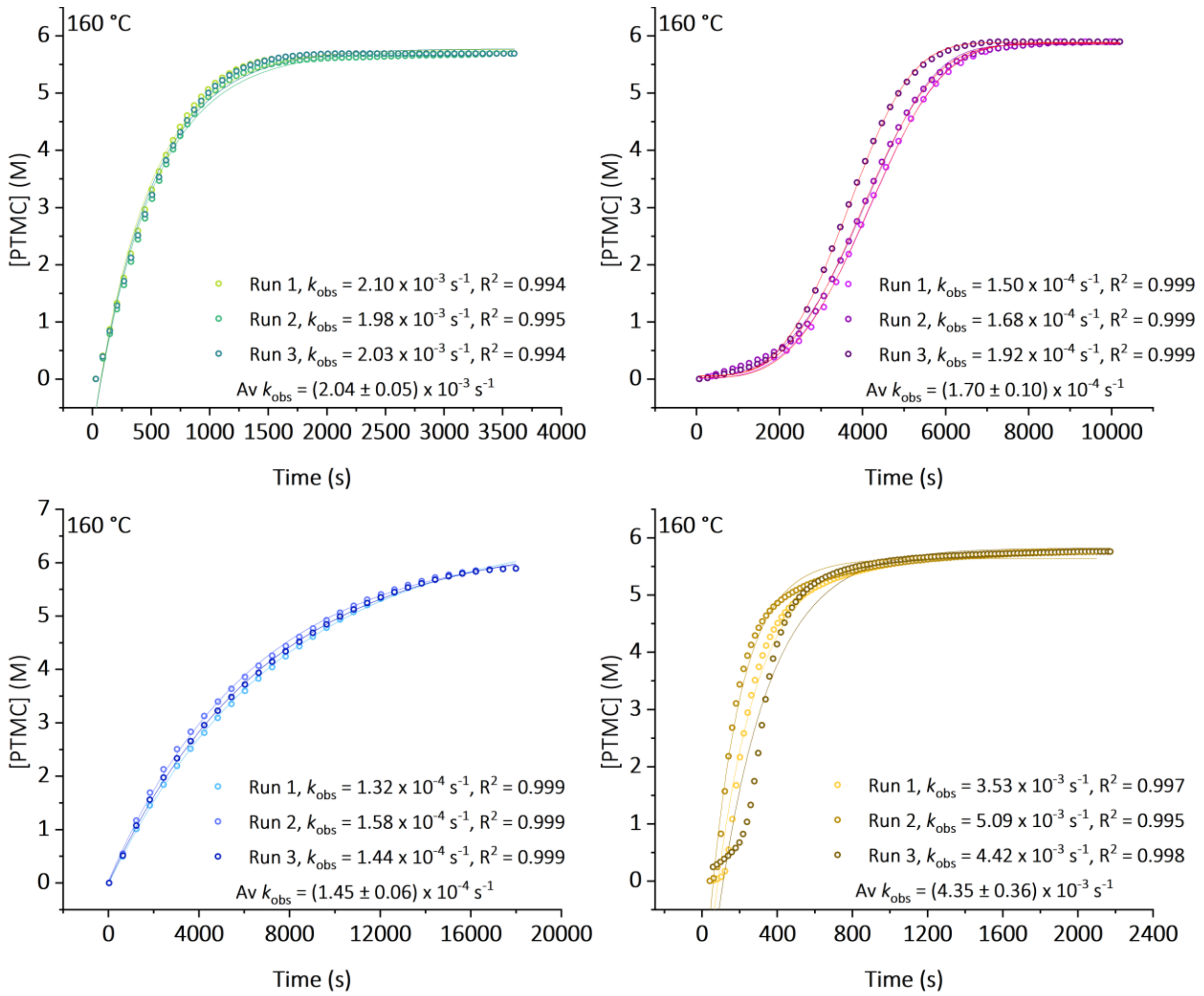

Fig. S15 Isothermal polymerization kinetic experiments, conducted on a DSC instrument, at 160 °C for the polymerization of TMC with the $Sn(Oct)_2$ catalyst (plot in green, top LHS), $Bi(Oct)_3$ catalyst (plot in purple, top RHS), $Zr(Oct)_4$ catalyst (plot in blue, bottom LHS), and $Mg(Oct)_2$ catalyst (plot in yellow, bottom RHS). For clarity, every n^{th} data point from the datasets is shown (n = 600, 1000, 6000, 8000 for Sn(II), Bi(III), Zr(IV), Mg(II) respectively). For the polymerization with $Bi(Oct)_3$ k_{obs} was extracted from a sigmoidal instead of exponential fit, of the form: [PTMC] = $A(1-\exp(-k_{obs}t))^s$, where s is the 'sigmoidal factor' (=3.3-3.5). Polymerization conditions: 1:10:1000 $M(Oct)_n$:BnOH:TMC, [TMC] = 6.25 M. In each case $[PTMC]_t = 1 - [TMC]_t$.

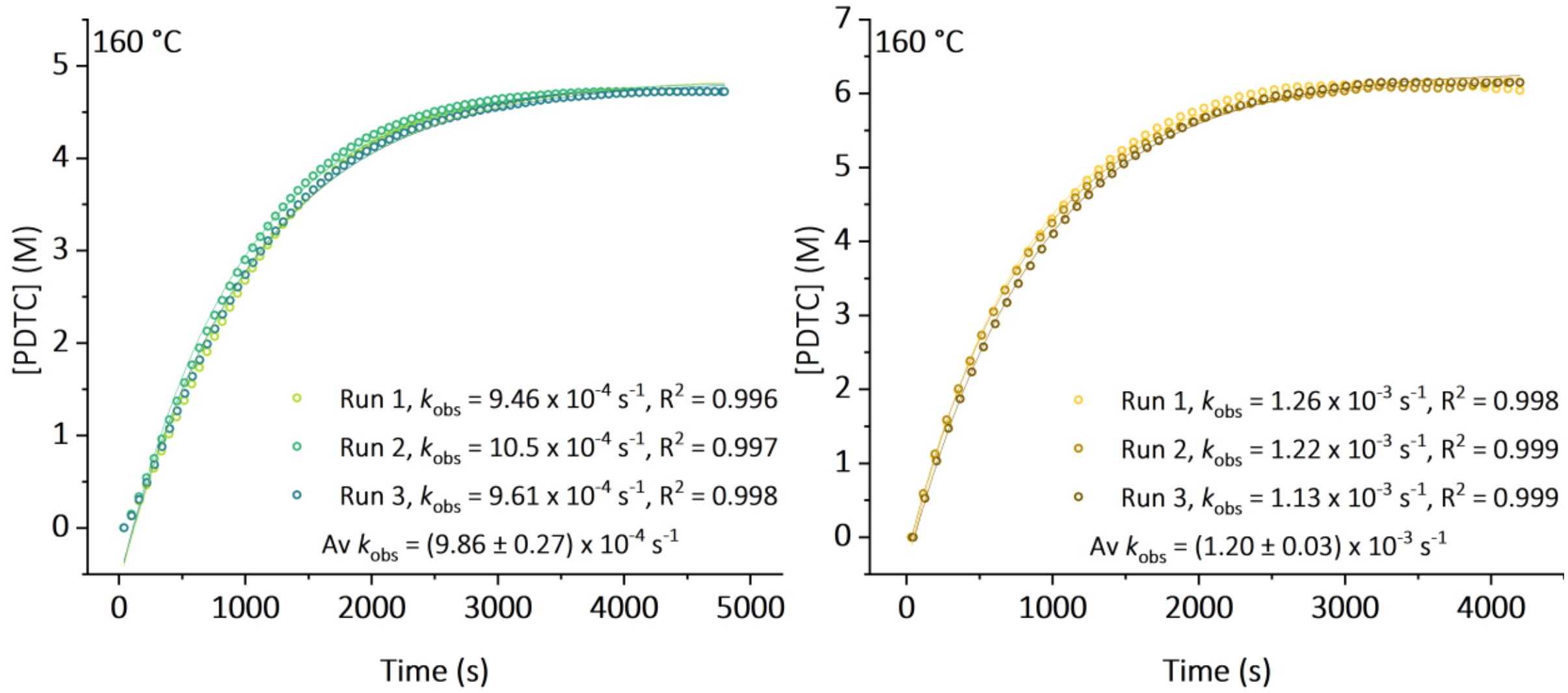

Fig. S16 Isothermal polymerization kinetic experiments, conducted on a DSC instrument, at 160 °C for the polymerization of DTC with the $Sn(Oct)_2$ catalyst (plot in green, LHS), and $Mg(Oct)_2$ catalyst (plot in yellow, RHS). For clarity, every nth data point from the datasets is shown (n = 600 and 8000 for Sn(II) and Mg(II) respectively). Polymerization conditions: 1:10:1000 $M(Oct)_n$:BnOH:DTC, [DTC] = 6.25 M. In each case $[PDTC]_t$ = 1 - $[DTC]_t$.

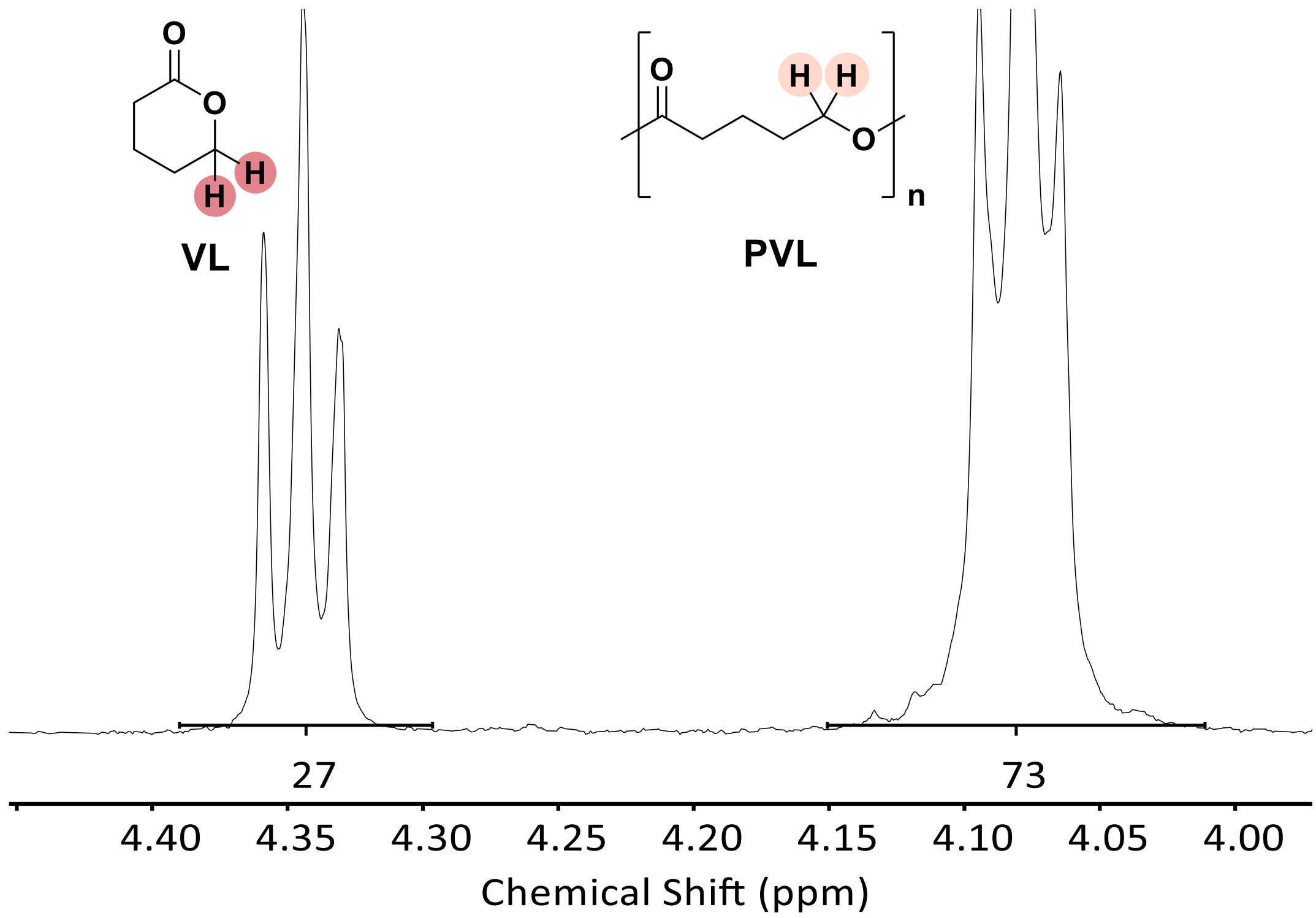

Fig. S17 Representative ^{1}H NMR spectrum (400 MHz, $CDCl_3$, 298 K) for calculation of conversion of VL to PVL. Polymerization conditions: 1:10:1000 $Zn(Oct)_2$:BnOH:VL, [VL] = 6.25 M, at 200 °C.

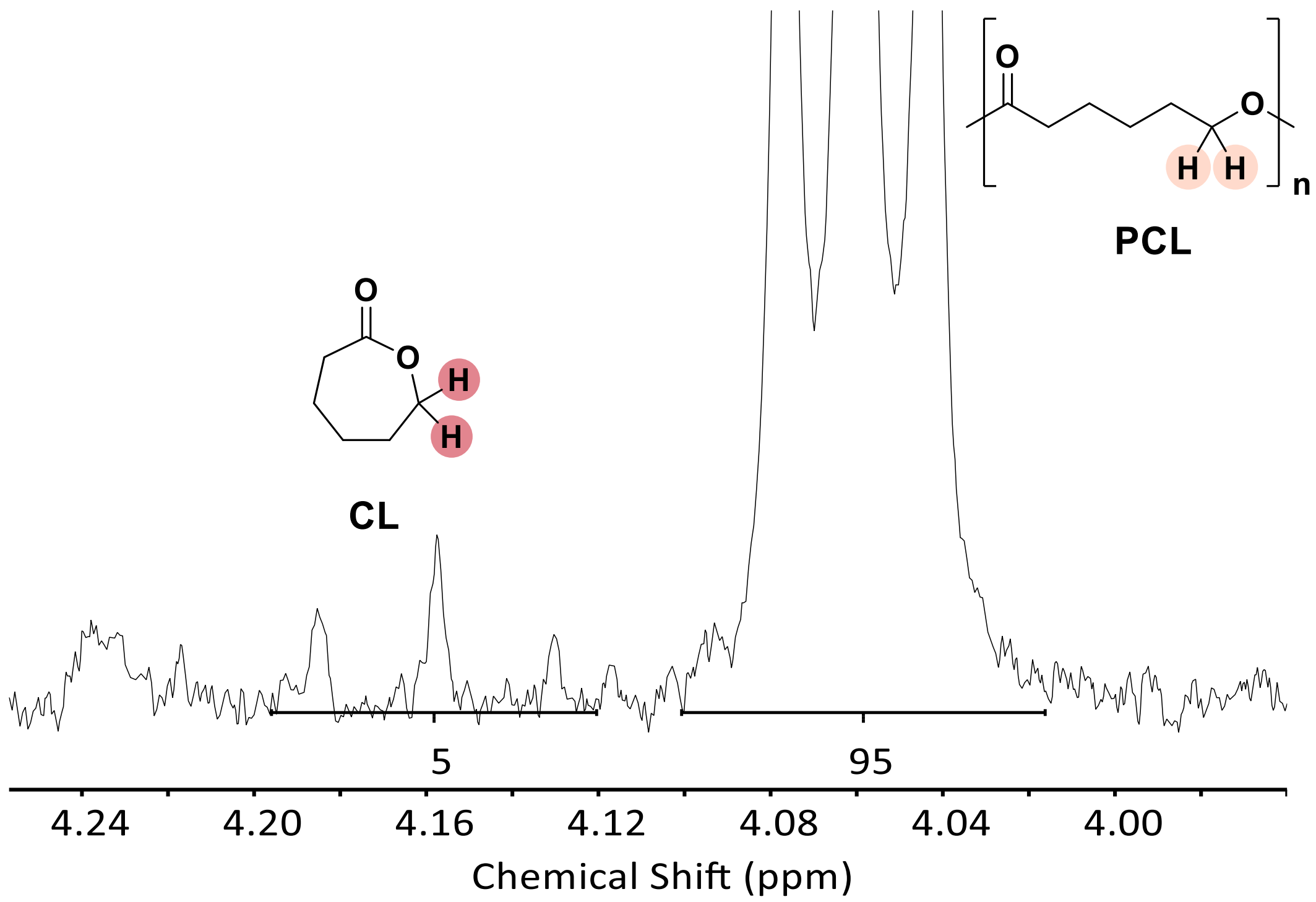

Fig. S18 Representative ^{1}H NMR spectrum (400 MHz, $CDCl_3$, 298 K) for calculation of conversion of CL to PCL. Polymerization conditions: 1:10:1000 $Zn(Oct)_2$:BnOH:CL, [CL] = 6.25 M, at 200 °C.

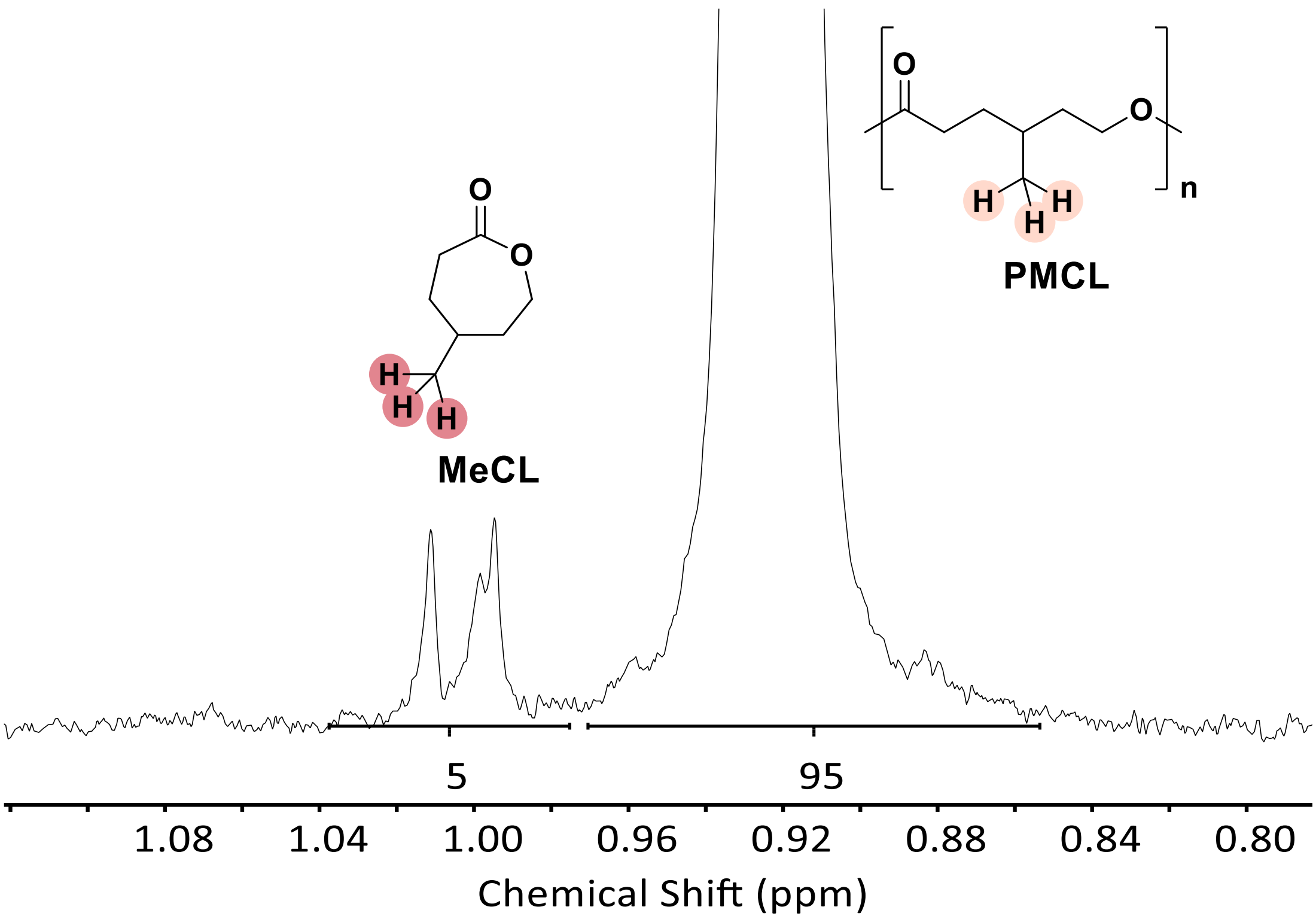

Fig. S19 Representative ^{1}H NMR spectrum (400 MHz, $CDCl_3$, 298 K) for calculation of conversion of MeCL to PMCL. Polymerization conditions: 1:10:1000 $Zn(Oct)_2$:BnOH:MeCL, [MeCL] = 6.25 M, at 200 °C.

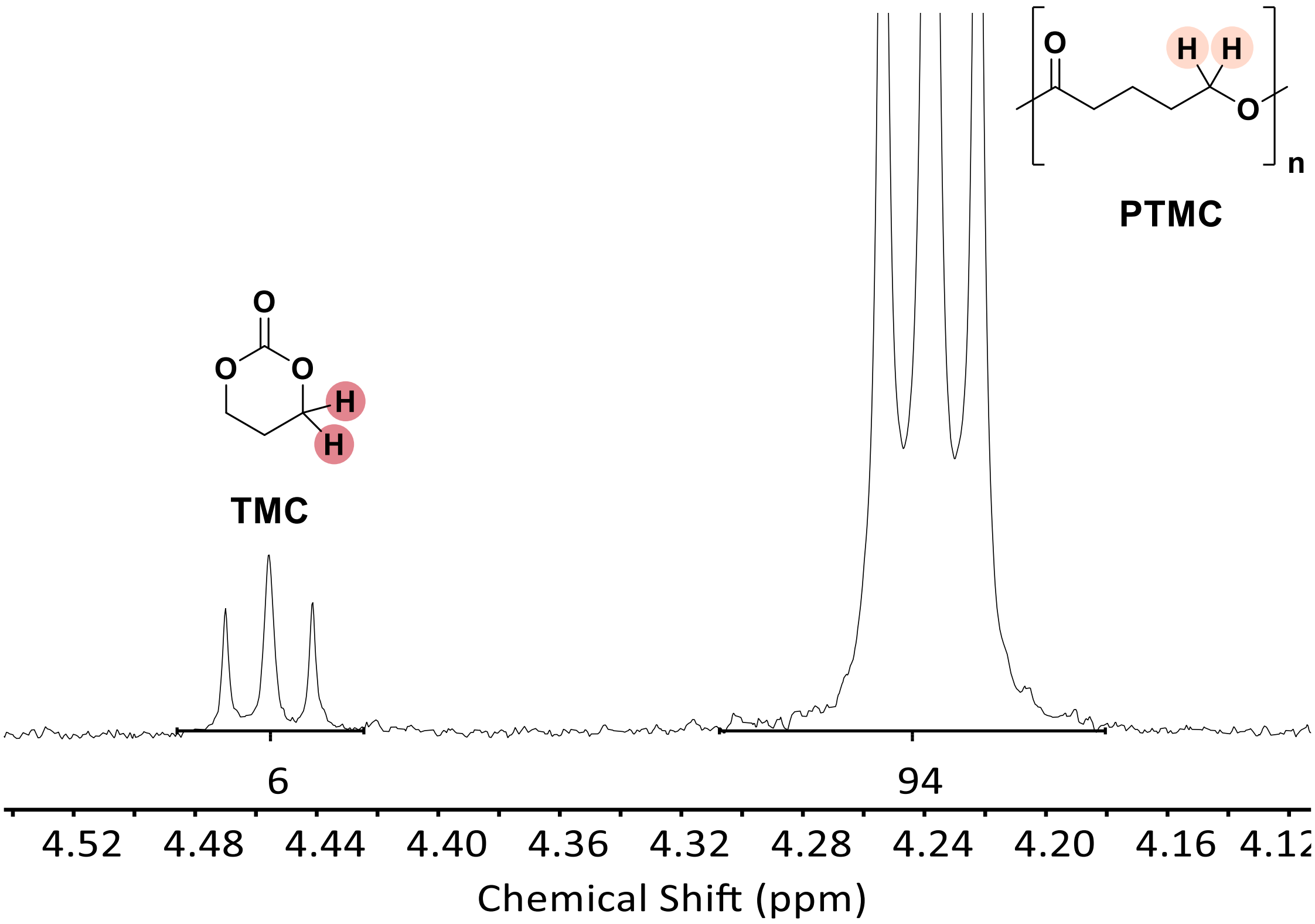

Fig. S20 Representative ^{1}H NMR spectrum (400 MHz, $CDCl_3$, 298 K) for calculation of conversion of TMC to PTMC. Polymerization conditions: 1:10:1000 $Zn(Oct)_2$:BnOH:TMC, [TMC] = 6.25 M, at 180 °C.

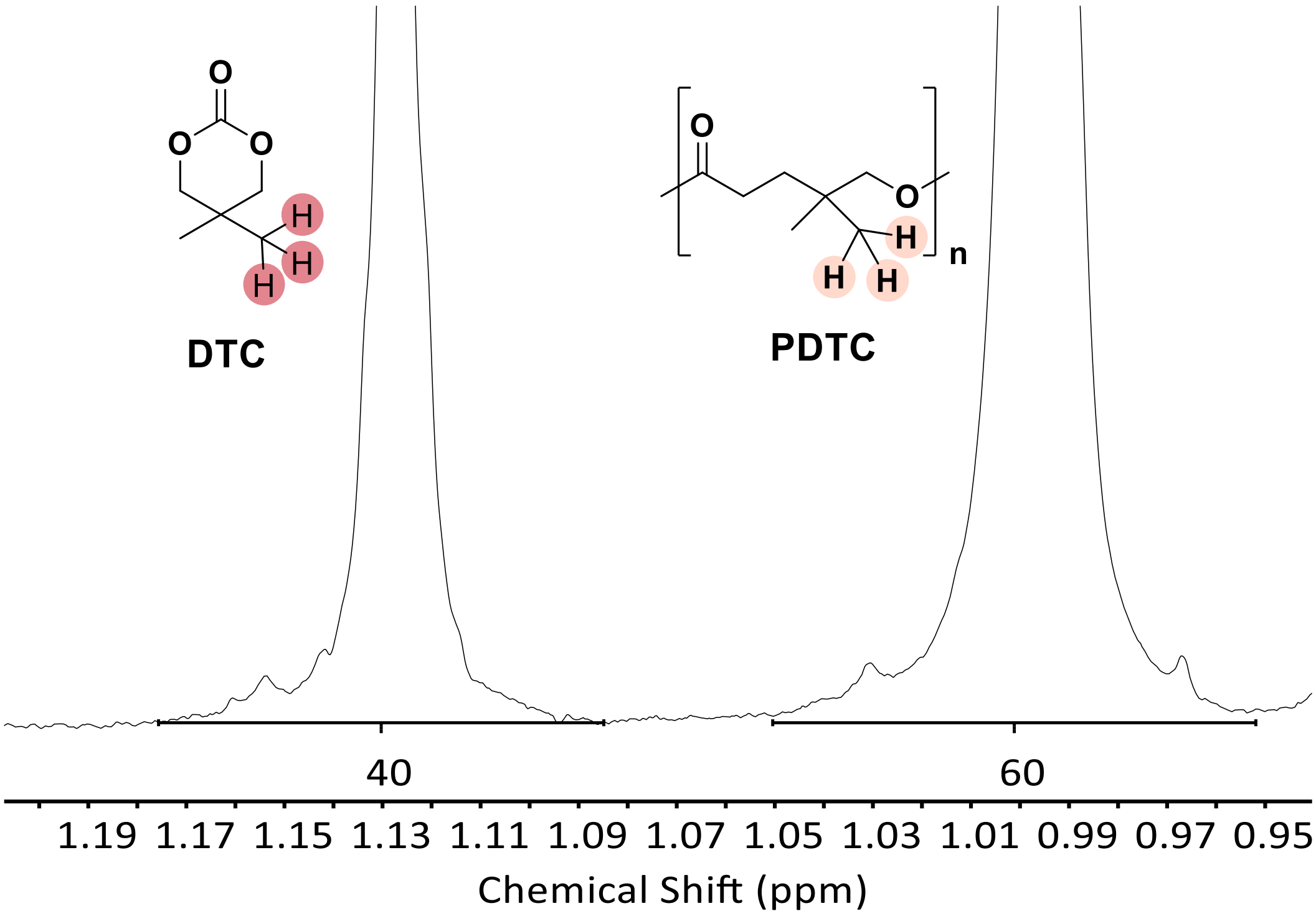

Fig. S21 Representative ^{1}H NMR spectrum (400 MHz, $CDCl_3$, 298 K) for calculation of conversion of DTC to PDTC. Polymerization conditions: 1:10:1000 $Zn(Oct)_2$:BnOH:DTC, [DTC] = 6.25 M, at 200 °C.

Table S1 Polymerization data for polymerization of VL. Polymerization conditions: 1:10:1000 $M(Oct)_n$:BnOH:VL, [VL] = 6.25 M.

Catalyst	Temperature (°C)	k_{obs} / 10^{-3} (s^{-1})[a]	Av. k_{obs} / 10^{-3} (s^{-1})[a]	Conversion to polymer[b]	Av. Conversion to polymer	M_n [Đ] (kg mol^{-1})[c]
$Zn(Oct)_2$	160	1.08		0.79		
		1.11	1.09 ± 0.01	0.83	0.81 ± 0.01	12.1 [1.95]
		1.07		0.79		
	170	1.59		0.76		
		1.55	1.52 ± 0.04	0.79	0.80 ± 0.02	17.0 [1.96]
		1.42		0.85		
	180	1.72		0.72		
		1.84	1.69 ± 0.08	0.74	0.75 ± 0.02	12.5 [1.93]
		1.52		0.79		
	190	3.08		0.79		
		2.89	2.86 ± 0.11	0.68	0.75 ± 0.03	15.9 [1.74]
		2.60		0.77		
	200	3.45		0.75		
		3.60	3.54 ± 0.04	0.73	0.70 ± 0.03	13.9 [1.84]
		3.58		0.63		
$Sn(Oct)_2$	160	1.55		0.83		
		1.17	1.18 ± 0.17	0.78	0.79 ± 0.01	22.9 [1.49]
		0.83		0.77		
	170	1.56		0.81		
		1.42	1.48 ± 0.03	0.79	0.79 ± 0.01	20.7 [1.97]
		1.46		0.78		
	180	3.04		0.78		
		2.23	2.95 ± 0.32	0.76	0.76 ± 0.01	28.1 [1.23]
		3.59		0.74		
	190	3.61		0.77		
		3.31	3.32 ± 0.14	0.71	0.74 ± 0.02	31.6 [1.16]
		3.03		0.75		
	200	4.60		0.74		
		4.72	4.79 ± 0.11	0.73	0.72 ± 0.02	20.9 [1.67]
		5.05		0.68		
$Bi(Oct)_3$	160	0.14		0.77		
		0.14	0.13 ± 0.007	0.77	0.77 ± 0.01	9.2 [1.52]
		0.12		0.76		
	170	0.16		0.75		
		0.17	0.17 ± 0.003	0.76	0.76 ± 0.01	9.7 [1.66]
		0.17		0.76		
	180	0.23		0.74		
		0.15	0.20 ± 0.02	0.73	0.74 ± 0.01	11.8 [1.78]
		0.20		0.74		
	190	0.37		0.72		
		0.26	0.32 ± 0.03	0.72	0.72 ± 0.01	12.0 [1.64]
		0.32		0.72		
	200	0.38		0.60		
		0.46	0.43 ± 0.02	0.66	0.62 ± 0.02	10.2 [1.76]
		0.44		0.60		
$Zr(Oct)_4$	160	0.10		0.79		
		0.11	0.11 ± 0.004	0.79	0.80 ± 0.01	15.1 [1.55]
		0.12		0.81		
	170	0.15		0.77		
		0.16	0.15 ± 0.008	0.77	0.77 ± 0.01	14.5 [1.59]

		0.13		0.76		
	180	0.32<br>0.33<br>0.32	0.32 ± 0.002	0.76<br>0.73<br>0.74	0.74 ± 0.01	12.2 [1.63]
	190	0.55<br>0.55<br>0.56	0.55 ± 0.004	0.73<br>0.73<br>0.72	0.73 ± 0.01	14.1 [1.57]
	200	0.78<br>0.89<br>0.85	0.84 ± 0.046	0.71<br>0.71<br>0.57	0.66 ± 0.04	15.1 [1.53]
$Mg(Oct)_2$	160	0.04<br>0.07<br>0.07	0.06 ± 0.007	0.78<br>0.99<br>0.66	0.81 ± 0.08	20.2 [1.54]
	170	0.11<br>0.09<br>0.08	0.09 ± 0.008	0.80<br>0.75<br>0.76	0.77 ± 0.01	20.0 [1.43]
	180	0.08<br>0.14<br>0.10	0.11 ± 0.01	0.75<br>0.72<br>0.73	0.73 ± 0.01	23.0 [1.40]
	190	0.16<br>0.12<br>0.13	0.14 ± 0.01	0.73<br>0.73<br>0.72	0.73 ± 0.01	26.2 [1.30]
	200	0.19<br>0.15<br>0.17	0.17 ± 0.009	0.73<br>0.69<br>0.73	0.72 ± 0.01	18.6 [1.55]

[a]k_{obs} determined from the DSC isotherm at the temperature indicated: integration of the DSC normalized heat flow *vs*. time and normalizing to polymer conversion produced exponential fits, where the k_{obs} is taken from the exponential fitting of each line ($y = A - Be^{-k_{obs}x}$), with the average k_{obs} taken as the mean of the three repeats and error determined from the standard deviation of the mean of the three repeats (Fig. S3, and S8-S11). [b]Conversion to polymer was calculated from ^1H NMR integrals of the VL and PVL (Fig. S17). [c]Determined by GPC in THF using narrow dispersity polystyrene standards.

Table S2 Polymerization data for polymerization of CL. Polymerization conditions: 1:10:1000 $M(Oct)_n$:BnOH:CL, [CL] = 6.25 M.

Catalyst	Temperature (°C)	k_{obs} / 10^{-4} (s^{-1})[a]	Av. k_{obs} / 10^{-4} (s^{-1})[a]	Conversion to polymer[b]	Av. Conversion to polymer	M_n [Đ] (kg mol^{-1})[c]
$Zn(Oct)_2$	160	6.52<br>6.06<br>5.58	6.05 ± 0.22	0.98<br>1.00<br>0.98	0.99 ± 0.01	24.9 [1.52]
	170	9.91<br>8.00<br>9.23	9.05 ± 0.46	0.98<br>1.00<br>0.98	0.99 ± 0.01	30.3 [1.63]
	180	9.84<br>13.6<br>14.8	12.7 ± 1.21	0.98<br>0.99<br>0.98	0.98 ± 0.01	26.1 [1.41]
	190	18.1<br>17.4<br>20.0	18.5 ± 0.63	0.97<br>0.99<br>0.95	0.97 ± 0.01	34.7 [1.52]
	200	34.8<br>35.0<br>30.4	33.4 ± 1.23	0.95<br>0.98<br>0.95	0.96 ± 0.01	26.2 [1.67]
$Sn(Oct)_2$	140	10.9<br>11.5<br>10.6	11.0 ± 0.22	0.99<br>1.00<br>0.97	0.99 ± 0.02	28.6 [1.46]
	150	27.8<br>23.2<br>23.9	25.0 ± 1.17	0.99<br>0.98<br>0.99	0.99 ± 0.01	26.9 [1.54]
	160	39.4<br>28.7<br>41.4	36.5 ± 3.22	0.95<br>0.95<br>0.99	0.97 ± 0.01	26.2 [1.64]
	170	68.4<br>67.2<br>76.7	70.8 ± 2.44	0.99<br>0.99<br>0.99	0.99 ± 0.01	28.6 [1.52]
	180	123.0<br>196.3<br>160.1	159.7 ± 17.2	0.98<br>0.98<br>0.98	0.98 ± 0.01	27.1 [1.54]
$Bi(Oct)_3$	160	3.33<br>3.30<br>3.61	3.41 ± 0.08	0.92<br>0.90<br>0.92	0.91 ± 0.01	22.3 [1.49]
$Zr(Oct)_4$	160	2.25<br>2.55<br>1.52	2.11 ± 0.25	0.95<br>0.98<br>0.90	0.94 ± 0.02	26.8 [1.72]
$Mg(Oct)_2$	160	0.49<br>0.50<br>0.48	0.49 ± 0.006	0.99<br>1.00<br>1.00	0.99 ± 0.01	39.1 [1.42]

[a]k_{obs} determined from the DSC isotherm at the temperature indicated: integration of the DSC normalized heat flow *vs*. time and normalizing to polymer conversion produced exponential fits, where the k_{obs} is taken from the exponential fitting of each line ($y = A - Be^{-k_{obs}x}$), with the average k_{obs} taken as the mean of the three repeats and error determined from the standard deviation of the mean of the three repeats (Fig. S4, S12 and S13). [b]Conversion to polymer was calculated from ^{1}H NMR integrals of the CL and PCL (Fig. S18). [c]Determined by GPC in THF using narrow dispersity polystyrene standards.

Table S3 Polymerization data for polymerization of MeCL. Polymerization conditions: 1:10:1000 M(Oct)$_n$:BnOH:MeCL, [MeCL] = 6.25 M.

Catalyst	Temperature (°C)	k_{obs} / 10^{-4} (s^{-1})[a]	Av. k_{obs} / 10^{-4} (s^{-1})[a]	Conversion to polymer[b]	Av. Conversion to polymer	M_n [Đ] (kg mol^{-1})[c]
Zn(Oct)$_2$	160	3.23<br>3.08<br>3.43	3.25 ± 0.08	0.95<br>0.96<br>0.96	0.96 ± 0.01	25.2 [1.54]
	170	4.57<br>4.52<br>4.60	4.56 ± 0.02	0.95<br>0.95<br>0.95	0.95 ± 0.01	18.6 [1.60]
	180	5.11<br>5.06<br>7.89	6.02 ± 0.76	0.93<br>0.90<br>0.92	0.911 ± 0.01	25.2 [1.36]
	190	7.71<br>6.15<br>7.25	7.04 ± 0.38	0.90<br>0.90<br>0.88	0.90 ± 0.01	24.9 [1.52]
	200	9.36<br>8.97<br>9.37	9.23 ± 0.11	0.86<br>0.85<br>0.85	0.85 ± 0.01	26.7 [1.53]
Sn(Oct)$_2$	160	16.9<br>9.17<br>7.93	11.3 ± 2.29	0.97<br>0.93<br>0.91	0.94 ± 0.02	23.4 [1.65]
Bi(Oct)$_3$	160	2.87<br>2.84<br>3.21	2.97 ± 0.10	0.90<br>0.90<br>0.90	0.90 ± 0.01	21.0 [1.51]
Zr(Oct)$_4$	160	2.55<br>2.53<br>1.49	2.19 ± 0.29	0.82<br>0.96<br>0.97	0.92 ± 0.04	28.0 [1.56]
Mg(Oct)$_2$	160	0.47<br>0.51<br>0.46	0.48 ± 0.01	0.92<br>0.92<br>0.92	0.92 ± 0.01	15.2 [1.65]

[a]k_{obs} determined from the DSC isotherm at the temperature indicated: integration of the DSC normalized heat flow *vs.* time and normalizing to polymer conversion produced exponential fits, where the k_{obs} is taken from the exponential fitting of each line ($y = A - Be^{-k_{obs}x}$), with the average k_{obs} taken as the mean of the three repeats and error determined from the standard deviation of the mean of the three repeats (Fig. S5 and S14). [b]Conversion to polymer was calculated from ^{1}H NMR integrals of the MeCL and PMCL (Fig. S19). [c]Determined by GPC in THF using narrow dispersity polystyrene standards.

Table S4 Polymerization data for polymerization of TMC. Polymerization conditions: 1:10:1000 $M(Oct)_n$:BnOH:TMC, [TMC] = 6.25 M.

Catalyst	Temperature (°C)	k_{obs} / 10^{-3} (s^{-1})[a]	Av. k_{obs} / 10^{-3} (s^{-1})[a]	Conversion to polymer[b]	Av. Conversion to polymer	M_n [Đ] (kg mol^{-1})[c]
$Zn(Oct)_2$	140	3.33<br>3.19<br>3.92	3.48 ± 0.18	0.98<br>0.98<br>0.97	0.98 ± 0.01	24.6 [1.20]
	150	5.54<br>5.67<br>6.05	5.75 ± 0.12	0.98<br>0.96<br>0.97	0.97 ± 0.01	22.0 [1.39]
	160	7.34<br>10.7<br>8.77	8.94 ± 0.80	0.96<br>0.95<br>0.94	0.96 ± 0.01	22.6 [1.47]
	170	13.9<br>11.6<br>9.81	11.8 ± 0.97	0.96<br>0.94<br>0.95	0.94 ± 0.01	22.5 [1.38]
	180	15.5<br>16.3<br>17.1	16.3 ± 0.40	0.94<br>0.94<br>0.94	0.94 ± 0.01	28.8 [1.22]
$Sn(Oct)_2$	160	2.10<br>1.98<br>2.03	2.04 ± 0.03	0.95<br>0.94<br>0.95	0.94 ± 0.01	22.9 [1.38]
$Bi(Oct)_3$	160	0.15<br>0.17<br>0.19	0.17 ± 0.01	0.97<br>0.94<br>0.94	0.95 ± 0.01	19.3 [1.55]
$Zr(Oct)_4$	160	0.13<br>0.16<br>0.14	0.15 ± 0.01	0.95<br>0.95<br>0.95	0.95 ± 0.01	21.2 [1.47]
$Mg(Oct)_2$	160	3.53<br>5.09<br>4.42	4.35 ± 0.37	0.89<br>0.92<br>0.95	0.92 ± 0.01	38.4 [1.17]

[a]k_{obs} determined from the DSC isotherm at the temperature indicated: integration of the DSC normalized heat flow *vs.* time and normalizing to polymer conversion produced exponential fits, where the k_{obs} is taken from the exponential fitting of each line ($y = A - Be^{-k_{obs}x}$), with the average k_{obs} taken as the mean of the three repeats and error determined from the standard deviation of the mean of the three repeats (Fig. S6 and S15). [b]Conversion to polymer was calculated from ^{1}H NMR integrals of the TMC and PTMC (Fig. S20). For the polymerization with $Bi(Oct)_3$, k_{obs} was extracted from a sigmoidal instead of exponential fit, of the form: $[PTMC] = A(1-\exp(-k_{obs}t))^s$, where s is the 'sigmoidal factor' (=3.3-3.5). [c]Determined by GPC in THF using narrow dispersity polystyrene standards.

Table S5 Polymerization data for polymerization of DTC. Polymerization conditions: 1:10:1000 $M(Oct)_n$:BnOH:DTC, [DTC] = 6.25 M.

Catalyst	Temperature (°C)	k_{obs} / 10^{-3} (s^{-1})[a]	Av. k_{obs} / 10^{-3} (s^{-1})[a]	Conversion to polymer[b]	Av. Conversion to polymer	M_n [Đ] (kg mol^{-1})[c]
$Zn(Oct)_2$	160	4.09		0.68		
		3.95	4.13 ± 0.09	0.85	0.80 ± 0.05	13.7 [1.47]
		4.34		0.86		
	170	4.67		0.78		
		4.79	5.15 ± 0.34	0.82	0.76 ± 0.03	15.2 [1.22]
		6.00		0.69		
	180	7.11		0.75		
		5.75	5.98 ± 0.49	0.70	0.73 ± 0.01	18.4 [1.24]
		5.07		0.72		
	190	7.99		0.62		
		7.93	7.41 ± 0.45	0.64	0.63 ± 0.01	12.5 [1.48]
		6.31		0.62		
	200	11.2		0.60		
		10.8	10.8 ± 0.21	0.62	0.60 ± 0.01	9.5 [1.32]
		10.3		0.57		
$Sn(Oct)_2$	160	0.95		0.75		
		1.05	0.99 ± 0.03	0.76	0.75 ± 0.01	23.2 [1.62]
		0.96		0.75		
$Bi(Oct)_3$	160	-	-	0.03		-
				0.04	0.03 ± 0.01	
				0.02		
$Zr(Oct)_4$	160	-	-	0.01		-
				0.01	0.01	
				0.01		
$Mg(Oct)_2$	160	1.26		0.98		
		1.22	1.20 ± 0.03	0.99	0.98 ± 0.01	7.9 [1.22]
		1.13		0.98		

[a]k_{obs} determined from the DSC isotherm at the temperature indicated: integration of the DSC normalized heat flow *vs.* time and normalizing to polymer conversion produced exponential fits, where the k_{obs} is taken from the exponential fitting of each line ($y = A - Be^{-k_{obs}x}$), with the average k_{obs} taken as the mean of the three repeats and error determined from the standard deviation of the mean of the three repeats (Fig. S7 and S16). [b]Conversion to polymer was calculated from ^1H NMR integrals of the DTC and PDTC (Fig. S21). [c]Determined by GPC in THF using narrow dispersity polystyrene standards.

To determine the order in $Zn(Oct)_2$ catalyst for the 6- and 7- membered lactones and cyclic carbonates, a series of polymerizations with varying catalyst loading (x) were completed using the TMC monomer at 100 °C (Table S6). Each reaction was run in triplicate, with an average k_{obs} determined as the average of three runs, and errors determined from the standard deviation of the mean of the three repeats. An example of this for the polymerization conducted using 20 equivalents of catalyst is shown in Fig. S22.

Table S6 k_{obs} determined at incrementally increasing catalyst loadings for the polymerization of TMC using the $Zn(Oct)_2$ catalyst and BnOH chain-transfer agent at 100 °C.

Eq $Zn(Oct)_2$	k_{obs} /10^{-3} (s^{-1})
1	0.78 ± 0.02
10	6.19 ± 0.51
20	16.3 ± 0.52
25	20.6 ± 1.04

Polymerization conditions: x:10x:1000 $Zn(Oct)_2$:BnOH:TMC, 100 °C, [TMC] = 6.25 M

Plotting ln(k_{obs}) *vs.* ln([$Zn(Oct)_2$]) yields a straight line with a gradient of 1.03, indicating a first-order dependence on $Zn(Oct)_2$ catalyst (Fig. S24). With this result, the same order was assumed for the remainder of the series of investigated monomers.

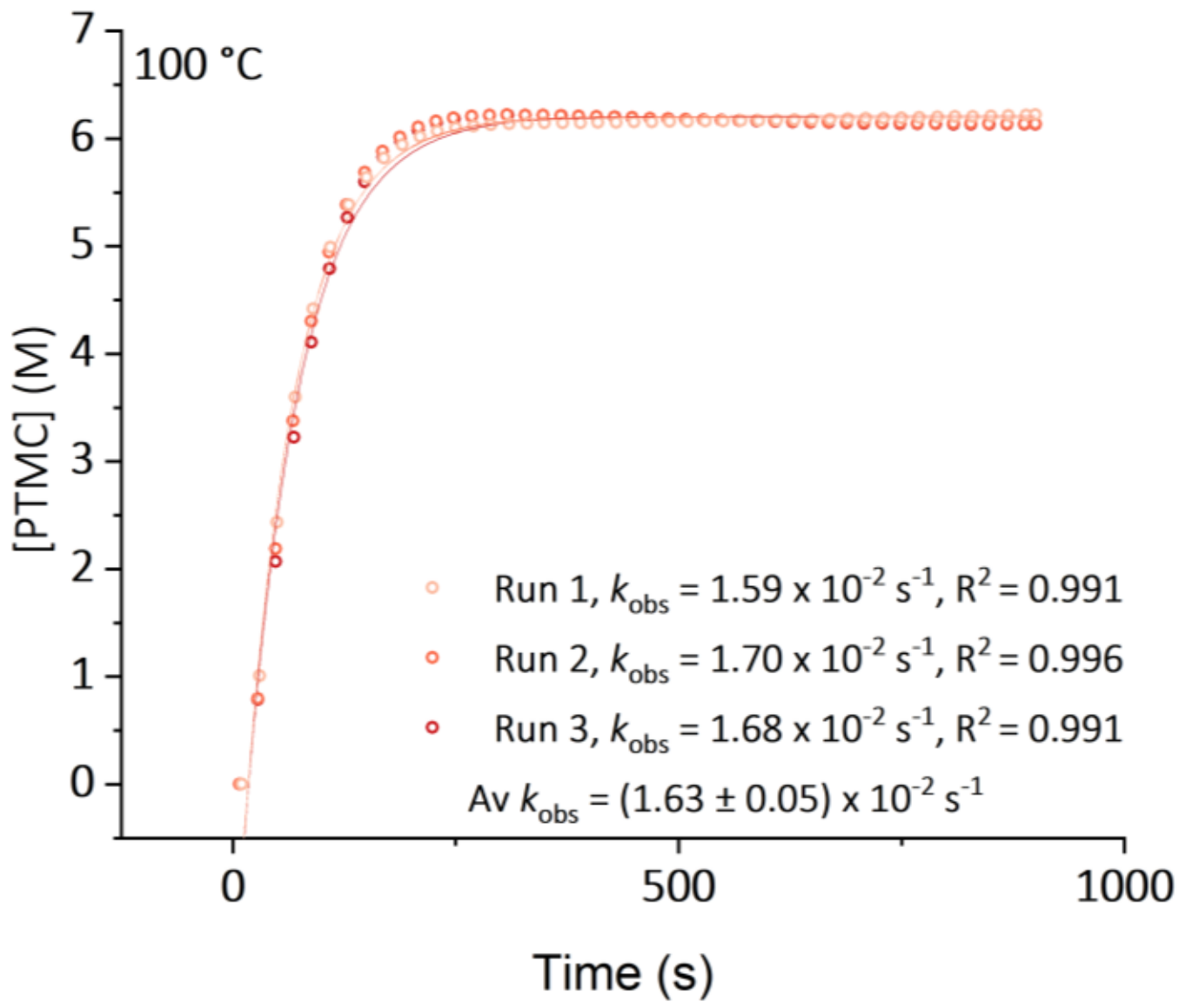

Fig. S22 Plots of [PTMC] *vs*. time for isotherms conducted at 100 °C. k_{obs} is taken from the exponential fitting of each line ($y = A - Be^{-k_{obs}x}$), with the average k_{obs} taken as the mean of the three repeats and error determined from the standard deviation of the mean of the three repeats. Polymerization conditions: 20:200:1000 $Zn(Oct)_2$:BnOH:TMC.

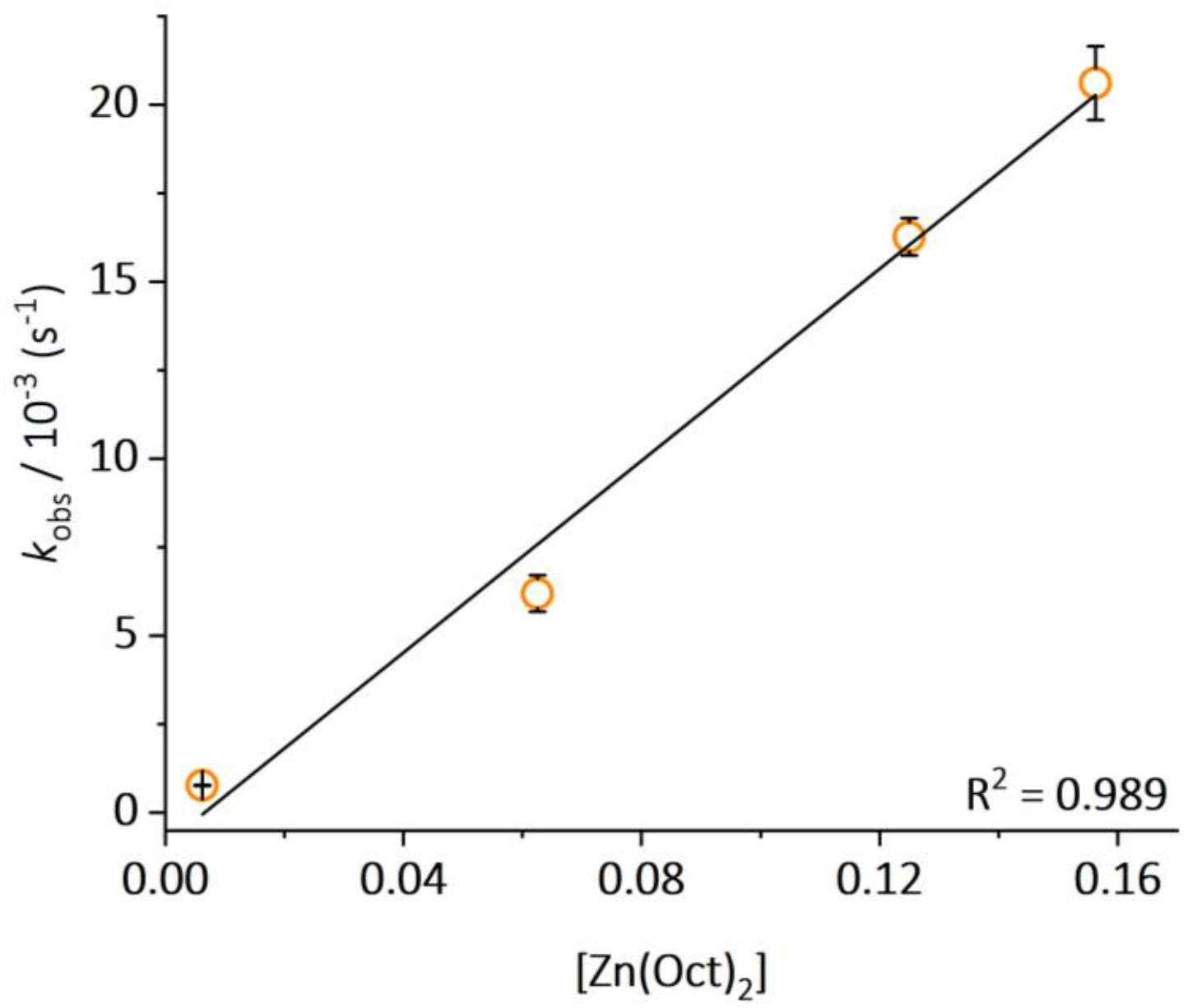

Fig. S23 Plot of k_{obs} *vs*. [Zn(Oct)$_2$] for isotherms conducted at 100 °C with polymerization conditions: x:10x:1000 Zn(Oct)$_2$:BnOH:TMC where x= 1, 10, 20, and 25, [TMC] = 6.25 M.

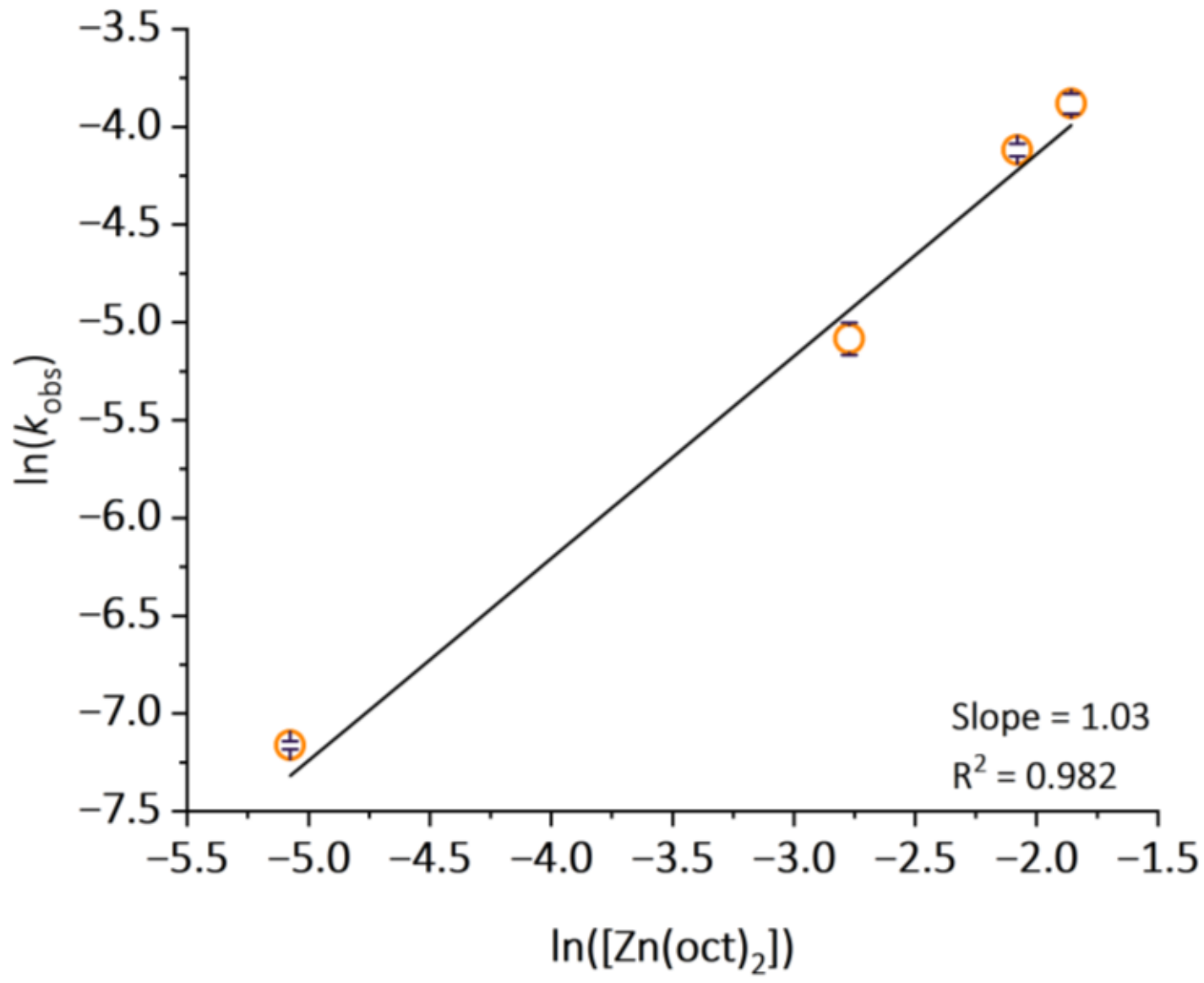

Fig. S24 Plot of ln(k_{obs}) *vs*. ln([Zn(Oct)$_2$]) for isotherms conducted at 100 °C with polymerization conditions: x:10x:1000 Zn(Oct)$_2$:BnOH:TMC where x= 1, 10, 20, and 25, [TMC] = 6.25 M.

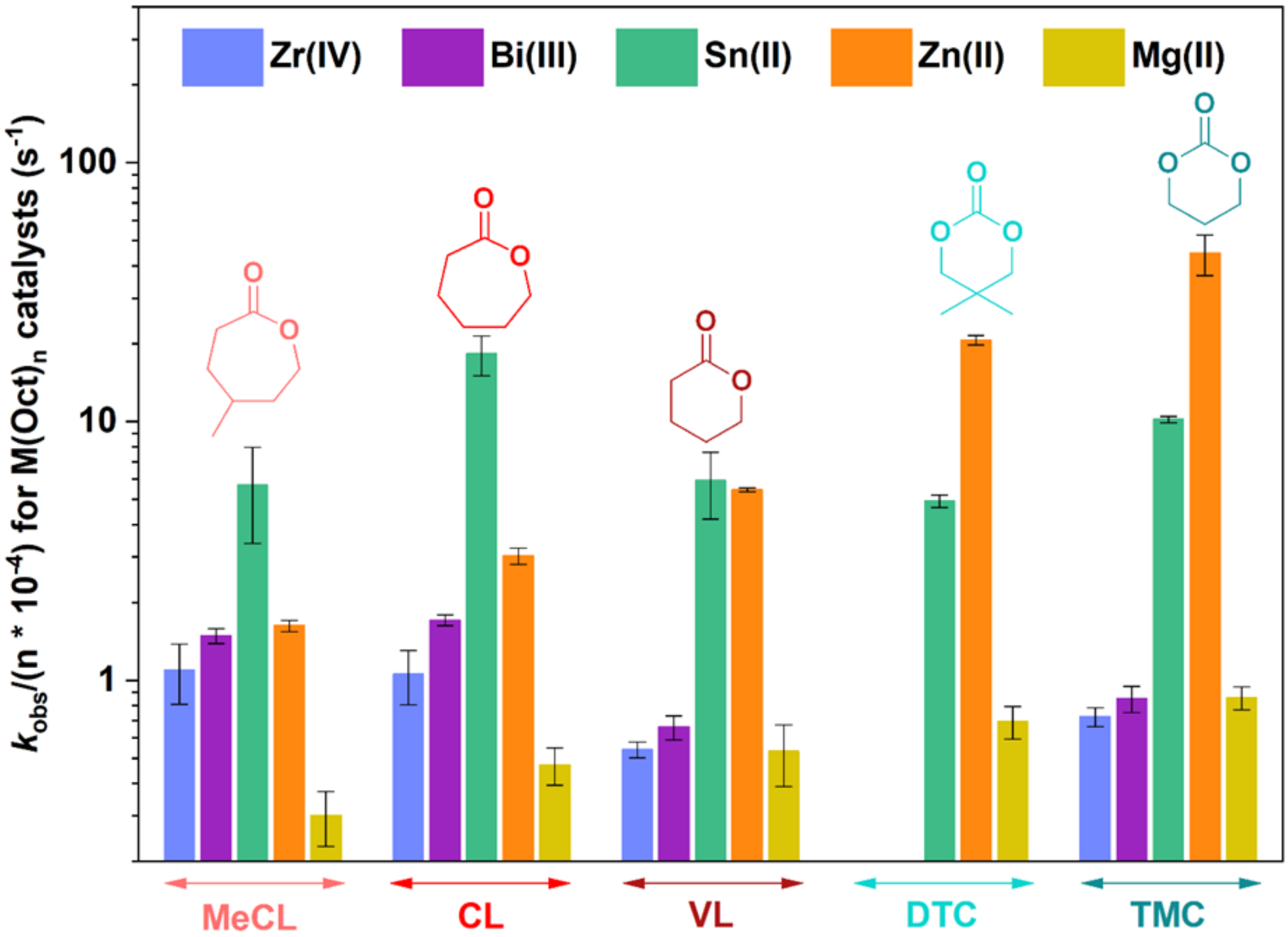

Fig. S25 Bar chart of polymerization rate constant, k_{obs}, normalized for the number of octoate chains per metal center (n), for each monomer tested with each $M(Oct)_n$ catalyst. Polymerization conditions: 1:10:1000 $M(Oct)_n$:BnOH:monomer, [monomer] = 6.25 M in 1,2-dimethoxybenzene, 160 ^{o}C. Catalysts $Bi(Oct)_3$ and $Zr(Oct)_4$ showed negligible activity for DTC polymerization under these conditions.

Table S7 Polymerization data for polymerization of VL. Polymerization conditions: 1:1:1000 $M(Oct)_n$:BnOH:VL, [VL] = 6.25 M, 160 ^oC.

Catalyst	k_{obs} / 10^{-4} (s^{-1})[a]	Av. k_{obs} / 10^{-4} (s^{-1})[a]	TOF (h^{-1})[b]	Av. TOF(h^{-1})[b]	Conversion to polymer[c]	Av. Conversion to polymer[c]	M_n [Đ] (kg mol^{-1})[d]
$Sn(Oct)_2$	6.16		626		0.73		
	8.14	7.62 ± 0.60	531	589 ± 24	0.62	0.68 ± 0.03	63.3 [1.65]
	8.56		609		0.71		
$Zn(Oct)_2$	5.23		609		0.71		
	5.14	5.38 ± 0.16	591	551 ± 40	0.69	0.65 ± 0.05	70.4 [1.43]
	5.77		454		0.53		
$Bi(Oct)_3$	3.16		168		0.84		
	2.93	3.10 ± 0.07	166	164 ± 3	0.83	0.82 ± 0.01	73.7 [1.46]
	3.21		158		0.79		
$Zr(Oct)_4$			14		0.23		
	-	-	15	17 ± 2	0.25	0.28 ± 0.03	26.2 [1.29]
			22		0.36		
$Mg(Oct)_2$			4		0.06		
	-	-	4	4 ± 0.2	0.06	0.06 ± 0.003	-
			3		0.06		

[a]k_{obs} determined from the DSC isotherm at the temperature indicated: integration of the DSC normalized heat flow *vs*. time and normalizing to polymer conversion produced exponential fits, where the k_{obs} is taken from the exponential fitting of each line ($y = A - Be^{-k_{obs}x}$), with the average k_{obs} taken as the mean of the three repeats and error determined from the standard deviation of the mean of the three repeats (Fig. S26). [b]Where k_{obs} determination from DSC was not possible, TOF was determined from ((NMR calculated conversion to polymer) x monomer loading)/time, at 160 ^oC. [c]Conversion to polymer was calculated from ^1H NMR integrals of the VL and PVL (Fig. S17). [d]Determined by GPC in THF using narrow dispersity polystyrene standards.

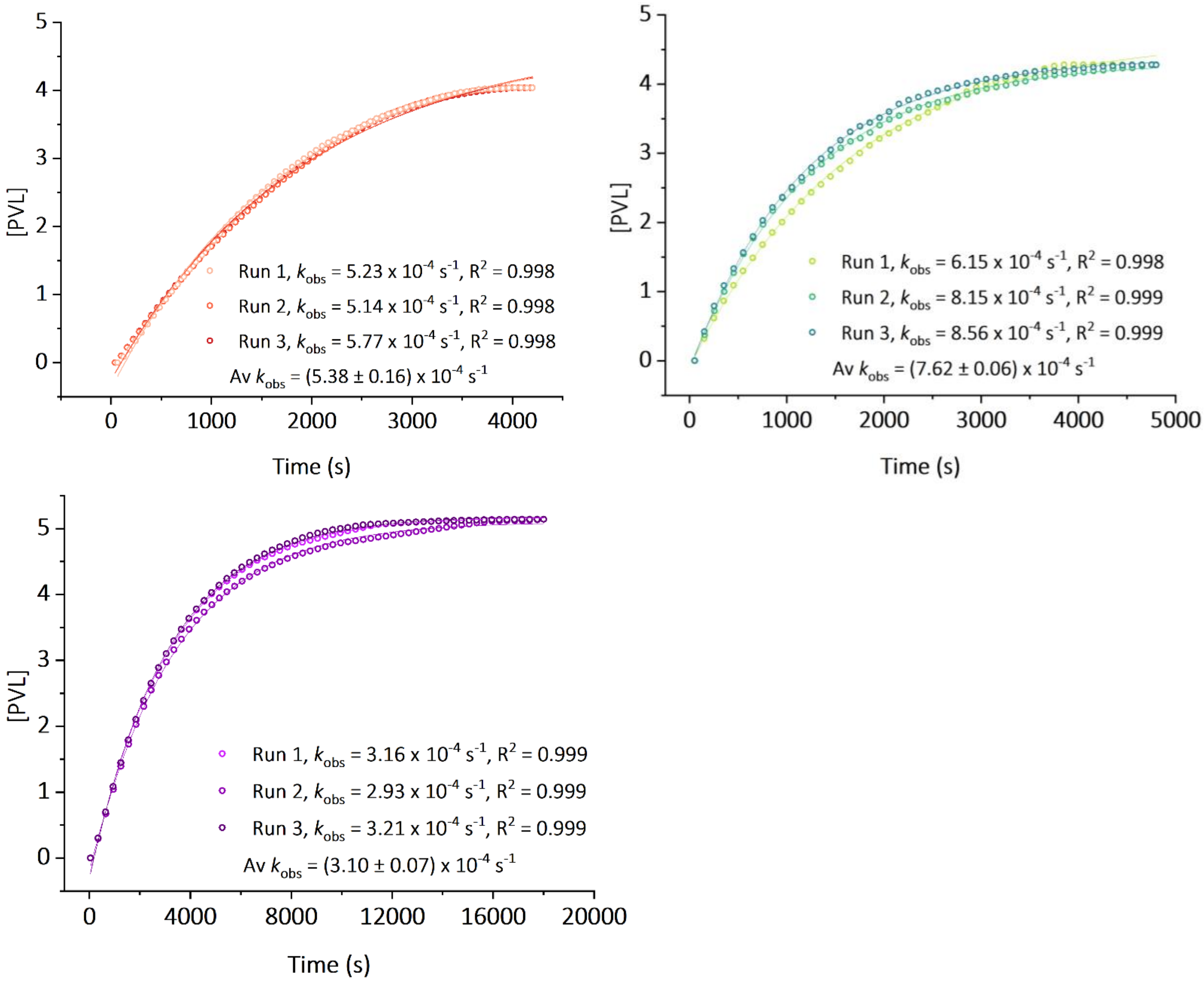

Fig. S26 Isothermal polymerization kinetic experiments, conducted on a DSC instrument, at 160 ^{o}C (conducted in triplicate) for the polymerization of VL with the $Zn(Oct)_2$ catalyst (top row, LHS), $Sn(Oct)_2$ catalyst (top row, RHS), and $Bi(Oct)_3$ catalyst (bottom row, LHS). For clarity, every nth data point from the datasets is shown, where n = 600, 1000 and 3000 for Zn(II), Sn(II) and Bi(III) respectively. Polymerization conditions: 1:1:1000 $M(Oct)_n$:BnOH:VL, [VL] = 6.25 M. In each case $[PVL]_t = 1 – [VL]_t$.

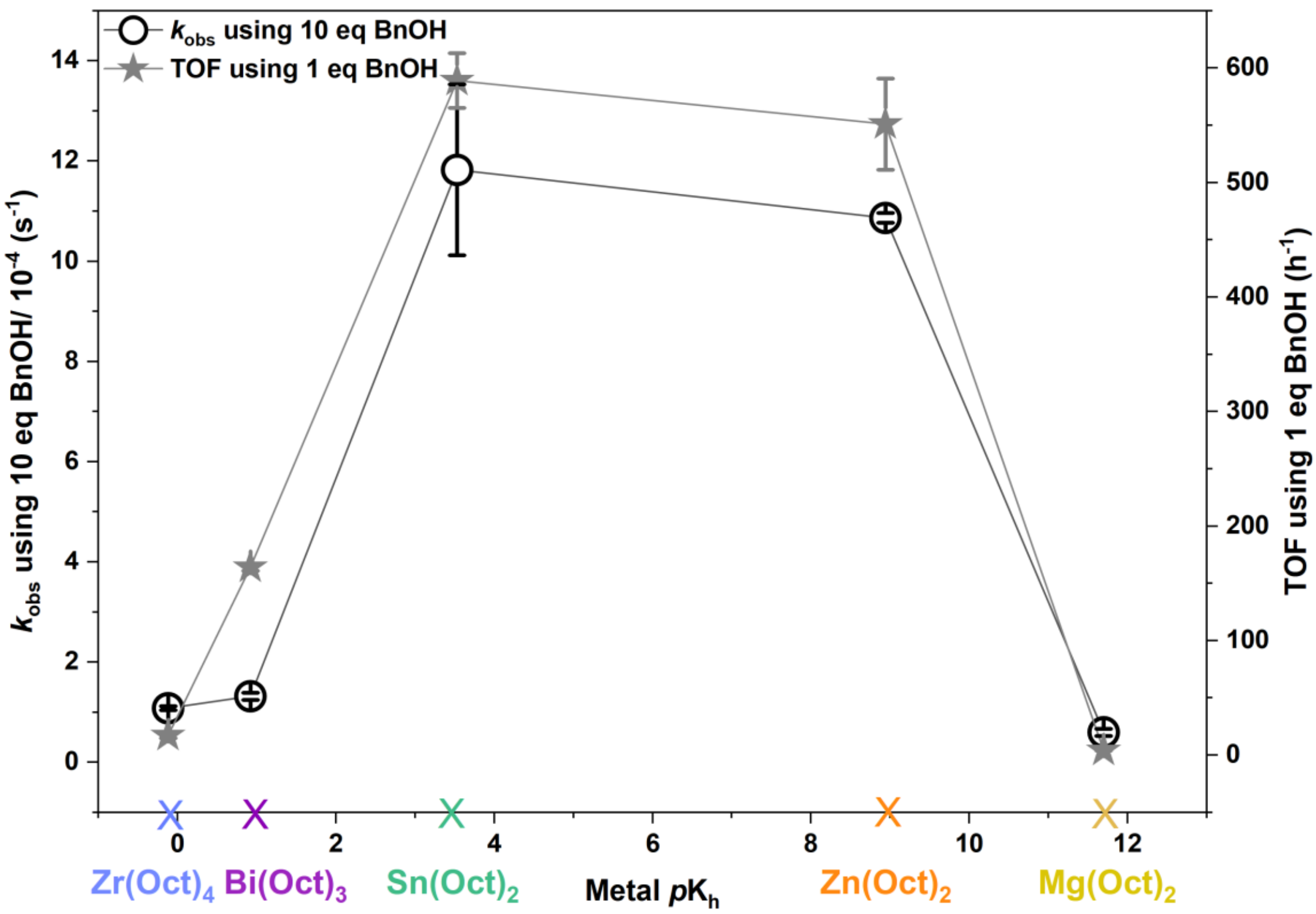

Fig. S27 Plot of k_{obs} using 10 eq BnOH and TOF using 1 eq BnOH *vs*. metal pK_h, indicating the LFER is upheld under both sets of polymerization conditions. Polymerization conditions: 1:x:1000 $M(Oct)_n$:BnOH:VL, x = 1 or 10, [VL] = 6.25 M. In each case $[PVL]_t = 1 – [VL]_t$.

To determine the order in $Mg(Oct)_2$ catalyst for the 6- and 7- membered lactones and cyclic carbonates, a series of polymerizations with varying catalyst loading (x) were completed using the TMC monomer at 160 °C (Table S8). Each reaction was run in triplicate, with an average k_{obs} determined as the average of three runs, and errors determined from the standard deviation of the mean of the three repeats. An example of this for the polymerization conducted using 2 equivalents of catalyst is shown in Fig. S28.

Table S8 k_{obs} determined at systematically varied catalyst loadings for the polymerization of TMC using the $Mg(Oct)_2$ catalyst and BnOH chain-transfer agent at 160 °C.

Eq $Mg(Oct)_2$	k_{obs} /10^{-3} (s^{-1})
0.25	0.74 ± 0.01
0.5	1.88 ± 0.18
1	4.35 ± 0.37
2	7.97 ± 0.49

Polymerization conditions: x:10x:1000 $Mg(Oct)_2$:BnOH:TMC, 160 °C, [TMC] = 6.25 M

Plotting ln(k_{obs}) *vs.* ln([$Mg(Oct)_2$]) yields a straight line with a gradient of 1.17, indicating a first-order dependence on $Mg(Oct)_2$ catalyst (Fig. S28). With this result, the same order was assumed for the remainder of the series of investigated monomers.

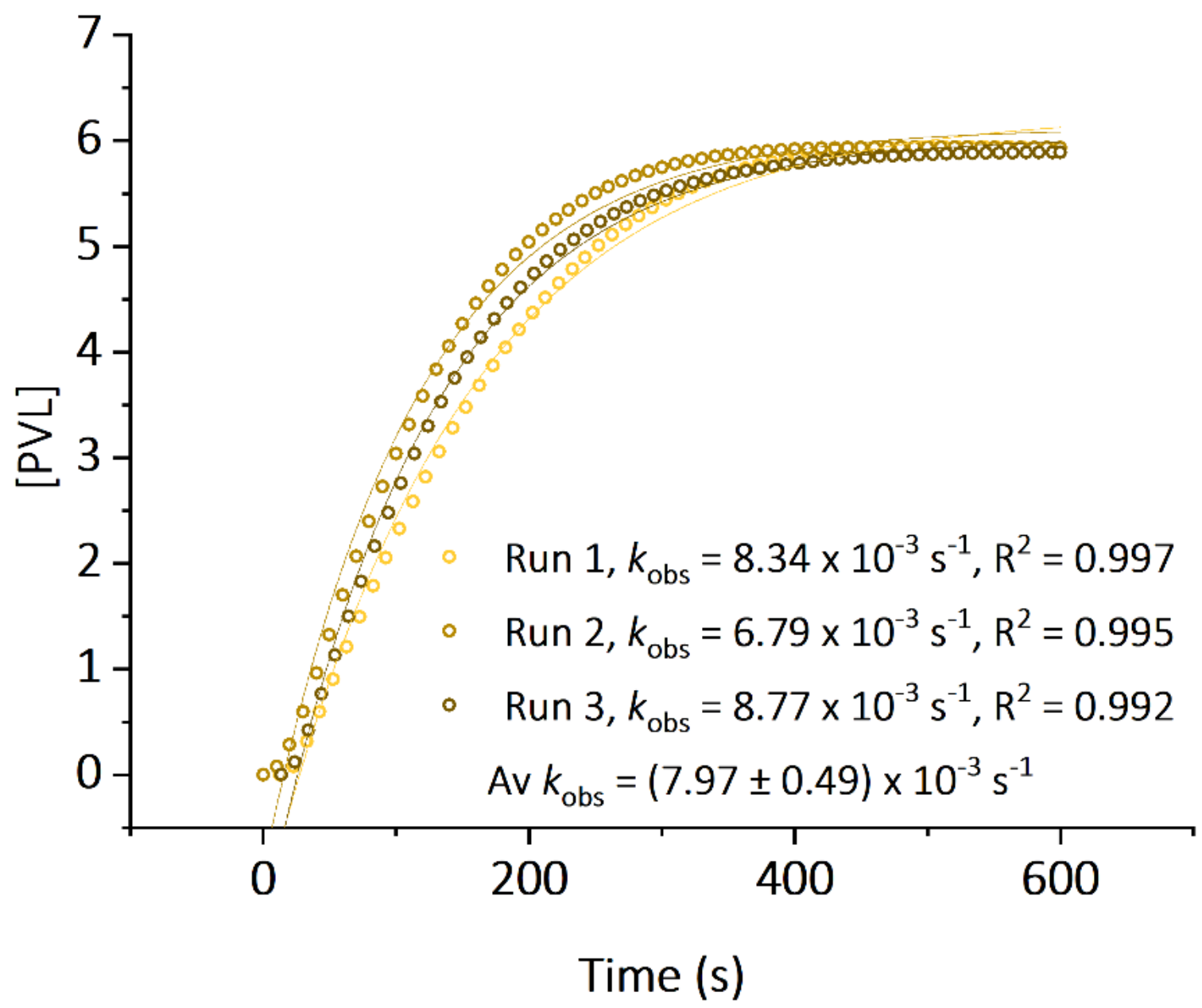

Fig. S28 Plots of [PTMC] *vs*. time for isotherms conducted at 160 °C. k_{obs} is taken from the exponential fitting of each line ($y = A - Be^{-k_{obs}x}$), with the average k_{obs} taken as the mean of the three repeats and error determined from the standard deviation of the mean of the three repeats. Polymerization conditions: 2:20:1000 $Mg(Oct)_2$:BnOH:TMC.

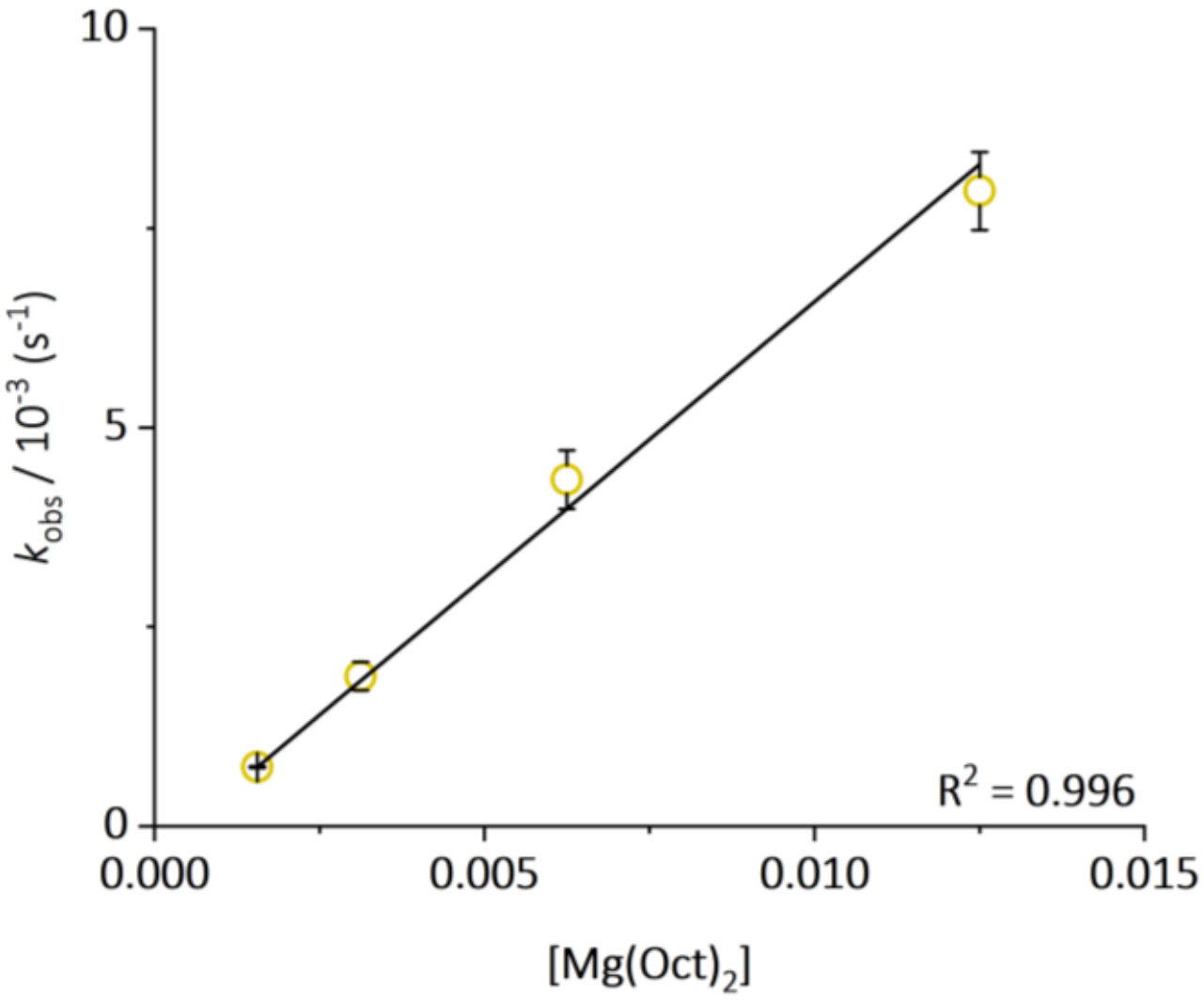

Fig. S29 Plot of k_{obs} *vs*. [Mg(Oct)$_2$] for isotherms conducted at 160 °C with polymerization conditions: x:10x:1000 Mg(Oct)$_2$:BnOH:TMC where x= 0.25, 0.5, 1, and 2, [TMC] = 6.25 M.

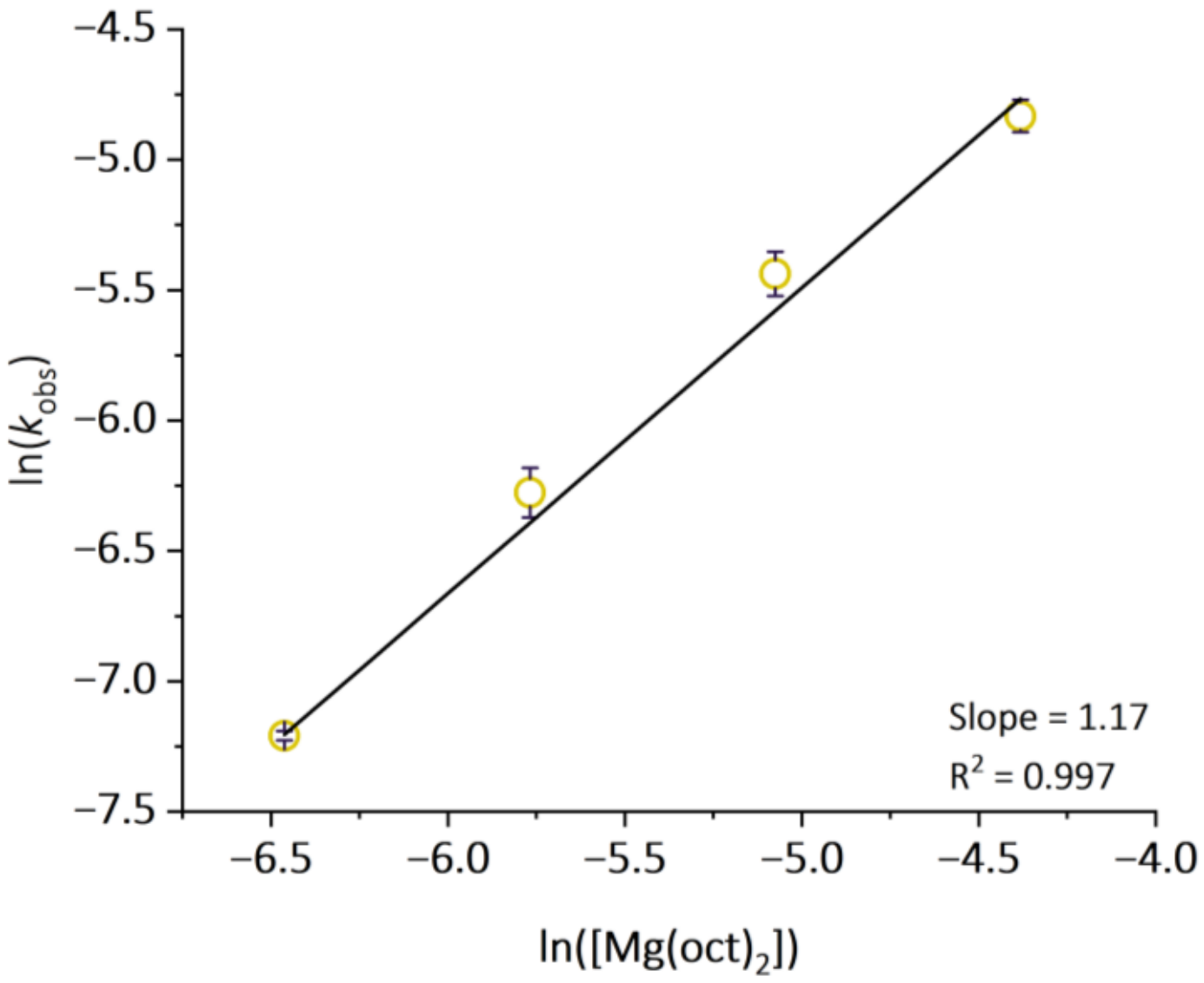

Fig. S30 Plot of ln(k_{obs}) *vs*. ln([Mg(Oct)$_2$]) for isotherms conducted at 160 °C with polymerization conditions: x:10x:1000 Mg(Oct)$_2$:BnOH:TMC where x= 0.25, 0.5, 1, and 2, [TMC] = 6.25 M.

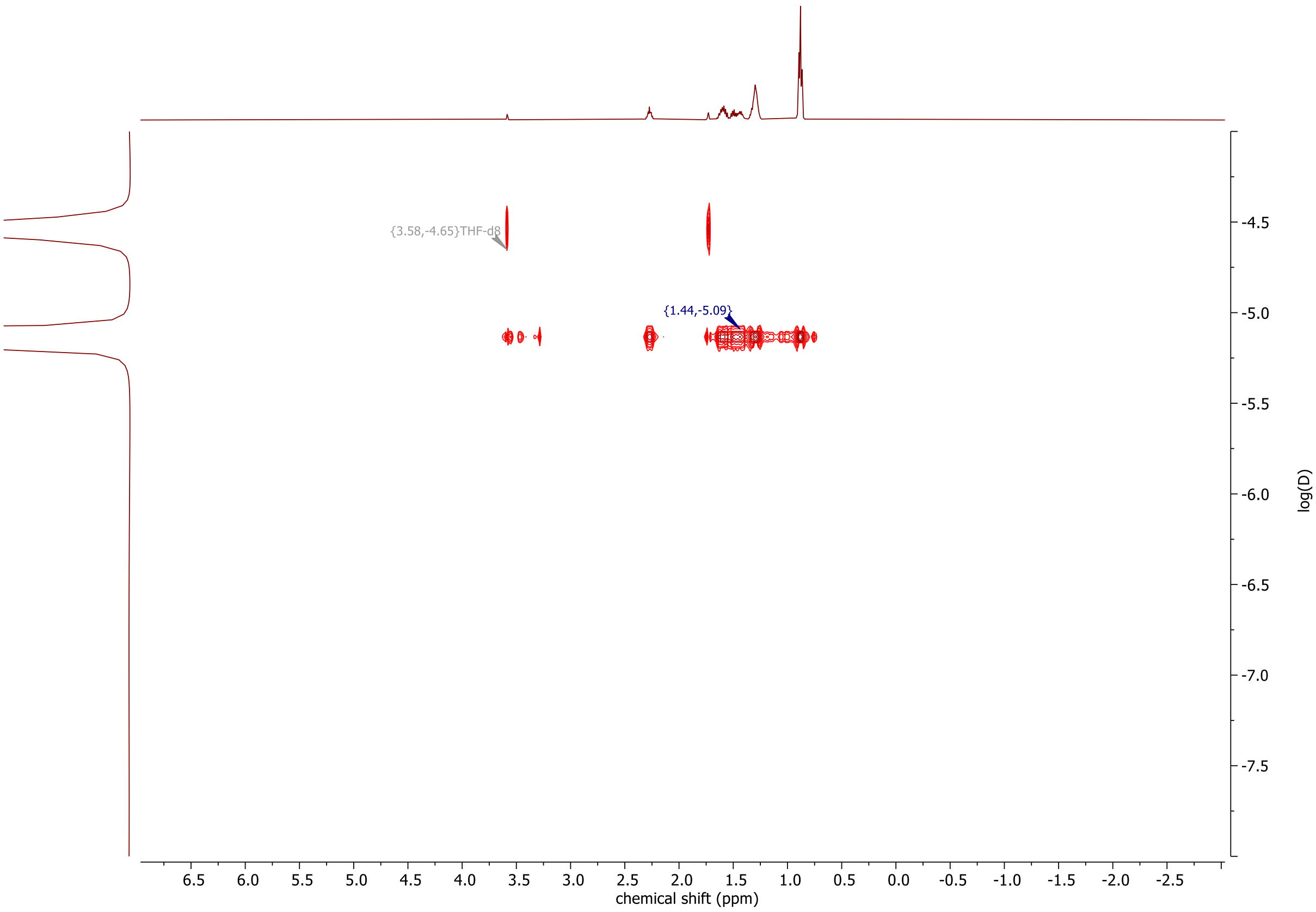

Fig. S31. DOSY NMR Spectrum of $Mg(Oct)_2$ catalyst in d-THF at 50 ^oC. Two diffusion coefficients are observed, for the d-THF solvent (log(D) = -4.65) and for the $Mg(Oct)_2$ catalyst (log(D) = -5.09).

The apparent molecular weight of the $Mg(Oct)_2$ species may be predicted using external calibration curves (ECCs) and the diffusion coefficients of the d-THF and $Mg(Oct)_2$.[6] Using such an approach, the molecular weight estimation of small molecules is significantly affected by the applied shape model. For alkali organometallics, the ECC for dissipated spheres and ellipsoids was shown to be the best model. Therefore, this ECC_{DSE} was applied:

From the log(D) values in Fig. S31, the ratio of diffusion coefficients, $D_{Mg}/D_{d\text{-}THF} = 0.3631$.

Then, using the power-law relationship which correlates diffusion coefficient (D) and molecular weight (MW), $D = K\,MW^{\alpha}$, and the external calibration curve presented by Neufeld and Stalke (which give values for K and α, assuming the dissipated sphere or ellipsoid (DSE)),[6] the following equation was used to estimate the molecular weight:

$MW_{Mg} = MW_{d\text{-}THF}(D_{Mg}/D_{d\text{-}THF})^{1/\alpha}$, where using the ECC_{DSE}, $\alpha = -0.5824$

This gives $MW_{Mg} = 465.5$ g mol^{-1}. This value is consistent with a monomeric $Mg(Oct)_2$ species, [$Mg(Oct)_2 \cdot 2$ THF] (MW = 454.9 g mol^{-1}).

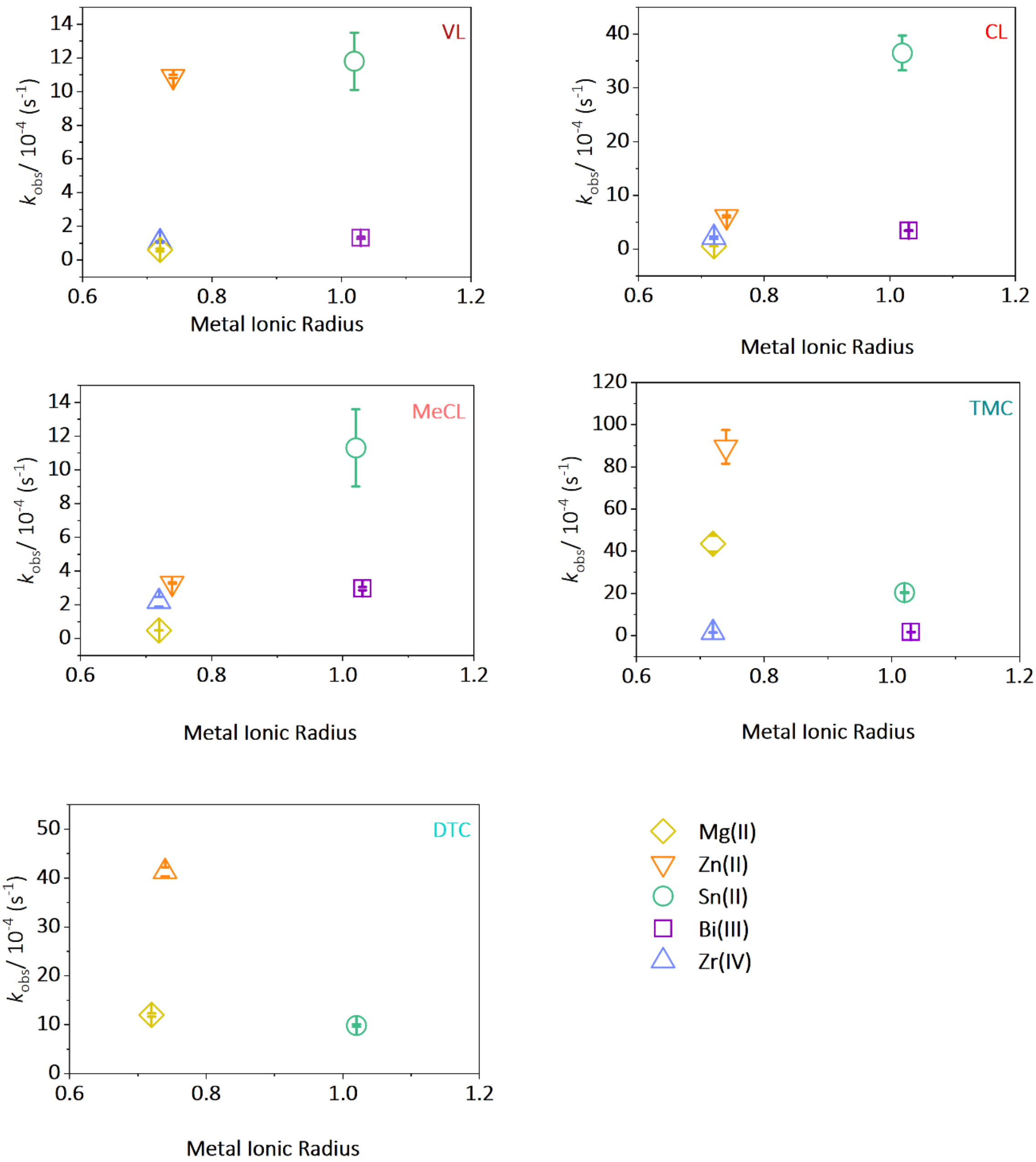

Fig. S32. Plot showing k_{obs} *vs*. metal ionic radius (coordination number 6) for the polymerization of each monomer (VL, CL, MeCL, TMC and DTC) catalyzed by $M(Oct)_n$. Polymerization conditions: $M(Oct)_n$:BnOH:monomer, 1:10:1000, 160 °C, [monomer] = 6.25 M in 1,2-dimethoxybenzene. Ionic radii were taken from [7, 8].

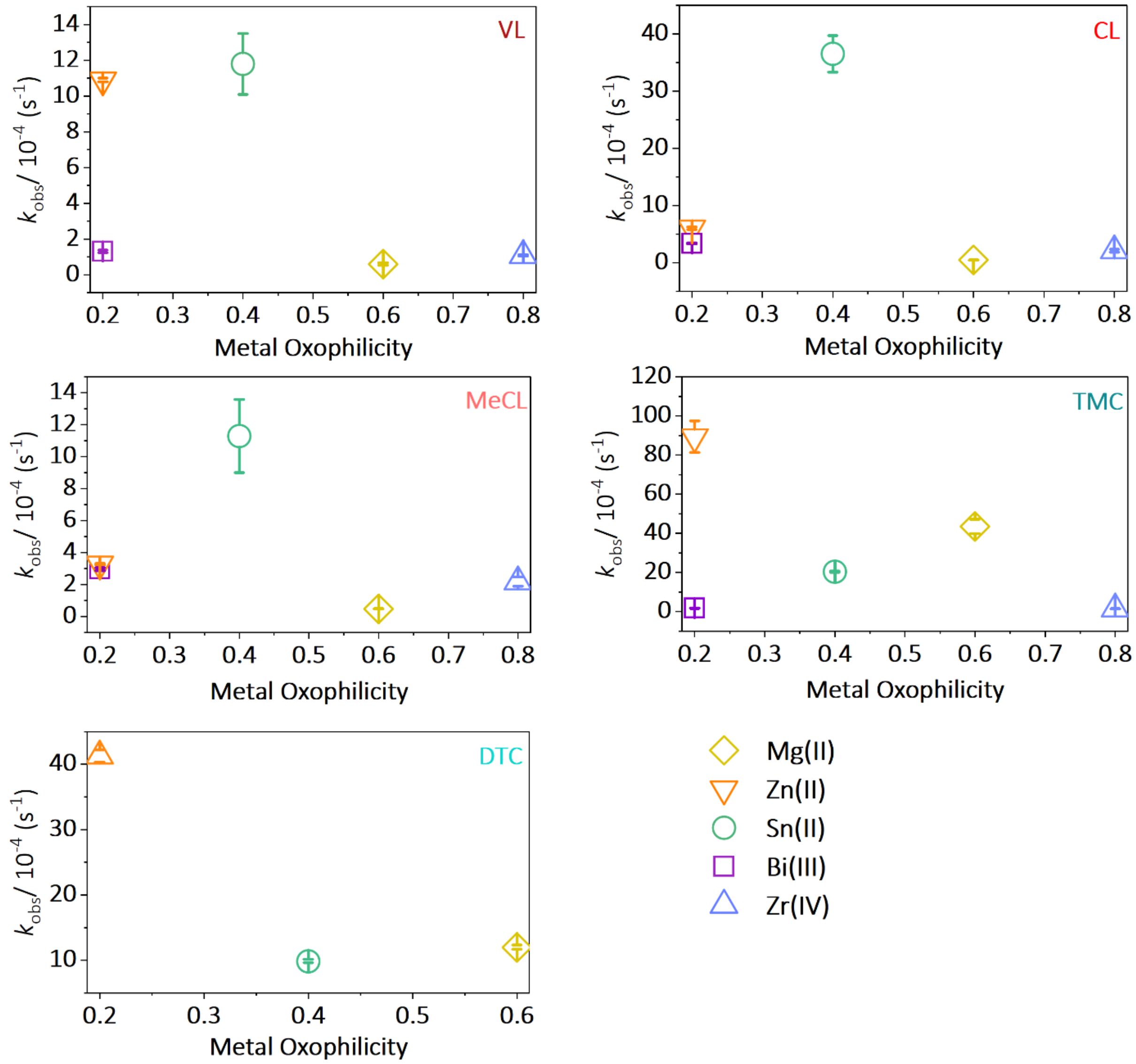

Fig. S33. Plot showing k_{obs} *vs*. metal oxophilicity for the polymerization of each monomer (VL, CL, MeCL, TMC and DTC) catalyzed by $M(Oct)_n$. Polymerization conditions: $M(Oct)_n$:BnOH:monomer, 1:10:1000, 160 °C, [monomer] = 6.25 M in 1,2-dimethoxybenzene. Oxophilicity values taken from [9].

MALDI-TOF analysis of poly(valerolactone) end groups for two polymer samples, one produced using $Zn(Oct)_2$ revealed species reflective of the coordination-insertion mechanism for $Zn(Oct)_2$.[10]

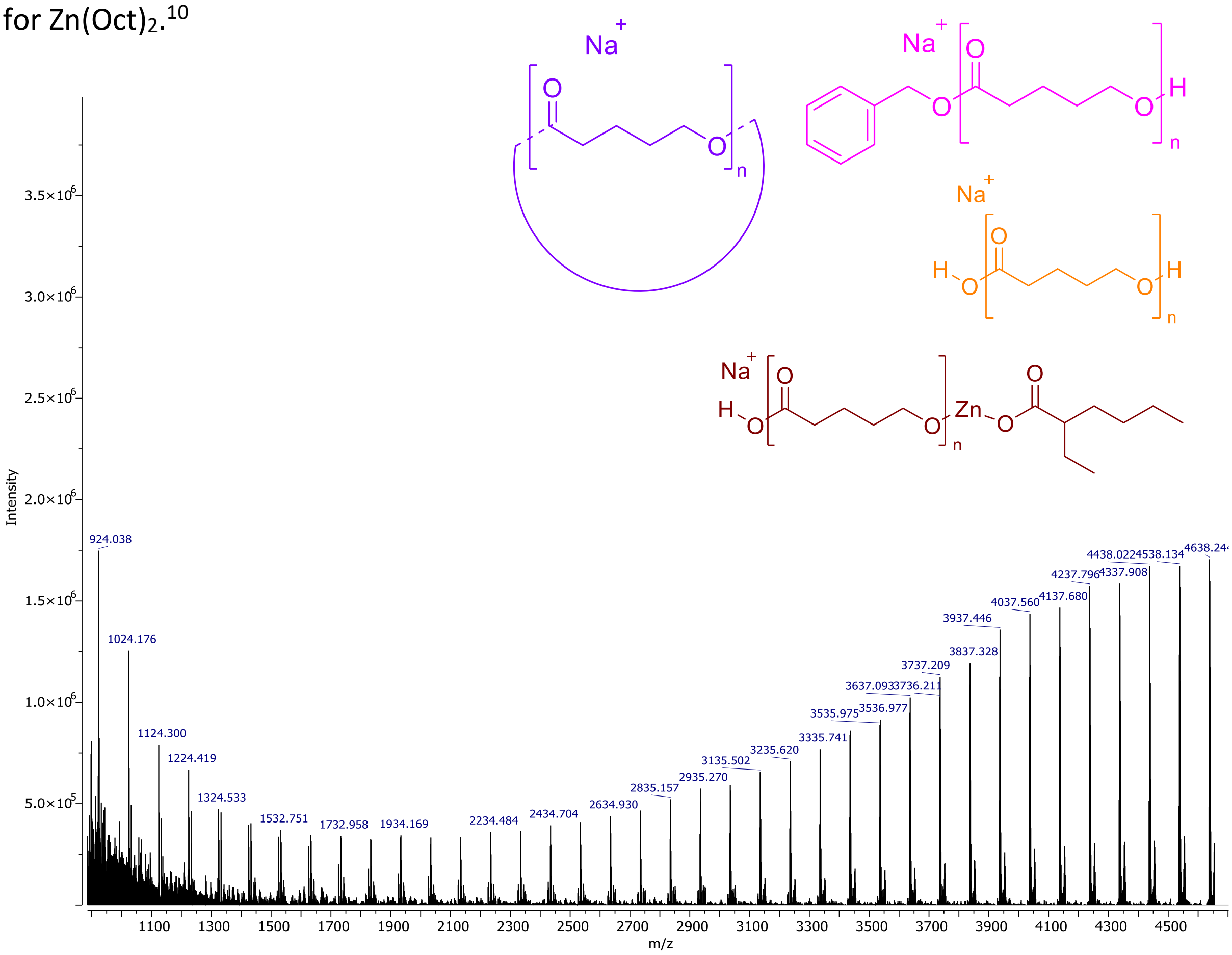

Fig. S34 MALDI-TOF Spectrum for PVL produced using $Zn(Oct)_2$ catalyst. The polymer (10 mg mL^{-1} in THF), and dithranol (10 mg mL^{-1} in THF) were premixed in a 1:25 ratio (v/v). Identified end group structures are given and these are analyzed in detail in the following figures (Fig. S35-S46).

4 end-groups were identified with MALDI-TOF analysis of poly(valerolactone) synthesised using the $Zn(Oct)_2$ catalyst: a benzyl alcohol initiated chain (Na^+) (pink structure above, Fig. S35-S37), a water initiated chain (Na^+) (orange structure above, Fig. S38-S40), cyclic PVL (Na^+) (purple structure above, Fig. S41-S43), and a Zn(Oct) initiated chain (Na^+) (maroon structure above, Fig. S44-S46). All four of these species are analogous to those identified in prior work on the coordination-insertion mechanism, using Sn(Oct)2 for the polymerization of CL.[10]

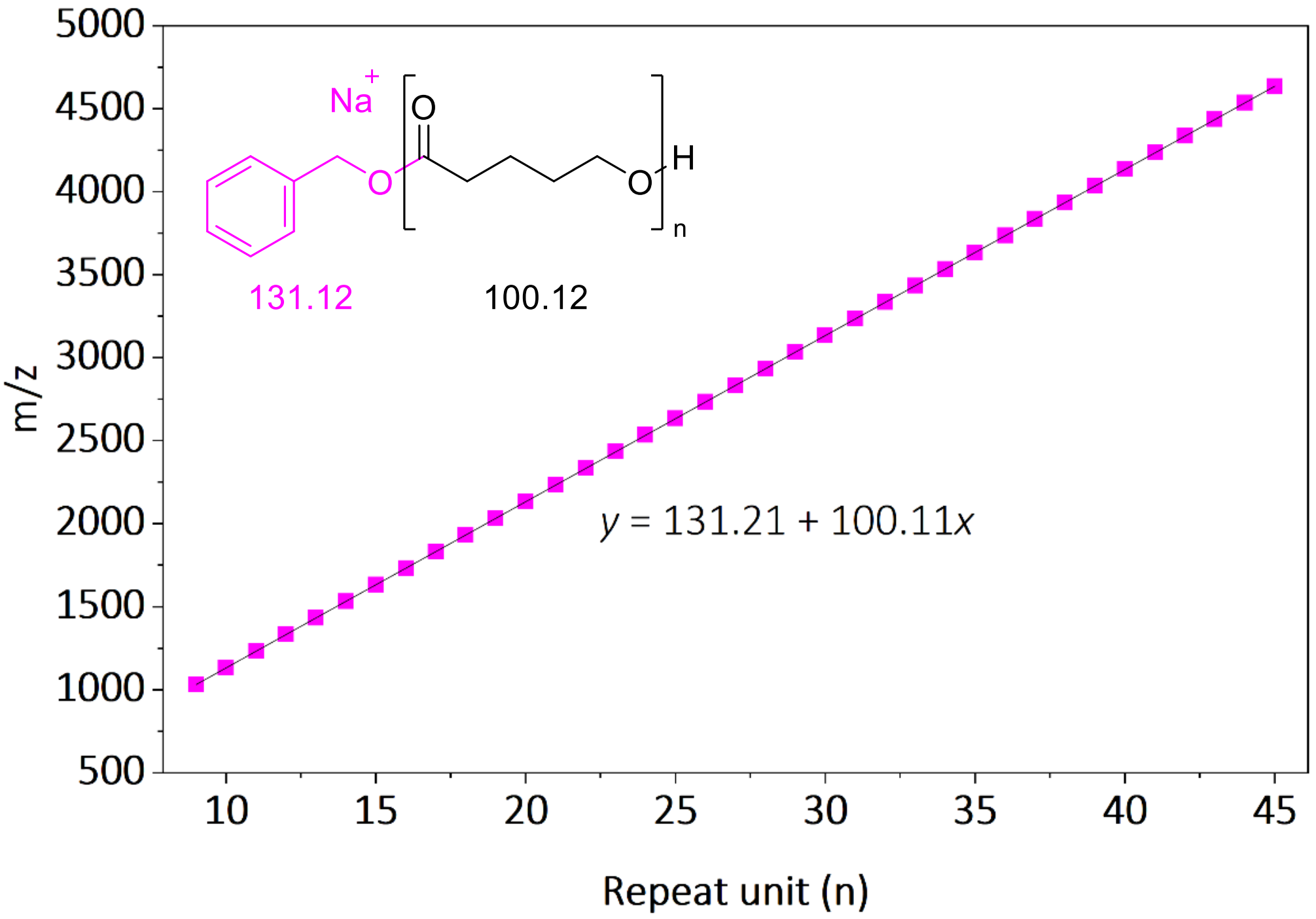

Fig. S35 m/z *vs.* repeat unit plot for the first series of identified peaks from MALDI-TOF analysis of PVL produced using the Zn(Oct)$_2$ catalyst, where the slope gives the repeat unit and the intercept gives the end group molar mass.

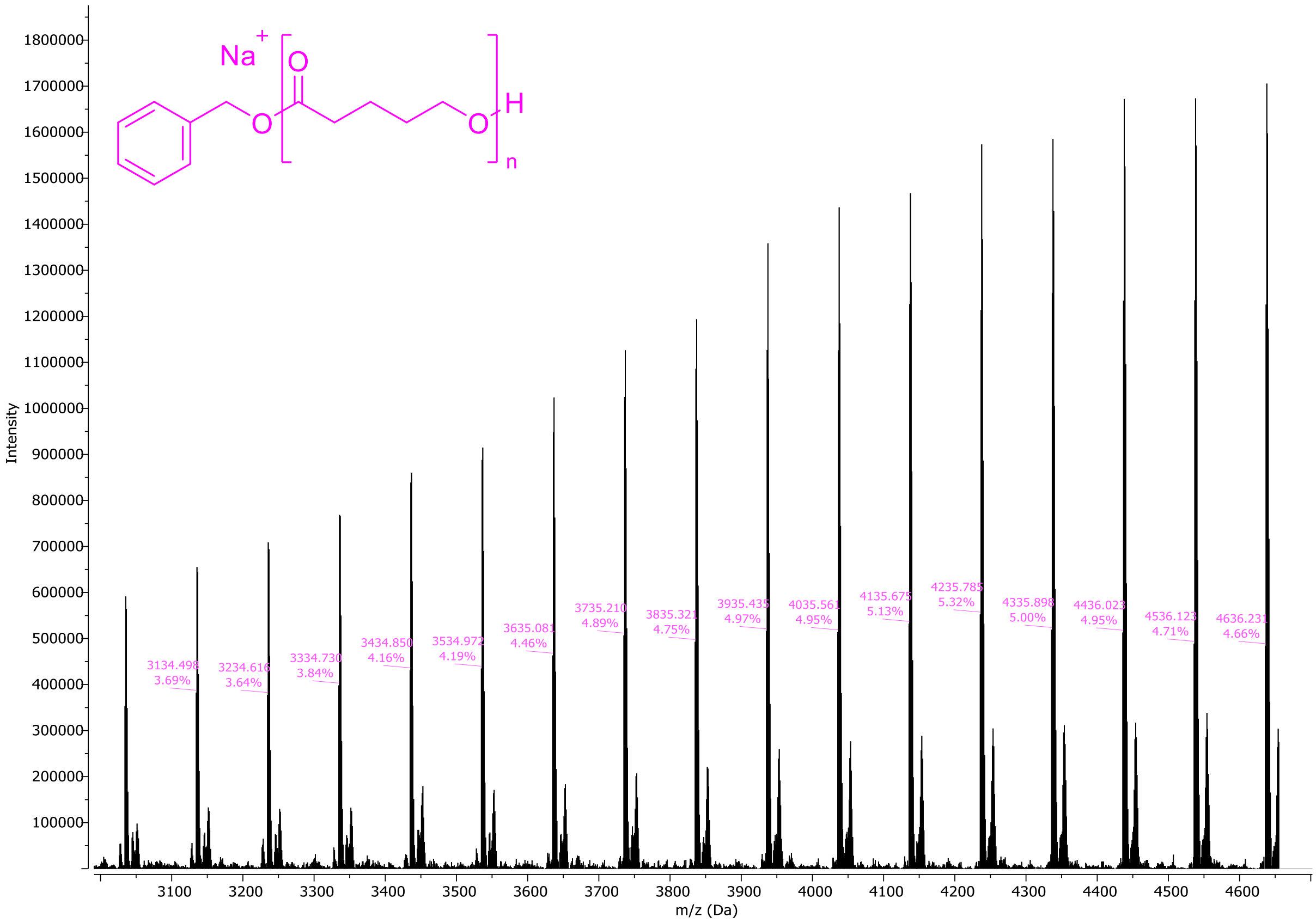

Fig. S36 MALDI-TOF Spectrum for PVL produced using Zn(Oct)$_2$ catalyst, focusing on peaks identified as PVL with a benzyl alcohol end group (doped with Na^+). The polymer (10 mg mL^{-1} in THF), and dithranol (10 mg mL^{-1} in THF) were premixed in a 1:25 ratio (v/v).

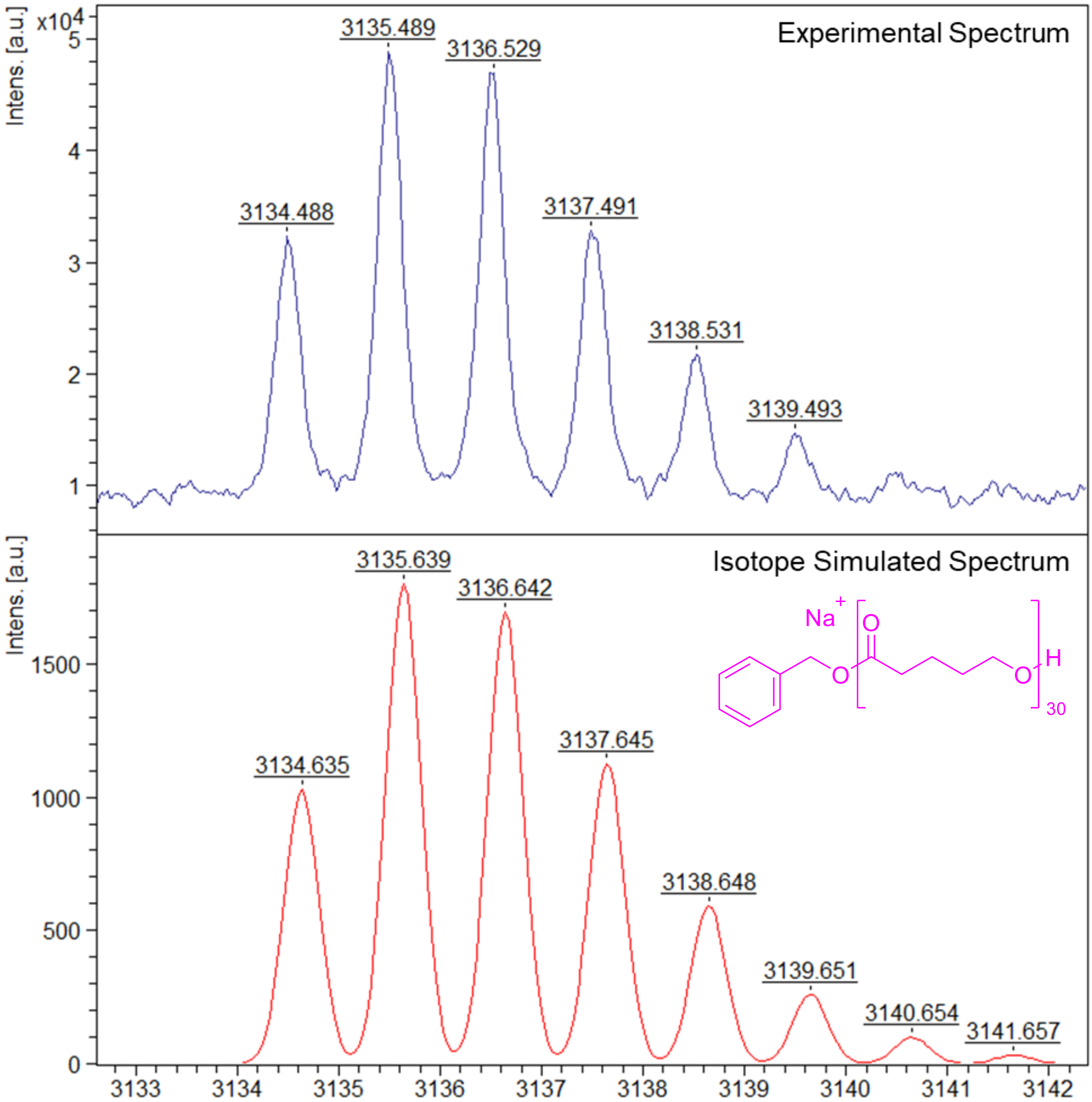

Fig. S37 The experimental spectrum stacked with an isotope simulated spectrum for $C_5H_7O[C_5H_8O_2]_{30}H$, Na^+ m/z = 3134.64.

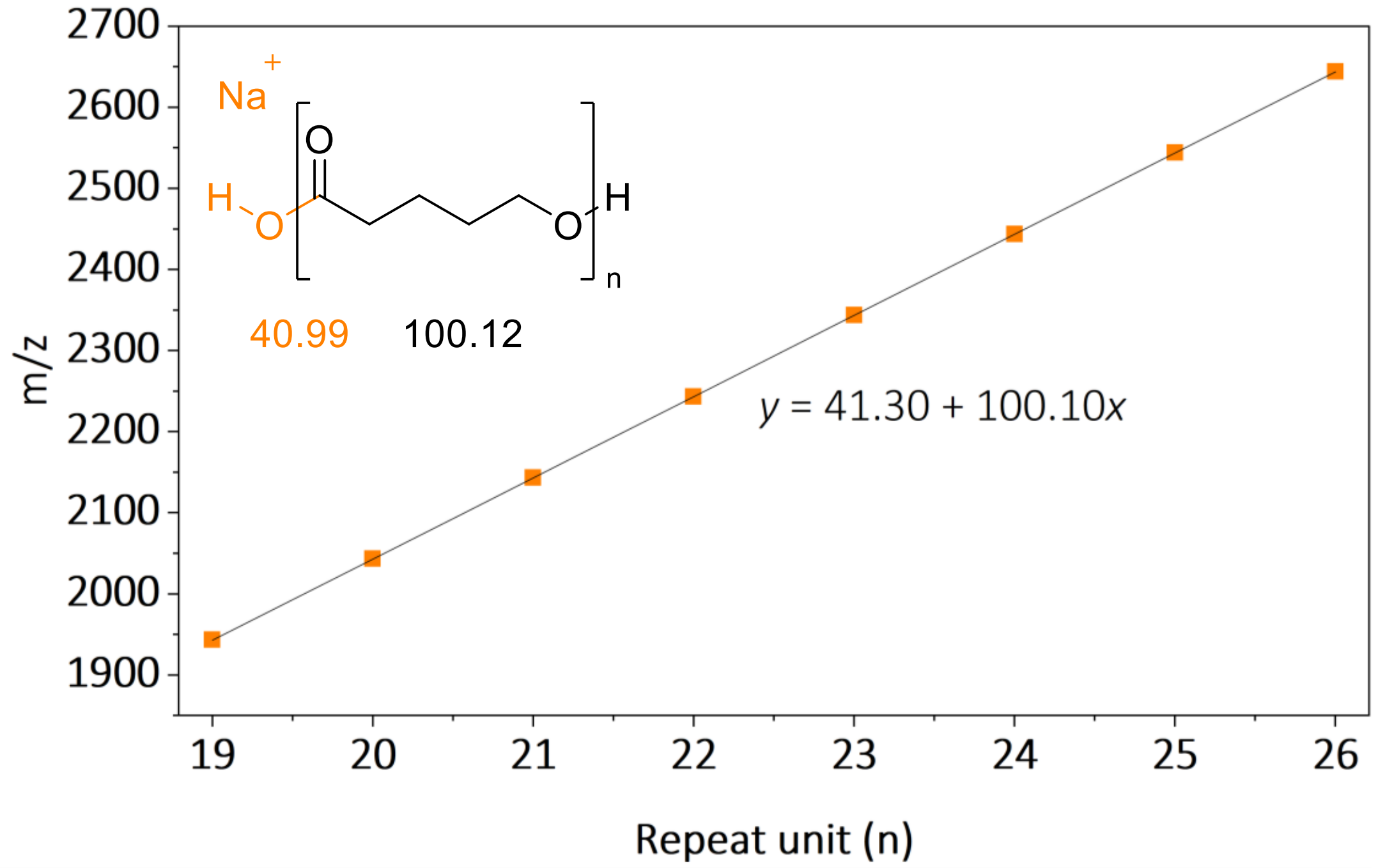

Fig. S38 m/z *vs.* repeat unit plot for the second series of identified peaks from MALDI-TOF analysis of PVL produced using the Zn(Oct)$_2$ catalyst, where the slope gives the repeat unit and the intercept gives the end group molar mass.

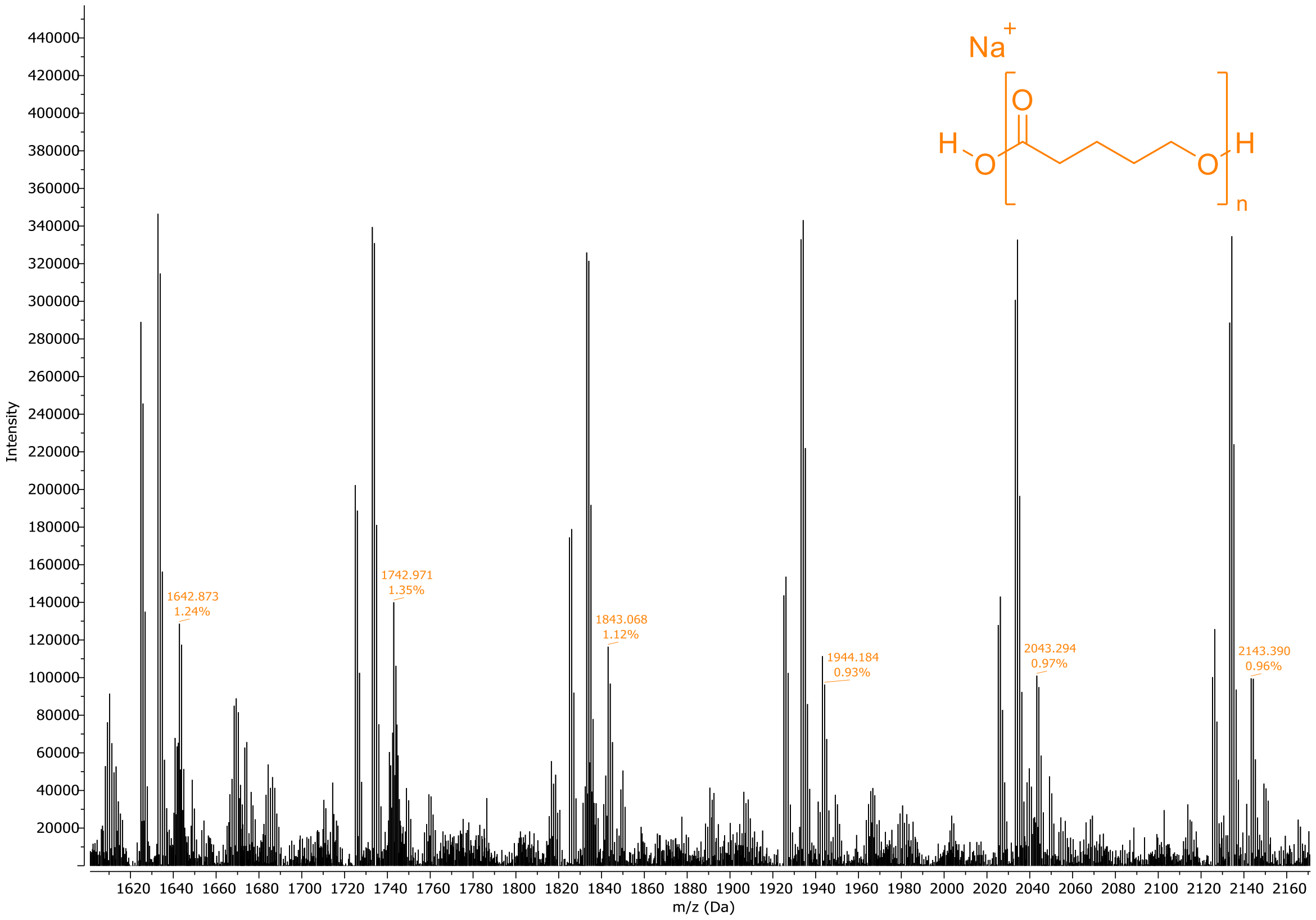

Fig. S39 MALDI-TOF Spectrum for PVL produced using Zn(Oct)$_2$ catalyst, focusing on peaks identified as PVL with a hydroxyl end group (doped with Na$^+$). The polymer (10 mg mL^{-1} in THF), and dithranol (10 mg mL^{-1} in THF) were premixed in a 1:25 ratio (v/v).

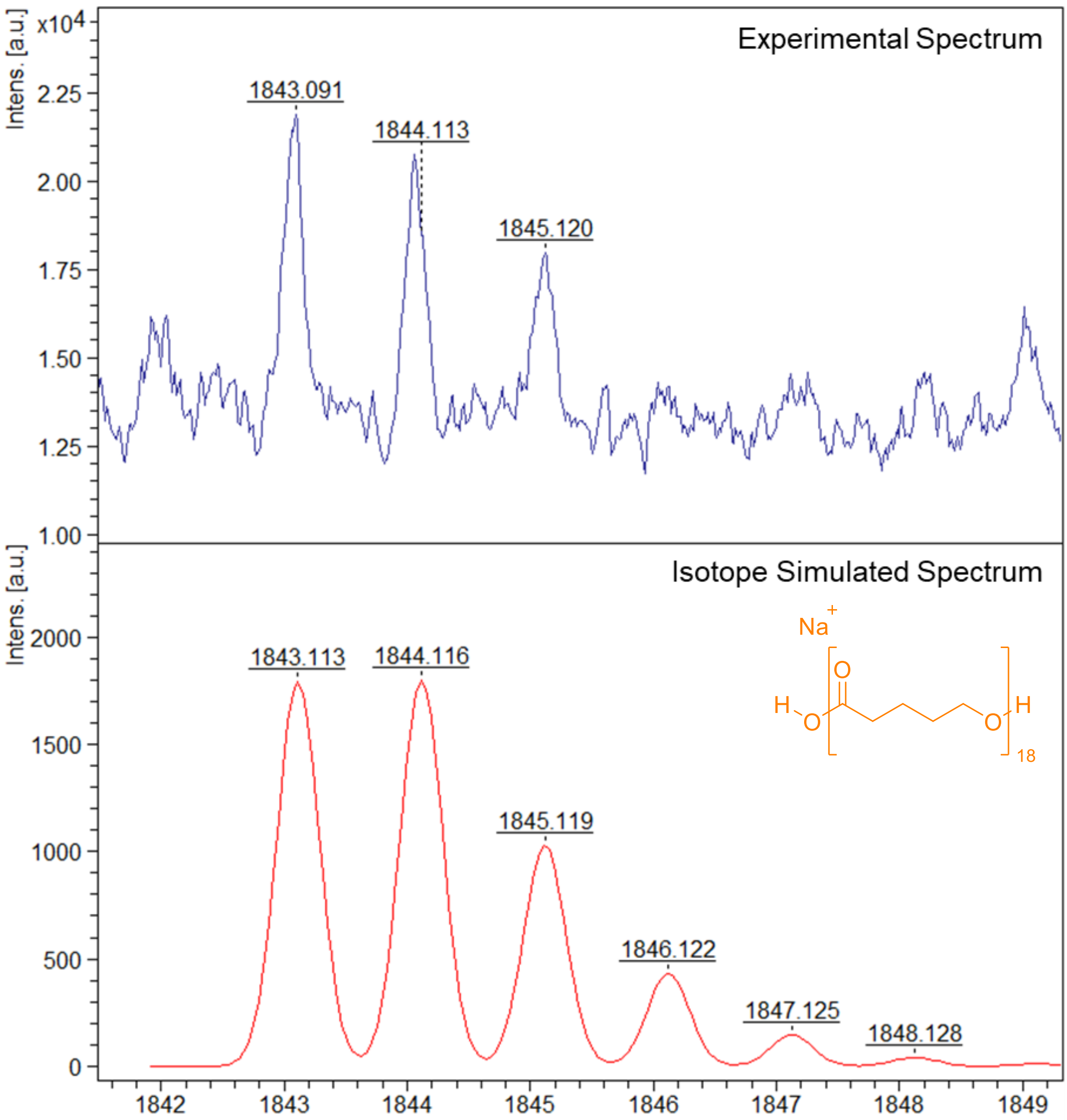

Fig. S40 The experimental spectrum stacked with an isotope simulated spectrum for HO[$C_5H_8O_2$]$_{18}$H, Na^+ m/z = 1843.11.

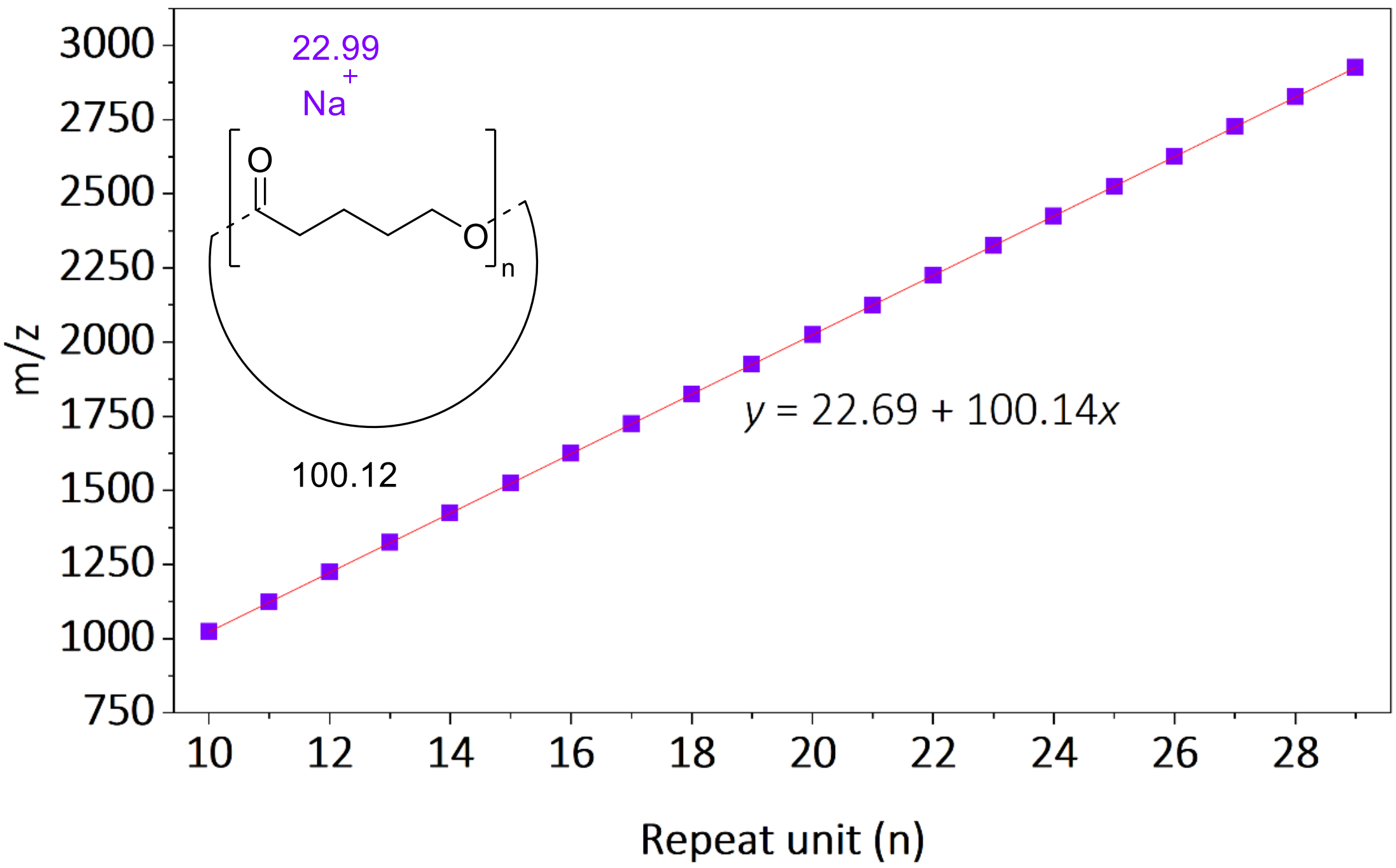

Fig. S41 m/z *vs*. repeat unit plot for the third series of identified peaks from MALDI-TOF analysis of PVL produced using the Zn(Oct)$_2$ catalyst, where the slope gives the repeat unit and the intercept gives the end group molar mass.

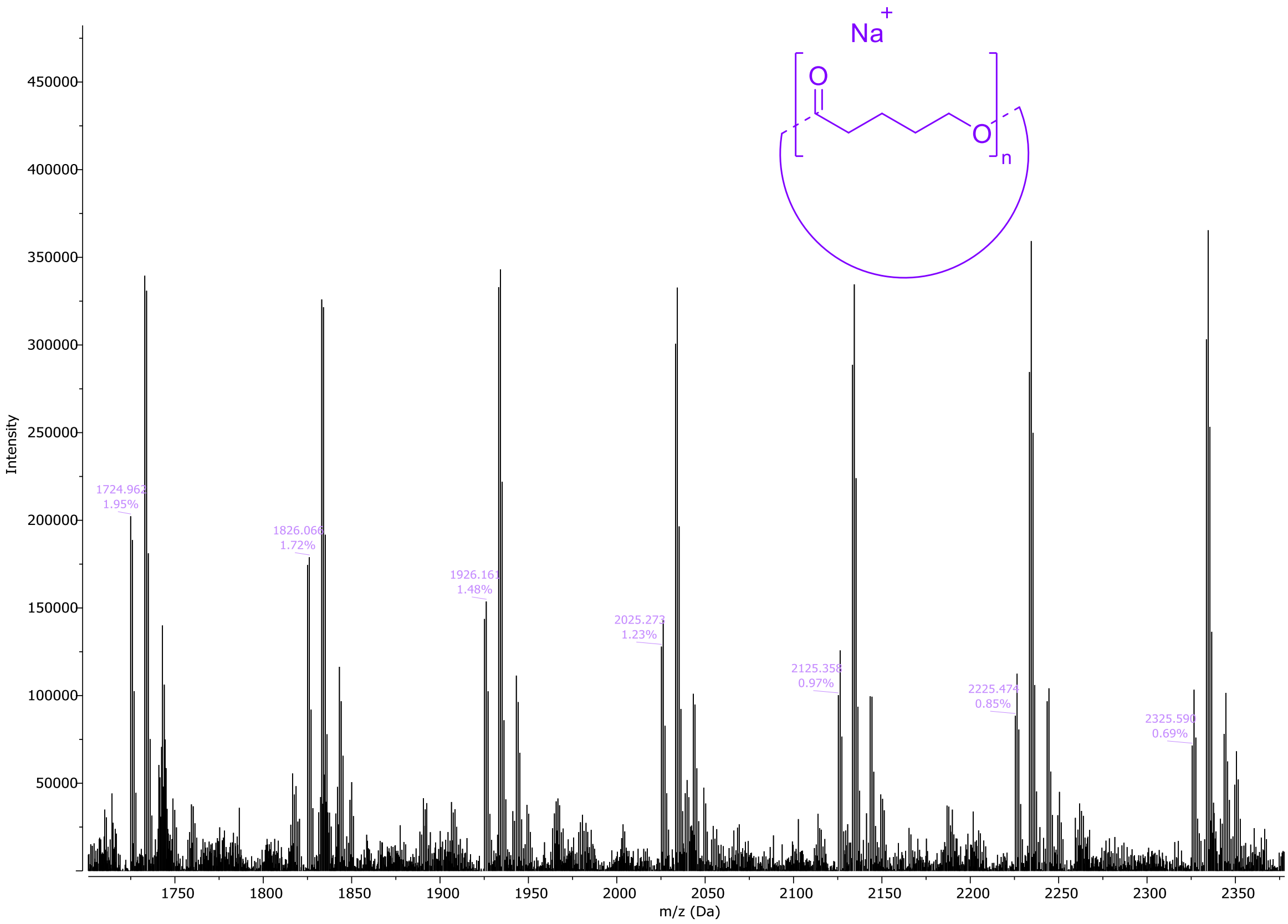

Fig. S42 MALDI-TOF Spectrum for PVL produced using Zn(Oct)$_2$ catalyst, focusing on peaks identified as cyclic PVL (doped with Na$^+$). The polymer (10 mg mL^{-1} in THF), and dithranol (10 mg mL^{-1} in THF) were premixed in a 1:25 ratio (v/v).

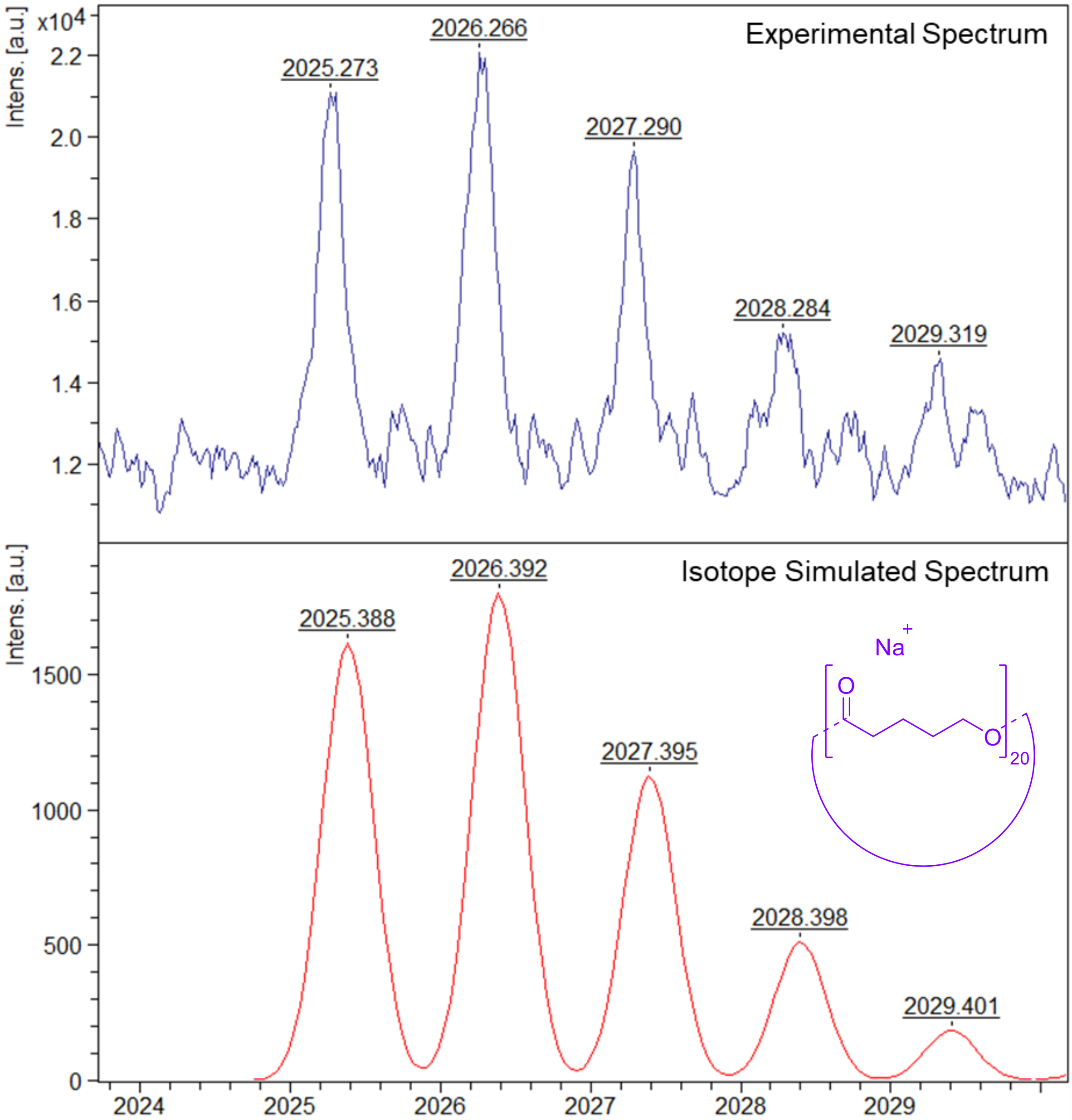

Fig. S43 The experimental spectrum stacked with an isotope simulated spectrum for $[C_5H_8O_2]_{20}$, Na^+ m/z = 2025.39.

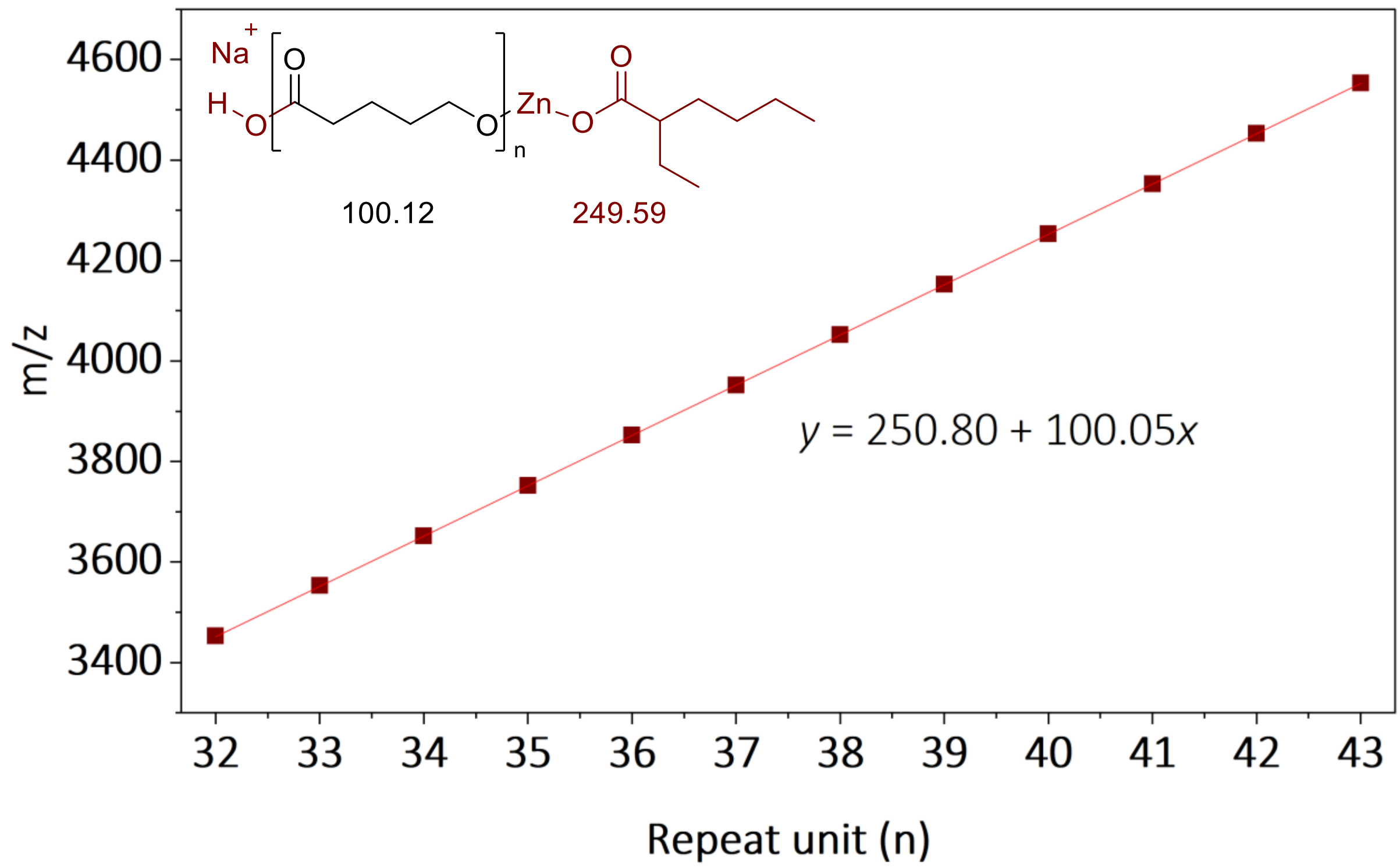

Fig. S44 m/z *vs*. repeat unit plot for the fourth series of identified peaks from MALDI-TOF analysis of PVL produced using the Zn(Oct)$_2$ catalyst, where the slope gives the repeat unit and the intercept gives the end group molar mass.

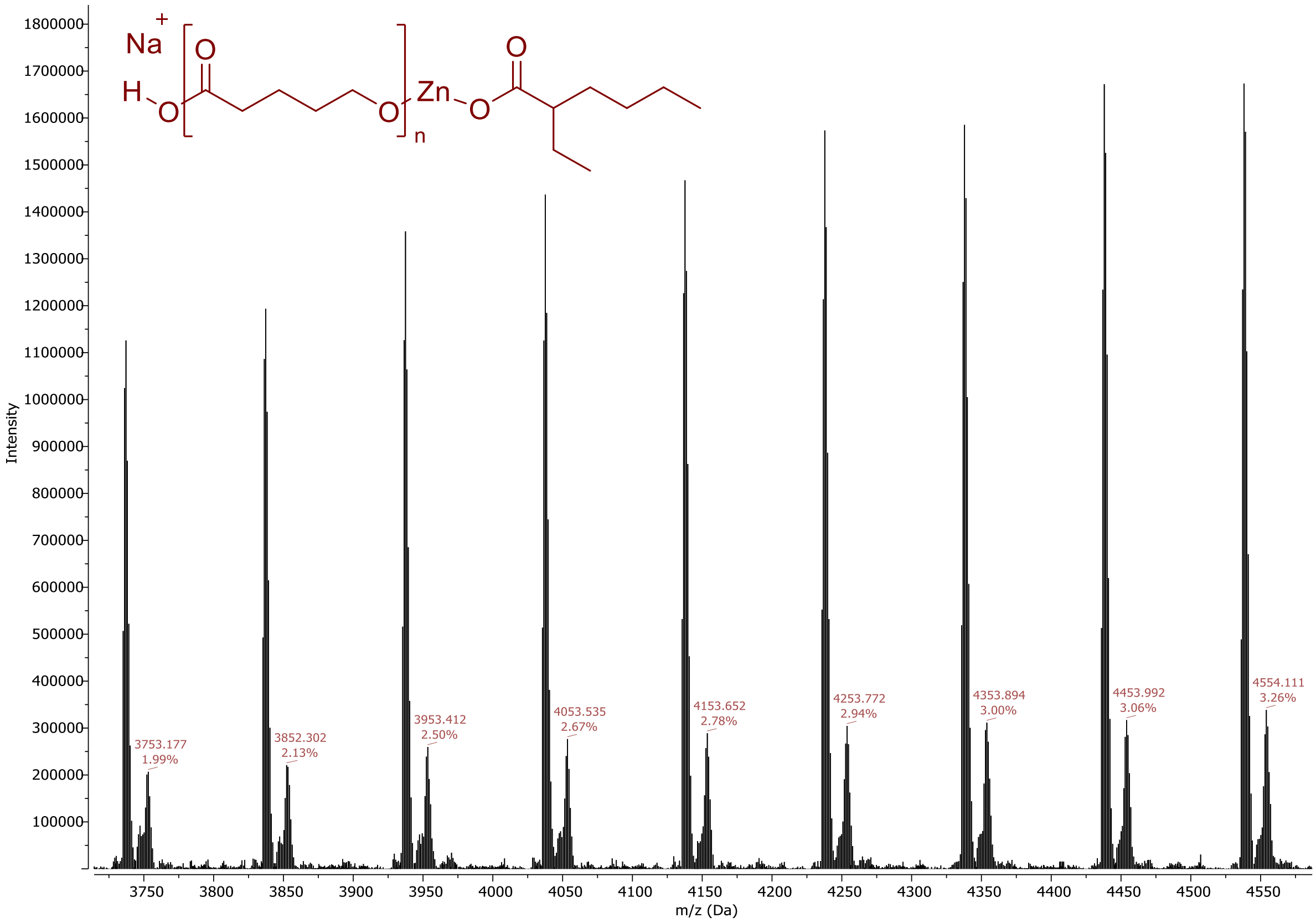

Fig. S45 MALDI-TOF Spectrum for PVL produced using Zn(Oct)$_2$ catalyst, focusing on peaks identified as PVL with a Zn(Oct) end group (doped with Na^+). The polymer (10 mg mL^{-1} in THF), and dithranol (10 mg mL^{-1} in THF) were premixed in a 1:25 ratio (v/v).

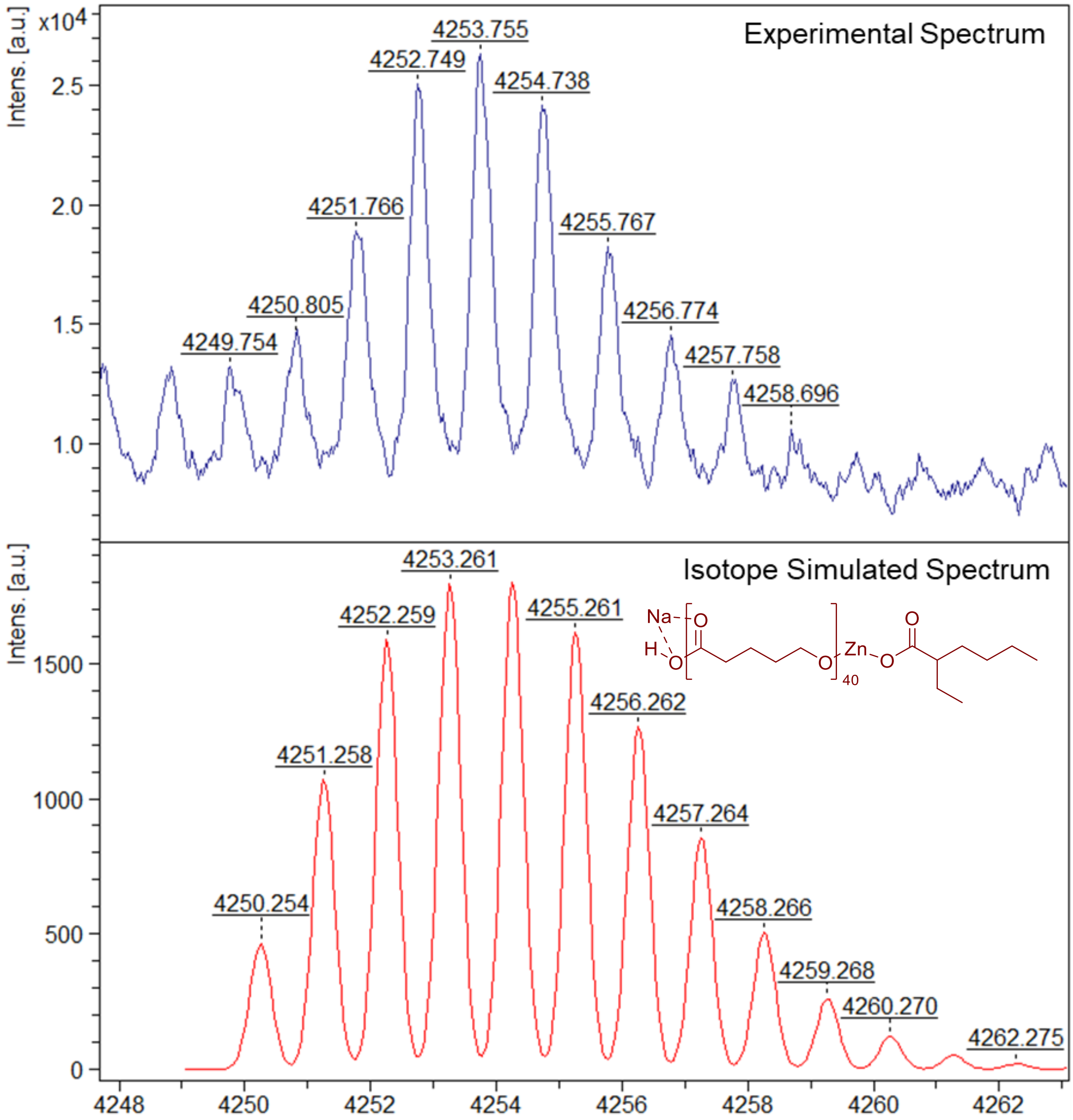

Fig. S46 The experimental spectrum stacked with an isotope simulated spectrum for $HO[C_5H_8O_2]_{40}Zn(CO_2(CH(C_4H_9)(C_2H_5)))$, Na^+ m/z = 4253.26.

4 end-groups were identified with MALDI-TOF analysis of poly(valerolactone) synthesised using the $Bi(Oct)_3$ catalyst (Fig. S47): a water initiated chain (Na^+) (orange structure below, Fig. S48-S50), a water initiated chain (K^+) (green structure below, Fig. S51-S53), a benzyl alcohol initiated chain (Na^+) (pink structure below, Fig. S54-S56), and a benzyl alcohol initiated chain (K^+) (blue structure below, Fig. S57-S59). Species differ from those identified using $Zn(Oct)_2$ (Fig. S34-46) and those previously reported for $Sn(Oct)_2$, indicative of the coordination-insertion mechanism, suggesting that the $Bi(Oct)_3$ follows a different mechanism of polymerization.[10]

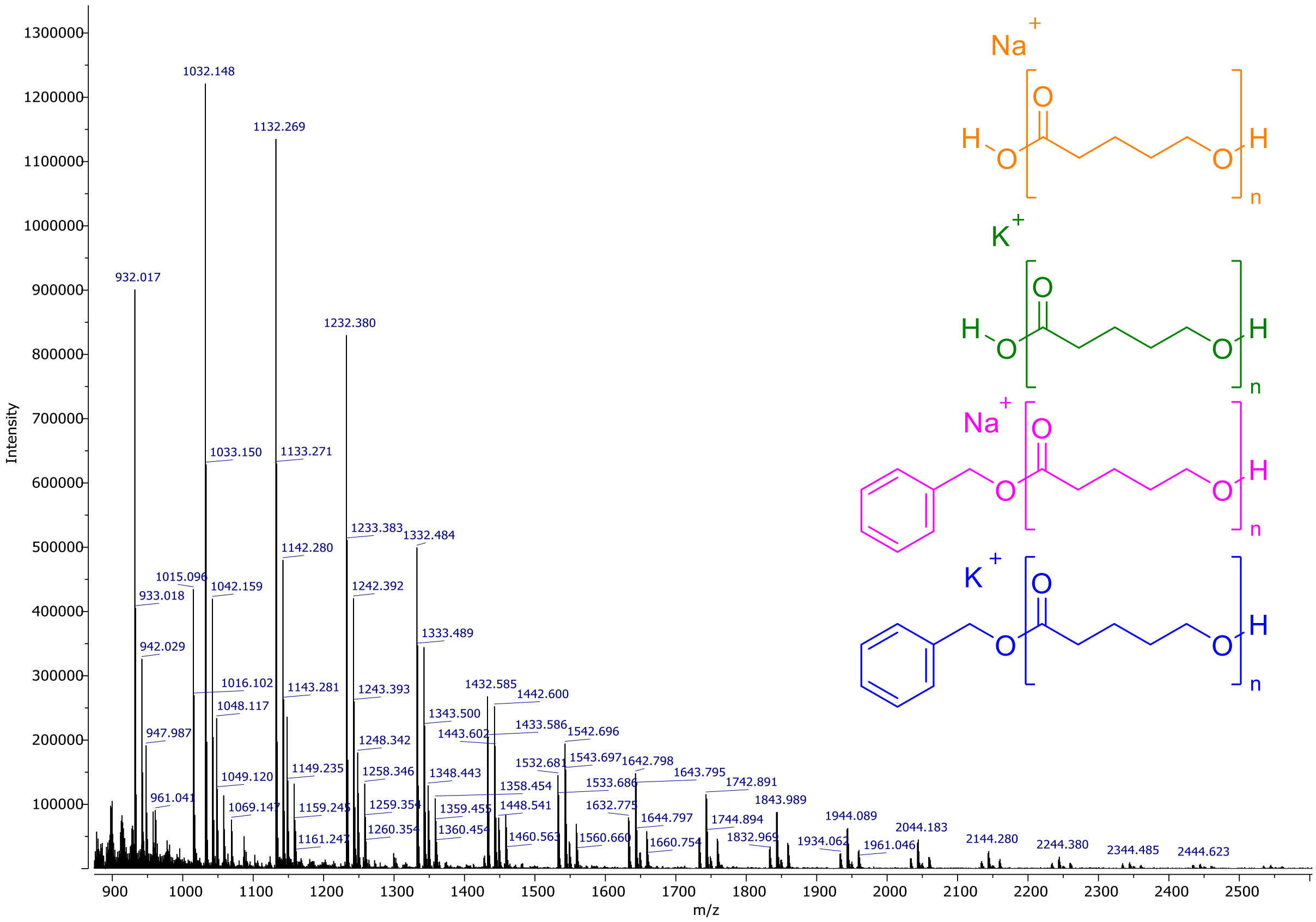

Fig. S47 MALDI-TOF Spectrum for PVL produced using Bi(Oct)$_3$ catalyst. The polymer (10 mg mL^{-1} in THF), and dithranol (10 mg mL^{-1} in THF) were premixed in a 1:25 ratio (v/v). Identified end group structures are given and these are analyzed in detail in the following figures (Fig. S48-S59).

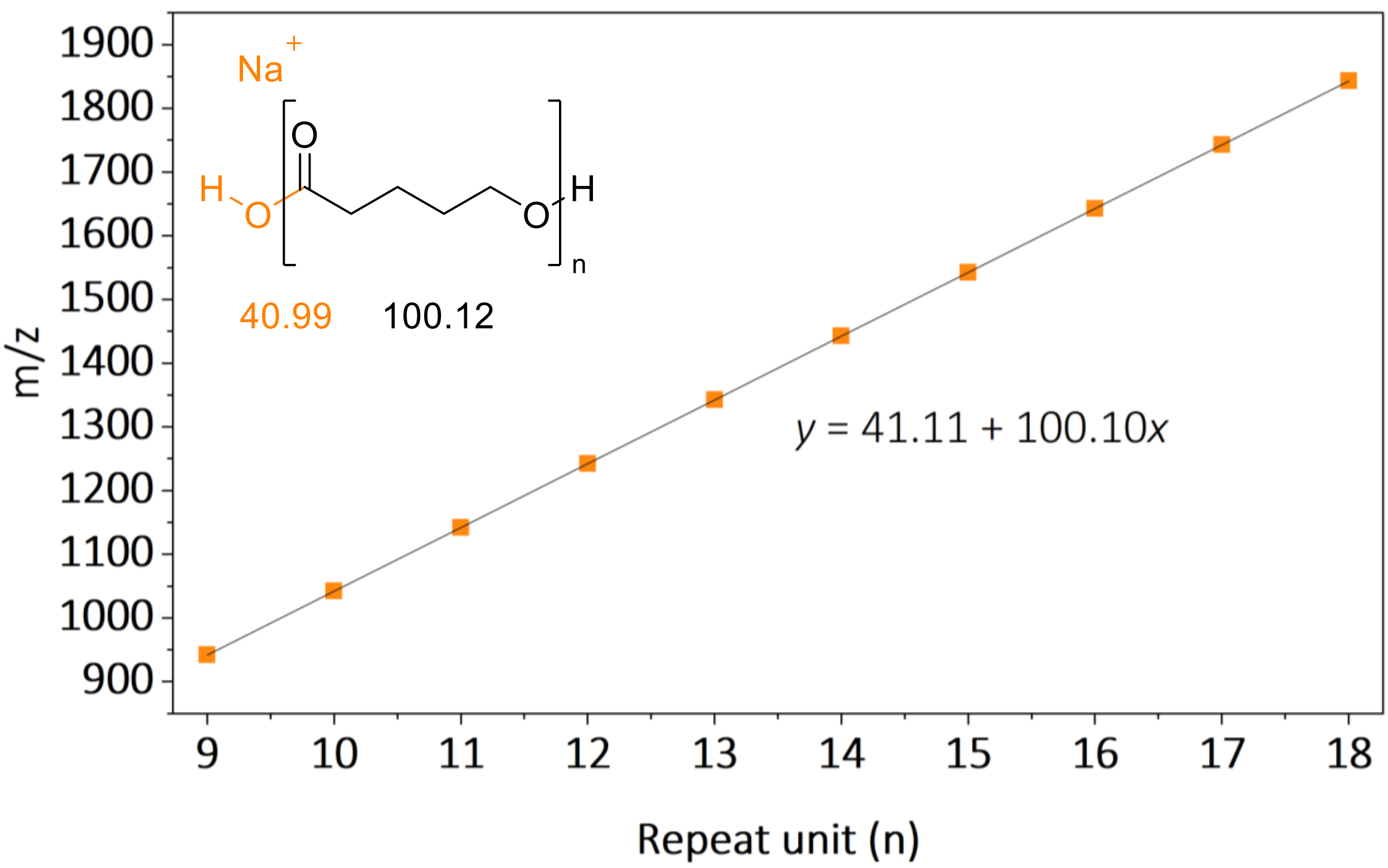

Fig. S48 m/z *vs*. repeat unit plot for the first series of identified peaks from MALDI-TOF analysis of PVL produced using the Bi(Oct)$_3$ catalyst, where the slope gives the repeat unit and the intercept gives the end group molar mass.

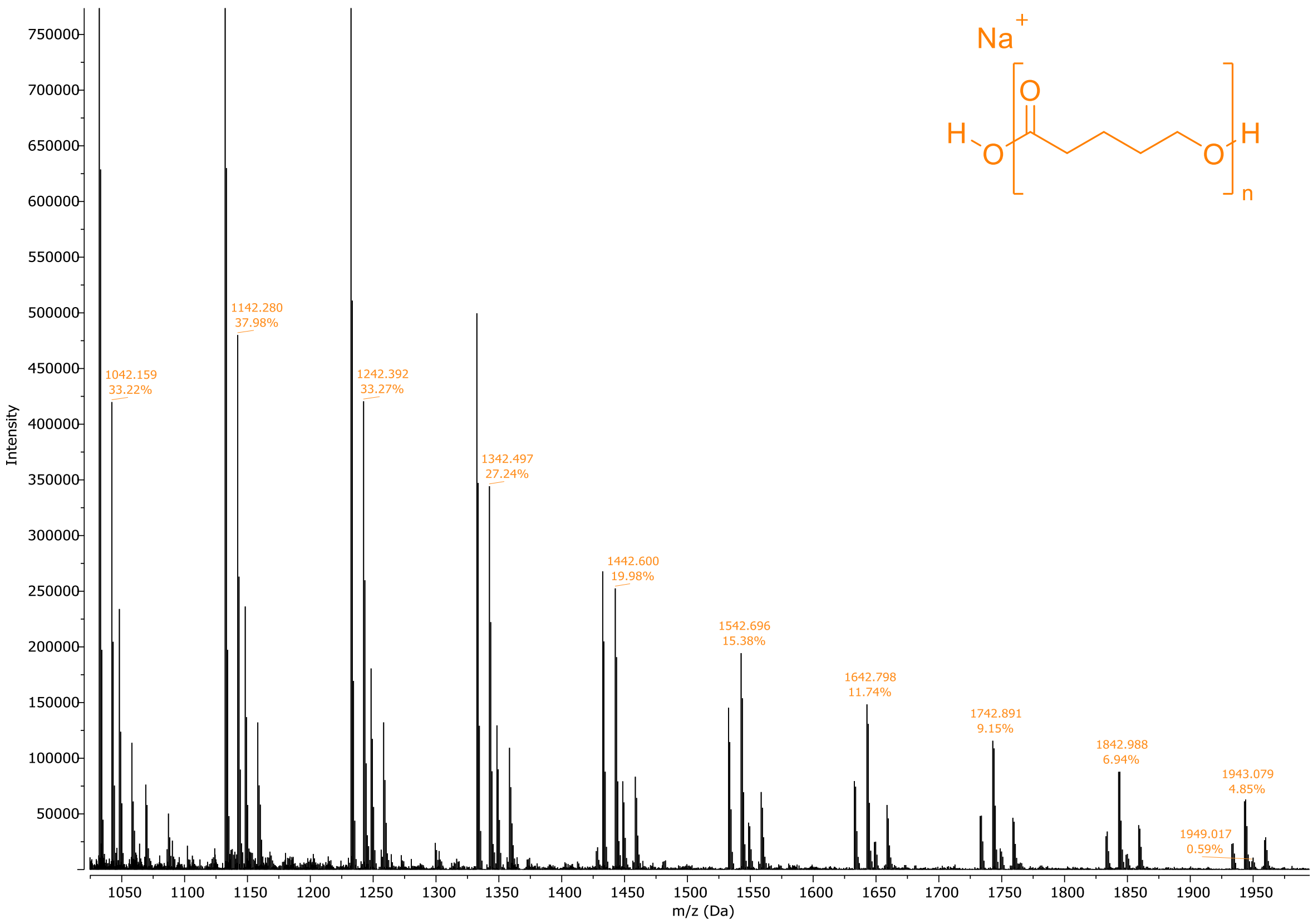

Fig. S49 MALDI-TOF Spectrum for PVL produced using Bi(Oct)$_3$ catalyst, focusing on peaks identified as PVL with a hydroxyl end group (doped with Na$^+$). The polymer (10 mg mL^{-1} in THF), and dithranol (10 mg mL^{-1} in THF) were premixed in a 1:25 ratio (v/v).

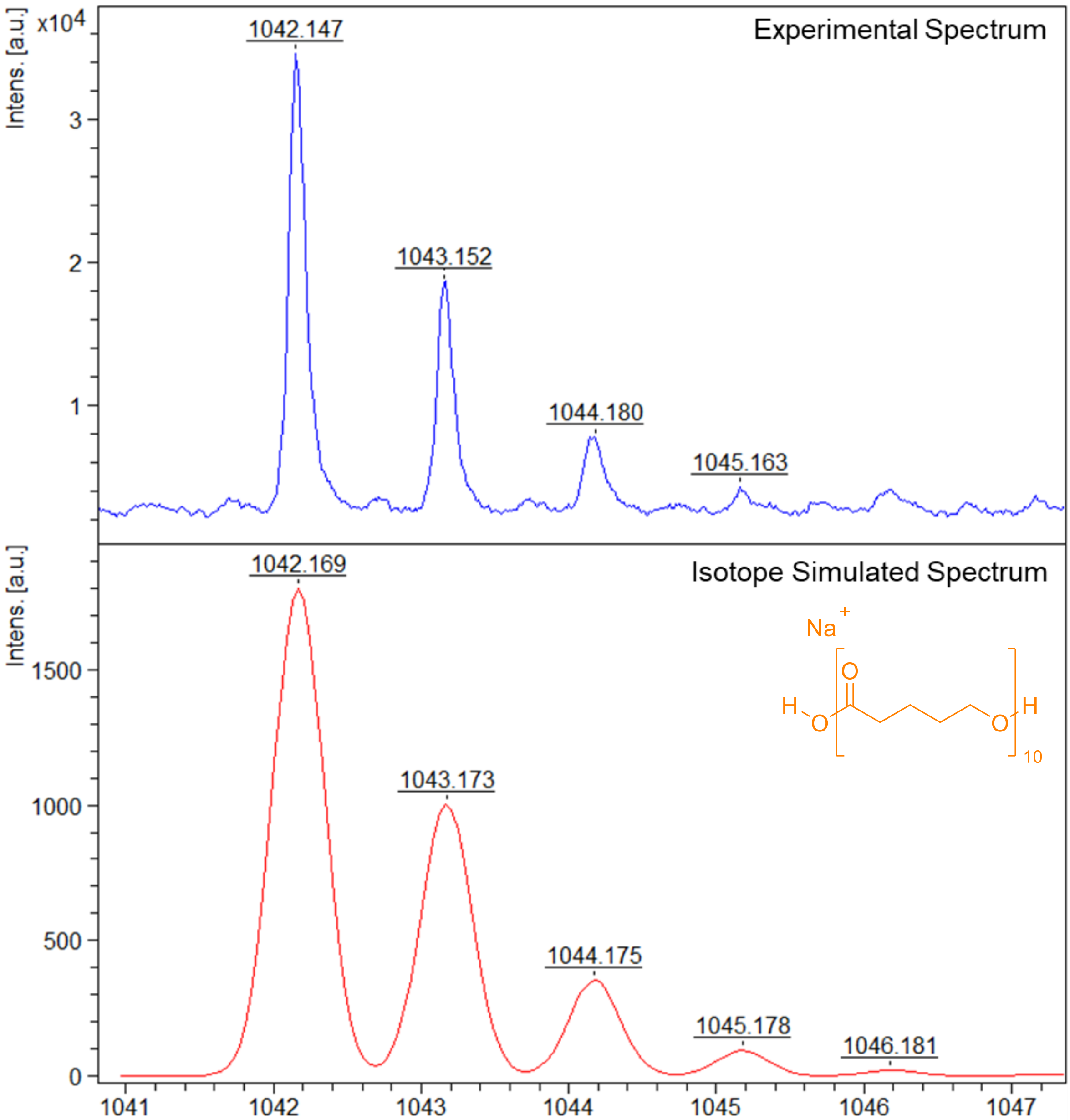

Fig. S50 The experimental spectrum stacked with an isotope simulated spectrum for $HO[C_5H_8O_2]_{10}H$, Na^+ m/z = 1042.17.

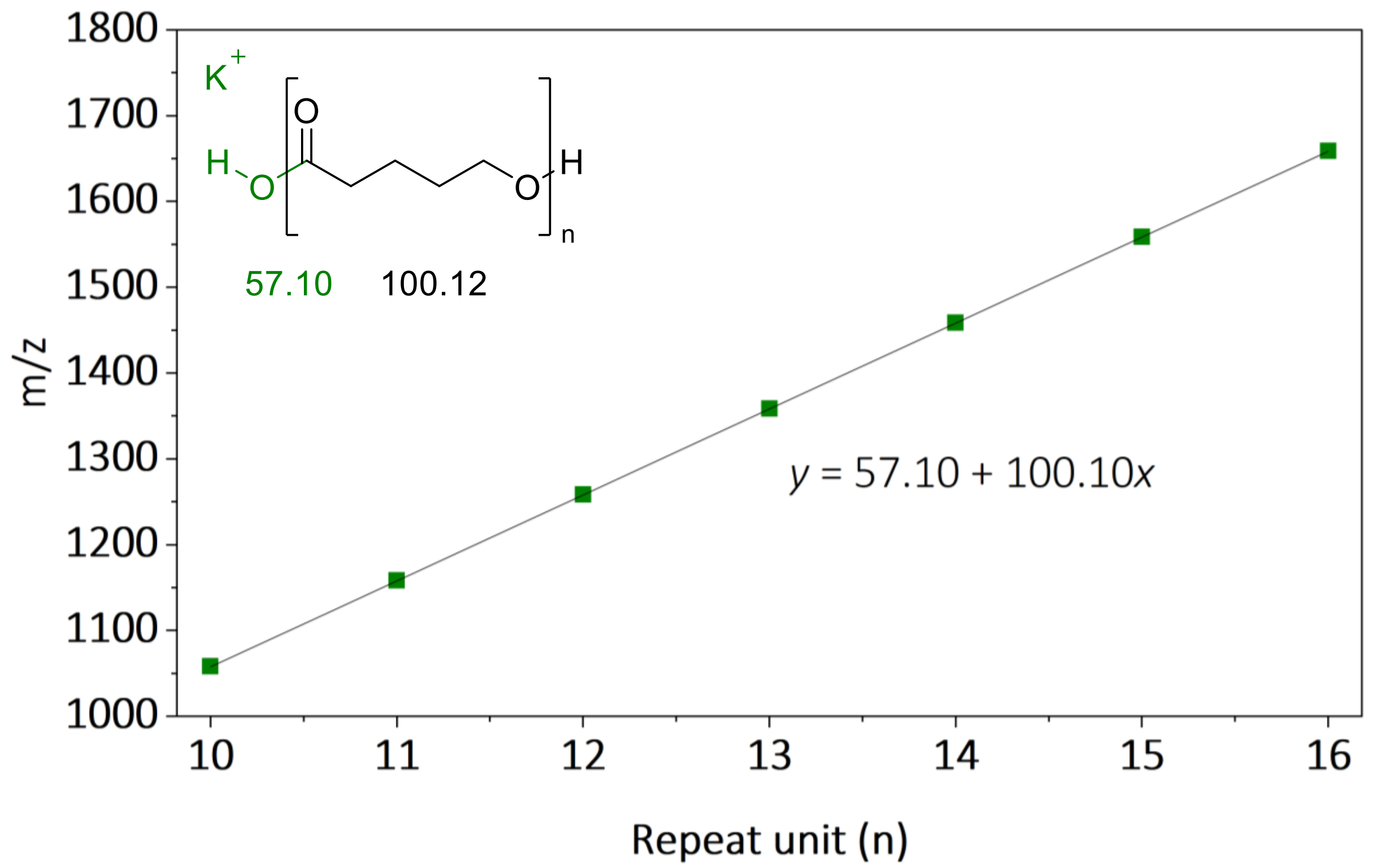

Fig. S51 m/z *vs*. repeat unit plot for the second series of identified peaks from MALDI-TOF analysis of PVL produced using the $Bi(Oct)_3$ catalyst, where the slope gives the repeat unit and the intercept gives the end group molar mass.

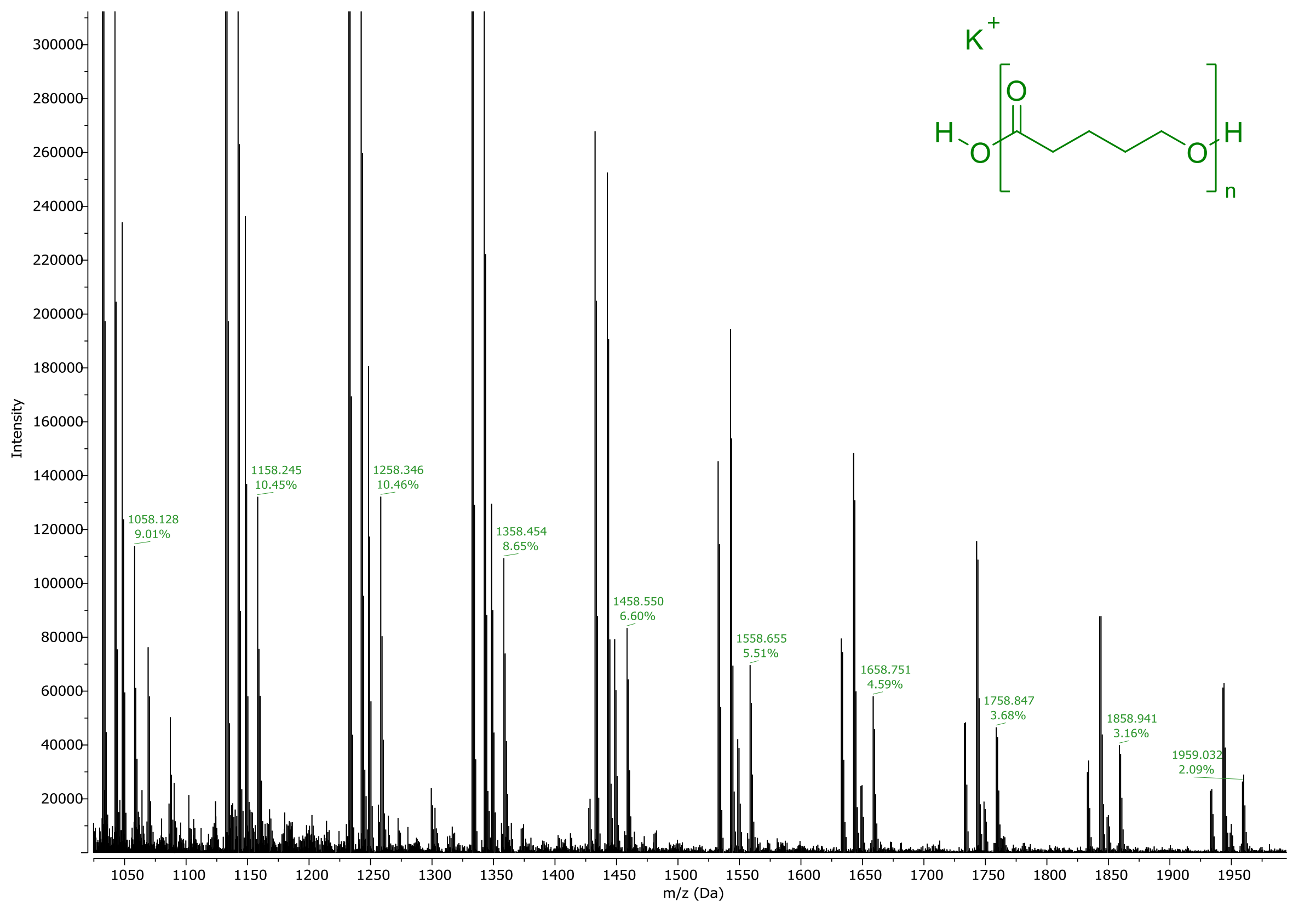

Fig. S52 MALDI-TOF Spectrum for PVL produced using $Bi(Oct)_3$ catalyst, focusing on peaks identified as PVL with a hydroxyl end group (doped with K^+). The polymer (10 mg mL^{-1} in THF), and dithranol (10 mg mL^{-1} in THF) were premixed in a 1:25 ratio (v/v).

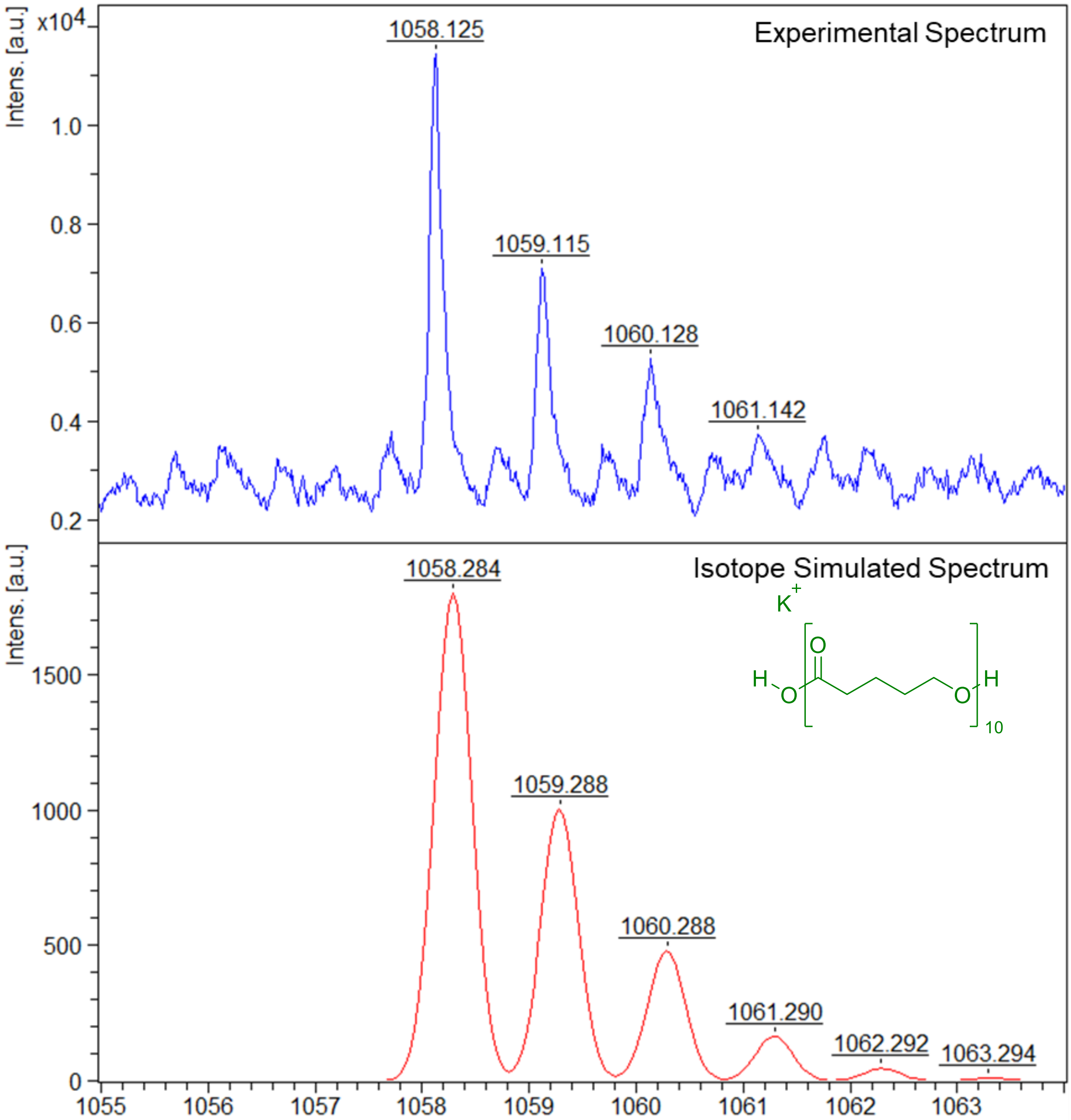

Fig. S53 The experimental spectrum stacked with an isotope simulated spectrum for HO[$C_5H_8O_2$]$_{10}$H, K^+ m/z = 1058.28.

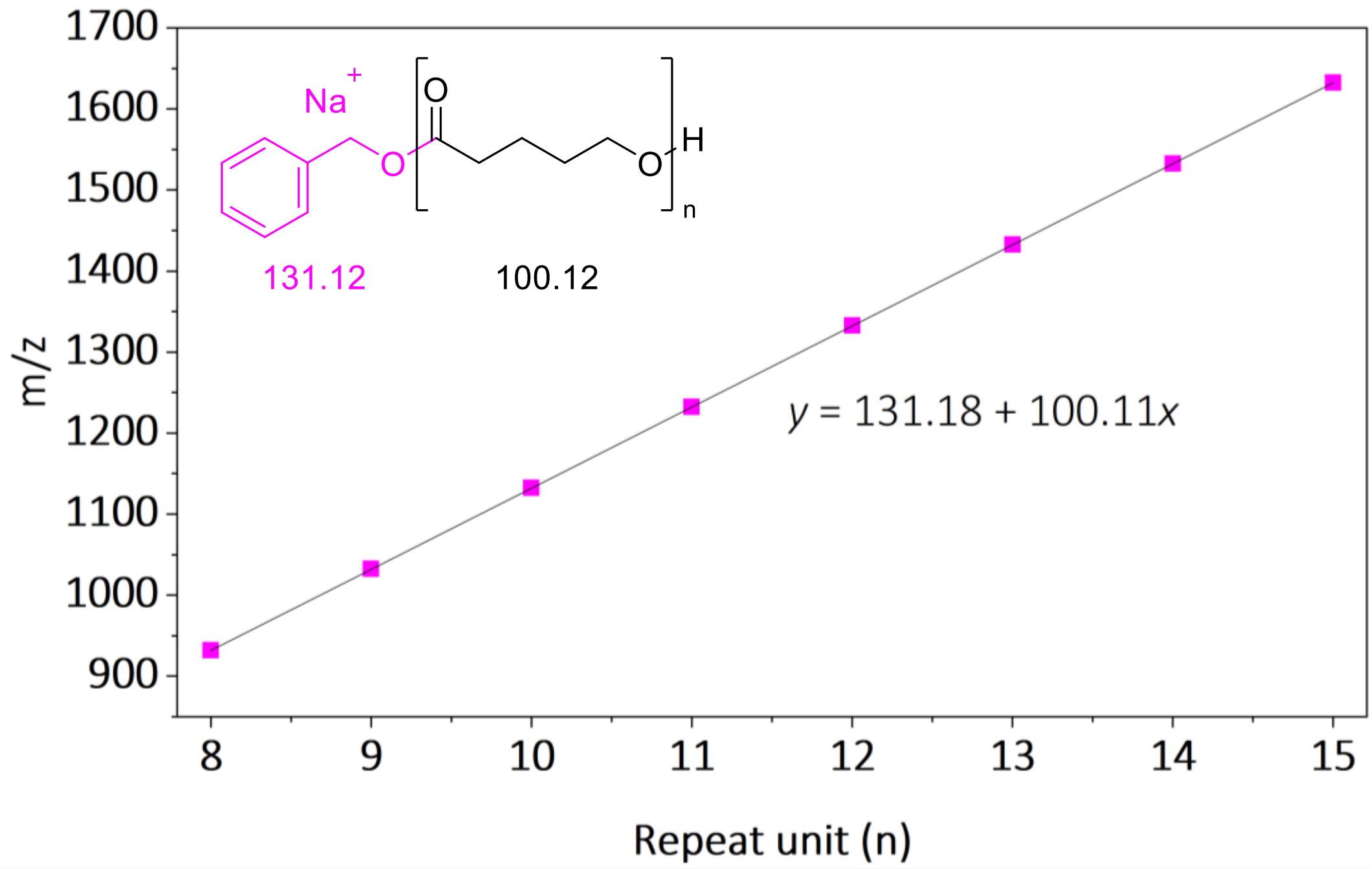

Fig. S54 m/z *vs*. repeat unit plot for the third series of identified peaks from MALDI-TOF analysis of PVL produced using the Bi(Oct)$_3$ catalyst, where the slope gives the repeat unit and the intercept gives the end group molar mass.

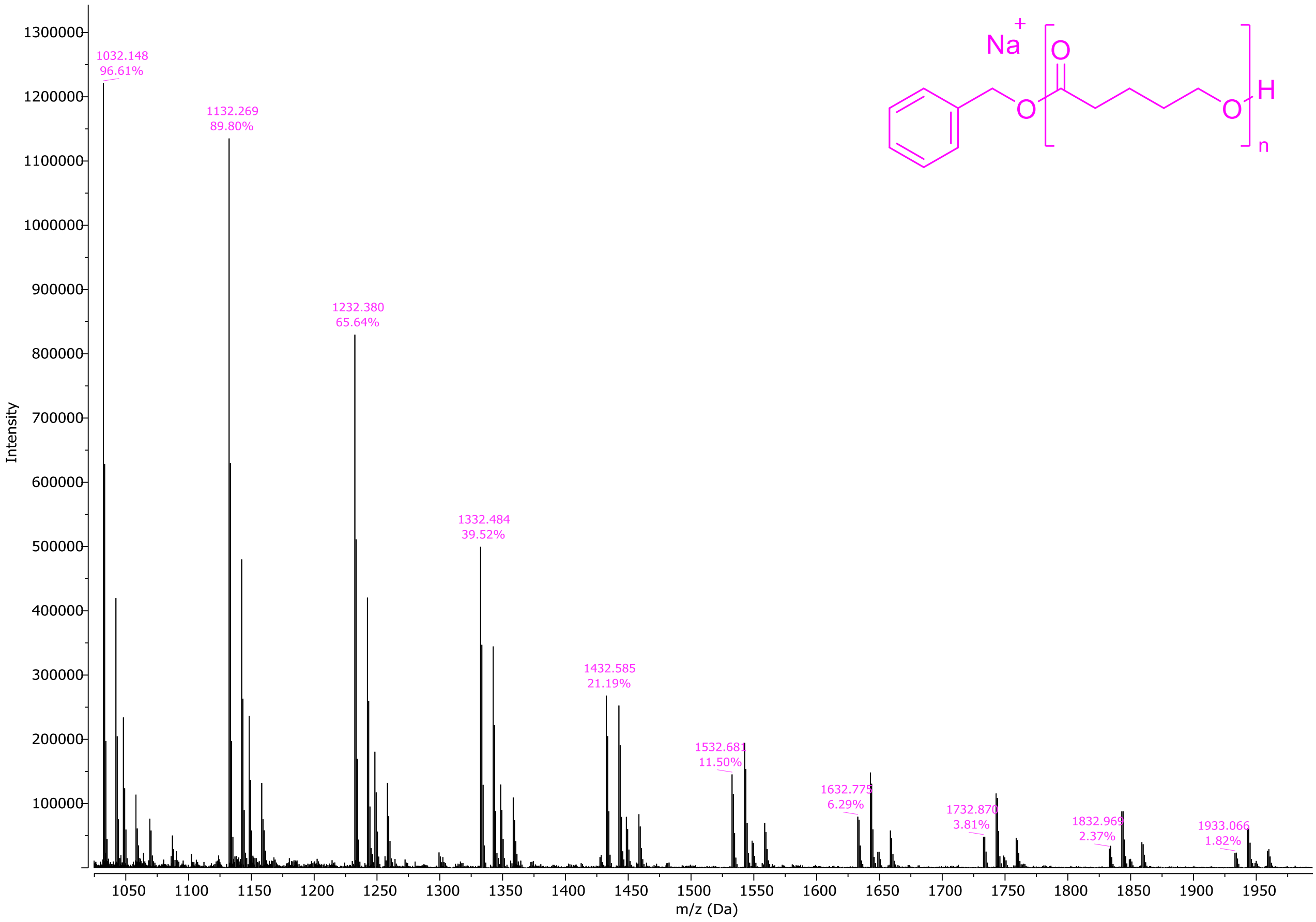

Fig. S55 MALDI-TOF Spectrum for PVL produced using Bi(Oct)$_3$ catalyst, focusing on peaks identified as PVL with a benzyl alcohol end group (doped with Na$^+$). The polymer (10 mg mL^{-1} in THF), and dithranol (10 mg mL^{-1} in THF) were premixed in a 1:25 ratio (v/v).

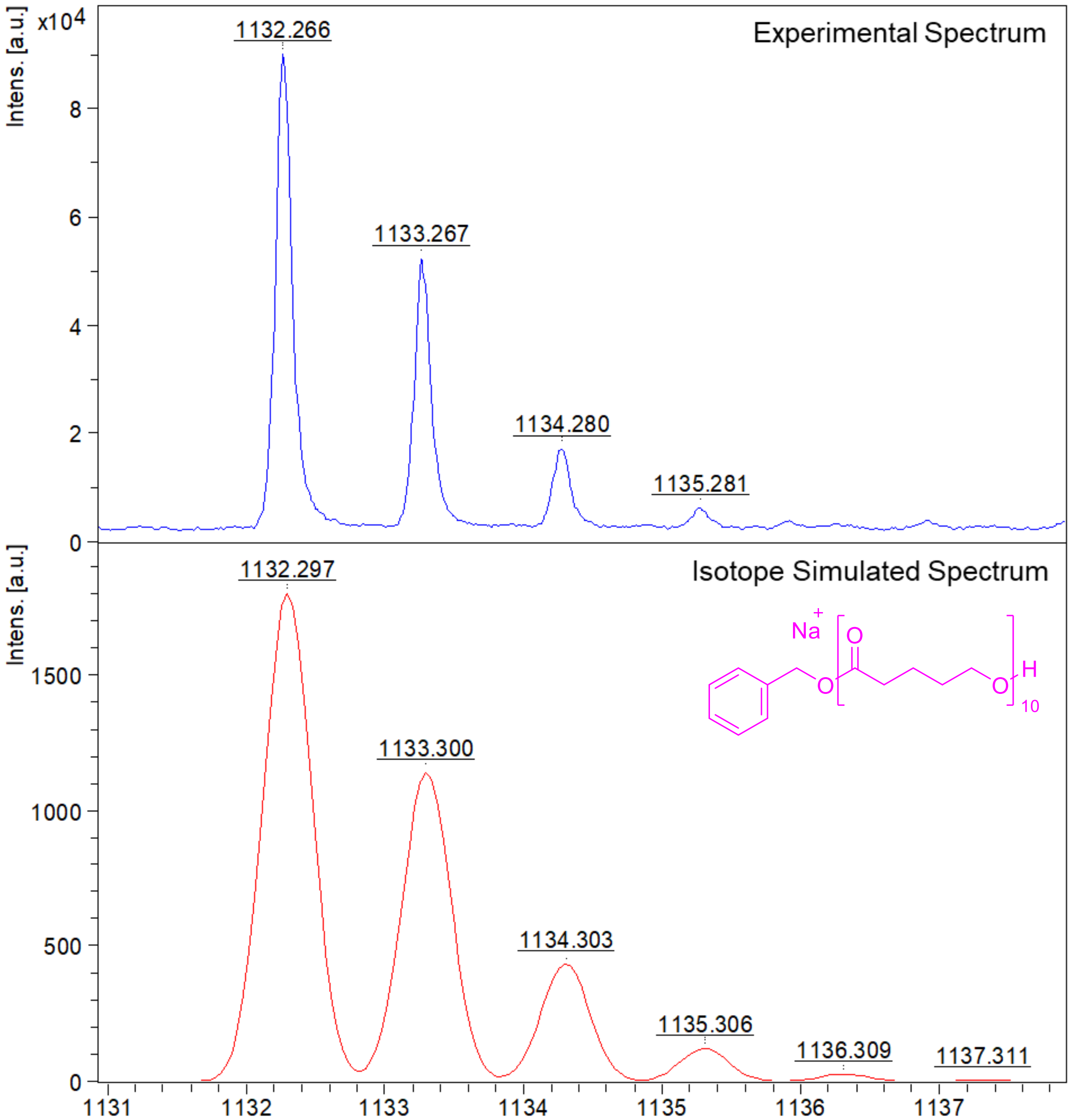

Fig. S56 The experimental spectrum stacked with an isotope simulated spectrum for $C_7H_8O[C_5H_8O_2]_{10}H$, Na^+ m/z = 1132.30.

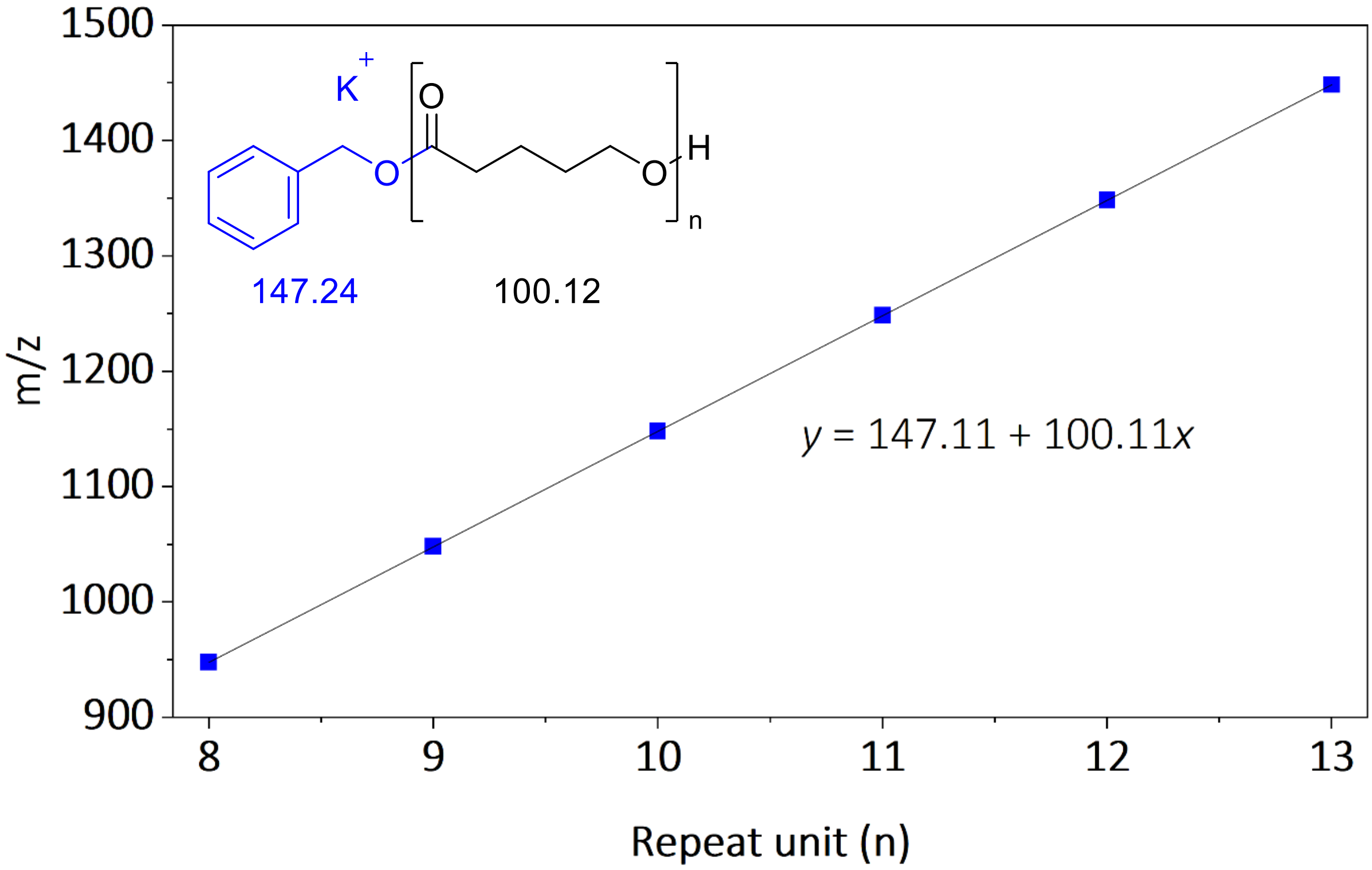

Fig. S57 m/z *vs*. repeat unit plot for the fourth series of identified peaks from MALDI-TOF analysis of PVL produced using the $Bi(Oct)_3$ catalyst, where the slope gives the repeat unit and the intercept gives the end group molar mass.

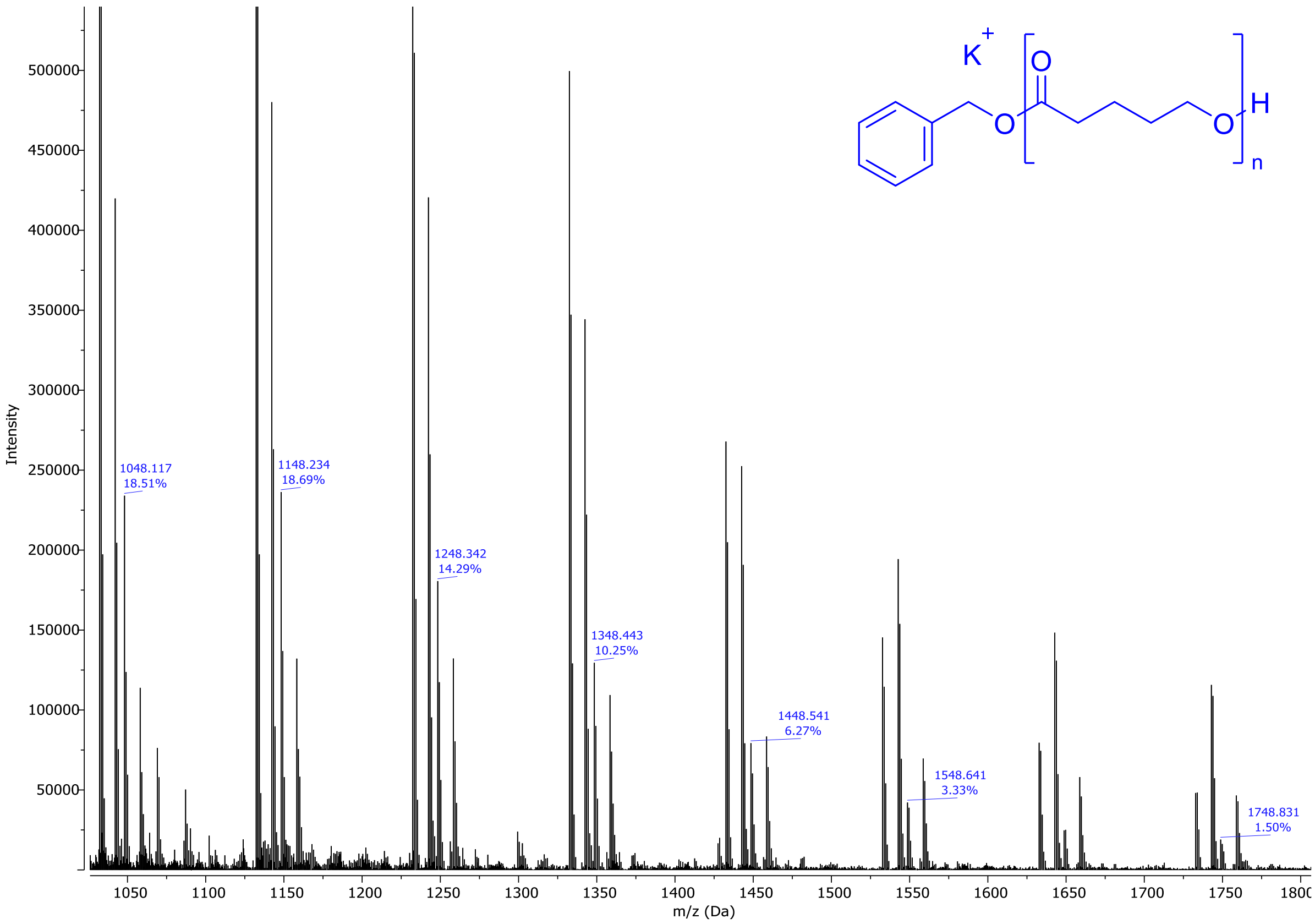

Fig. S58 MALDI-TOF Spectrum for PVL produced using $Bi(Oct)_3$ catalyst, focusing on peaks identified as PVL with a benzyl alcohol end group (doped with K^+). The polymer (10 mg mL^{-1} in THF), and dithranol (10 mg mL^{-1} in THF) were premixed in a 1:25 ratio (v/v).

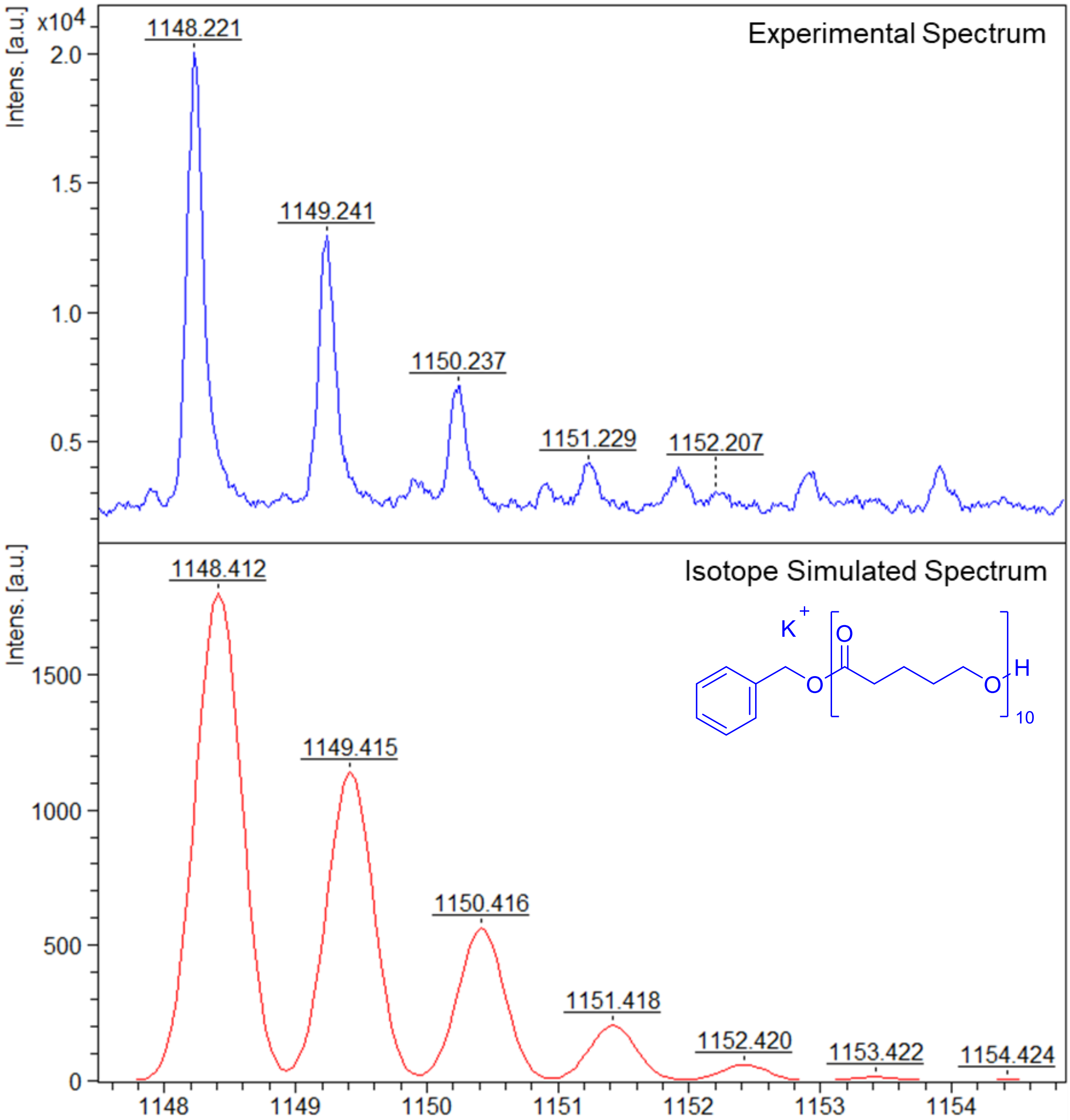

Fig. S59 The experimental spectrum stacked with an isotope simulated spectrum for $C_7H_8O[C_5H_8O_2]_{10}H$, K^+ m/z = 1148.41.

To investigate the order in $Bi(Oct)_3$, a series of polymerizations with varying catalyst loading (x) were completed using the CL monomer at 200 °C (Table S9). Each reaction was run in triplicate, with an average k_{obs} determined as the average of three runs, and errors determined from the standard deviation of the mean of the three repeats. An example of this for the polymerization conducted using 0.5 equivalents of catalyst is shown in Fig. S60.

Table S9 k_{obs} determined at incrementally increasing catalyst loadings for the polymerization of CL using the $Bi(Oct)_3$ catalyst and BnOH chain-transfer agent at 200 °C.

Eq $Bi(Oct)_3$	k_{obs} /10^{-3} (s^{-1})
0.5	0.74 ± 0.02
1	0.81 ± 0.04
5	0.87 ± 0.01
10	1.13 ± 0.06
20	6.28 ± 0.27

Polymerization conditions: x:10x:1000 $Bi(Oct)_3$:BnOH:CL, 200 °C, [CL] = 6.25 M

Plotting k_{obs} *vs*. [$Bi(Oct)_3$] reveals a non-linear relationship between catalyst concentration and rate of polymerization and the logarithmic plot is also non-linear, suggesting a more complex dependence on catalyst concentration for this catalyst (Fig. S61 and S62).

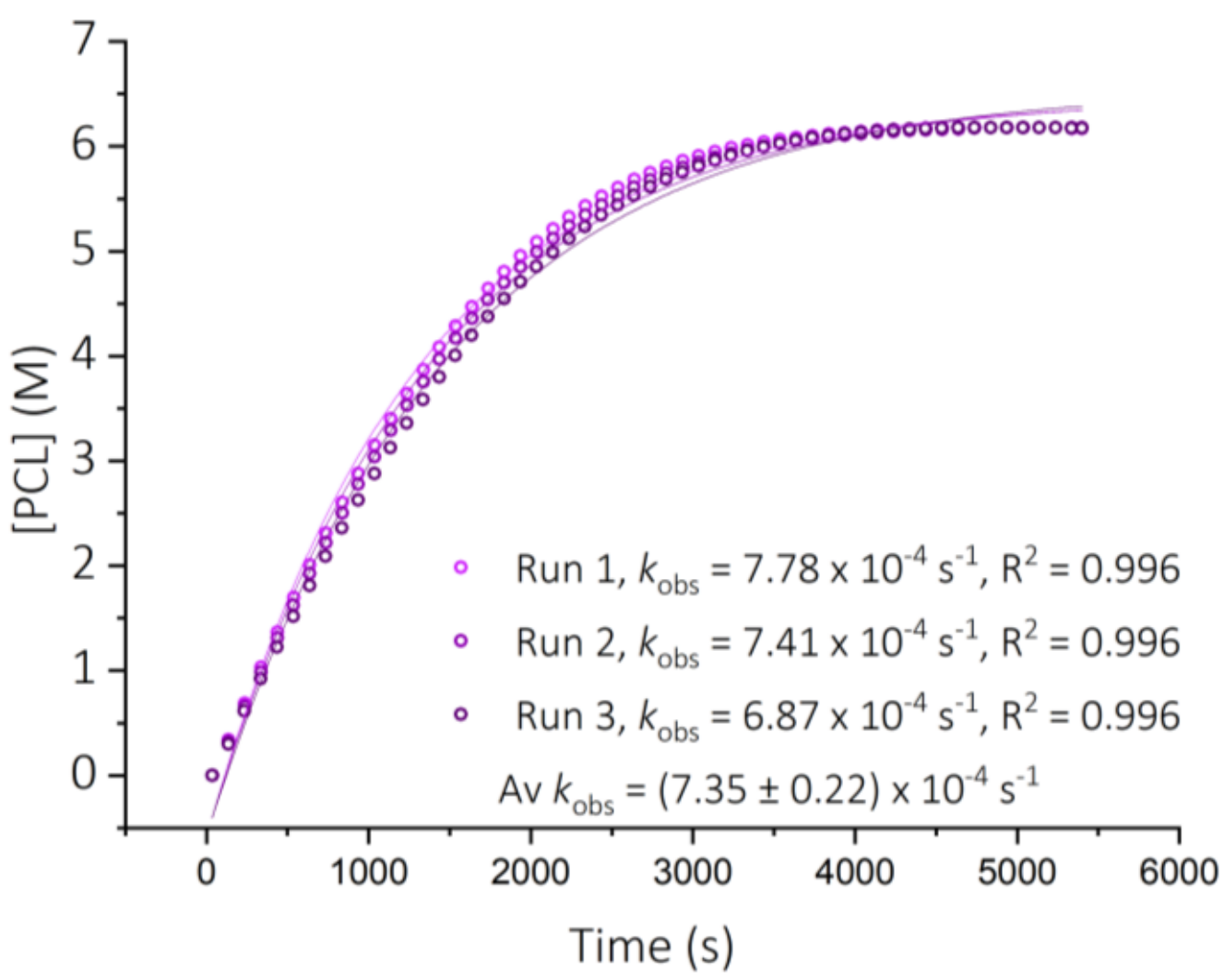

Fig. S60 Plots of [PCL] *vs*. time for isotherms conducted at 200 °C. k_{obs} is taken from the exponential fitting of each line ($y = A - Be^{-k_{obs}x}$), with the average k_{obs} taken as the mean of the three repeats and error determined from the standard deviation of the mean of the three repeats. Polymerization conditions: 0.5:5:1000 $Bi(Oct)_3$:BnOH:CL.

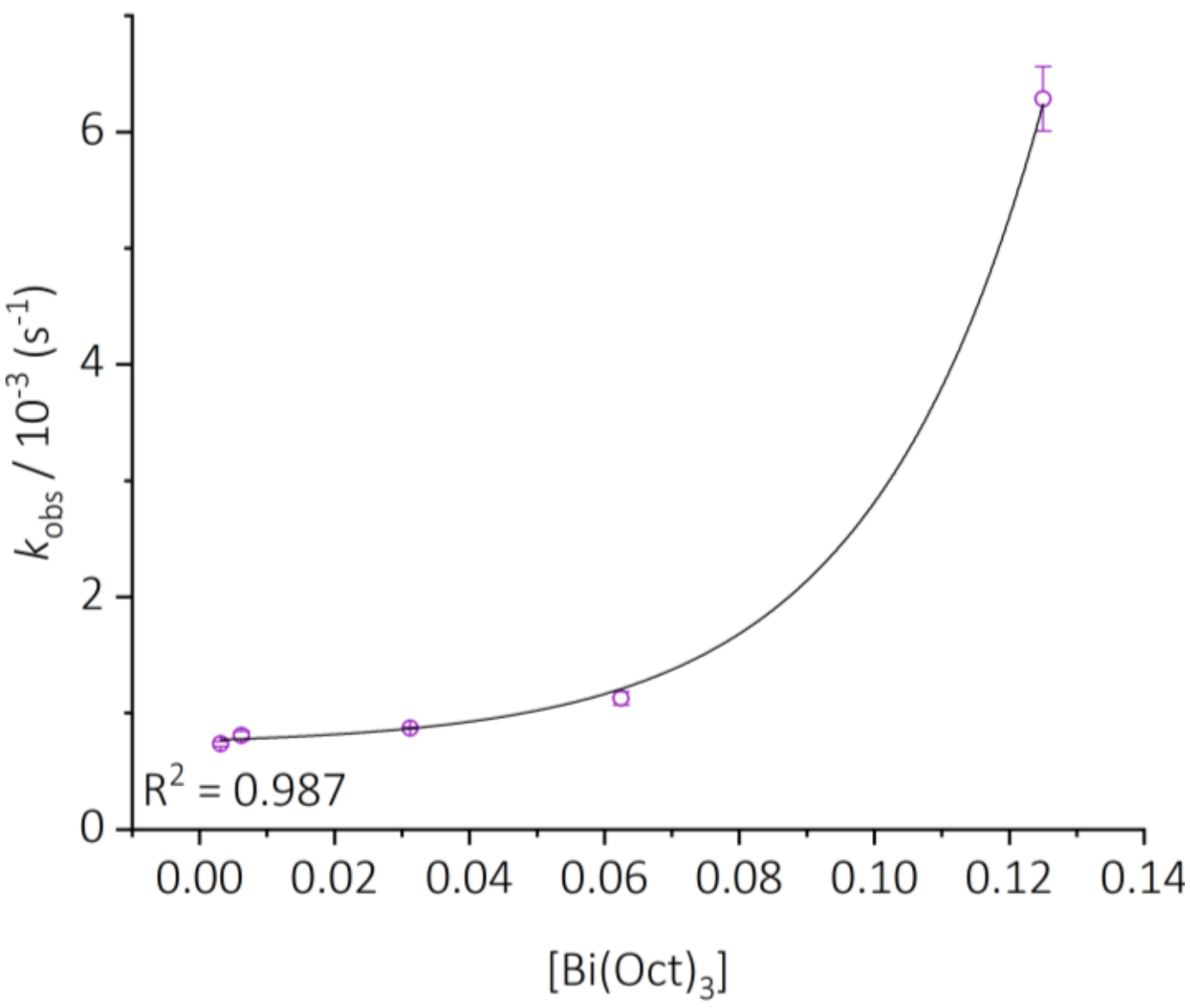

Fig. S61 Plot of k_{obs} *vs*. [Bi(Oct)$_3$] for isotherms conducted at 200 °C with polymerization conditions: x:10x:1000 Bi(Oct)$_3$:BnOH:CL where x= 0.5, 1, 5, 10, and 20, [CL] = 6.25 M.

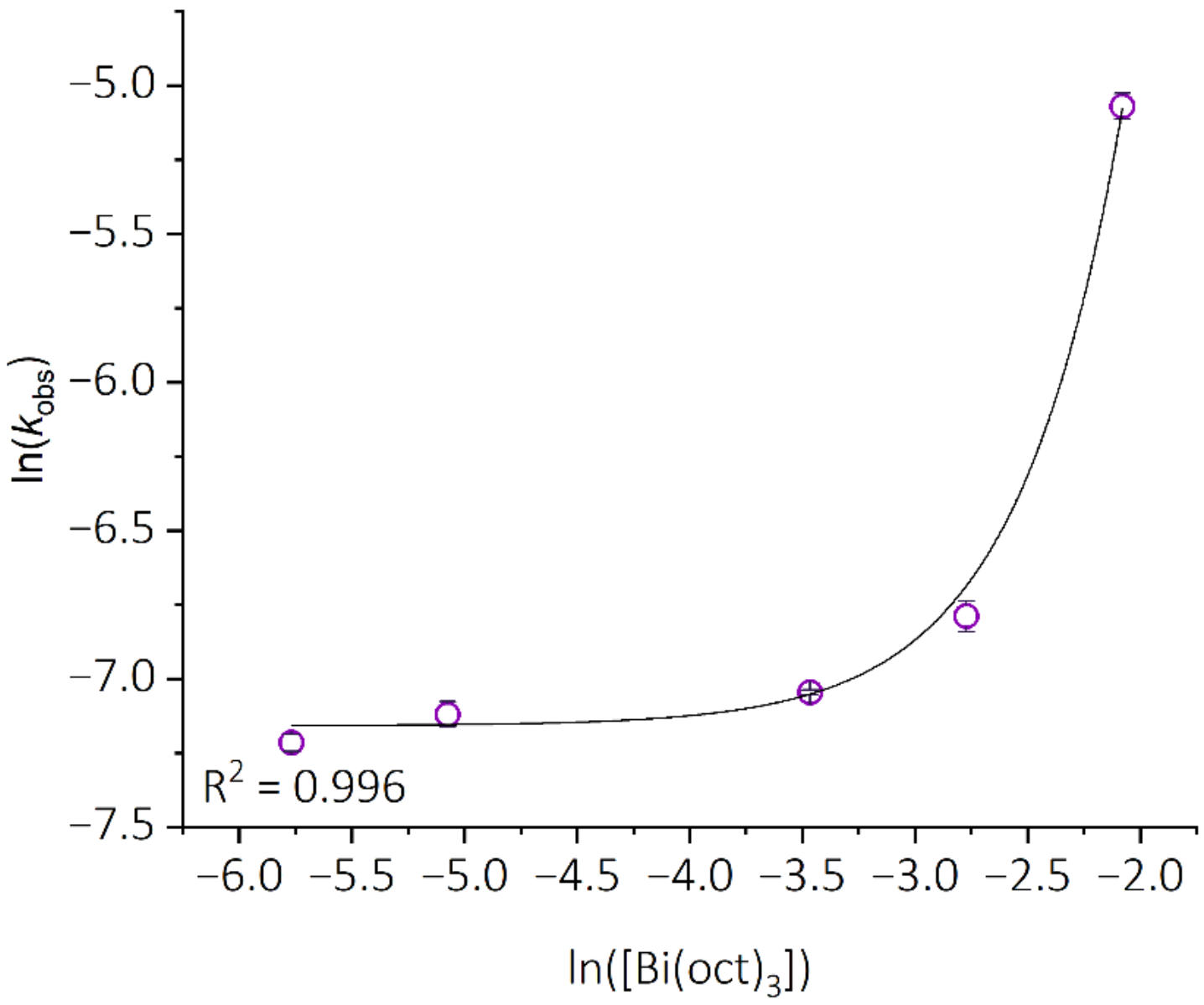

Fig. S62 Plot of ln(k_{obs}) *vs*. ln([Bi(Oct)$_3$]) for isotherms conducted at 200 °C with polymerization conditions: x:10x:1000 Bi(Oct)$_3$:BnOH:CL where x= 0.5, 1, 5, 10, and 20, [CL] = 6.25 M.

Further kinetic treatments suggest aggregation of Bi to form a less active species, as plots of $k_p^{(1-m)}$ *vs*. $[cat]_0 k_p^{-m}$ where m = estimated degree of aggregation = 2, 3, 4,… tend to a linear fit (increasing R^2) with increasing m (Fig. S63).[11]

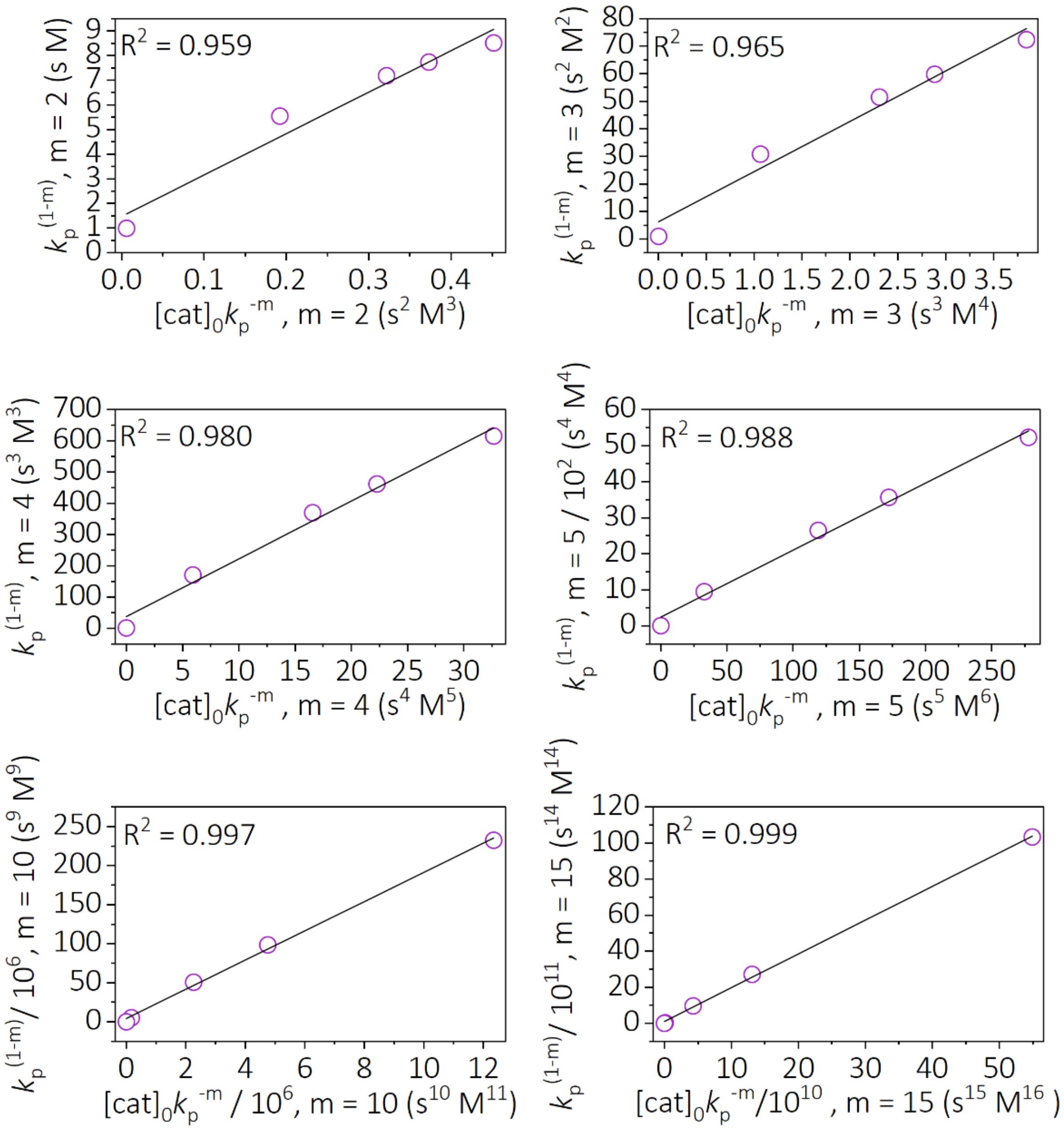

Fig. S63 Plots of $k_p^{(1\text{-}m)}$ *vs*. $[cat]_0 k_p^{-m}$ where m = estimated degree of aggregation = 2, 3, 4, 5, 10 and 15. Polymerization conditions: x:10x:1000 $Bi(Oct)_3$:BnOH:CL where x= 0.5, 1, 5, 10, and 20, [CL] = 6.25 M, 200 °C.

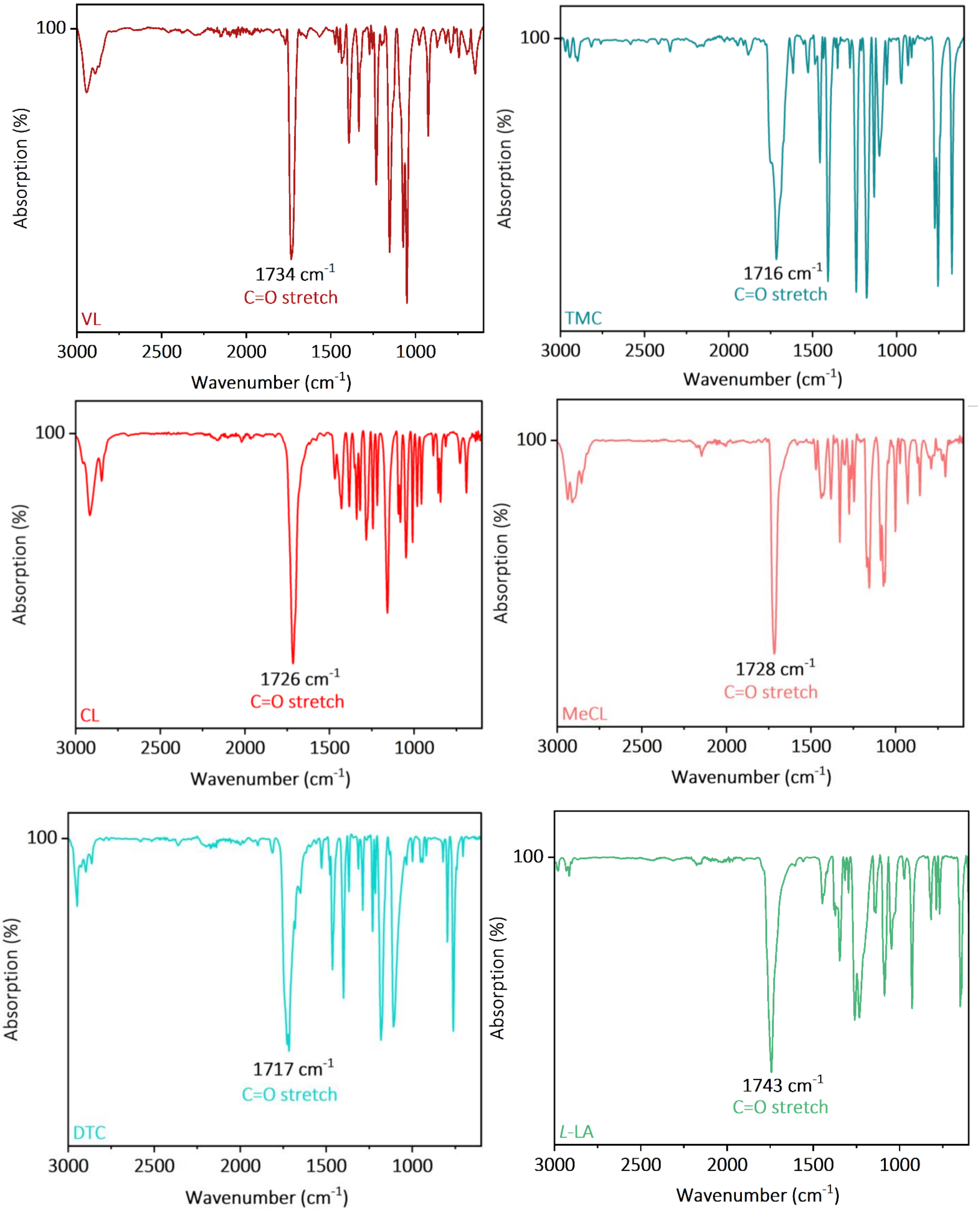

Fig. S64 IR Spectra of monomers VL, TMC, CL, MeCL, DTC and *L*-LA, indicating the C=O stretching frequency in each case.

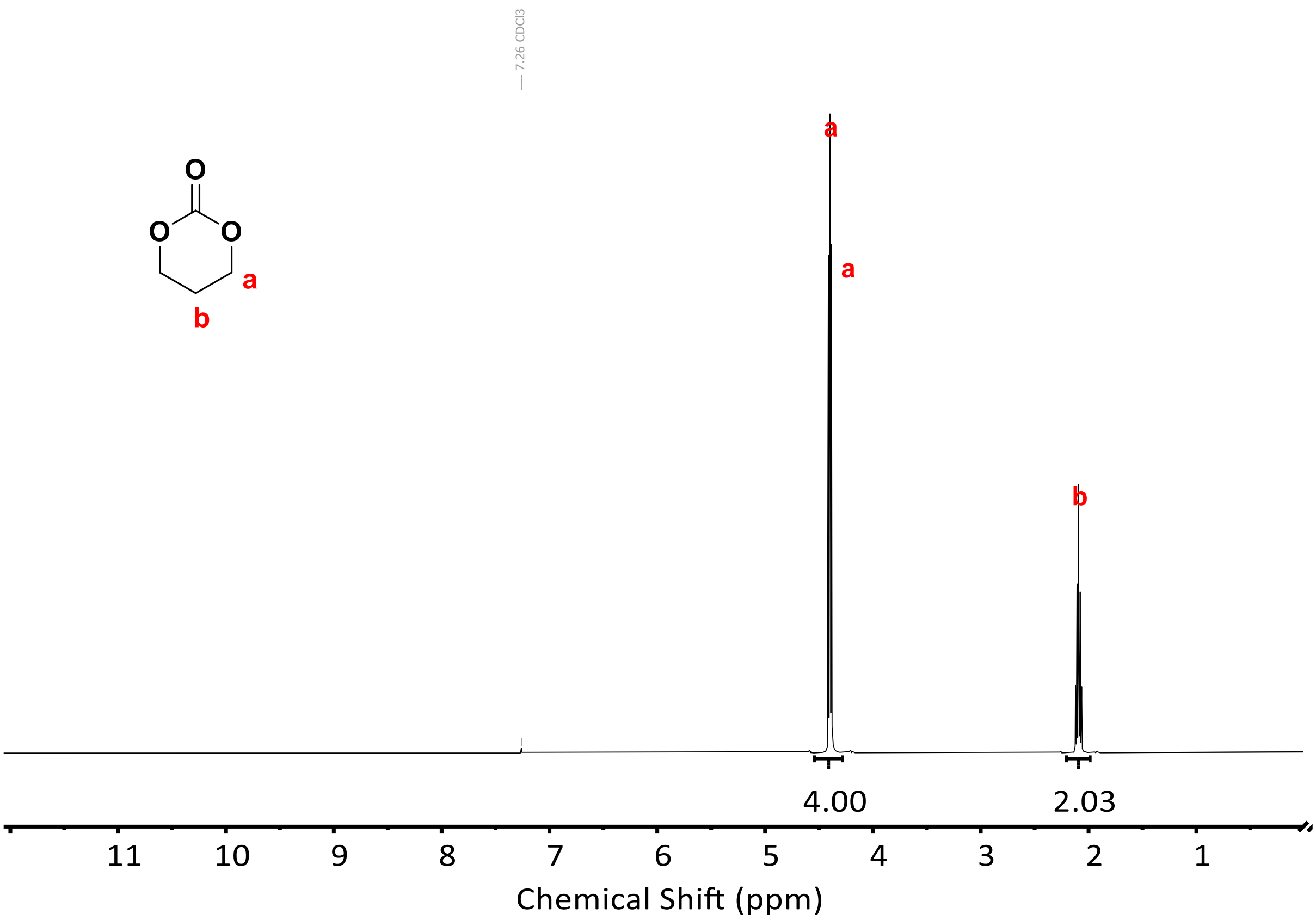

Fig. S65 ^{1}H NMR spectrum (400 MHz, $CDCl_3$, 298 K) of TMC.

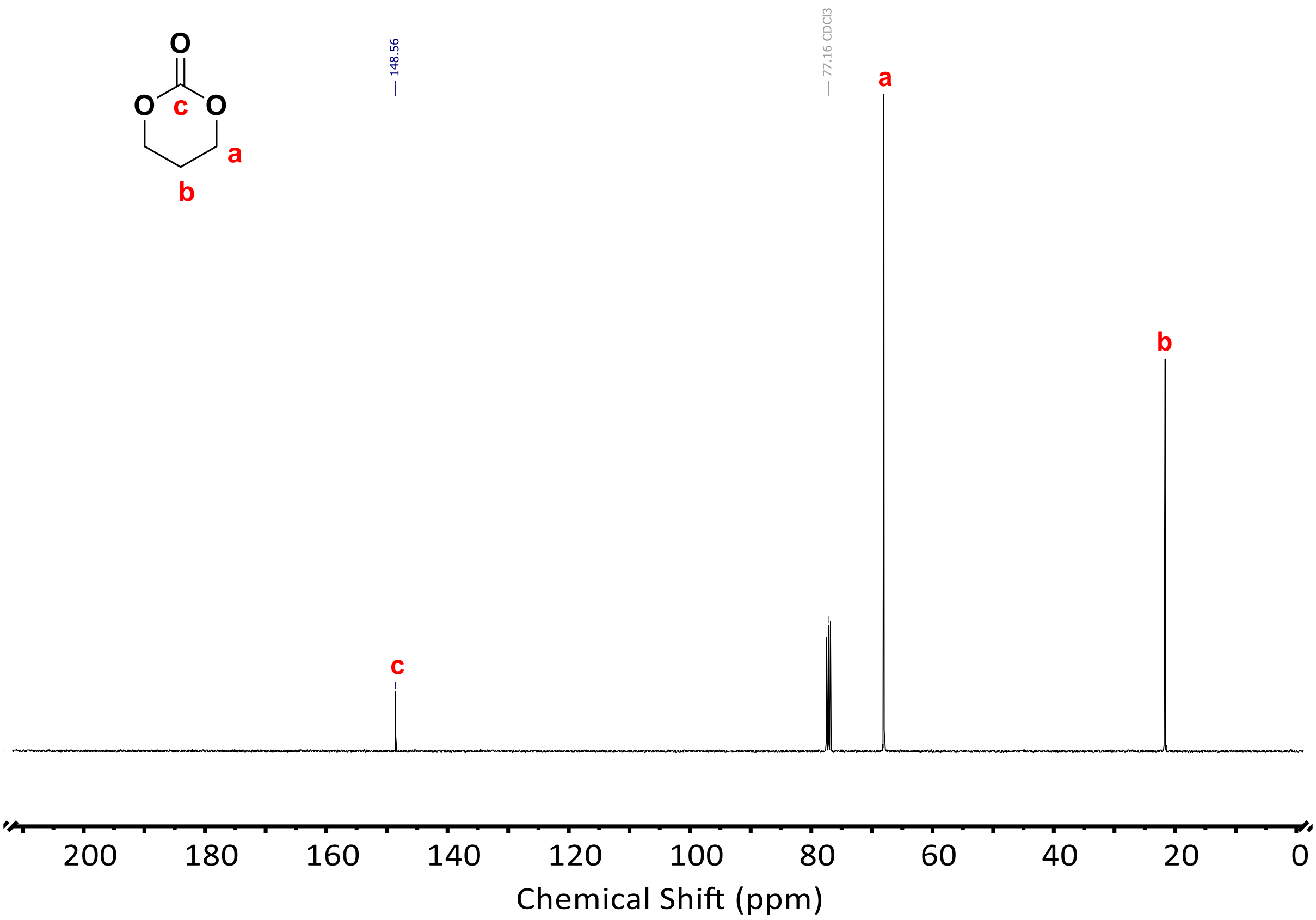

Fig. S66 $^{13}C\{^{1}H\}$ NMR spectrum (400 MHz, $CDCl_3$, 298 K) of TMC.

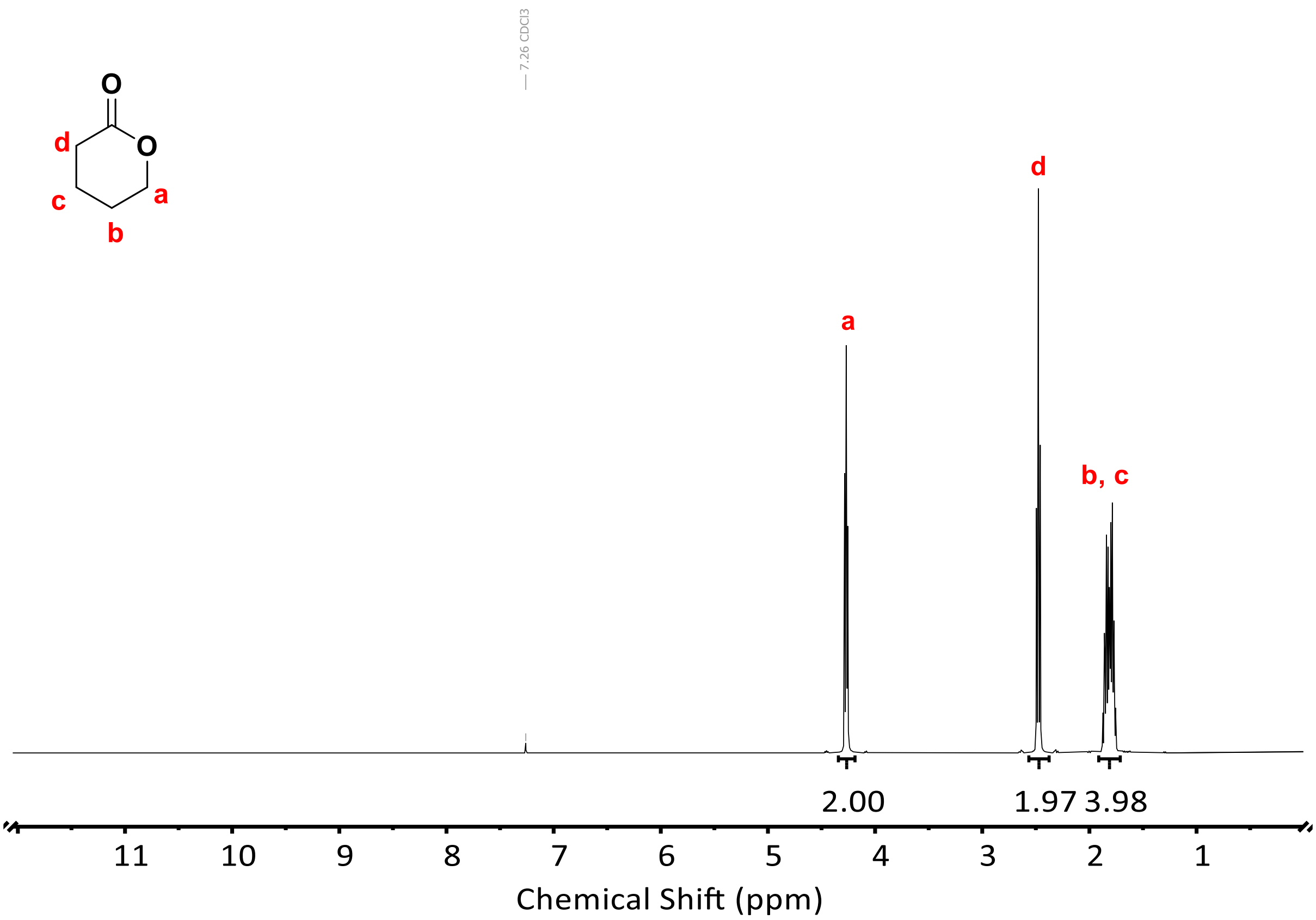

Fig. S67 ^{1}H NMR spectrum (400 MHz, $CDCl_3$, 298 K) of VL.

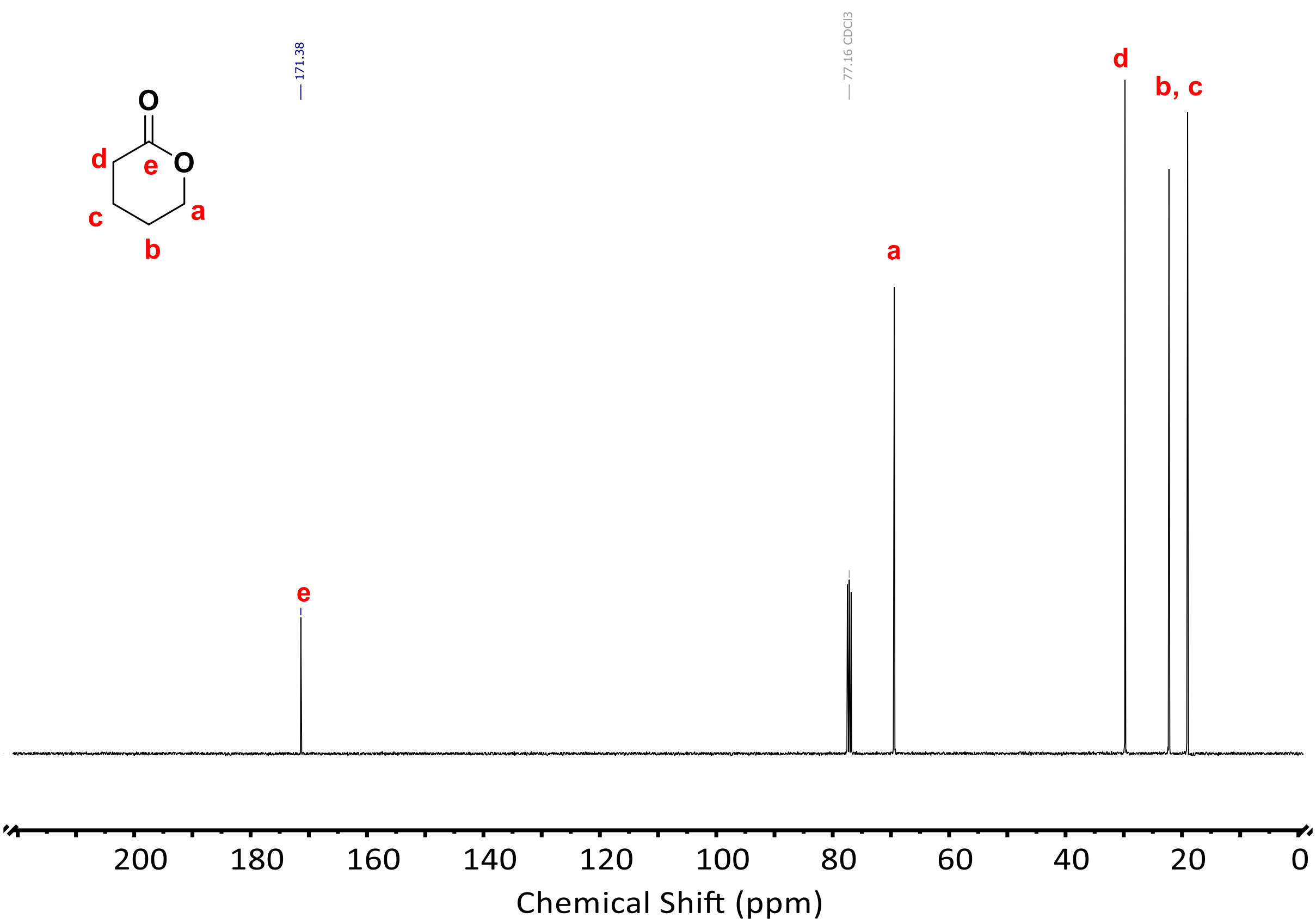

Fig. S68 $^{13}C\{^{1}H\}$ NMR spectrum (400 MHz, $CDCl_3$, 298 K) of VL.

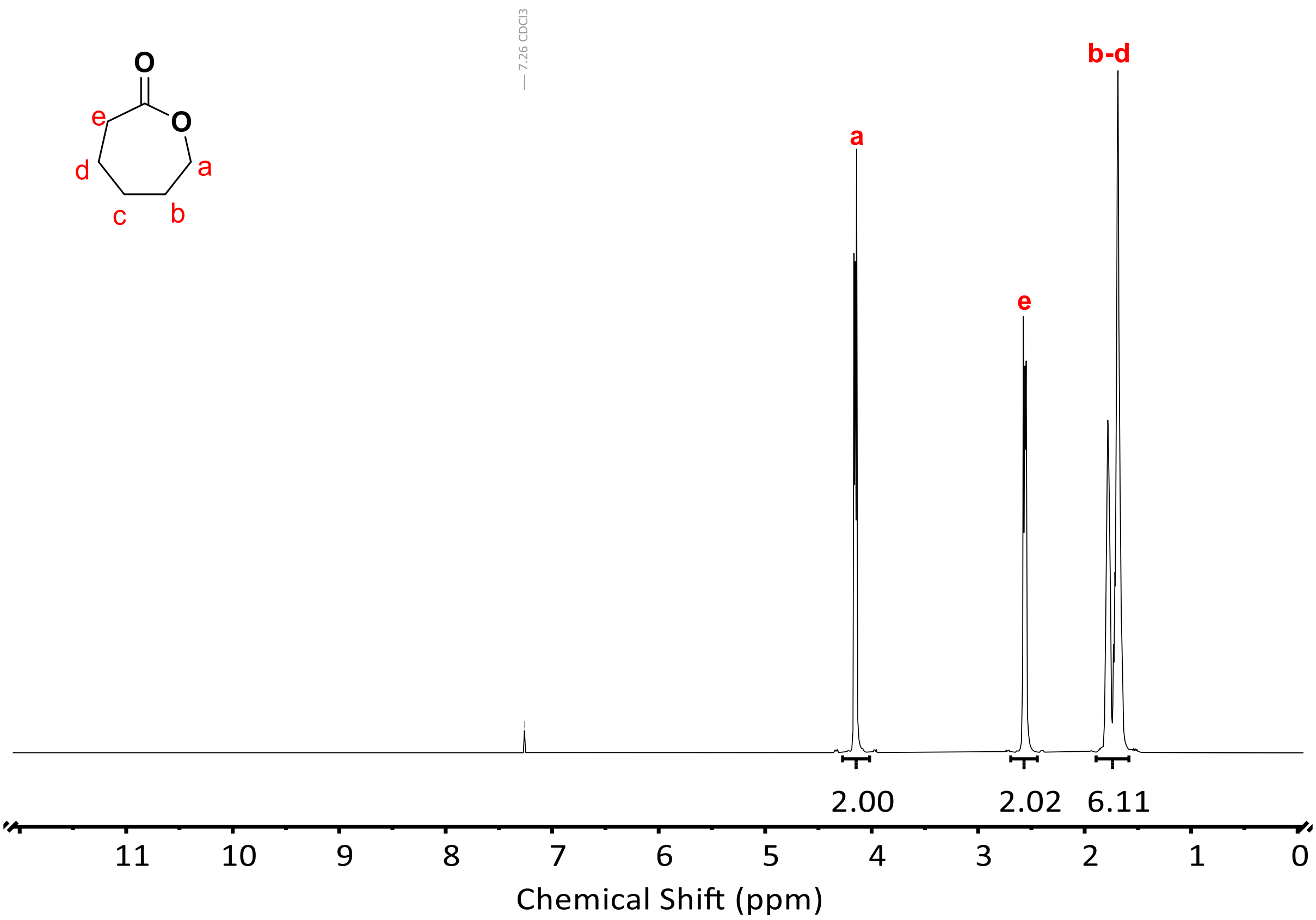

Fig. S69 ^{1}H NMR spectrum (400 MHz, $CDCl_3$, 298 K) of CL.

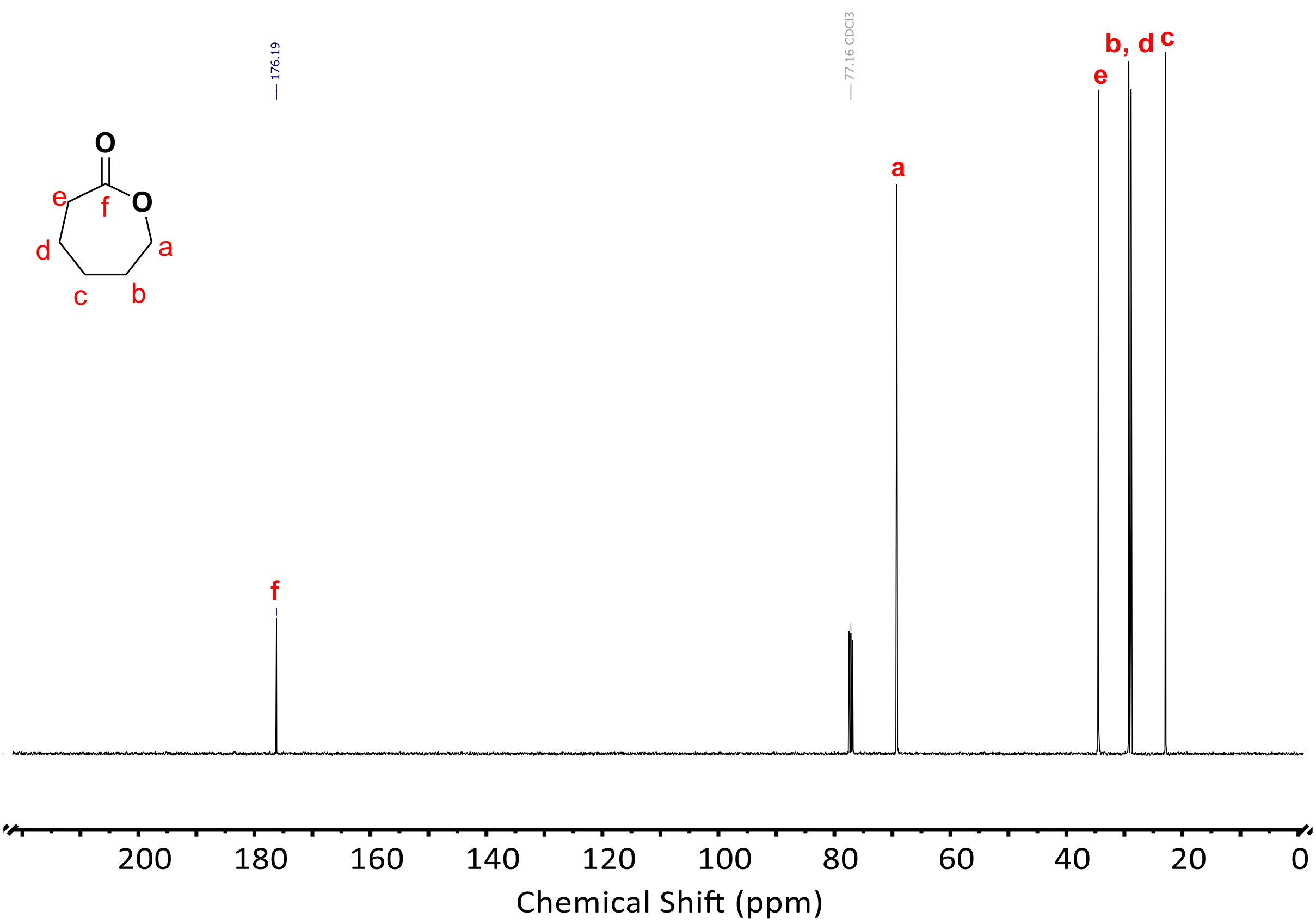

Fig. S70 $^{13}C\{^{1}H\}$ NMR spectrum (400 MHz, $CDCl_3$, 298 K) of CL.

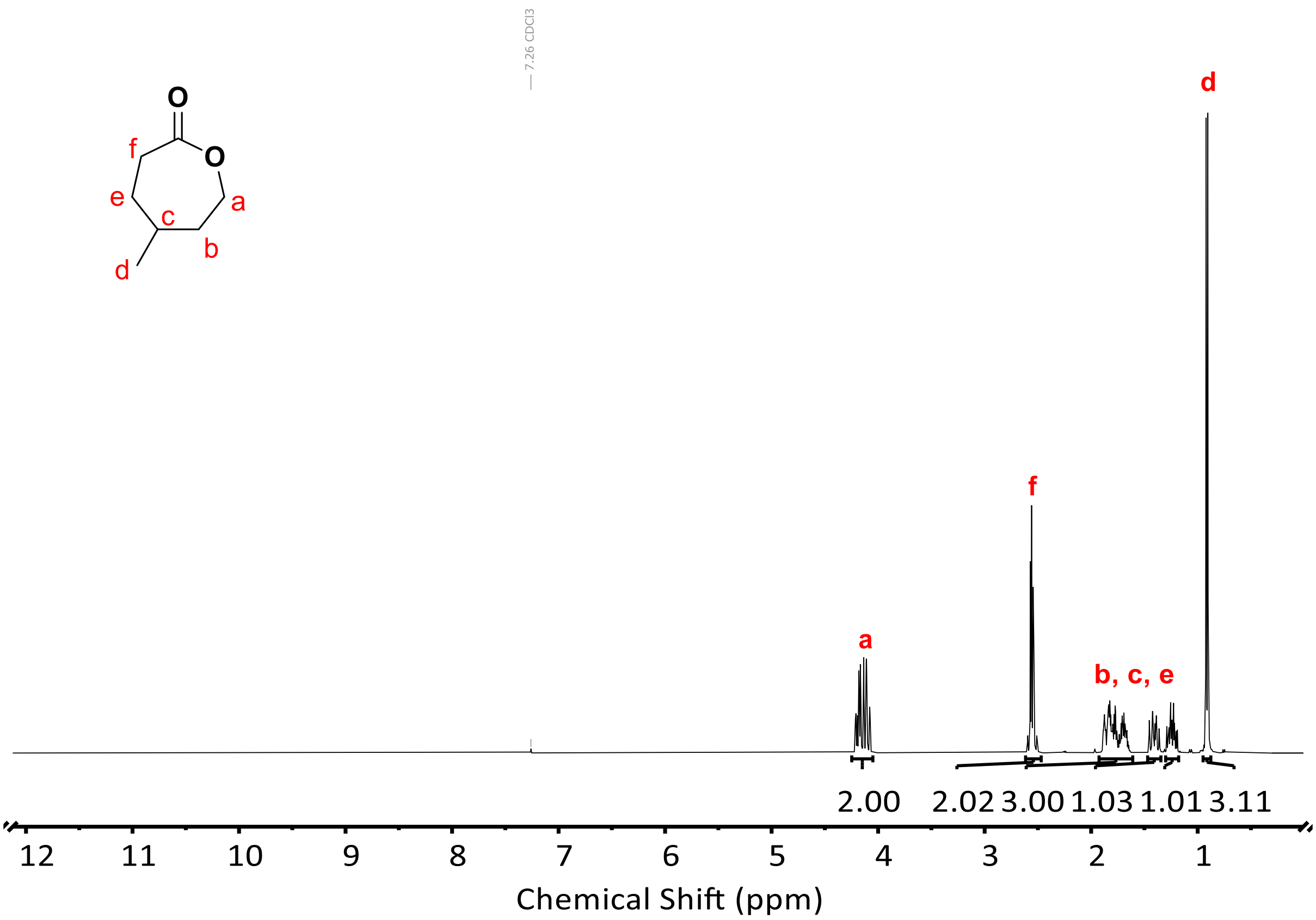

Fig. S71 ^{1}H NMR spectrum (400 MHz, $CDCl_3$, 298 K) of MeCL.

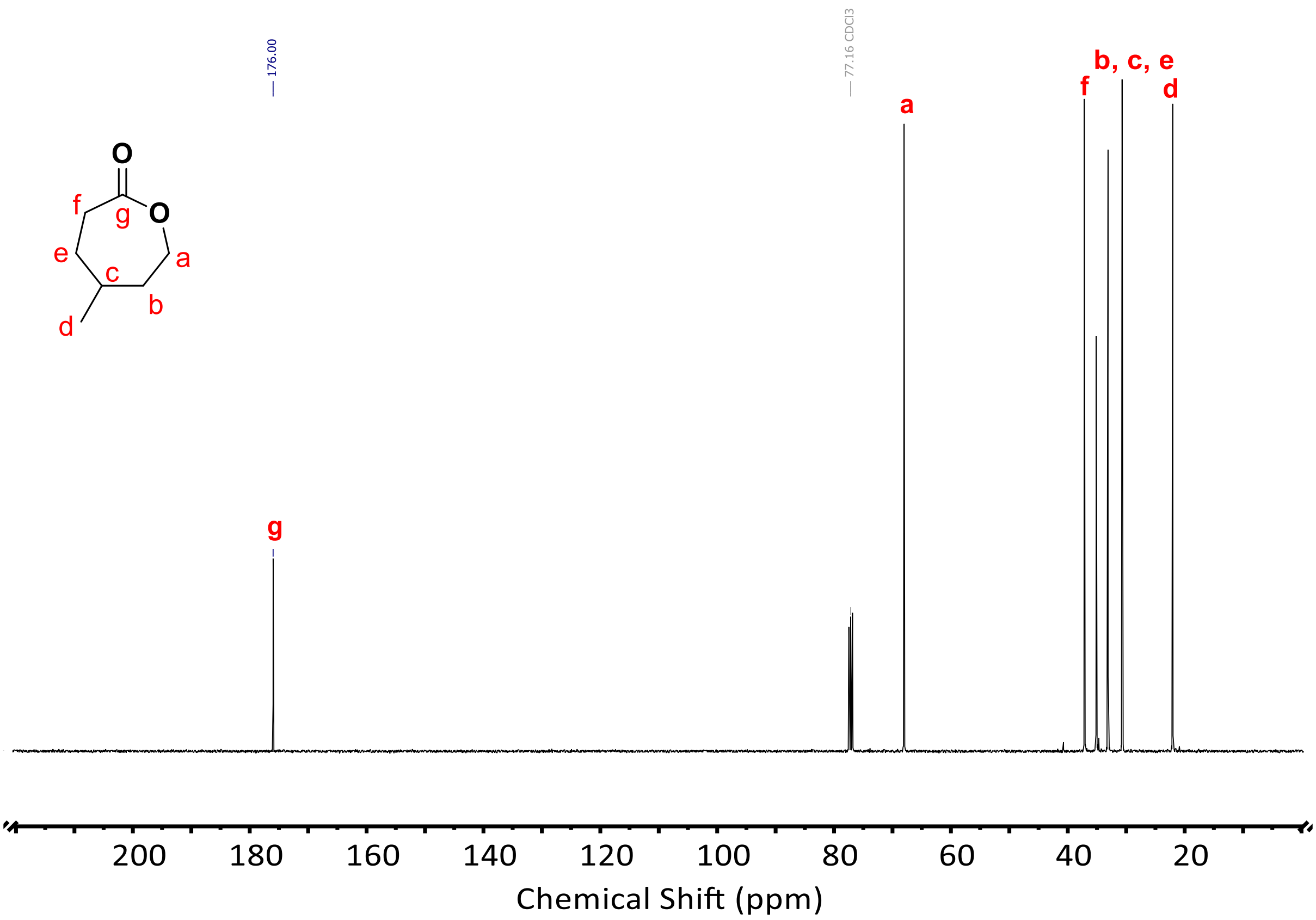

Fig. S72 $^{13}C\{^{1}H\}$ NMR spectrum (400 MHz, $CDCl_3$, 298 K) of MeCL.

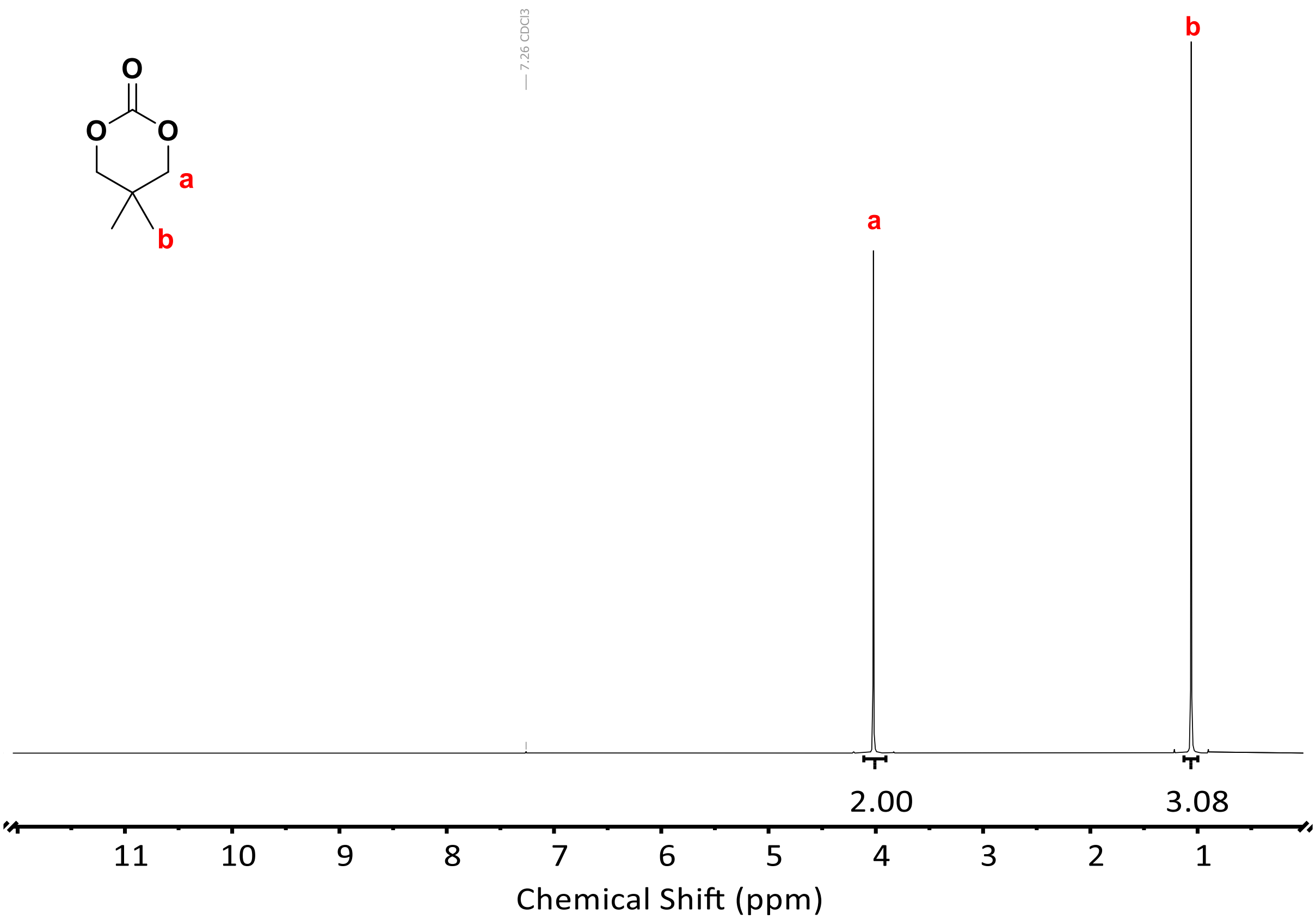

Fig. S73 ^{1}H NMR spectrum (400 MHz, $CDCl_3$, 298 K) of DTC.

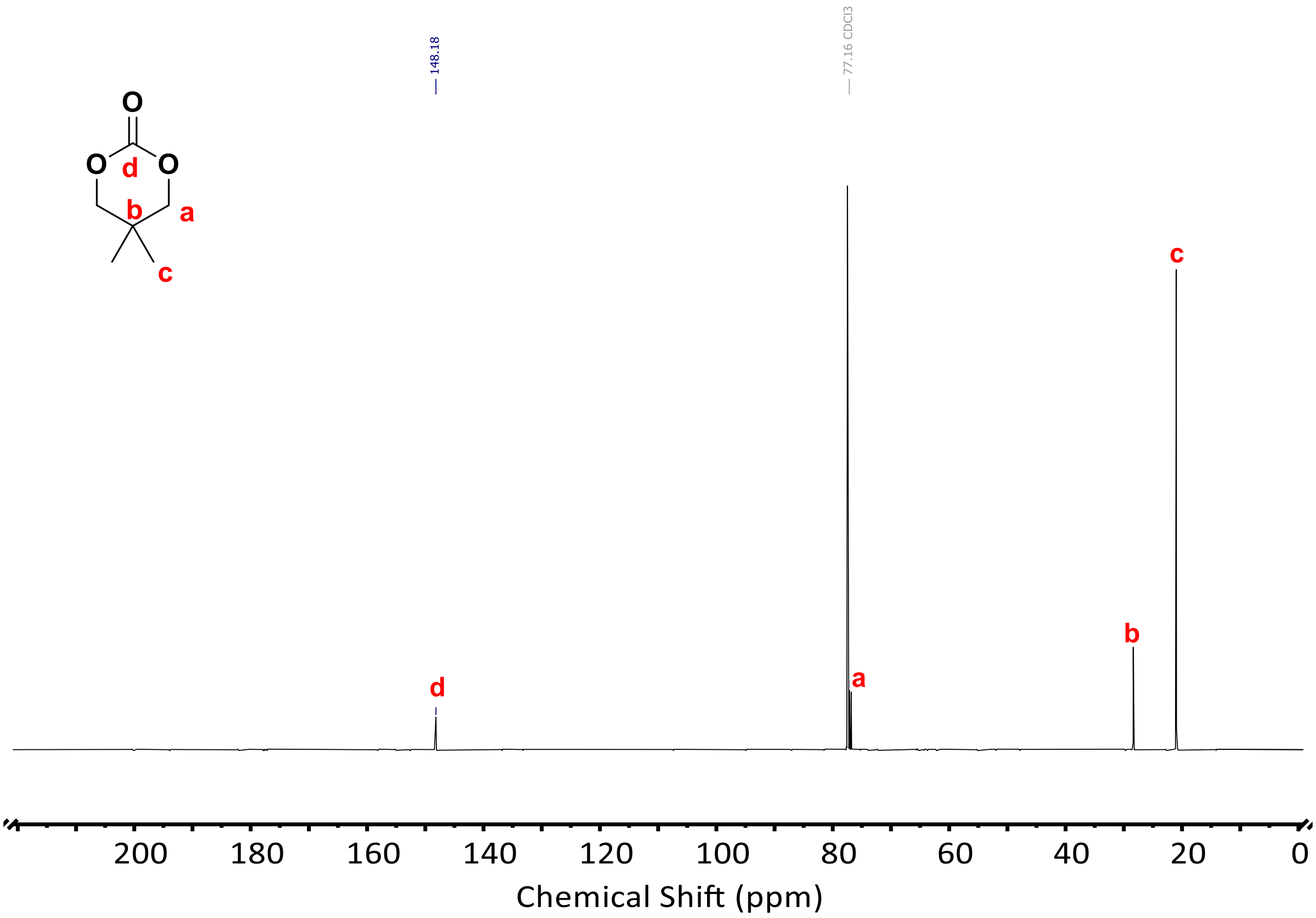

Fig. S74 $^{13}C\{^{1}H\}$ NMR spectrum (400 MHz, $CDCl_3$, 298 K) of DTC.

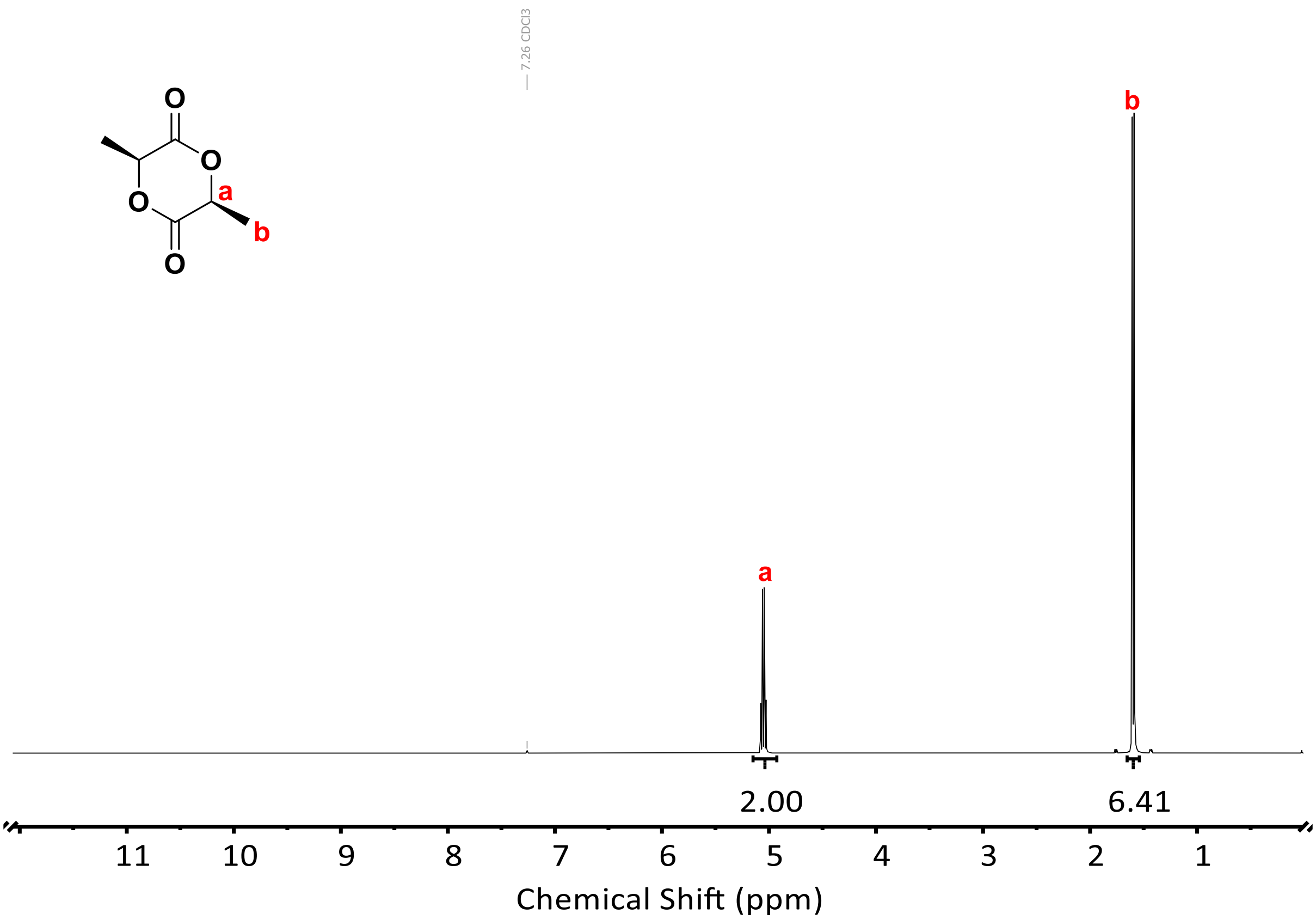

Fig. S75 ^{1}H NMR spectrum (400 MHz, $CDCl_3$, 298 K) of *L*-LA.

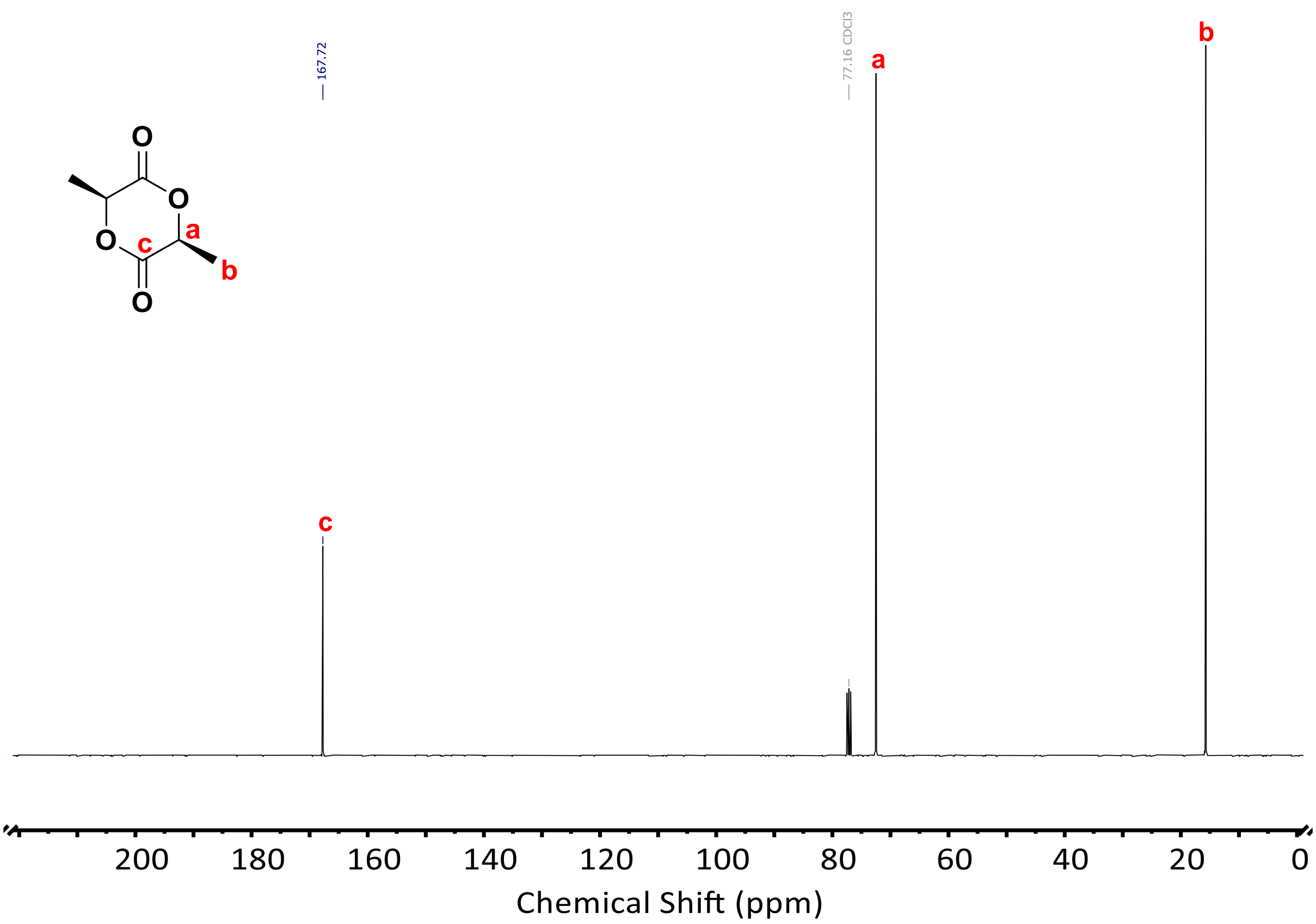

Fig. S76 $^{13}C\{^{1}H\}$ NMR spectrum (400 MHz, $CDCl_3$, 298 K) of *L*-LA.

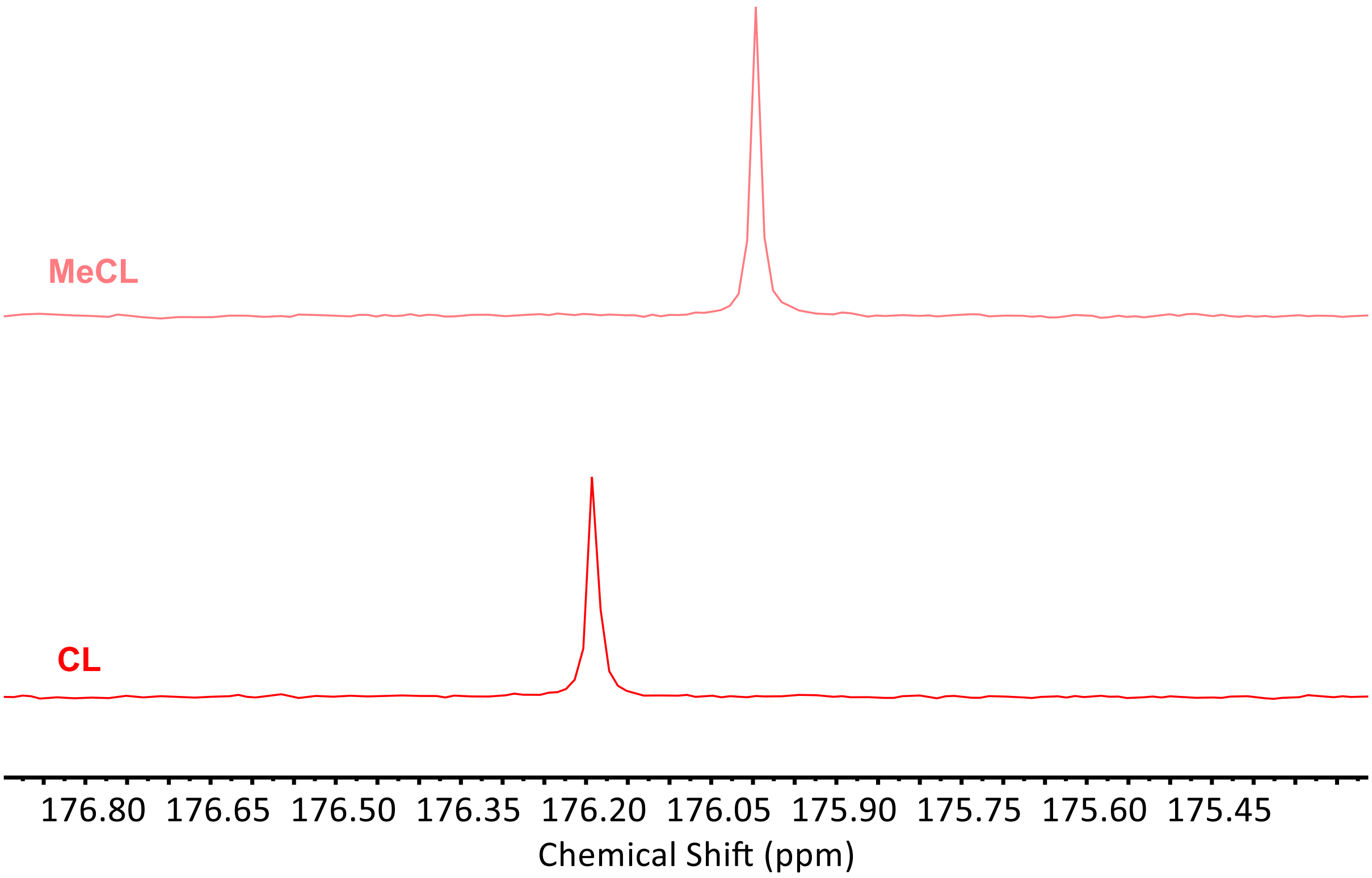

Fig. S77 Zoomed in stacked $^{13}C\{^1H\}$ NMR spectra (400 MHz, $CDCl_3$, 298 K) of MeCL and CL. The full spectrum for CL can be found in Fig. S70 and the full spectrum for MeCL can be found in Fig. S72.

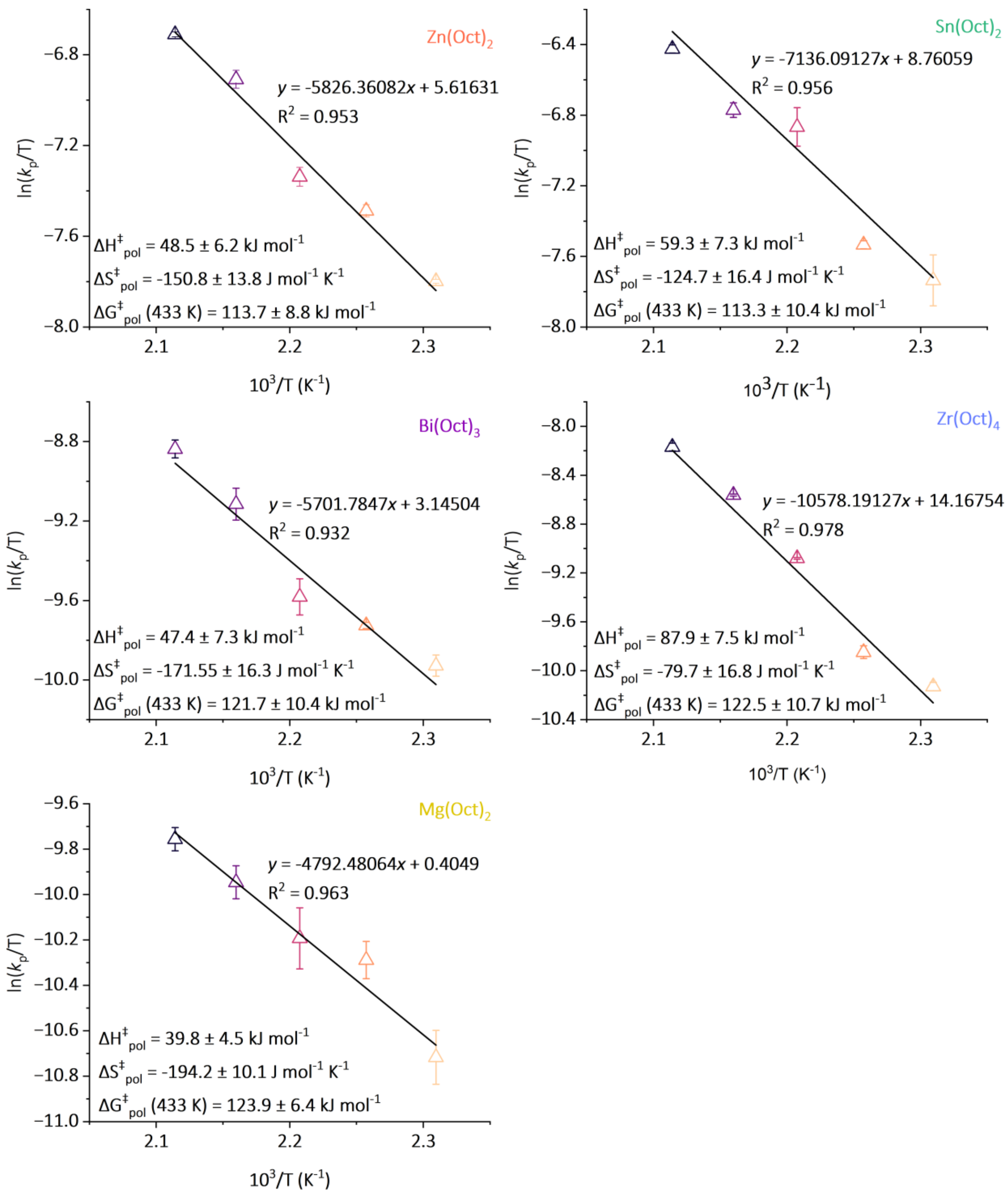

Fig. S78 Plots of ln(k_p/T) *vs*. 1/T as a part of Eyring analyses to determine transition state enthalpy ($\Delta H^{\ddagger}_{pol}$), entropy ($\Delta S^{\ddagger}_{pol}$) and Gibbs energy of activation ($\Delta G^{\ddagger}_{pol}$) for polymerization of VL, using each M(Oct)$_n$ (M= Zn(II), Sn(II), Bi(III), Zr(IV), Mg(II)) catalyst. Polymerization conditions: 1:10:1000 M(Oct)$_n$:BnOH:VL, [VL] = 6.25 M.

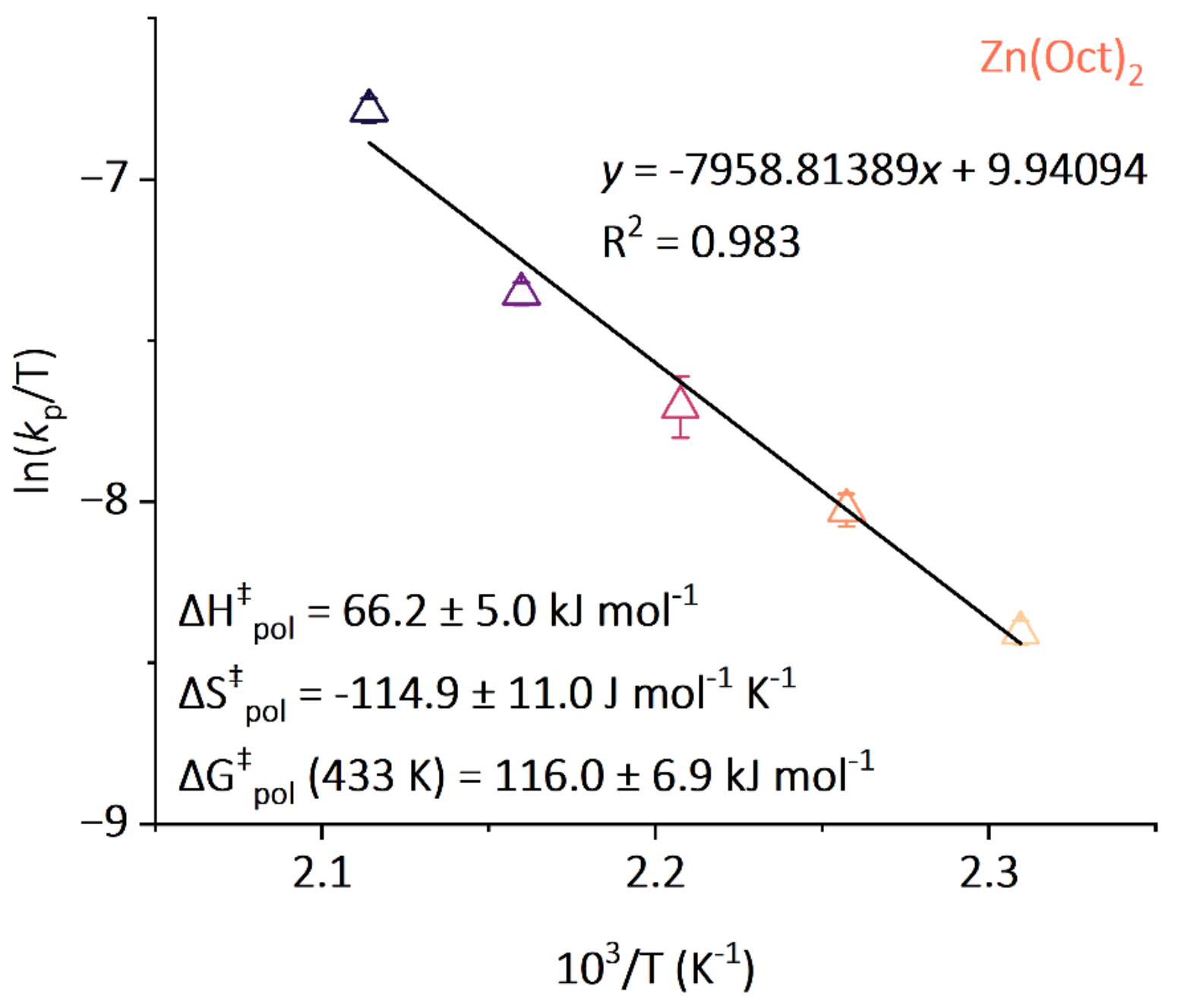

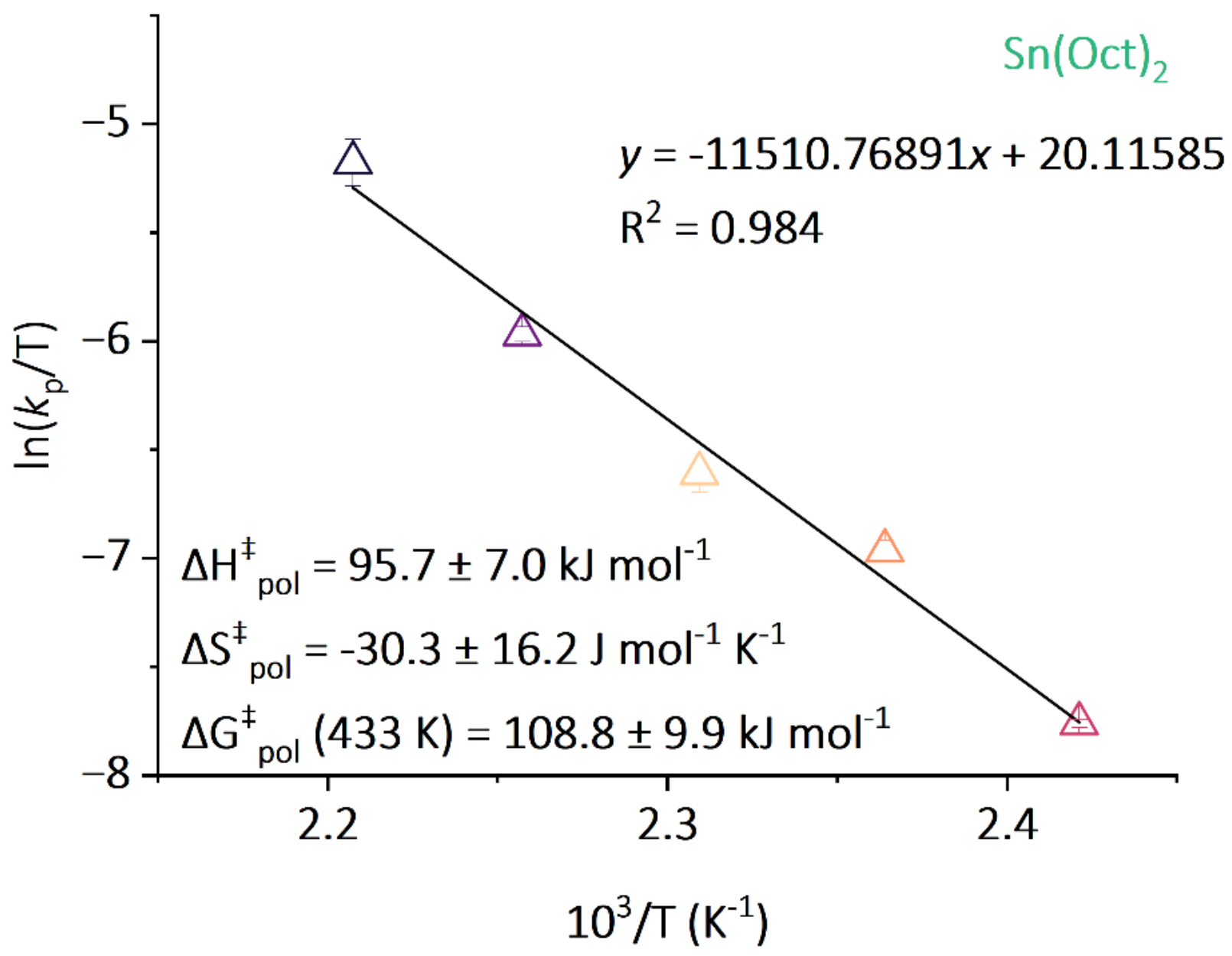

Fig. S79 Plots of ln(k_p/T) *vs*. 1/T as a part of Eyring analyses to determine transition state enthalpy ($\Delta H^{\ddagger}_{pol}$), entropy ($\Delta S^{\ddagger}_{pol}$) and Gibbs energy of activation ($\Delta G^{\ddagger}_{pol}$) for polymerization of CL, using the $Zn(Oct)_2$ and $Sn(Oct)_2$ catalysts. Polymerization conditions: 1:10:1000 $M(Oct)_n$:BnOH:CL, [CL] = 6.25 M.

Table S10 Polymerization Rate Data Table for the Polymerization of *L*-LA to PLLA using $Zn(Oct)_2$ and $Sn(Oct)_2$ catalysts. Polymerization conditions: 1:10:250 $M(Oct)_2$:BnOH:*L*-LA, [*L*-LA] = 1.56 M.

Temperature (°C)	Catalyst	k_{obs} / 10^{-3} (s^{-1})[a]	Av. k_{obs} / 10^{-3} (s^{-1})[a]	Conversion to polymer[b]	Av. Conversion to polymer	M_n [Đ] (kg mol^{-1})[c]
160	$Zn(Oct)_2$	1.12		0.80		
		0.81	0.98 ± 0.07	0.78	0.78 ± 0.01	7200 [1.26]
		1.01		0.78		
	$Sn(Oct)_2$	11.5		0.82		
		11.2	11.5 ± 0.12	0.82	0.82 ± 0.01	8600 [1.26]
		11.7		0.82		

[a]k_{obs} determined from the DSC isotherm at the temperature indicated: integration of the DSC normalized heat flow *vs*. time and normalizing to polymer conversion produced exponential fits, where the k_{obs} is taken from the exponential fitting of each line ($y = A - Be^{-k_{obs}x}$), with the average k_{obs} taken as the mean of the three repeats and error determined from the standard deviation of the mean of the three repeats (Fig. S80). [b]Conversion to polymer was calculated from ^1H NMR integrals of the *L*-LA and PLLA (Fig. S81). [c]Determined by GPC in THF using narrow dispersity polystyrene standards.

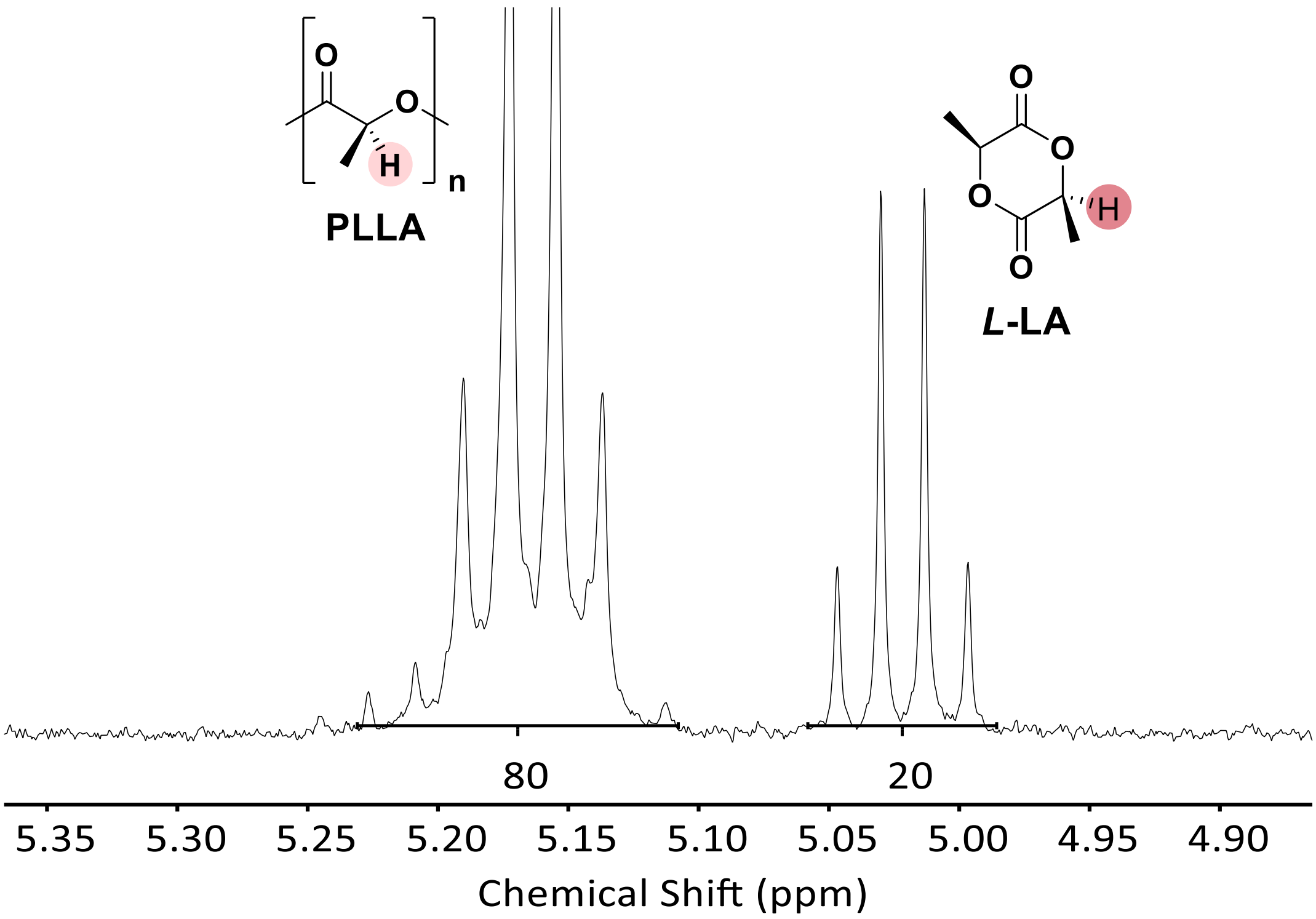

Fig. S80 Representative ^1H NMR spectrum (400 MHz, $CDCl_3$, 298 K) for calculation of conversion of *L*-LA to PLLA. Polymerization conditions: 1:10:250 $M(Oct)_2$:BnOH:*L*-LA, [*L*-LA] = 1.56 M, at 160 °C.

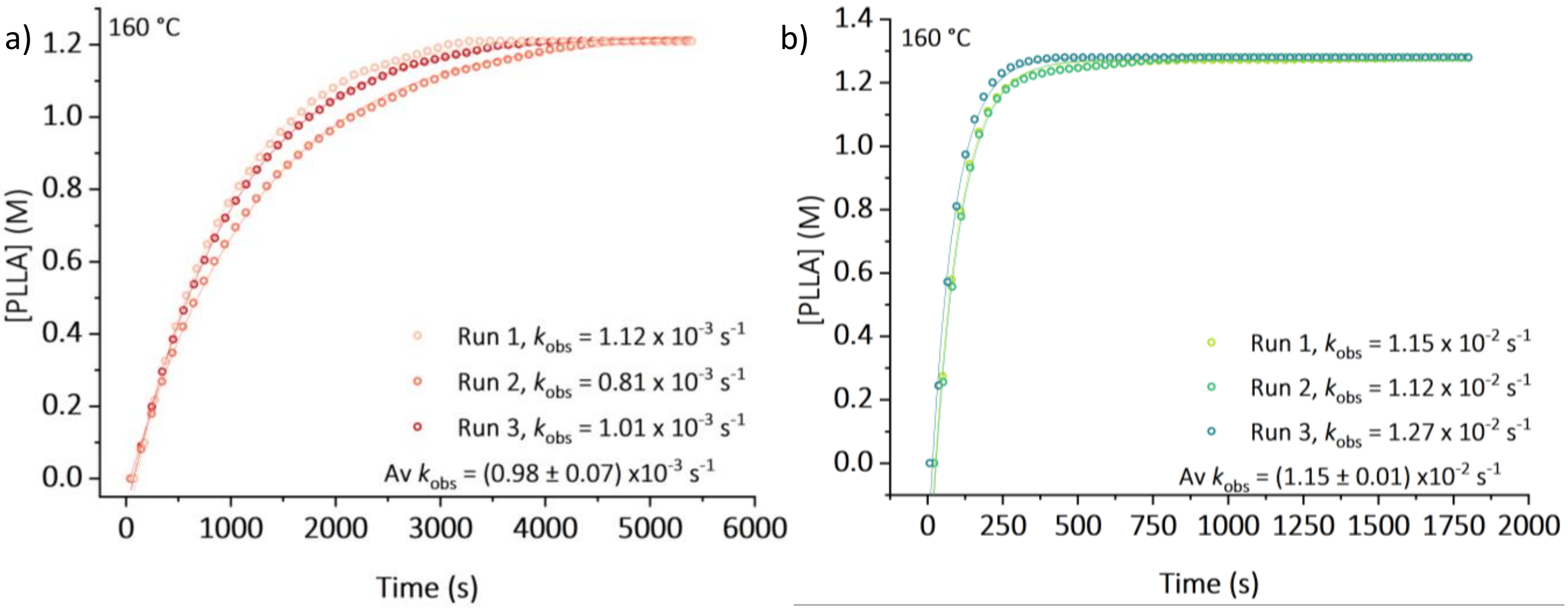

Fig. S81 Isothermal polymerization kinetic experiments, conducted on a DSC instrument, for polymerization of *L*-LA (conducted in triplicate). (a) Polymerization conditions: 1:10:250 Zn(Oct)$_2$:BnOH:*L*-LA, [*L*-LA] = 1.56 M, 160 °C. (b) Polymerization conditions: 1:10:250 Sn(Oct)$_2$:BnOH:*L*-LA, [*L*-LA] = 1.56 M, 160 °C.

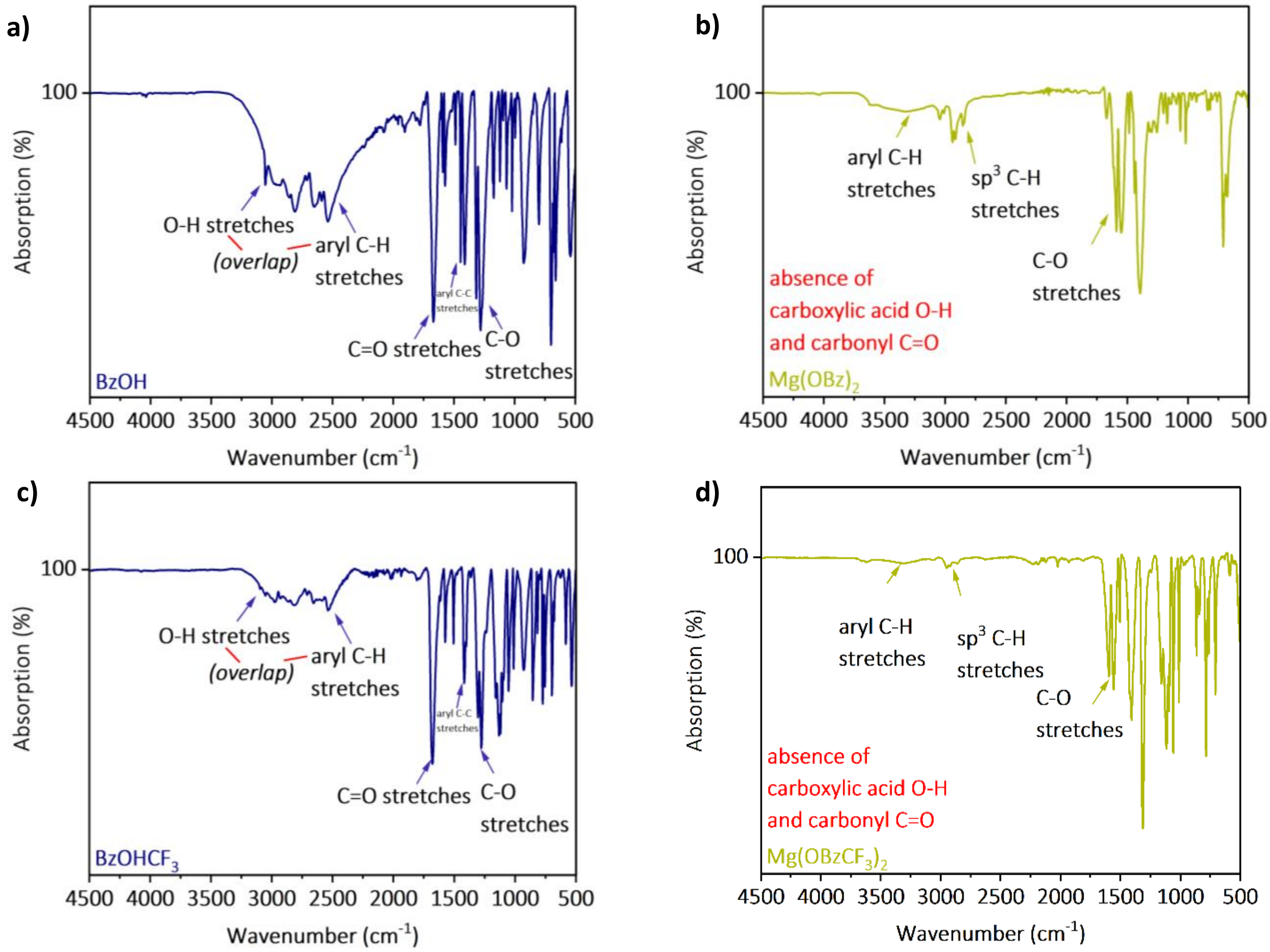

Fig. S82 IR Spectra **of the benzoic acid starting material and corresponding** bis **Mg(II) benzoate species. Disappearance of the C=O stretch and carboxylic acid O-H stretch present in the starting material**, **is in line with the formation of the Mg(II)**(carboxylate)$_2$ **species. a) BzOH. b) Mg(OBz)$_2$. c) F$_3$CBzOH. d) Mg(OBzCF$_3$)$_2$.**

a)

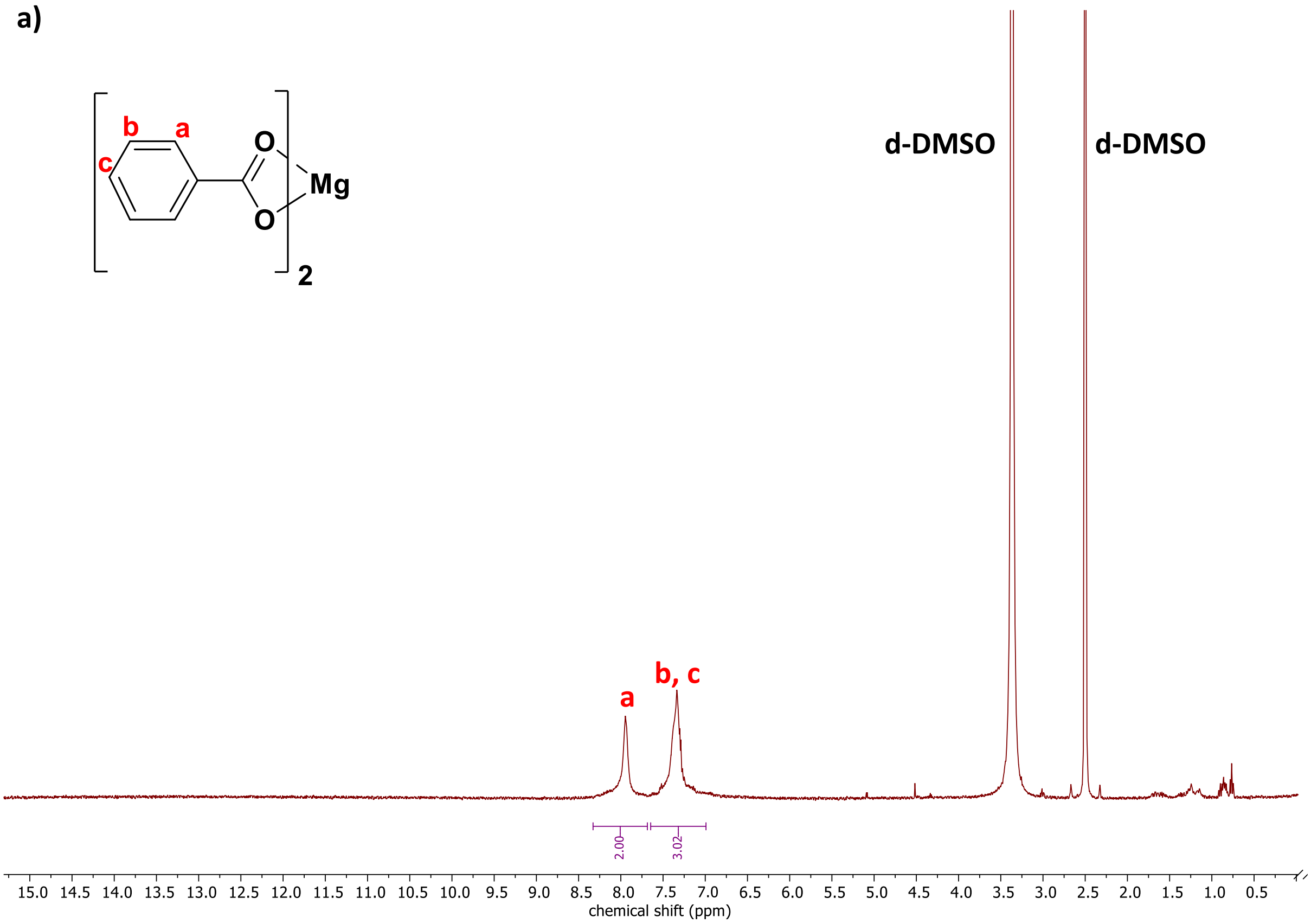

b
a
c
O
Mg
O
2
d-DMSO
d-DMSO
a
b, c
2.00
3.02
15.0 14.5 14.0 13.5 13.0 12.5 12.0 11.5 11.0 10.5 10.0 9.5 9.0 8.5 8.0 7.5 7.0 6.5 6.0 5.5 5.0 4.5 4.0 3.5 3.0 2.5 2.0 1.5 1.0 0.5
chemical shift (ppm)

b)

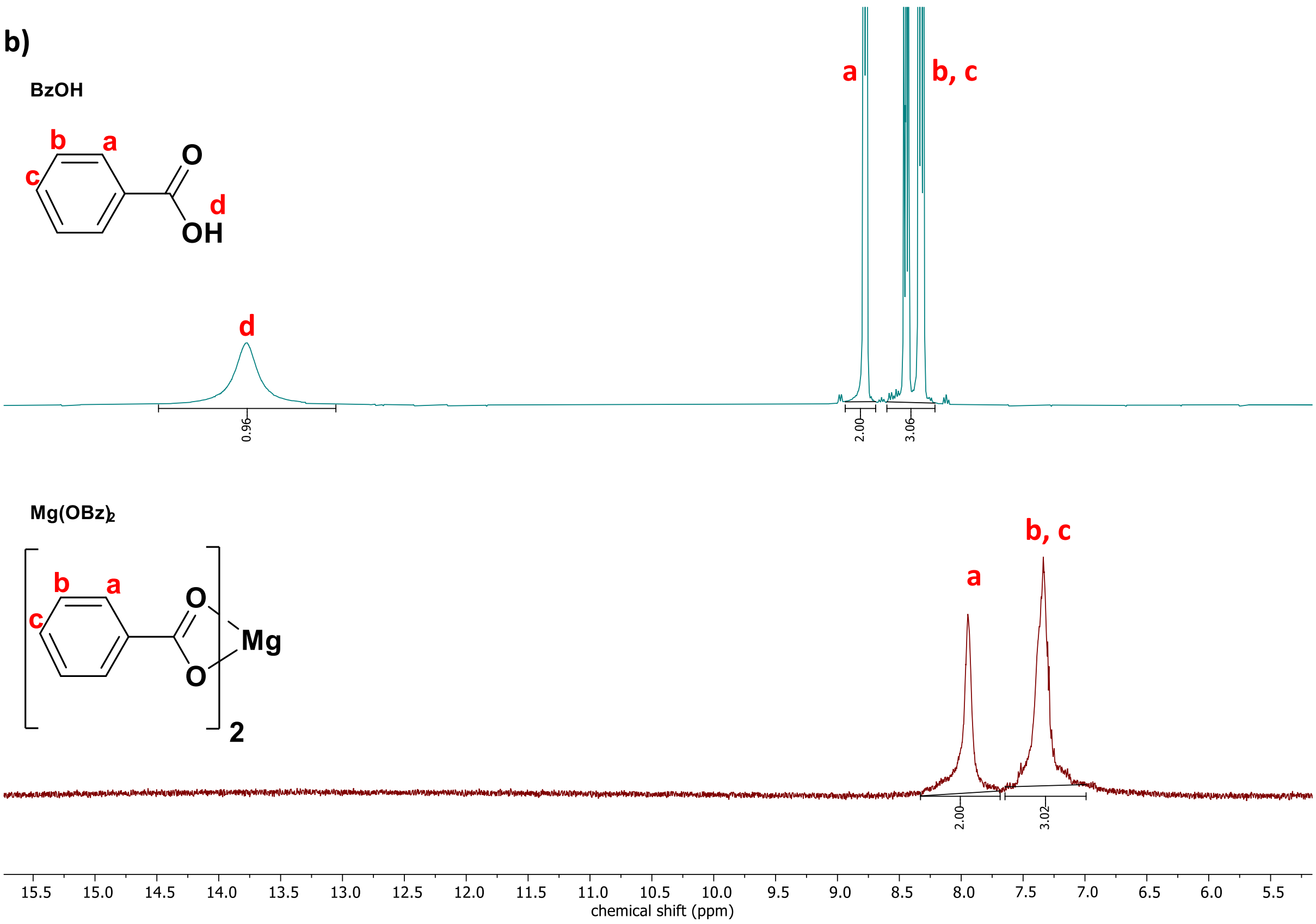

BzOH
b
a
c
O
d
OH
a
b, c
d
0.96
2.00
3.06
Mg(OBz)2
b
a
c
O
Mg
O
2
a
b, c
2.00
3.02
15.5 15.0 14.5 14.0 13.5 13.0 12.5 12.0 11.5 11.0 10.5 10.0 9.5 9.0 8.5 8.0 7.5 7.0 6.5 6.0 5.5
chemical shift (ppm)

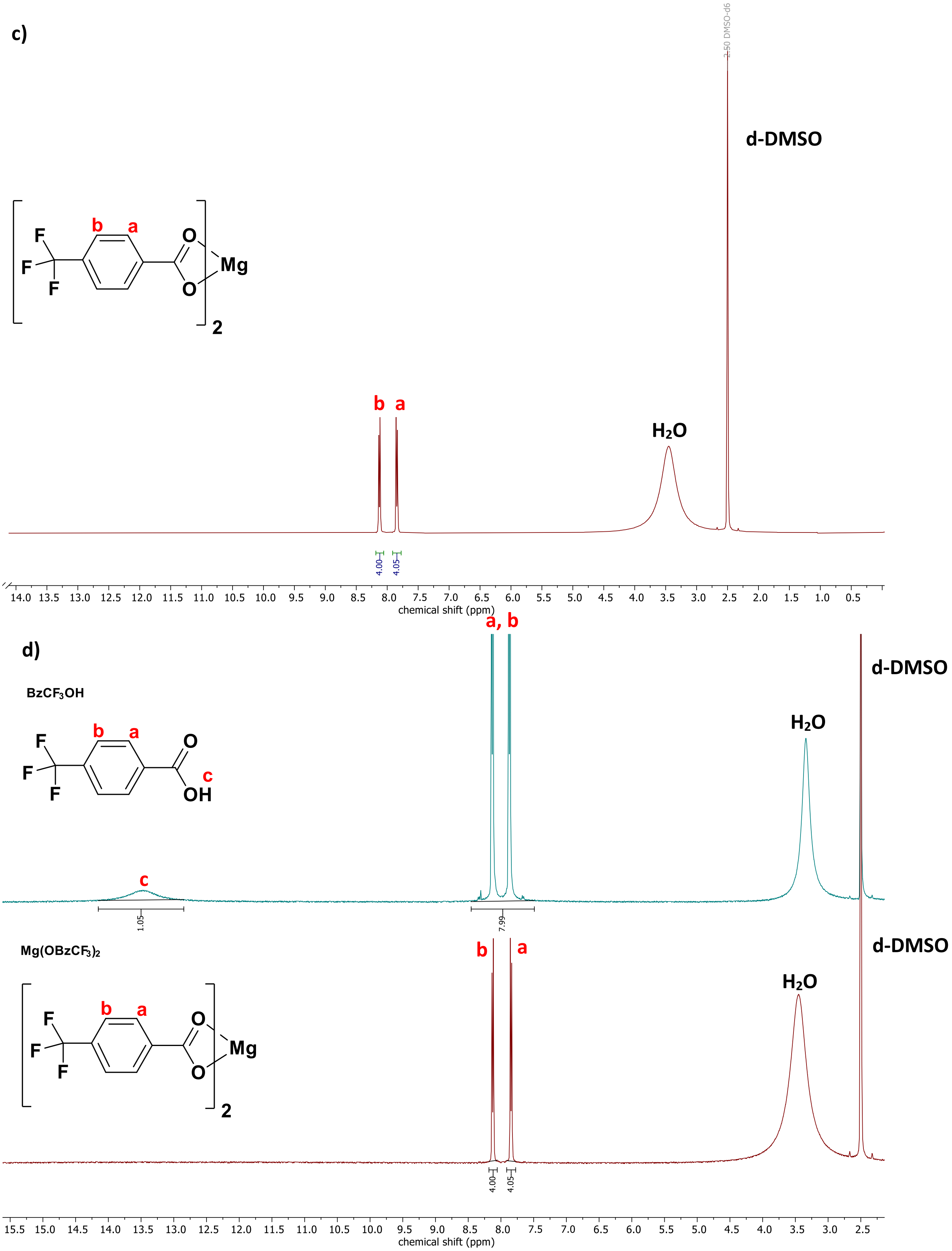

Fig. S83 a) ^1H NMR Spectra of the $Mg(OBz)_2$ catalyst. b) Zoomed-in stacked ^1H NMR spectra of the BzOH starting material and $Mg(OBz)_2$ product. c) ^1H NMR spectrum of $Mg(OBzCF_3)_2$ catalyst. d) Zoomed-in stacked ^1H NMR spectra of the F_3CBzOH starting material and $Mg(OBzCF_3)_2$ product. Disappearance of the carboxylic acid O-H proton and shifting of the aromatic signals is observed in line with formation of the magnesium carboxylate species.

Following the observation of an improved rate of reaction using $Mg(Bu)_2$ in combination with BzOH to form a $Mg(OBz)_2$ species, in comparison to the original $Mg(Oct)_2$ catalyst (Entries 1 and 2, Table S11), a control reaction using only $Mg(Bu)_2$ and 2-ethylhexanoic acid (OctOH), to preform an $Mg(Oct)_2$ species, was completed (Entry 3, Table S11).

Table S11 Polymerization Data for the Polymerization of VL using MgR_2 (R = Oct, OBz, $OBzCF_3$) catalysts. Polymerization conditions: 1:10:1000 $Mg(R)_2$:BnOH:VL, [VL] = 6.25 M.

Catalyst	Temperature (°C)	k_{obs} / 10^{-4} (s^{-1})[a]	Av. k_{obs} / 10^{-4} (s^{-1})[a]	Conversion to polymer[b]	Av. Conversion to polymer	M_n [Đ] (kg mol^{-1})[c]
$Mg(Oct)_2$	160	0.43<br>0.67<br>0.70	0.60 ± 0.07	0.78<br>0.99<br>0.66	0.81 ± 0.08	20.2 [1.54]
"$Mg(OBz)_2$" (1:2 $Mg(Bu)_2$: BzOH)	160	2.31<br>2.20<br>1.56	2.02 ± 0.19	0.64<br>0.63<br>0.62	0.63 ± 0.01	33.5 [1.25]
"$Mg(OBzCF_3)_2$" (1:2 $Mg(Bu)_2$: F_3CBzOH)	160	3.36<br>3.58<br>5.41	4.12 ± 0.53	0.61<br>0.63<br>0.68	0.64 ± 0.02	25.5 [1.45]
"$Mg(Oct)_2$" (1:2 $Mg(Bu)_2$:OctOH)	160	0.46<br>0.53<br>0.46	0.48 ± 0.02	0.72<br>0.61<br>0.53	0.62 ± 0.04	18.4 [1.52]

[a]k_{obs} determined from the DSC isotherm at the temperature indicated: integration of the DSC normalized heat flow *vs*. time and normalizing to polymer conversion produced exponential fits, where the k_{obs} is taken from the exponential fitting of each line ($y = A - Be^{-k_{obs}x}$), with the average k_{obs} taken as the mean of the three repeats and error determined from the standard deviation of the mean of the three repeats (Fig. S84). [b]Conversion to polymer was calculated from ^1H NMR integrals of the VL and PVL (Fig. S17). [c]Determined by GPC in THF using narrow dispersity polystyrene standards.

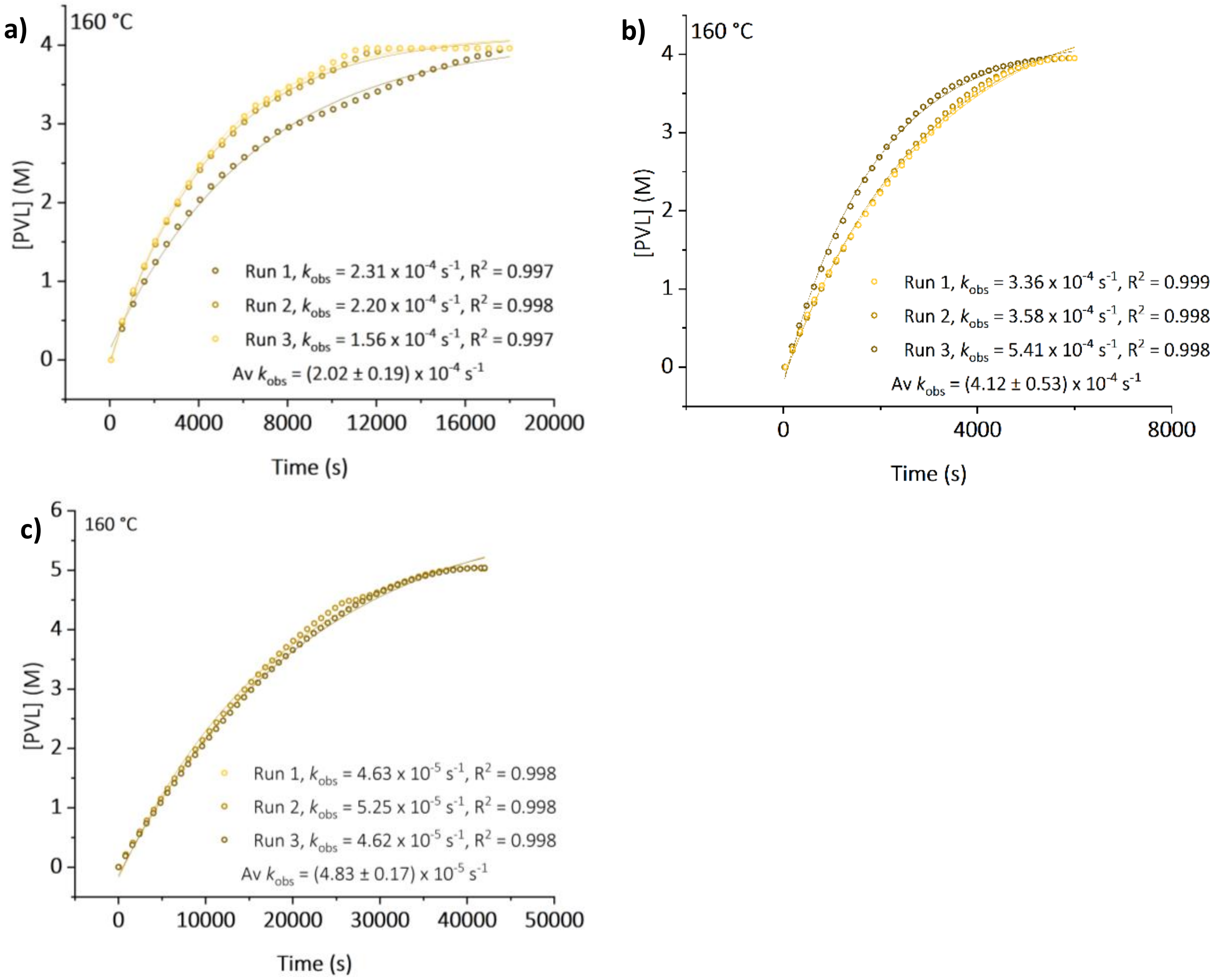

Fig. S84 Isothermal polymerization kinetic experiments, conducted on a DSC instrument, for polymerization of VL (conducted in triplicate). (a) Polymerization conditions: 1:2:10:1000 $Mg(Bu)_2$:BzOH:BnOH:VL, [VL] = 6.25 M, 160 °C. (b) Polymerization conditions: 1:2:10:1000 $Mg(Bu)_2$:F_3CBzOH:BnOH:VL, [VL] = 6.25 M, 160 °C. (c) Polymerization conditions: 1:2:10:1000 $Mg(Bu)_2$:OctOH:BnOH:VL, [VL] = 6.25 M, 160 °C.

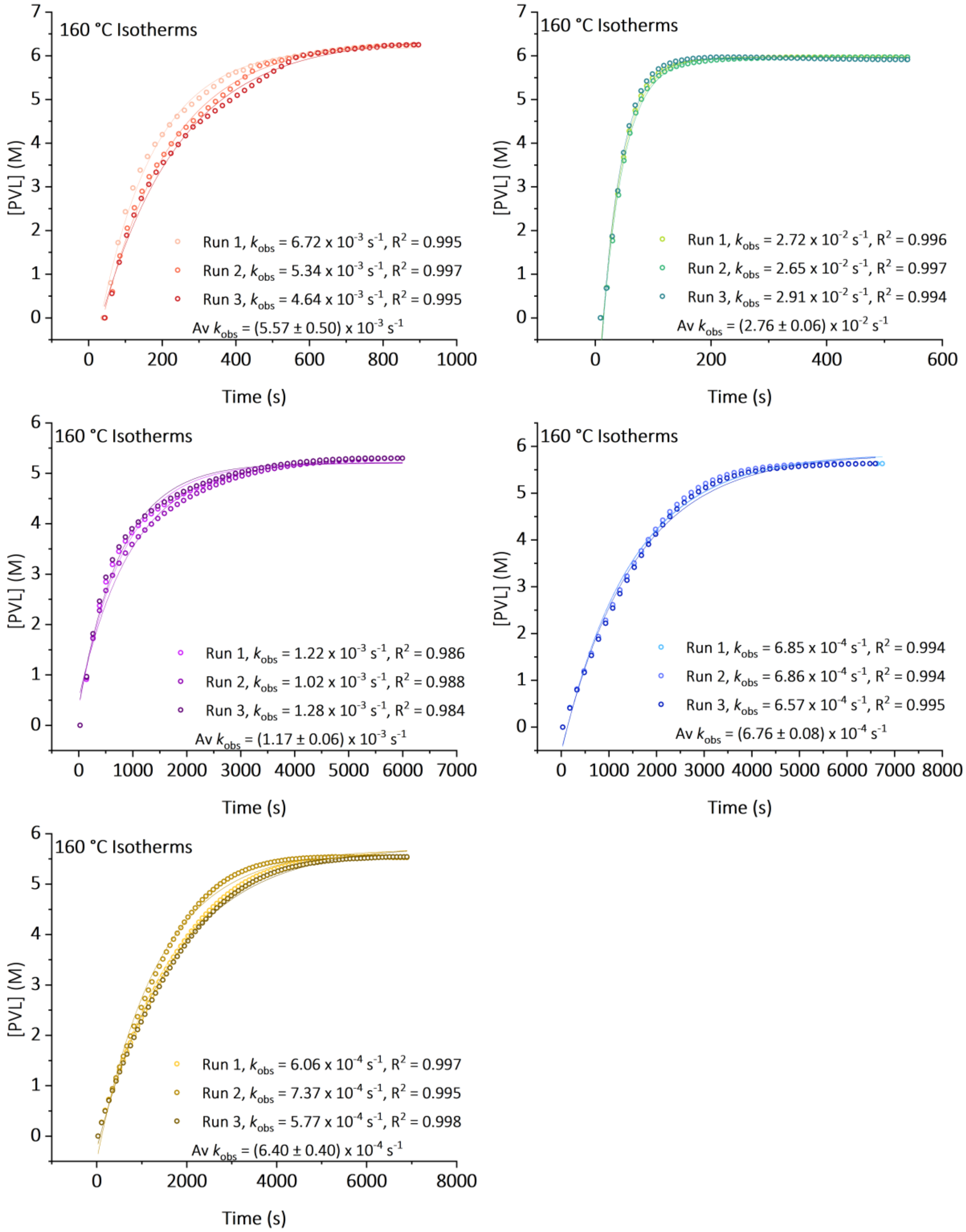

Fig. S85 Isothermal polymerization kinetic experiments, conducted on a DSC instrument, at 160 °C for the polymerization of VL with the $Zn(Oct)_2$ catalyst (plot in orange, top row, LHS), $Sn(Oct)_2$ catalyst (plot in green, top row, RHS), $Bi(Oct)_3$ catalyst (plot in purple, middle row, LHS), $Zr(Oct)_4$ catalyst (plot in blue, middle row, RHS), and $Mg(Oct)_2$ catalyst (plot in yellow, bottom row), run in neat VL. Polymerization conditions: 1:10:1000 $M(Oct)_n$:BnOH:VL.

Table S12 Polymerization data for polymerization of VL in absence of solvent. Polymerization conditions: 1:10:1000 M(Oct)$_n$:BnOH:VL, neat in VL.

Temperature (°C)	Catalyst	k_{obs} / 10^{-3} (s^{-1})[a]	Av. k_{obs} / 10^{-3} (s^{-1})[a]	Conversion to polymer[b]	Av. Conversion to polymer	M_n [Đ] (kg mol^{-1})[c]
160	Zn(Oct)$_2$	6.72<br>5.34<br>4.64	5.57 ± 0.50	1.00<br>0.99<br>0.99	1.00 ± 0.01	2.0 [1.86]
	Sn(Oct)$_2$	27.2<br>26.5<br>29.1	27.6 ± 0.63	0.93<br>0.96<br>0.97	0.95 ± 0.01	2.6 [1.46]
	Bi(Oct)$_3$	1.22<br>1.02<br>1.28	1.17 ± 0.06	0.83<br>0.86<br>0.85	0.85 ± 0.01	2.0 [1.24]
	Zr(Oct)$_4$	0.69<br>0.69<br>0.66	0.68 ± 0.008	0.89<br>0.91<br>0.90	0.90 ± 0.01	3.0 [1.44]
	Mg(Oct)$_2$	0.61<br>0.74<br>0.58	0.64 ± 0.04	0.88<br>0.89<br>0.88	0.89 ± 0.01	1.8 [1.50]

[a]k_{obs} determined from the DSC isotherm at the temperature indicated: integration of the DSC normalized heat flow *vs*. time and normalizing to polymer conversion produced exponential fits, where the k_{obs} is taken from the exponential fitting of each line ($y = A - Be^{-k_{obs}x}$), with the average k_{obs} taken as the mean of the three repeats and error determined from the standard deviation of the mean of the three repeats (Fig. S85). [b]Conversion to polymer was calculated from ^{1}H NMR integrals of the VL and PVL (Fig. S17). [c]Determined by GPC in THF using narrow dispersity polystyrene standards.

4. References

(1) Watts, A.; Kurokawa, N.; Hillmyer, M. A. Strong, Resilient, and Sustainable Aliphatic Polyester Thermoplastic Elastomers. *Biomacromolecules* **2017**, *18* (6), 1845–1854. DOI: 10.1021/acs.biomac.7b00283.
(2) Olsson, J. V.; Hult, D.; Cai, Y.; García-Gallego, S.; Malkoch, M. Reactive imidazole intermediates: simplified synthetic approach to functional aliphatic cyclic carbonates. *Polym. Chem.* **2014**, *5* (23), 6651–6655.
(3) McGuire, T. M.; Ning, D.; Williams, C. K. Using Differential Scanning Calorimetry to Accelerate Polymerization Catalysis: A Toolkit for Miniaturized and Automated Kinetics Measurements. *ACS Catal.* **2025**, *15* (9), 6760–6771. DOI: 10.1021/acscatal.5c01758.
(4) Kumar, A.; Blakemore, J. D. On the Use of Aqueous Metal-Aqua pKa Values as a Descriptor of Lewis Acidity. *Inorg. Chem.* **2021**, *60* (2), 1107–1115. DOI: 10.1021/acs.inorgchem.0c03239.
(5) Golwankar, R. R.; Curry, T. D., II; Paranjothi, C. J.; Blakemore, J. D. Molecular Influences on the Quantification of Lewis Acidity with Phosphine Oxide Probes. *Inorg. Chem.* **2023**, *62* (25), 9765–9780. DOI: 10.1021/acs.inorgchem.3c00084.
(6) Neufeld, R.; Stalke, D. Accurate molecular weight determination of small molecules via DOSY-NMR by using external calibration curves with normalized diffusion coefficients. *Chem. Sci.* **2015**, *6* (6), 3354–3364. DOI: 10.1039/C5SC00670H.
(7) Shannon, R. D. Revised effective ionic radii and systematic studies of interatomic distances in halides and chalcogenides. *Acta Crystallogr., Sect. A.* **1976**, *32* (5), 751–767. DOI: 10.1107/S0567739476001551.
(8) Sidey, V. On the effective ionic radii for the tin(II) cation. *J. Phys. Chem. Solids* **2022**, *171*, 110992. DOI: 10.1016/j.jpcs.2022.110992.
(9) Kepp, K. P. A Quantitative Scale of Oxophilicity and Thiophilicity. *Inorg. Chem.* **2016**, *55* (18), 9461–9470. DOI: 10.1021/acs.inorgchem.6b01702.
(10) Kowalski, A.; Duda, A.; Penczek, S. Mechanism of Cyclic Ester Polymerization Initiated with Tin(II) Octoate. 2. Macromolecules Fitted with Tin(II) Alkoxide Species Observed Directly in MALDI−TOF Spectra. *Macromolecules* **2000**, *33* (3), 689–695. DOI: 10.1021/ma9906940.
(11) Duda, A.; Penczek, S. Kinetics of polymerization involving reversible deactivation due to aggregation of active centers. Analytical vs. numerical solution for the ε-caprolactone/dialkylaluminium alkoxide system. *Macromol. Rapid Commun.* **1994**, *15* (6), 559–566. DOI: 10.1002/marc.1994.030150617.
